# Identification and validation of critical alternative splicing events and splicing factors in gastric cancer progression

**DOI:** 10.1111/jcmm.15835

**Published:** 2020-09-16

**Authors:** Haoran Feng, Zhijian Jin, Kun Liu, Yi Peng, Songyao Jiang, Changgang Wang, Jiele Hu, Xiaoyun Shen, Weihua Qiu, Xi Cheng, Ren Zhao

**Affiliations:** ^1^ Department of General Surgery Ruijin Hospital Shanghai Jiao Tong University School of Medicine Shanghai China; ^2^ Department of General Surgery Ruijin Hospital North Shanghai Jiao Tong University School of Medicine Shanghai China; ^3^ Shanghai Institute of Digestive Surgery Ruijin Hospital Shanghai Jiao Tong University School of Medicine Shanghai China

**Keywords:** alternative splicing, gastric cancer, invasion, mRNA, prognosis

## Abstract

Gene expression and alternative splicing (AS) interact in complex ways to regulate biological process which is associated with cancer development. Here, by integrated analysis of gene expression and AS events, we aimed to identify the hub AS events and splicing factors relevant in gastric cancer development (GC). RNA‐seq data, clinical data and AS events of 348 GC samples were obtained from the TCGA and TCGASpliceSeq databases. Cox univariable and multivariable analyses, KEGG and GO pathway analyses were performed to identify hub AS events and splicing factor/spliceosome genes, which were further validated in 53 GCs. By bioinformatics methods, we found that gene AS event‐ and gene expression‐mediated GC progression shared the same mechanisms, such as PI3K/AKT pathway, but the involved genes were different. Though expression of 17 hub AS events were confirmed in 53 GC tissues, only 10 AS events in seven genes were identified as critical candidates related to GC progression, notably the AS events (Exon Skip) in CLSTN1 and SEC16A. Expression of these AS events in GC correlated with activation of the PI3K/AKT pathway. Genes with AS events associated with clinical parameters and prognosis were different from the genes whose mRNA levels were related to clinical parameters and prognosis. Besides, we further revealed that QKI and NOVA1 were the crucial splicing factors regulating expression of AS events in GC, but not spliceosome genes. Our integrated analysis revealed hub AS events in GC development, which might be the potential therapeutic targets for GC.

## 1 INTRODUCTION

Gastric cancer is one of the most common cancers worldwide, especially in developing countries, and causes 783 000 cancer‐related deaths globally.^1^ Cancer growth, invasion and metastasis are critical steps in GC progression^2^, ^3^, ^4^ and involve complex regulatory mechanisms among protein‐coding genes, lncRNAs and microRNAs. Alternative splicing (AS) is an important post‐transcriptional regulatory mechanism involved in protein diversity and is involved in cancer progression.^5^ Distinct pre‐mRNA AS events can produce protein isoforms with diverse structures and functions,^6^ which can affect mechanisms regulating cancer progression. Investigating the role of AS in GC progression may clarify mechanisms of tumour progression and may uncover novel therapeutic opportunities.

There are seven basic splicing patterns, including exon skipping (ES), alternate acceptor sites (AA), mutually exclusive exons (ME), alternate donor sites (AD), alternate terminator (AT), alternate promoter (AP) and retained intron (RI). Approximately 95% of genes in the human genome undergo AS.^7^ Expression of aberrant splicing can promote cancer progression by activating cancer‐related pathways.^8^ Li et al revealed an aberrantly spliced transcript of FGFR3, termed FGFR3Δ7‐9, which lacks exons encoding the immunoglobulin‐like III domain; this isoform exerted potent oncogenic functions, enhancing migration and lung metastatic capacity in hepatocellular carcinoma via activation of the MAPK and PI3K/AKT pathways.^9^ The high incidence of AS events in cancer increases the complexity of mechanisms of cancer initiation and progression, and focused evaluation of AS events may not comprehensively describe how AS can regulate cancer biology. Fortunately, with the development of next‐generation sequencing technology, we can perform in‐depth and comprehensive analysis of AS events in cancer.^10^ Comprehensive evaluation of AS events has been performed in several cancer types, including lung,^11^ ovarian^12^ and bladder carcinomas,^13^ as well as in gastrointestinal adenocarcinomas.^14^ Integrated analysis of gene expression and AS may facilitate better understanding of mechanisms of cancer progression,^15^, ^16^, ^17^ because gene expression and AS events could interact in complex mutual regulation.^18^, ^19^, ^20^

In this study, we performed integrated analysis of gene expression and AS in GC. We identified genes with prognostic mRNA expression or/and AS events, and we performed GO and KEGG pathway analysis to identify mechanisms of gene expression‐ and AS‐mediated GC progression. We further established a Cox regression model to compare the prognostic ability between AS events and gene expression. By constructing co‐expression networks between gene expression and AS events, we identified the hub AS events associated with cancer progression and hub splicing factors or/and spliceosome genes that regulated expression of AS events. By analysing the relationships between AS events and TNM staging, Lauren classification and cancer stromal score, we revealed associations between hub AS events and GC invasion. Finally, further in vivo validation was performed on a cohort of 53 GC tissue samples. In summary, our study identified and validated hub AS events involved in GC progression, which may be potential therapeutic targets for GC.

## 2 METHODS AND MATERIALS

### 2.1 Data extraction

We downloaded RNA‐seq row count data for 374 GC samples from the TCGA database (https://tcga‐data.nci.nih.gov/tcga/). RNA‐seq data were normalized by calculating Fragments Per Kilobase of exon model per Million mapped reads (FPKM). The formula was FPKM = (1 000 000*A)/(mapped reads*gene length/1000) (A: read count of a gene). Then, the gene expression value was transformed by Log2(FPKM + 1). Using the TCGASpliceSeq database (http://projects.insilico.us.com/TCGASpliceSeq/index.jsp), we obtained data for AS events in 412 GC samples, with 75% of samples with PSI Value. Finally, a cohort of 348 patient samples with gene expression data, AS event data and matched clinical data were evaluated in this study.

### 2.2 Cox univariate and multivariate analysis

Cox univariate regression analysis was performed on genes and all seven types of AS events using the “survival” package in R. Genes whose AS events or mRNA levels associated with prognosis (*P* < 0.01) were further used to conduct Cox multivariate analysis and construct Cox regression model. Finally, the risk score was calculated for every case.

### 2.3 GO and KEGG pathway analysis

Genes with prognostic AS events or prognosis‐related mRNA expression (*P* < 0.05) were utilized to performed GO and KEGG pathway analysis in the Metascape database (http://metascape.org/gp/index.html#/main/step1). The enriched GO terms or KEGG pathways with *P* < 0.01 were displayed.

### 2.4 Co‐expression network analysis

In order to explore the relationships between gene expression and AS events, Spearman correlation coefficients were calculated. Genes with paired gene expression and AS events with correlation *P* < 0.01 and R > 0.3 were imported into Cytoscape software (version 3.6.0, Palo Alto, CA, USA). Hub genes or AS events were identified according to the degree of interaction. Splicing factor genes were downloaded from SpliceAid 2 database (http://193.206.120.249/splicing_tissue.html), and spliceosome genes were downloaded from KEGG database (https://www.kegg.jp/kegg/).

### 2.5 Patient characteristics and GC tissue specimens

We acquired a total of 53 fresh GC specimens for this study, including T1 GCs (n = 12), T2 GCs (n = 16), T3 GCs (n = 12) and T4 GCs (n = 13). The GC specimens were obtained from GC patients who underwent gastrectomy from 2013 to 2014 in Ruijin Hospital of Shanghai Jiaotong University. Patients were diagnosed with GC by histopathology and did not receive any adjuvant treatment prior to surgery. These GC tissues were stored at −80°C. All patients provided signed informed consent. The stage of GC was determined in accordance with the 8th edition of the UICC/AJCC cancer staging manual.^21^ Follow‐up was conducted on these 53 GC patients, with an average follow‐up period of 48 months (range: 6‐60 months). This study was approved by the Ethical Review Committee of Ruijin Hospital of Shanghai Jiaotong University. Clinicopathological characteristics of the 53 GC patients are summarized in Table 1.

**TABLE 1:** Clinicopathological characteristics of 53 GC patients included in this study

Parameters	Case number (53)
Gender	
Male	35
Female	18
Age	
≤60	35
>60	18
Location	
Stomach	10
Gastric body	23
Gastric antrum	20
Extent of invasion	
T1	12
T2	16
T3	12
T4	13
Lymphatic metastasis	
N0	29
N1 + N2 + N3	24
Metastasis	
M0	52
M1	1
Lauren's type	
Intestinal	39
Diffuse	9
Mix	5
Differentiation	
High	4
Middle	12
Low	37
Borrmann	
I	8
II	20
III	24
IV	1
Microsatellite instability	
MSS	49
MSI‐H	4

### 2.6 RT‐PCR and quantitative analysis for AS

Total RNA was extracted from fresh GC tissue with TRIzol reagent (Invitrogen, Thermo Fisher Scientific, Waltham, MA, USA). Subsequent cDNA synthesis was performed with a reverse transcription kit HiScript II Q Select RT SuperMix for qPCR (Vazyme, Nanjing, China), according to the manufacturer's instructions. Synthesized cDNA was PCR‐amplified for 40 cycles (step 1:95°C at 5 minutes; step 2:40 cycles at 94°C for 20 seconds, 60°C for 30 seconds and 72°C for 30 seconds; step 3:72°C at 5 minutes) using 2× PCR Master Mix (LifeFeng, Shanghai, China). GAPDH was used as an internal control. In order to quantify AS for every gene, we designed primers across the skipped exon for the ES type of AS. To quantify the results of the RT‐PCR, we calculated grey value for every WT (wild type) and AS band using ImageJ software (National Institutes of Health). According to the definition of PSI values in TCGASpliceSeq database, we calculated the relative PSI value of every AS event as WT/(WT + AS).^22^ To evaluate the AT type of AS, we designed primers located at the terminated exon and the near exon preceding the terminated exon; the relative PSI value of AS events was calculated as the ratio of grey value of the terminated exon and the near exon ahead of the terminated exon. In order to quantify the total mRNA level of genes amplified by the primers, we also calculated the grey value of all brands (including WT and AS) by normalizing to GAPDH. The primer sequences for AS are illustrated in Table S1.

### 2.7 Quantitative RT‐PCR analysis for genes encoding splicing factors

Total RNA extraction and cDNA synthesis were performed as described above. The mRNA expression of genes encoding splicing factors were measured using AceQ^®^ Universal SYBR qPCR Master Mix (Vazyme) on an Applied Biosystems 7900HT sequence detection system (Applied Biosystems, Waltham, MA, USA). The procedures for quantitative RT‐PCR were conducted as described above. GAPDH was used as an internal control, and relative expression levels of mRNA were evaluated using the 2^−ΔΔCt^ method.

### 2.8 Western blot analysis

Total protein was extracted from 13 selected GC tissues, and Western blot performed as previously described.^23^ The antibodies used in this study were anti‐AKT (1:1000), anti‐p‐AKT (1:1000), anti‐ERK (1:1000), anti‐p‐ERK (1:1000), anti‐STAT3 (1:1000), anti‐p‐STAT3 (1:1000) (all from Cell Signaling Technology, Boston, MA, USA) and GAPDH (Abcam, 1:10 000, Cambridge, UK). The grey values of protein bands were calculated using ImageJ software (National Institutes of Health).

### 2.9 Statistical analysis

Heatmaps were plotted using the “pheatmap” package in R. The stromal score of every GC sample was calculated by using the “estimate” package in R. Other statistical analyses were conducted using the GraphPad Prism 6.0 software, including t test, K‐M analysis, survival curve generation and ROC curve analysis. For prognostic analysis of splicing factors and stromal scores, an optimal cut‐off value was calculated to divide all GC cases into high and low groups. In order to assess the prognostic significance of the Cox regression model, a median value was set to determine GC cases as high and low groups, according to the risk scores. *P* values of *P* < 0.05 were considered statistically significant.

## 3 RESULTS

### 3.1 Prognostic AS and gene expression events in GC

**FIGURE 1:**
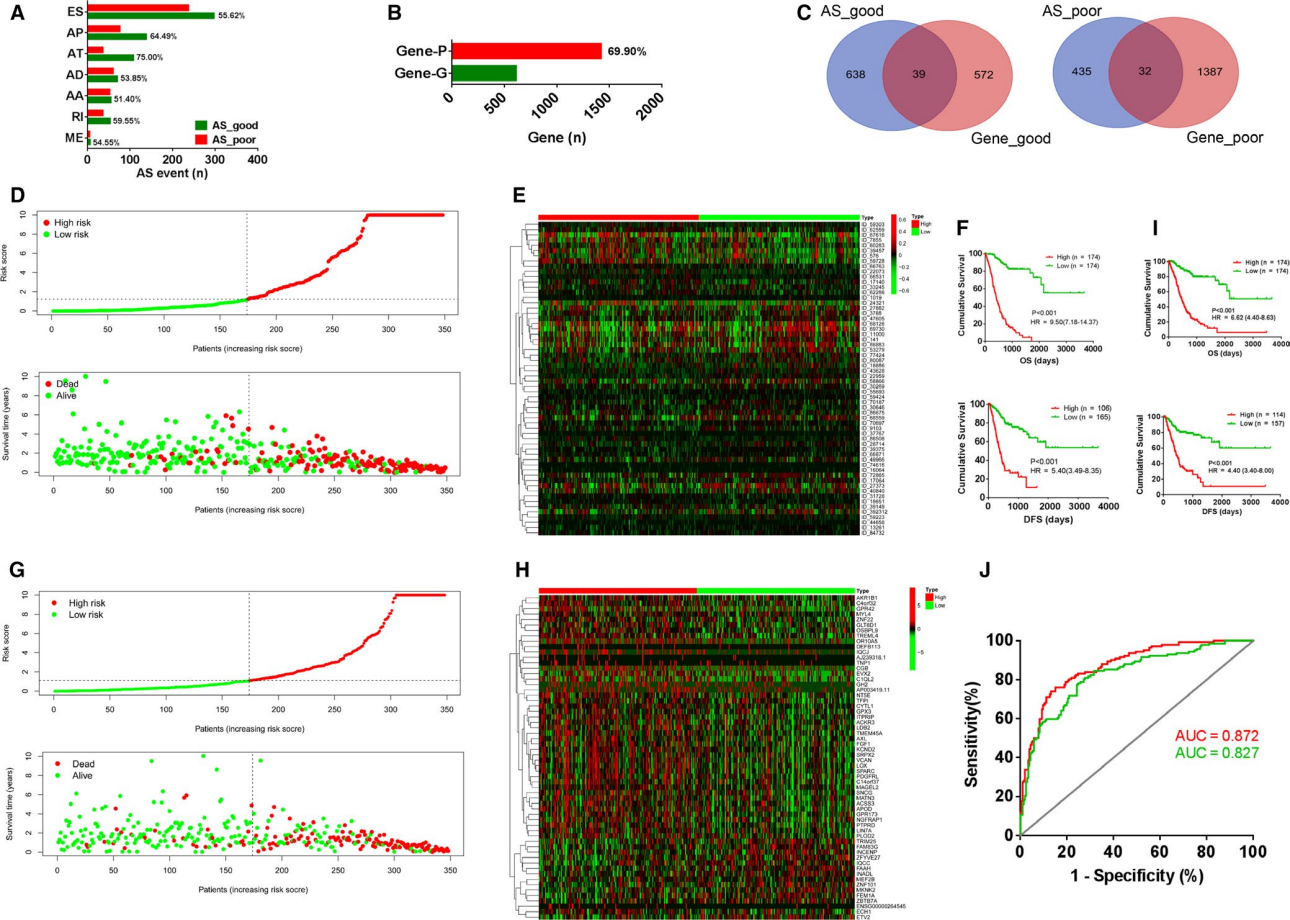
Prognosis‐related alternative splicing (AS) events and gene mRNA expression levels in gastric cancer (GC). A, Percentage of AS events of specific types significantly related to poor or good prognosis. AA, alternate acceptor sites; AD, alternate donor sites; AP, alternate promoter; AT, alternate terminator; ES, exon skipping; ME, mutually exclusive exons; RI, retained intron. B, Percentage of genes whose mRNA levels significantly related to poor (Gene‐P) or good prognosis (Gene‐G). C, Venn diagram showing the overlapping genes with both prognosis‐related mRNA levels and AS events. AS_poor, genes with AS events involved in poor prognosis; AS_good, genes with AS events involved in good prognosis; Gene_poor, genes whose mRNA levels associated with poor prognosis; Gene_good, genes whose mRNA levels associated with good prognosis. D, By constructing Cox regression model based on 60 AS events, all GC cases were divided into high‐risk (n = 174) and low‐risk (n = 174) groups, according to the median value of the risk scores (top). The distribution of survival time and survival status of the high‐risk and low‐risk groups (bottom). E, Heatmap showing expression of 60 AS events used to construct the model in the high‐risk and low‐risk groups. F, Kaplan‐Meier survival curve showing differences in OS and DFS between the low‐risk and high‐risk groups. G, By constructing Cox regression model based on 60‐gene expression data, all GC cases were divided into high‐risk (n = 174) and low‐risk (n = 174) groups, according to the median value of the risk scores (top). The distribution of survival time and survival status of the high‐risk and low‐risk groups (bottom). H, Heatmap showing expression of 60 genes used to construct the model in the high‐risk and low‐risk groups. I, Kaplan‐Meier survival curve showing differences in OS and DFS between the low‐risk and high‐risk groups. J, ROC curves were plotted to compare the predictive abilities of the Cox regression models constructed from the gene AS events (red line, AUC = 0.872) and the mRNA expression (green line, AUC = 0.827). AUC, area under the curve

We obtained a total of 31 911 AS events from the TCGASpliceSeq database, including 14 873 ES events, 3586 AA events, 167 ME events, 2952 AD events, 3626 AT events, 3826 AP events and 2881 RI events. Cox univariate regression analysis showed 1229 AS events significantly associated with prognosis, and 2030 genes associated with prognosis. Most AS events were significantly related with favourable prognosis, especially for the AP and AT types of AS (*P* < 0.05, Figure 1A; Table S2), but most gene expression‐related prognostic events were significantly associated with poor prognosis (*P* < 0.05, Figure 1B; Table S3). Venn diagrams were plotted to visualize the interactions between genes with prognostic AS events and genes with prognosis‐related mRNA expression (Figure 1C). There were only a few genes with both prognostic AS events and prognosis‐related mRNA levels in GC.

### 3.2 Prognostic prediction of Cox regression model based on AS events is superior to the model using gene expression

In order to compare the prognostic predictive ability of AS events and gene expression, prognostic AS events or prognosis‐related gene expression events were utilized to develop Cox regression model. Using prognostic AS events with *P* value <0.01, a Cox regression model based on 60 AS events were established (Table S4). Then, we used top 100 genes whose mRNA levels associated with prognosis to construct another Cox regression model. In order to make the two Cox model comparable, the Cox regression model was also developed based on 60‐gene expression data (Table S5). Risk scores based on these 60 AS events were significantly related to poor prognosis (high risk vs low risk: overall survival [OS], HR = 9.50 [7.18‐14.37], *P* < 0.001; disease‐free survival [DFS], HR = 5.40 [3.49‐8.35], *P* < 0.001, Figure 1D‐F). Risk scores based on expression of these 60 genes were also significantly associated with poor prognosis (high risk vs low risk: OS, HR = 4.70 [3.36‐6.59], *P* < 0.001; DFS, HR = 3.96 [3.04‐7.13], *P* < 0.001, Figure 1G‐I). The prognostic predictive ability of the Cox regression model based on AS events (red line, AUC = 0.872, Figure 1J) was more powerful than the model based on gene expression (green line: AUC = 0.827, Figure 1J).

### 3.3 GO and KEGG pathways analysis of prognostic AS events and genes

**FIGURE 2:**
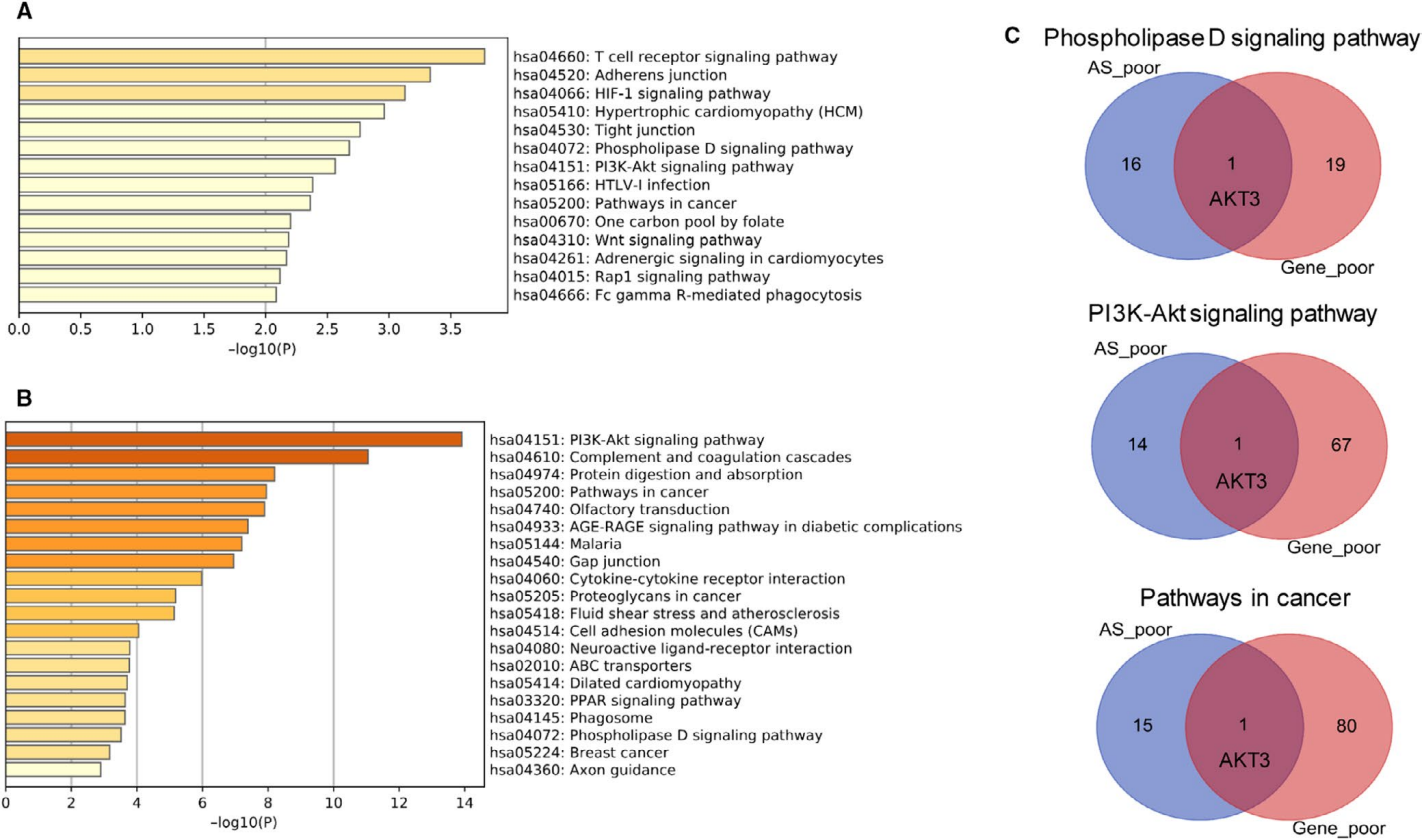
KEGG pathway analysis of genes whose expression and alternative splicing (AS) events associated with poor prognosis in gastric cancer. A, KEGG pathways analysis of genes with AS events associated with poor prognosis. B, KEGG pathways analysis of genes whose mRNA expression levels associated with poor prognosis. C, Genes with both poor prognostic AS events and poor prognosis‐related mRNA levels was significantly enriched in the Phospholipase D signalling pathway, the PI3K/AKT signalling pathway and pathways in cancer, but venn diagrams showed only one overlapping gene (AKT3)

In order to study the different functions of AS events and gene expression in GC progression, genes with prognostic AS events and mRNA expression were used to perform GO and KEGG pathways analysis (Figures S1 and S2; Figure 2A,B). Genes with poor prognostic AS events were most significantly enriched in the T‐cell receptor signalling pathway, and genes with poor prognostic mRNA expression levels were most significantly enriched in the PI3K/AKT signalling pathway (*P* < 0.001). Interestingly, we noticed that genes whose AS events (Figure 2A) or mRNA levels (Figure 2B) were associated with poor prognosis were simultaneously enriched in the PI3K/AKT signalling pathway, the phospholipase D signalling pathway and in pathways in cancer. However, AKT3 was the only overlapping gene among the three pathways (Figure 2C). These results suggested that AS events and gene expression promoted GC progression via the same mechanisms, but that the genes involved are distinct.

### 3.4 Screening and validation of hub genes with AS events significantly associated with tumour progression, stromal proportion and Lauren classification in GC

**FIGURE 3:**
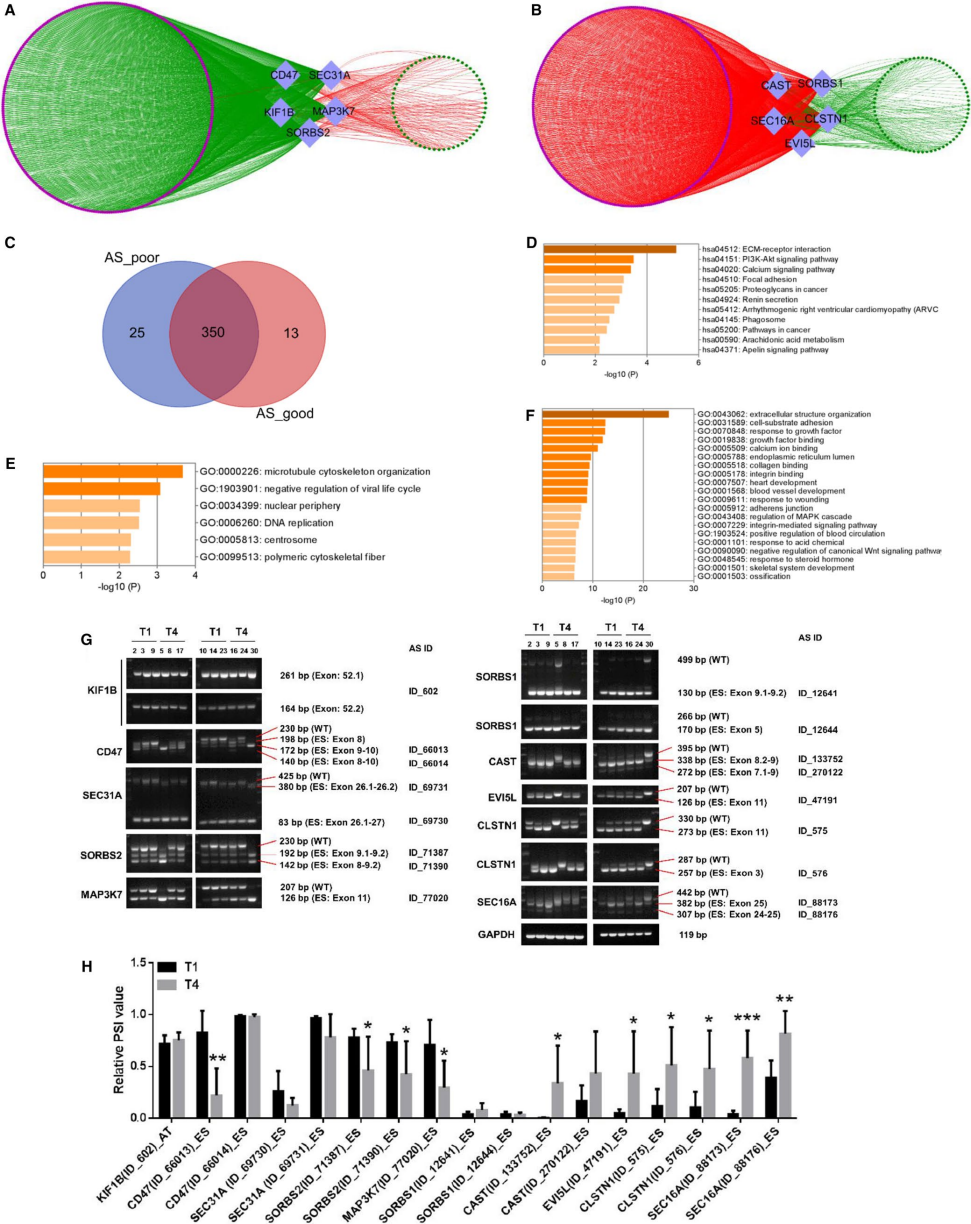
Identification and validation of hub survival‐associated alternative splicing (AS) events in gastric cancer (GC). A, Co‐expression network analysis between 363 genes with prognosis‐related mRNA levels and 5 hub genes with favourable prognostic AS events (R > 0.3, *P* < 0.001). B, Co‐expression network analysis between 375 genes with prognosis‐related mRNA levels and 5 hub genes with unfavourable prognostic AS events (R > 0.3, *P* < 0.001). The diamonds represent AS events. Purple nodes indicate genes with poor prognosis, and green nodes indicate genes with favourable prognosis. Red lines represent positive correlations, and green lines represent negative correlations. C, Between the above two networks (A and B), there were 350 overlapping genes whose RNA levels were significantly related to prognosis. AS_good: the genes whose mRNA levels were negatively related to the 8 favourable prognostic AS events of 5 hub genes (A); AS_poor: the genes whose RNA level were positively related to the 9 unfavourable prognostic AS events of 5 hub genes (B). D, KEGG pathway analysis of 297 of 350 overlapping genes whose mRNA levels were associated with poor prognosis. GO analysis of 297 poor prognostic genes of the overlapping 350 genes (E, biological process; F, cell component). G, Expression of 17 hub AS events of 10 genes in T1 (n = 6) and T4 (n = 6) GC tissues. H, Comparison of the 17 hub AS events expression between T1 and T4 GC tissues. *P < 0.05, **P < 0.01, ***P < 0.001

To screen for critical AS events involved in GC progression, we established co‐expression networks between genes with prognostic mRNA levels and genes with prognostic AS events. We identified 5 hub genes with 8 favourable prognostic AS events (Figure 3A) and 5 hub genes with 9 unfavourable prognostic AS events (Figure 3B; Table S6). We found that the hub prognostic AS events mainly correlated with expression of genes whose RNA levels were positively related to poor prognosis in GC (Figure 3B; Table S6). Between the two networks, there were 350 overlapping genes whose RNA levels were significantly related to prognosis (Figure 3C). RNA levels of 297 of 350 overlapping genes were positively related to poor prognosis. Then, GO and KEGG pathway analysis were performed with the 297 overlapping genes whose RNA levels were positively related to poor prognosis. The results showed that these genes were significantly enriched in the PI3K/AKT signalling pathway, phospholipase D signalling pathway, pathways in cancer, and in the GO term extracellular structure organization (Figure 3D‐F).

In order to explore whether these hub AS events are involved in GC progression, we further analysed the relationship between expression of AS events and clinicopathological features, such as T, N, M stage and Lauren classification. The favourable prognostic AS events were under‐expressed in invasive GC tissue, whereas poor prognostic AS events were over‐expressed in invasive GC tissue (Figures S3A,B). Additionally, we observed decreased AS events associated with good prognosis and increased AS events associated with poor prognosis in diffuse GC tissue (Figure S3C,D).

Because the hub AS events were significantly associated with extracellular matrix, we calculated stromal scores in the GC tissue by using the “estimate” package in R. Kaplan‐Meier survival analysis showed that higher stromal scores were significantly associated with poor OS (Figure S4A, HR = 1.59 [1.10‐2.17], *P* = 0.012) and DFS (Figure S4B, HR = 1.58 [1.03‐2.32], *P* = 0.037). We analysed correlations between the stromal score and 17 hub AS events in GC using Spearman correlation (Figure S4C,D). Favourable prognostic AS events were negatively correlated with stromal score (Figure S4C), whereas unfavourable prognostic AS events were positively correlated with stromal score (Figure S4D).

**FIGURE 4:**
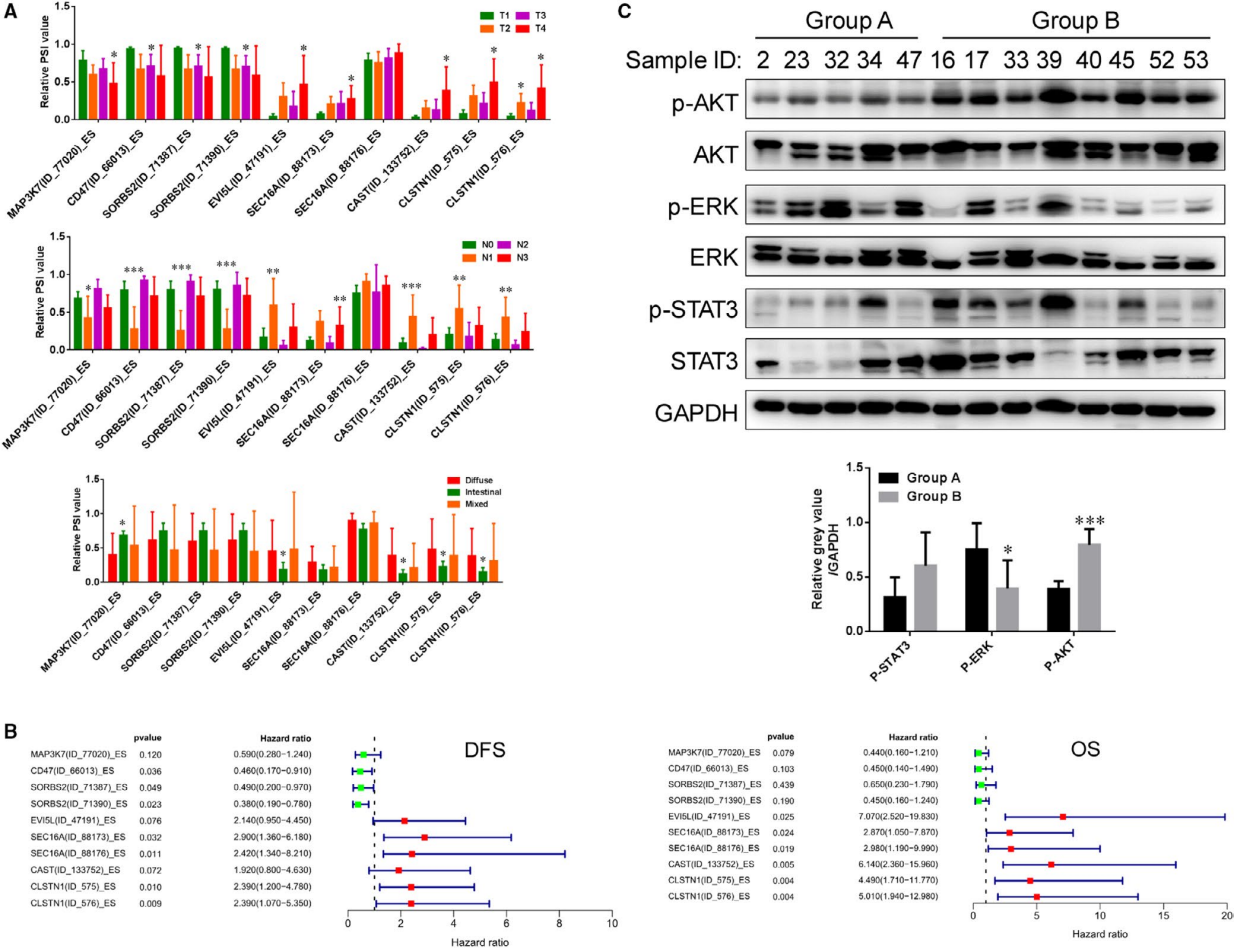
Validation of alternative splicing (AS) events involved in gastric cancer (GC) progression. A, Expression of 10 hub AS events of 7 genes in GC tissues with different T stages, N stages and Lauren classification. B, Forest plots showing the relationships between expression of AS events and patient prognosis in GC (DFS and OS). C, According to the expression of AS events in GC tissues, 5 GC tissues with high expression of AS events associated with good prognosis were named “Group A”, and 8 GC tissues with high expression of AS events associated poor prognosis were named “Group B”. Western blot was used to examine expression of p‐AKT, p‐ERK and p‐STAT3 in fresh GC tissues. The grey values of p‐AKT, p‐ERK and p‐STAT3 were calculated with respect to the internal control GAPDH and were compared between Group A and Group B. *P < 0.05, **P < 0.01, ***P < 0.001

In order to explore whether AS events were significantly associated with GC invasion, we initially compared expression of the AS events in T1 cancers (n = 6) and T4 cancers (n = 6). RT‐PCR was performed to verify AS expression in GC tissue (Figure 3G). Among the 17 hub AS events, we confirmed that 10 were differentially expressed in T4 GC tissue (Figure 3H). AS events in CD47, SORBS1 and MAP3K7 were decreased in T4 cancers, and AS events in CAST, EVI5L, CLSTN1 and SEC16A were increased in T4 cancers. We further examined expression of these AS events in 41 GC tissues (Figure S5). AS events in CD47, SORBS2 and MAP3K7 were down‐regulated in invasive, diffuse and lymph node metastatic GC tissue, and AS events in CAST, EVI5L, CLSTN1 and SEC16A were up‐regulated in invasive, diffuse and lymph node metastatic GC tissue (Figure 4A). High expression of AS events in CD47 and SORBS2 was significantly associated with longer DFS, and high expression of AS events in CLSTN1 and SEC16A was significantly associated with shorter DFS (Figure 4B). High expression of AS events in CAST, EVI5L, CLSTN1 and SEC16A was positively associated with shorter overall survival time (Figure 4B).

Simultaneously, we also calculated the total mRNA levels of genes by normalizing to GAPDH, including brands of WT and AS. Then, we also analysed the relationships between their RNA levels and invasion, lymph node metastasis and Lauren classification. Unlike AS event, we only observed decreased SEC16A in T2 GC tissues and decreased CLSTN1 in lymph node metastatic GC tissues (Figure S6A). Other genes did not show positive or negative correlations with invasion, lymph node metastasis and Lauren classification (Figure S6A). Besides, as for the above 7 genes with prognostic AS events, we only found CAST mRNA level was significantly related poor prognosis, and SEC16A mRNA level was significantly associated with good prognosis (Figure S6B) in TCGA database. In our validation cohort (n = 41), MAP3K7 mRNA level was significantly shorter DFS, and high expression of CLSTN1 and CAST was positively related to longer DFS (Figure S6C). SORBS2 mRNA level was significantly poor OS, and high expression of CD47 was positively related to better OS (Figure S6D). Our results indicated that the genes whose RNA levels were associated with clinical parameters and prognosis were different from the genes whose AS events were involved in clinical parameters and prognosis. Therefore, gene expression and gene AS event should be simultaneously concerned when we explore the function of certain gene.

Differential expression of AS events was found to significantly associate with activation of the PI3K/AKT pathway. By referencing the above results, we selected 5 GC tissues with high expression of AS events associated with good prognosis (ES type of MAP3K7, CD47 and SORBS2) and low expression of AS events associated with poor prognosis (ES type of EVI5L, SEC16A, CAST and CLSTN1), which were classified as Group A. Correspondingly, we also identified 8 GC tissues with low expression of AS events associated with good prognosis and high expression of AS events associated with poor prognosis, which were defined as Group B. Then, we performed Western blot to examine expression of p‐AKT, p‐ERK and p‐STAT3 in these tissues. We found that phosphorylated AKT was up‐regulated in Group B, but p‐ERK and p‐STAT3 were not (Figure 4C). Our results suggested that some hub AS events played critical roles in GC progression, especially AS events (ES) in CLSTN1 and SEC16A, and that these AS events might promote GC progression via activation of the PI3K/AKT pathway.

### 3.5 Identification of prognostic splicing factors and spliceosome genes correlated with AS events in GC

**FIGURE 5:**
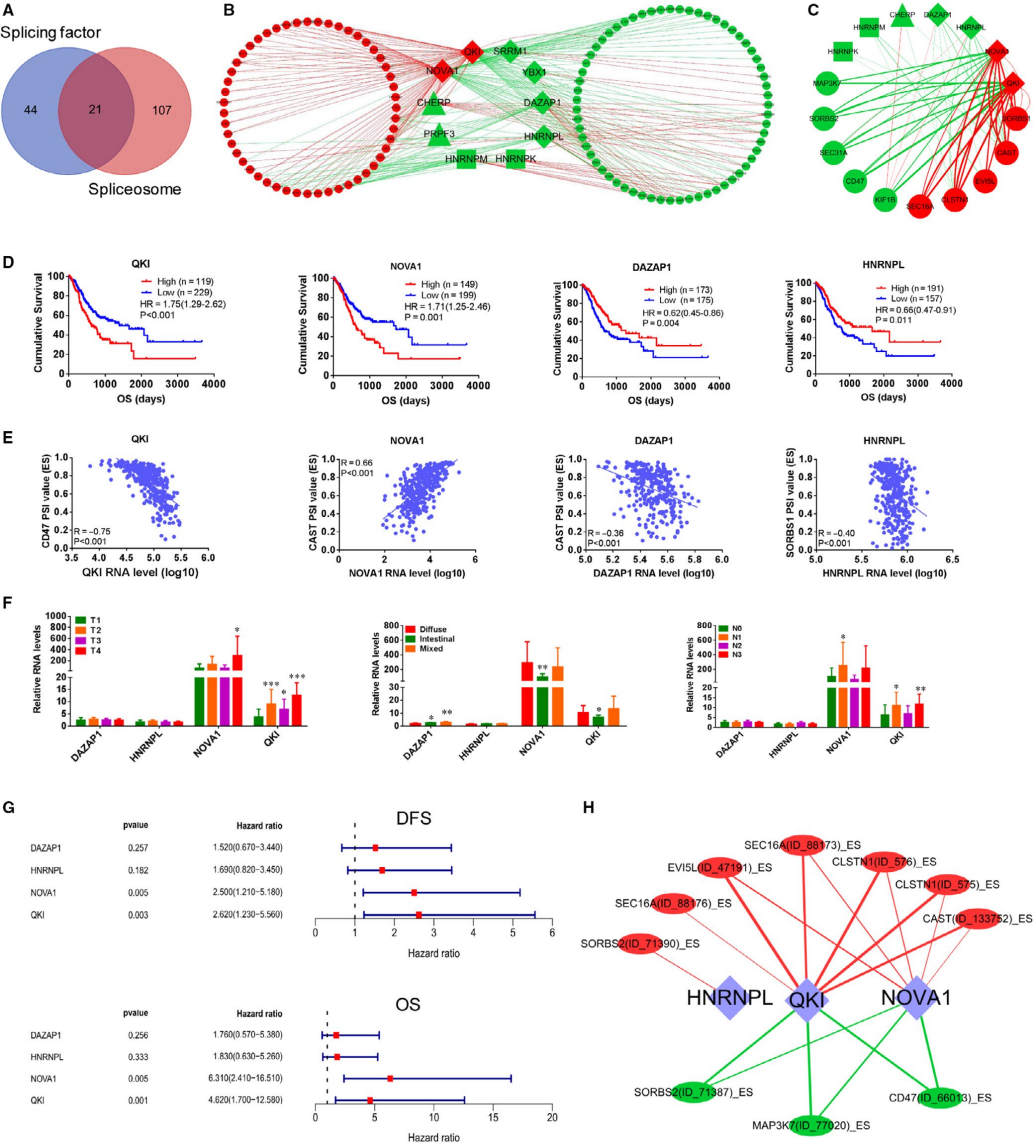
Co‐expression network analysis of survival‐associated splicing factors and spliceosome genes and alternative splicing (AS) events in gastric cancer (GC). A, Venn diagram showed the overlapping 21 gene playing dual roles of splicing factor and spliceosome. B and C, Co‐expression network analysis of prognostic splicing factor/spliceosome genes and all genes with prognostic AS events (B, R > 0.3, *P* < 0.001) Co‐expression network analysis of prognostic splicing factor/spliceosome genes and 10 hub genes with 17 prognostic AS events (C, R > 0.3, *P* < 0.001). Red circles: genes with poor prognostic AS events; Green circles: genes with favourable prognostic AS events; Red diamonds: splicing factor genes associated with poor prognosis; Green diamonds: splicing factor genes associated with good prognosis; Green triangles: spliceosome genes associated with good prognosis; Green rectangles: splicing factor/spliceosome genes associated with good prognosis; Red lines: positive correlations; green lines: negative correlations. The thicker the line, the stronger the correlations. D, NOVA1 and QKI, were significantly associated with poor prognosis, and DAZAP1 and HNRNPL were significantly related to good prognosis by K‐M analysis. E, QKI was negatively related to CD47 AS event (ES, R = −0.75, *P* < 0.001); NOVA1 was positively correlated to CAST AS event (ES, R = 0.66, *P* < 0.001); DAZAP1 was negatively associated with CAST AS event (ES, R = −0.36, *P* < 0.001); HNRNPL was negatively correlated to SORBS1 AS event (ES, R = −0.40, *P* < 0.001). F, Expression of DAZAP1, HNRNPL, NOVA1 and QKI mRNA levels in 53 GC tissues with different T stages, N stages and Lauren classification. G, Forest plots showing the relationships between DAZAP1, HNRNPL, NOVA1 and QKI mRNA levels and patient prognosis (DFS and OS). H, Co‐expression network of prognostic genes and AS events (R > 0.3, *P* < 0.05) were constructed in 53 GC tissues. The diamonds represent splicing factors. Red circles indicate genes associated with poor prognosis; green circles indicate genes associated with favourable prognosis. Red lines represent positive correlations; green lines represent negative correlations. The thicker the line, the stronger the correlation. *P < 0.05, **P < 0.01, ***P < 0.001

Splicing factors and spliceosome genes participate in regulation of AS events.^24^, ^25^ We downloaded 65 splicing factor genes and 128 spliceosome genes from the SpliceAid 2 database (http://193.206.120.249/splicing_tissue.html) and the KEGG database (https://www.kegg.jp/kegg/), respectively. Splicing factor and spliceosome genes showed 21 overlapping genes (Figure 5A). In order to analyse the critical splicing factors and spliceosome genes that regulate AS events, we performed Cox univariate regression analysis. Among splicing factors and spliceosome genes, HNRNPM, HNRNPK, DAZAP1, HNRNPL, YBX1, SRRM1, CHERP and PRPF3 were significantly associated with favourable prognosis, and NOVA1 and QKI were significantly associated with poor prognosis (Table S7). Of these, NOVA1, QKI, HNRNPM, HNRNPK, DAZAP1, HNRNPL, YBX1 and SRRM1 are splicing factor genes, and CHERP, PRPF3, HNRNPM and HNRNPK are spliceosome genes. We constructed a co‐expression network of prognostic splicing factor or/and spliceosome genes and AS events using the Spearman correlation method (R > 0.3, *P* < 0.001), and we found that expression of these 10 prognostic splicing factor or/and spliceosome genes was correlated with 293 prognostic AS events in 114 genes (Figure 5B). Of these AS events, 144 events in 52 genes were associated with poor prognosis (red nodes) and 149 AS events in 63 genes were associated with favourable prognosis (green nodes). Interestingly, splicing factor genes with poor prognosis showed positive correlations (red lines) with poor prognostic AS events and negative correlations (green lines) with favourable prognostic AS events. Splicing factor or/and spliceosome genes with good prognosis showed positive correlations (red lines) with good prognostic AS events and negative correlations (green lines) with poor prognostic AS events (Figure 5B). We further performed co‐expression network analysis between splicing factor or/and spliceosome genes and the 17 hub AS events we identified. The two poor prognostic splicing factor genes, NOVA1 and QKI, showed the strongest correlations with AS events (Figure 5C). In addition, other splicing factor or/and spliceosome genes showed fewer and weaker correlations with AS events, including the splicing factor genes DAZAP1, HNRNPL, the splicing factor or/and spliceosome genes HNRNPM and HNRNPK, and the spliceosome gene CHERP. Kaplan‐Meier analysis showed that the splicing factors NOVA1 and QKI were significantly associated with poor prognosis, and DAZAP1 and HNRNPL were significantly associated with good prognosis (Figure 5D). QKI was negatively associated with the CD47 AS event (ES, R = −0.75, *P* < 0.001, Figure 5E); NOVA1 was positively correlated to the CAST AS event (ES, R = 0.66, *P* < 0.001, Figure 5E); DAZAP1 was negatively associated with the CAST AS event (ES, R = −0.36, *P* < 0.001, Figure 5E); HNRNPL was negatively correlated to the SORBS1 AS event (ES, R = −0.40, *P* < 0.001, Figure 5E). We observed increased mRNA levels of NOVA1 and QKI in invasive, lymph node metastatic, and diffuse GC tissues (Figure 5F). Kaplan‐Meier survival analysis also indicated that high mRNA levels of NOVA1 and QKI were positively related to poor prognosis (Figure 5G). We performed correlation analysis among the 10 AS events and splicing factors, and selected paired gene AS events and gene expressions with Spearman coefficient >0.3 and *P* value <0.05 to construct a co‐expression network. We found that NOVA1 and QKI showed positive correlations with AS events involved in poor prognosis and negative correlations with AS events associated with good prognosis (Figure 5H). Our results indicated that splicing factor genes, but not spliceosome genes, widely participated in regulating AS events in GC.

## 4 DISCUSSION

Alternative mRNA splicing is a major source of protein diversity, and abnormal AS events are closely associated with tumour initiation and tumour progression.^26^, ^27^ Lin et al identified a prognostic AS signature but did not further performed analysis by integrating gene expression and AS events.^14^ Lin et al performed systematic analysis of survival‐associated AS signatures in GC,^14^ but did not include integrated analysis of gene expression. Previous studies have shown that taking genes expression and AS events into account can provide additional insight into mechanisms of cancer progression.^28^, ^29^ We therefore undertook simultaneous evaluation of AS events and mRNA expression to further uncover hub AS events involved in GC progression.

In this study, we evaluated 348 GC cases with gene expression, AS events and clinical data. By integrating gene expression and AS events, we found that most of the prognostic AS events were associated with good outcomes, and most of the prognostic mRNA expression was associated with poor outcome. There were few genes whose RNA levels and AS events were both significantly related to prognosis. Cox regression modelling demonstrated that AS events were more significantly associated with prognosis than gene expression. KEGG pathway analysis indicated that genes with poor prognosis AS events or genes with prognosis‐related mRNA levels were involved in activation of phospholipase D signalling pathway, PI3K‐Akt signalling pathway and pathways in cancer. Co‐expression analysis among gene expression and AS events revealed hub splicing factors and spliceosome genes and 17 hub AS events. Finally, in clinical GC samples, we confirmed that 10 of 17 hub events and 2 splicing factors were significantly associated with GC invasion, Lauren classification and metastasis. We found that these AS events might accelerate GC development via activating the PI3K/AKT pathway.

Abnormal splicing variants are involved in cancer development, including in GC.^5^, ^25^ For example, Meng et al reported significant participation of surviving, including survivin‐2B and survivin‐DeltaEx3 splice variants, in GC development.^30^ Other reports have indicated that AS of mRNAs played a vital role in GC progression.^31^ In the present study, we identified 1229 prognostic AS events related to GC outcome. To study the varied functions of AS events and gene expression, genes related with good or poor survival were analysed, respectively. The genes associated with poor prognosis were most significantly enriched in the T‐cell receptor signalling pathway. AS event of these genes may impair T‐cell signalling and disrupt T‐cell activation, which results in tumour immunosuppression.^32^ KEGG pathway analysis of genes with poor prognosis AS events or prognostic gene expression revealed enrichment in the PI3K‐AKT signalling pathway, the phospholipase D signalling pathway, and pathways in cancer. These data suggested that these AS events may enhance activation of the PI3K‐AKT signalling pathway (https://www.kegg.jp/kegg/kegg2.html). These three pathways were also positively related to GC progression, invasion and metastasis.^33^, ^34^ Li et al also reported that the longer isoform of MRPL33 (MRPL33‐L) promotes GC epirubicin resistance by activating the PI3K/AKT pathway.^30^ In breast cancer, CD44 alternative splicing causally contributes to EMT cancer progression by activating PI3K/AKT pathway.^35^

Gene expression can regulate AS events, and AS events can also regulate gene expression.^18^, ^19^, ^20^ In order to identify critical AS events involved in GC progression, we performed co‐expression network analysis among the prognostic AS events and prognosis‐related genes with correlation coefficient >0.3 and *P* value <0.001. Ultimately, 17 hub AS events were identified, and we confirmed the expression of these events in GC tissue. Consistent with the above KEGG pathway analysis results, the 17 hub AS events were involved in activation of PI3K/AKT signalling pathway and pathways in cancer. Furthermore, we found that GC with high expression of AS events associated with poor prognosis and low expression of AS events associated with good prognosis exhibited activation of the PI3K/AKT pathway. In addition, these 17 hub genes were also significantly related to extracellular structure organization and ECM‐receptor interaction, which are also significantly related to activation of the PI3K/AKT pathway, the integrin‐mediated signalling pathway and the MAPK pathway.^36^, ^37^, ^38^ In GC, extracellular structure organization and ECM‐receptor interactions promote cancer invasion.^39^, ^40^ In this study, we found that the 17 hub AS events were significantly related to tumour invasion in GC in the TCGA database. Of import, our data indicated a tumour suppressive role for AS events in CD47 (ES) and KIF1B (AT). KIF1B functions as a tumour suppressor in neuroblastoma, but promotes GC invasion.^41^, ^42^ CD47 expression in human melanoma regulates NK cell function.^43^ CAST promotes GC cell responses to 5‐fluorouracil by regulating the thymidylate synthase‐5‐fluoro‐dUMP complex,^44^ but promotes radiation resistance in Glioblastoma multiforme cells by enhancing activity of calpain proteases.^45^ SORBS1, a member of the PPAR pathway, can suppress tumour metastasis and promote chemotherapy sensitivity in cancer,^46^, ^47^ but has also been reported to correlate with prostate cancer recurrence.^48^ AS events in genes may explain the diverse roles of those genes in cancer, including GC. AS events of KIF1B and CLSTN1 have been reported to be involved in ovarian cancer and lung cancer development.^49^, ^50^ Many of the hub AS events, including AS events in KIFB1, SEC31A, CAST and CLSTN1, were up‐regulated in invasive and diffuse GC tissues and were significantly related to extracellular matrix and tumour stromal score. Intratumour stromal proportion is closely associated with aggressive phenotypes in gastric signet ring cell carcinomas.^51^ We found that the stromal score of GC tumours was also significantly associated with poor prognosis. In GC, gene variants in genes such as CD44, E‐cadherin and RHOA are significantly related to invasive and diffuse cancer invasion.^52^, ^53^, ^54^ Finally, we confirmed that 10 of the 17 hub AS events of within 10 genes were significantly related to invasion, lymph node metastasis and Lauren classification in GC, especially AS events in CLSTN1 and SEC16A. Therefore, in this study, we not only presented novel potential markers to distinguish and diagnose early GCs, but also revealed some possible therapeutic targets for GC. Though these two genes have not been thoroughly studied, some previous reports indicate that they serve crucial roles in cancer progression.^50^, ^55^ Our results revealed that these hub AS events promoted GC progression via the PI3K/AKT signalling pathway and pathways in cancer.

Splice isoforms could exhibit strikingly opposite functions in cancers, such as CD44.^56^ CD44 standard splice isoform promote cancer stem cell traits, but the CD44 variant splice isoforms exhibit an inverse role.^56^ Similarly, our results indicated that a gene‐mediated GC progression may not depend on increased mRNA levels, but the spliced isoforms. Therefore, studies focused on gene AS event may help to more clearly classify the underling mechanisms involved in GC progression.

Both splicing factors and spliceosome genes can regulate tissue‐ and cancer‐specific alternative splicing events,^24^, ^25^ and dysregulation of splicing factors and spliceosome genes has been observed in cancer tissues.^5^, ^25^, ^57^ By regulating AS, splicing factors, such as PTBP1 and MBNL3, can function as oncogenes or pseudo‐oncogenes and promote cancer progression.^58^, ^59^ Liang et al reported that PTBP3, an essential RNA‐binding protein with roles in RNA splicing, promotes GC metastasis by mediating AS of CAV1.^60^ SNRPE and SNRPD1, core components of the spliceosome, can promote viability of lung cancer, breast cancer and melanoma.^61^ By constructing co‐expression network among prognostic splicing factors and AS events, we identified hub splicing factor genes, including QKI, NOVA1, HNRNPL and DAZAP1. In 53 GC clinical specimens, we found that QKI and NOVA1 were closely related to poor prognosis. Abnormal expression of QKI and NOVA1 has been reported to participate in the development of lung cancer and colorectal cancer.^62^, ^63^ Among the 128 spliceosome genes, CHERP, HNRNPK and HNRNPM were shown to correlate with limited AS events. Splicing factor genes showed stronger correlations with AS events than spliceosome genes. Our results indicated that splicing factor genes likely played critical roles in gene AS events in GC, especially QKI and NOVA1, but spliceosome genes do not. However, the functions of these splicing factors in GC need to be further elucidated.

In conclusion, by integrated analysis of AS events and gene expression, we identified hub AS events in CLSTN1 and SEC16A, and splicing factors QKI and NOVA1, which were significantly associated with GC progression. We validated expression of these AS events in GC tissues. AS events may accelerate GC progression via the PI3K/AKT pathway, and these AS events may be potential therapeutic targets in GC. Studies focused on gene AS event and gene expression help to revealed more key genes in GC progression.

## CONFLICT OF INTEREST

The authors declare that there is no conflict of interests.

## AUTHOR CONTRIBUTION

**Haoran Feng:** Data curation (equal); methodology (equal); resources (equal); software (equal); validation (equal); visualization (equal); writing – original draft (equal). **Zhijian Jin:** Data curation (equal); formal analysis (equal); validation (equal); visualization (equal). **Kun Liu:** Methodology (equal); validation (equal); visualization (equal). **Yi Peng:** Investigation (equal); software (equal). **Songyao Jiang:** Investigation (equal); methodology (equal). **Changgang Wang:** Data curation (equal); writing – original draft (equal). **Jiele Hu:** Investigation (equal); software (equal). **Xiaoyun Shen:** Validation (equal). **Weihua Qiu:** Formal analysis (equal); project administration (equal). **Xi Cheng:** Project administration (equal); supervision (equal); writing – review and editing (equal). **Ren Zhao:** Funding acquisition (equal); project administration (equal); supervision (equal); writing – review and editing (equal).

## Supporting information

Fig S1

Fig S2

Fig S3

Fig S4

Fig S5

Fig S6

Table S1

Table S2

Table S3

Table S4

Table S5

Table S6

Table S7

## Data Availability

The data used to support the findings of this study are included within the article.

## References

[1] Bray F , Ferlay J , Soerjomataram I , et al. Global cancer statistics 2018: GLOBOCAN estimates of incidence and mortality worldwide for 36 cancers in 185 countries. CA Cancer J Clin. 2018;68:394‐424. 30207593 10.3322/caac.21492

[2] Kang M‐H , Choi H , Oshima M , et al. Estrogen‐related receptor gamma functions as a tumor suppressor in gastric cancer. Nat Commun. 2018;9:1920. 29765046 10.1038/s41467-018-04244-2 PMC5954140

[3] Liang YU , Zhang C‐D , Zhang C , et al. DLX6‐AS1/miR‐204‐5p/OCT1 positive feedback loop promotes tumor progression and epithelial‐mesenchymal transition in gastric cancer. Gastric Cancer. 2020;23(2):212‐227. 31463827 10.1007/s10120-019-01002-1

[4] Zhang H , Deng T , Liu R , et al. Exosome‐delivered EGFR regulates liver microenvironment to promote gastric cancer liver metastasis. Nat Commun. 2017;8:15016. 28393839 10.1038/ncomms15016 PMC5394240

[5] Zhang Y , Yan L , Zeng J , et al. Pan‐cancer analysis of clinical relevance of alternative splicing events in 31 human cancers. Oncogene. 2019;38(40):6678‐6695. 31391553 10.1038/s41388-019-0910-7

[6] Climente‐González H , Porta‐Pardo E , Godzik A , et al. The functional impact of alternative splicing in cancer. Cell Rep. 2017;20:2215‐2226. 28854369 10.1016/j.celrep.2017.08.012

[7] Nilsen TW , Graveley BR . Expansion of the eukaryotic proteome by alternative splicing. Nature. 2010;463:457‐463. 20110989 10.1038/nature08909 PMC3443858

[8] Padgett RA . New connections between splicing and human disease. Trends Genet. 2012;28:147‐154. 22397991 10.1016/j.tig.2012.01.001 PMC3319163

[9] Li KE , Shen B , Cheng XI , et al. Phenotypic and signaling consequences of a novel aberrantly spliced transcript FGF receptor‐3 in hepatocellular carcinoma. Can Res. 2016;76:4205‐4215. 10.1158/0008-5472.CAN-15-3385 27267910

[10] Griffith M , Griffith OL , Mwenifumbo J , et al. Alternative expression analysis by RNA sequencing. Nat Methods. 2010;7:843‐847. 20835245 10.1038/nmeth.1503

[11] Li Y , Sun N , Lu Z , et al. Prognostic alternative mRNA splicing signature in non‐small cell lung cancer. Cancer Lett. 2017;393:40‐51. 28223168 10.1016/j.canlet.2017.02.016

[12] Zhu J , Chen Z , Yong L . Systematic profiling of alternative splicing signature reveals prognostic predictor for ovarian cancer. Gynecol Oncol. 2018;148:368‐374. 29191436 10.1016/j.ygyno.2017.11.028

[13] He R‐Q , Zhou X‐G , Yi Q‐Y , et al. Prognostic signature of alternative splicing events in bladder urothelial carcinoma based on spliceseq data from 317 cases. Cell Physiol Biochem. 2018;48:1355‐1368. 30048970 10.1159/000492094

[14] Lin P , He R‐Q , Ma F‐C , et al. Systematic analysis of survival‐associated alternative splicing signatures in gastrointestinal pan‐adenocarcinomas. EBioMedicine. 2018;34:46‐60. 30131306 10.1016/j.ebiom.2018.07.040 PMC6116578

[15] Bisognin A , Pizzini S , Perilli L , et al. An integrative framework identifies alternative splicing events in colorectal cancer development. Mol Oncol. 2014;8:129‐141. 24189147 10.1016/j.molonc.2013.10.004 PMC5528503

[16] Li Z , Zhao K , Tian H . Integrated analysis of differential expression and alternative splicing of non‐small cell lung cancer based on RNA sequencing. Oncol Lett. 2017;14:1519‐1525. 28789374 10.3892/ol.2017.6300 PMC5529932

[17] Kalari KR , Rossell D , Necela BM , et al. Deep sequence analysis of non‐small cell lung cancer: integrated analysis of gene expression, alternative splicing, and single nucleotide variations in lung adenocarcinomas with and without oncogenic KRAS mutations. Front Oncol. 2012;2:12. 22655260 10.3389/fonc.2012.00012 PMC3356053

[18] Pimentel H , Parra M , Gee S , et al. A dynamic alternative splicing program regulates gene expression during terminal erythropoiesis. Nucleic Acids Res. 2014;42:4031‐4042. 24442673 10.1093/nar/gkt1388 PMC3973340

[19] Gabut M , Samavarchi‐Tehrani P , Wang X , et al. An alternative splicing switch regulates embryonic stem cell pluripotency and reprogramming. Cell. 2011;147:132‐146. 21924763 10.1016/j.cell.2011.08.023

[20] Hu J , Khodadadi‐Jamayran A , Mao M , et al. AKAP95 regulates splicing through scaffolding RNAs and RNA processing factors. Nat Commun. 2016;7:13347. 27824034 10.1038/ncomms13347 PMC5105168

[21] Sano T , Coit DG , Kim HH , et al. Proposal of a new stage grouping of gastric cancer for TNM classification: International Gastric Cancer Association staging project. Gastric Cancer. 2017;20:217‐225. 26897166 10.1007/s10120-016-0601-9 PMC4992472

[22] Wu M‐F , Chuang C‐Y , Lin P , et al. Lung tumorigenesis alters the expression of Slit2‐exon15 splicing variants in tumor microenvironment. Cancers. 2019;11(2):166. 10.3390/cancers11020166 PMC6406468 30717252

[23] Feng H , Cheng XI , Kuang J , et al. Apatinib‐induced protective autophagy and apoptosis through the AKT‐mTOR pathway in anaplastic thyroid cancer. Cell Death Dis. 2018;9:1030. 30301881 10.1038/s41419-018-1054-3 PMC6177436

[24] Dvinge H , Guenthoer J , Porter PL , et al. RNA components of the spliceosome regulate tissue‐ and cancer‐specific alternative splicing. Genome Res. 2019;29(10):1591‐1604. 31434678 10.1101/gr.246678.118 PMC6771400

[25] Sveen A , Kilpinen S , Ruusulehto A , et al. Aberrant RNA splicing in cancer; expression changes and driver mutations of splicing factor genes. Oncogene. 2016;35:2413‐2427. 26300000 10.1038/onc.2015.318

[26] Paronetto MP , Passacantilli I , Sette C . Alternative splicing and cell survival: from tissue homeostasis to disease. Cell Death Differ. 2016;23:1919‐1929. 27689872 10.1038/cdd.2016.91 PMC5136496

[27] Narayanan SP , Singh S , Shukla S . A saga of cancer epigenetics: linking epigenetics to alternative splicing. Biochem J. 2017;474:885‐896. 28270561 10.1042/BCJ20161047

[28] Song J , Liu YD , Su J , et al. Systematic analysis of alternative splicing signature unveils prognostic predictor for kidney renal clear cell carcinoma. J Cell Physiol. 2019;234:22753‐22764. 31140607 10.1002/jcp.28840 PMC6771988

[29] Koch L . Alternative splicing: a thermometer controlling gene expression. Nat Rev Genet. 2017;18:515. 28736438 10.1038/nrg.2017.61

[30] Meng H , Lu C , Mabuchi H , et al. Prognostic significance and different properties of survivin splicing variants in gastric cancer. Cancer Lett. 2004;216:147‐155. 15533590 10.1016/j.canlet.2003.12.020

[31] Li Y , Yuan Y . Alternative RNA splicing and gastric cancer. Mutat Res. 2017;773:263‐273. 28927534 10.1016/j.mrrev.2016.07.011

[32] Meininger I , Griesbach RA , Hu D , et al. Alternative splicing of MALT1 controls signalling and activation of CD4(+) T cells. Nat Commun. 2016;7:11292. 27068814 10.1038/ncomms11292 PMC4832065

[33] Liu L , Ye Y , Zhu X . MMP‐9 secreted by tumor associated macrophages promoted gastric cancer metastasis through a PI3K/AKT/Snail pathway. Biomed Pharmacother. 2019;117:109096. 31202170 10.1016/j.biopha.2019.109096

[34] Kang DW , Hwang WC , Park MH , et al. Rebamipide abolishes *Helicobacter pylori* CagA‐induced phospholipase D1 expression via inhibition of NFkappaB and suppresses invasion of gastric cancer cells. Oncogene. 2013;32:3531‐3542. 22890316 10.1038/onc.2012.358

[35] Brown RL , Reinke LM , Damerow MS , et al. CD44 splice isoform switching in human and mouse epithelium is essential for epithelial‐mesenchymal transition and breast cancer progression. J Clin Invest. 2011;121:1064‐1074. 21393860 10.1172/JCI44540 PMC3049398

[36] Chaudhuri O , Koshy ST , Branco da Cunha C , et al. Extracellular matrix stiffness and composition jointly regulate the induction of malignant phenotypes in mammary epithelium. Nat Mater. 2014;13:970‐978. 24930031 10.1038/nmat4009

[37] Levental KR , Yu H , Kass L , et al. Matrix crosslinking forces tumor progression by enhancing integrin signaling. Cell. 2009;139:891‐906. 19931152 10.1016/j.cell.2009.10.027 PMC2788004

[38] Trappmann B , Gautrot JE , Connelly JT , et al. Extracellular‐matrix tethering regulates stem‐cell fate. Nat Mater. 2012;11:642‐649. 22635042 10.1038/nmat3339

[39] Huang J , Sun YI , Chen H , et al. ADAMTS5 acts as a tumor suppressor by inhibiting migration, invasion and angiogenesis in human gastric cancer. Gastric Cancer. 2019;22:287‐301. 30105548 10.1007/s10120-018-0866-2

[40] Hu C , Ni Z , Li B‐S , et al. hTERT promotes the invasion of gastric cancer cells by enhancing FOXO3a ubiquitination and subsequent ITGB1 upregulation. Gut. 2017;66:31‐42. 26370108 10.1136/gutjnl-2015-309322

[41] Dong Z , Xu X , Du L , et al. Leptin‐mediated regulation of MT1‐MMP localization is KIF1B dependent and enhances gastric cancer cell invasion. Carcinogenesis. 2013;34:974‐983. 23354307 10.1093/carcin/bgt028

[42] Chen ZX , Wallis K , Fell SM , et al. RNA helicase A is a downstream mediator of KIF1Bbeta tumor‐suppressor function in neuroblastoma. Cancer Discov. 2014;4:434‐451. 24469107 10.1158/2159-8290.CD-13-0362

[43] Nath PR , Pal‐Nath D , Mandal A , et al. Natural killer cell recruitment and activation are regulated by CD47 expression in the tumor microenvironment. Cancer Immunol Res. 2019;7:1547‐1561. 31362997 10.1158/2326-6066.CIR-18-0367 PMC6726576

[44] Nabeya Y , Suzuki T , Furuya A , et al. Calpain regulates thymidylate synthase‐5‐fluoro‐dUMP complex levels associated with response to 5‐fluorouracil in gastric cancer cells. Cancer Sci. 2011;102:1509‐1515. 21561529 10.1111/j.1349-7006.2011.01978.x PMC11158892

[45] Bassett EA , Palanichamy K , Pearson M , et al. Calpastatin phosphorylation regulates radiation‐induced calpain activity in glioblastoma. Oncotarget. 2018;9:14597‐14607. 29581866 10.18632/oncotarget.24523 PMC5865692

[46] Chen YZ , Xue JY , Chen CM , et al. PPAR signaling pathway may be an important predictor of breast cancer response to neoadjuvant chemotherapy. Cancer Chemother Pharmacol. 2012;70:637‐644. 22903535 10.1007/s00280-012-1949-0

[47] Song L , Chang R , Dai C , et al. SORBS1 suppresses tumor metastasis and improves the sensitivity of cancer to chemotherapy drug. Oncotarget. 2017;8:9108‐9122. 27791200 10.18632/oncotarget.12851 PMC5354718

[48] Aakula A , Kohonen P , Leivonen S‐K , et al. Systematic identification of microRNAs that impact on proliferation of prostate cancer cells and display changed expression in tumor tissue. Eur Urol. 2016;69:1120‐1128. 26489476 10.1016/j.eururo.2015.09.019

[49] Gordon MA , Babbs B , Cochrane DR , et al. The long non‐coding RNA MALAT1 promotes ovarian cancer progression by regulating RBFOX2‐mediated alternative splicing. Mol Carcinog. 2019;58:196‐205. 30294913 10.1002/mc.22919

[50] Langer W , Sohler F , Leder G , et al. Exon array analysis using re‐defined probe sets results in reliable identification of alternatively spliced genes in non‐small cell lung cancer. BMC Genomics. 2010;11:676. 21118496 10.1186/1471-2164-11-676 PMC3053589

[51] Lee D , Ham I‐H , Son SY , et al. Intratumor stromal proportion predicts aggressive phenotype of gastric signet ring cell carcinomas. Gastric Cancer. 2017;20:591‐601. 27858181 10.1007/s10120-016-0669-2

[52] da Cunha CB , Oliveira C , Wen X , et al. De novo expression of CD44 variants in sporadic and hereditary gastric cancer. Lab Invest. 2010;90:1604‐1614. 20856229 10.1038/labinvest.2010.155

[53] Becker KF , Atkinson MJ , Reich U , et al. E‐cadherin gene mutations provide clues to diffuse type gastric carcinomas. Cancer Res. 1994;54:3845‐3852. 8033105

[54] Miyamoto S , Nagamura Y , Nakabo A , et al. Aberrant alternative splicing of RHOA is associated with loss of its expression and activity in diffuse‐type gastric carcinoma cells. Biochem Biophys Res Commun. 2018;495:1942‐1947. 29247652 10.1016/j.bbrc.2017.12.067

[55] Thutkawkorapin J , Picelli S , Kontham V , et al. Exome sequencing in one family with gastric‐ and rectal cancer. BMC Genet. 2016;17:41. 26872740 10.1186/s12863-016-0351-z PMC4752738

[56] Zhang H , Brown RL , Wei Y , et al. CD44 splice isoform switching determines breast cancer stem cell state. Genes Dev. 2019;33:166‐179. 30692202 10.1101/gad.319889.118 PMC6362815

[57] Takayama K‐I , Suzuki T , Fujimura T , et al. Dysregulation of spliceosome gene expression in advanced prostate cancer by RNA‐binding protein PSF. Proc Natl Acad Sci USA. 2017;114:10461‐10466. 28893982 10.1073/pnas.1706076114 PMC5625911

[58] Yuan J‐H , Liu X‐N , Wang T‐T , et al. The MBNL3 splicing factor promotes hepatocellular carcinoma by increasing PXN expression through the alternative splicing of lncRNA‐PXN‐AS1. Nat Cell Biol. 2017;19:820‐832. 28553938 10.1038/ncb3538

[59] Bielli P , Panzeri V , Lattanzio R , et al. The splicing factor PTBP1 promotes expression of oncogenic splice variants and predicts poor prognosis in patients with non‐muscle‐invasive bladder cancer. Clin Cancer Res. 2018;24:5422‐5432. 30012566 10.1158/1078-0432.CCR-17-3850

[60] Liang X , Chen W , Shi H , et al. PTBP3 contributes to the metastasis of gastric cancer by mediating CAV1 alternative splicing. Cell Death Dis. 2018;9:569. 29752441 10.1038/s41419-018-0608-8 PMC5948206

[61] Quidville V , Alsafadi S , Goubar A , et al. Targeting the deregulated spliceosome core machinery in cancer cells triggers mTOR blockade and autophagy. Cancer Res. 2013;73:2247‐2258. 23358685 10.1158/0008-5472.CAN-12-2501

[62] Ludlow AT , Wong MS , Robin JD , et al. NOVA1 regulates hTERT splicing and cell growth in non‐small cell lung cancer. Nat Commun. 2018;9:3112. 30082712 10.1038/s41467-018-05582-x PMC6079032

[63] Mukohyama J , Isobe T , Hu Q , et al. miR‐221 targets QKI to enhance the tumorigenic capacity of human colorectal cancer stem cells. Cancer Res. 2019;79(20):5151‐5158. 31416845 10.1158/0008-5472.CAN-18-3544 PMC6801097

