# Supplementary figures and images for: Identification and validation of critical alternative splicing events and splicing factors in gastric cancer progression
Source: J Cell Mol Med. 2020 Sep 16;24(21):12667–80. doi: 10.1111/jcmm.15835 (PMC7686978; doi:10.1111/jcmm.15835)

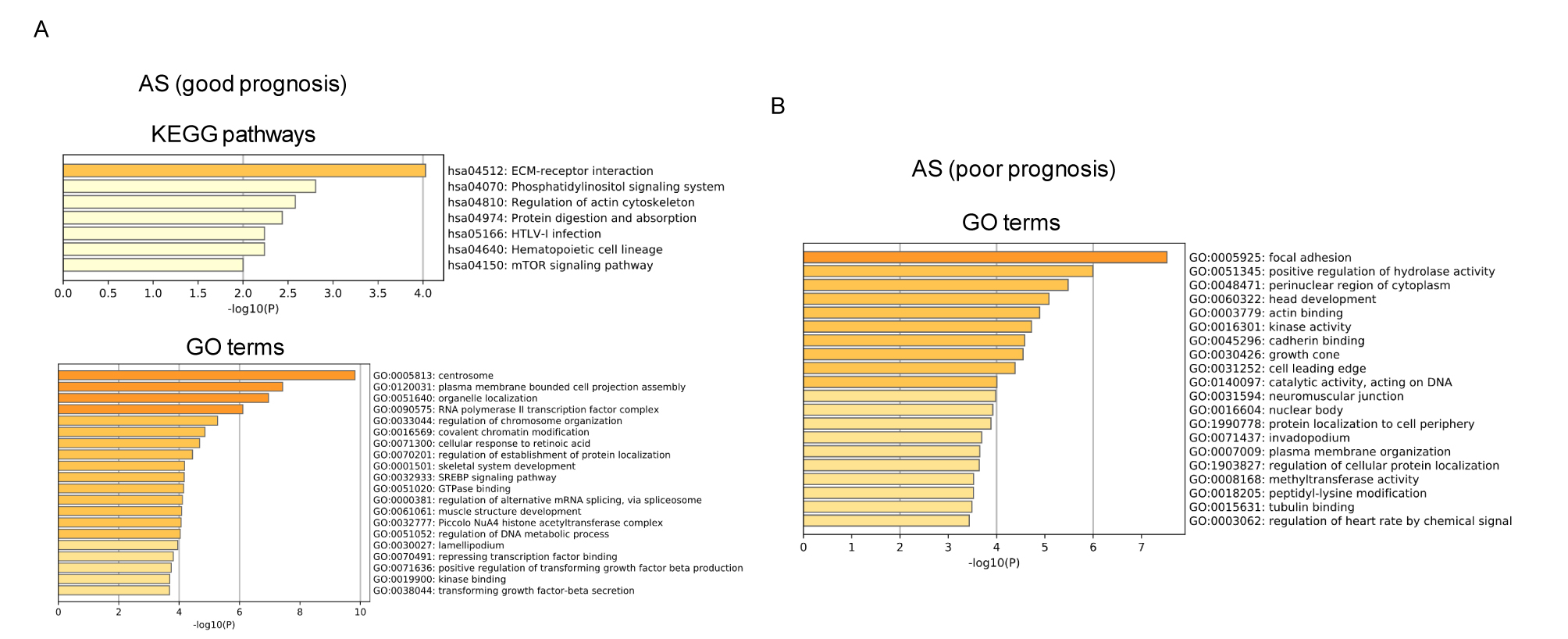

Supplement: Supplementary file 1 — Fig S1 [file JCMM-24-12667-s001.jpg]

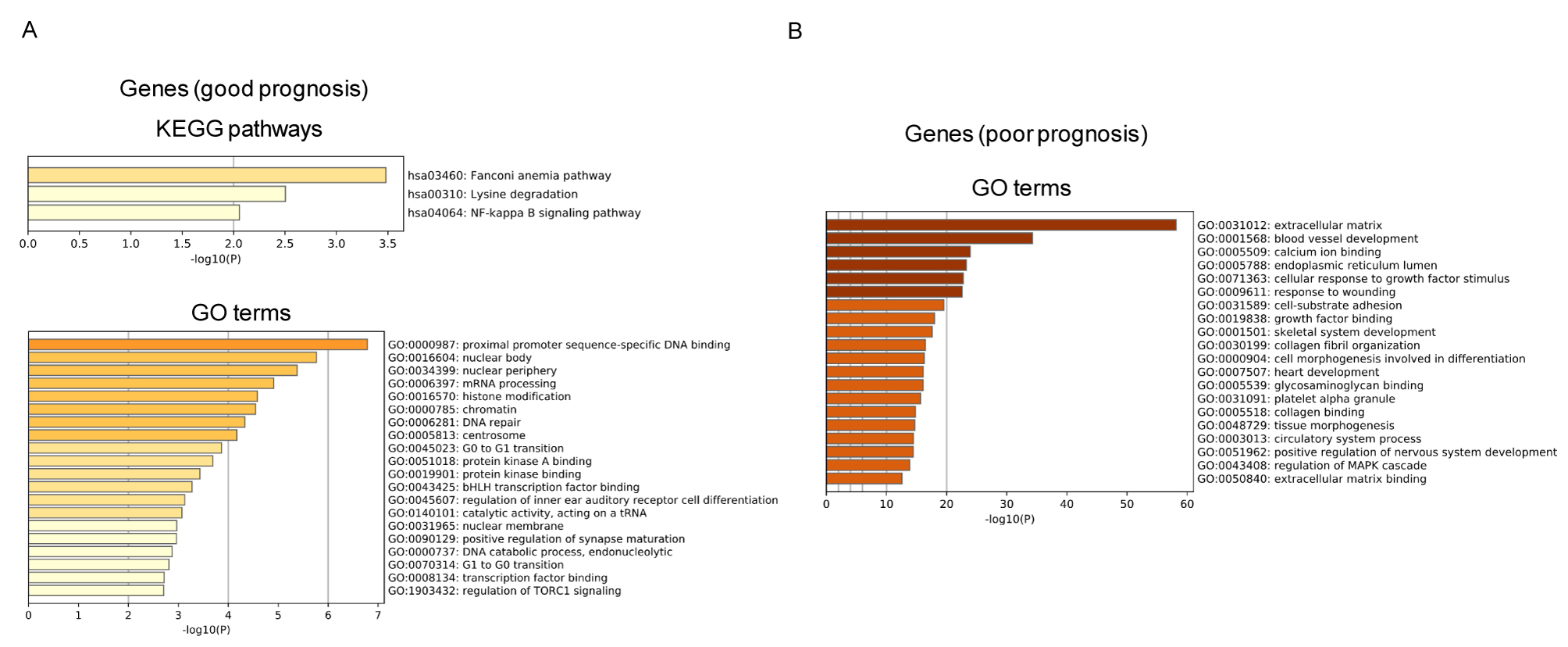

Supplement: Supplementary file 2 — Fig S2 [file JCMM-24-12667-s002.jpg]

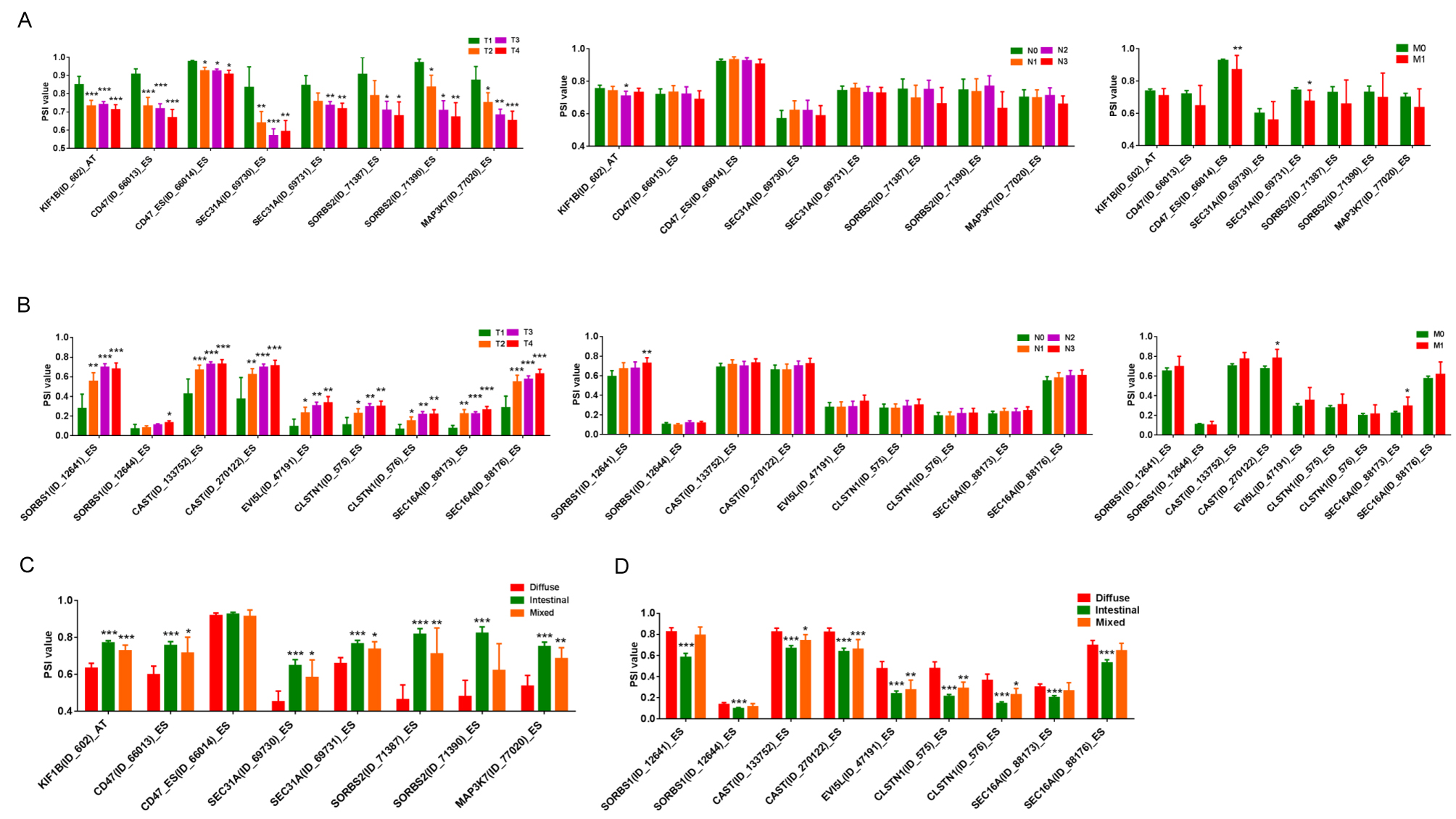

Supplement: Supplementary file 3 — Fig S3 [file JCMM-24-12667-s003.jpg]

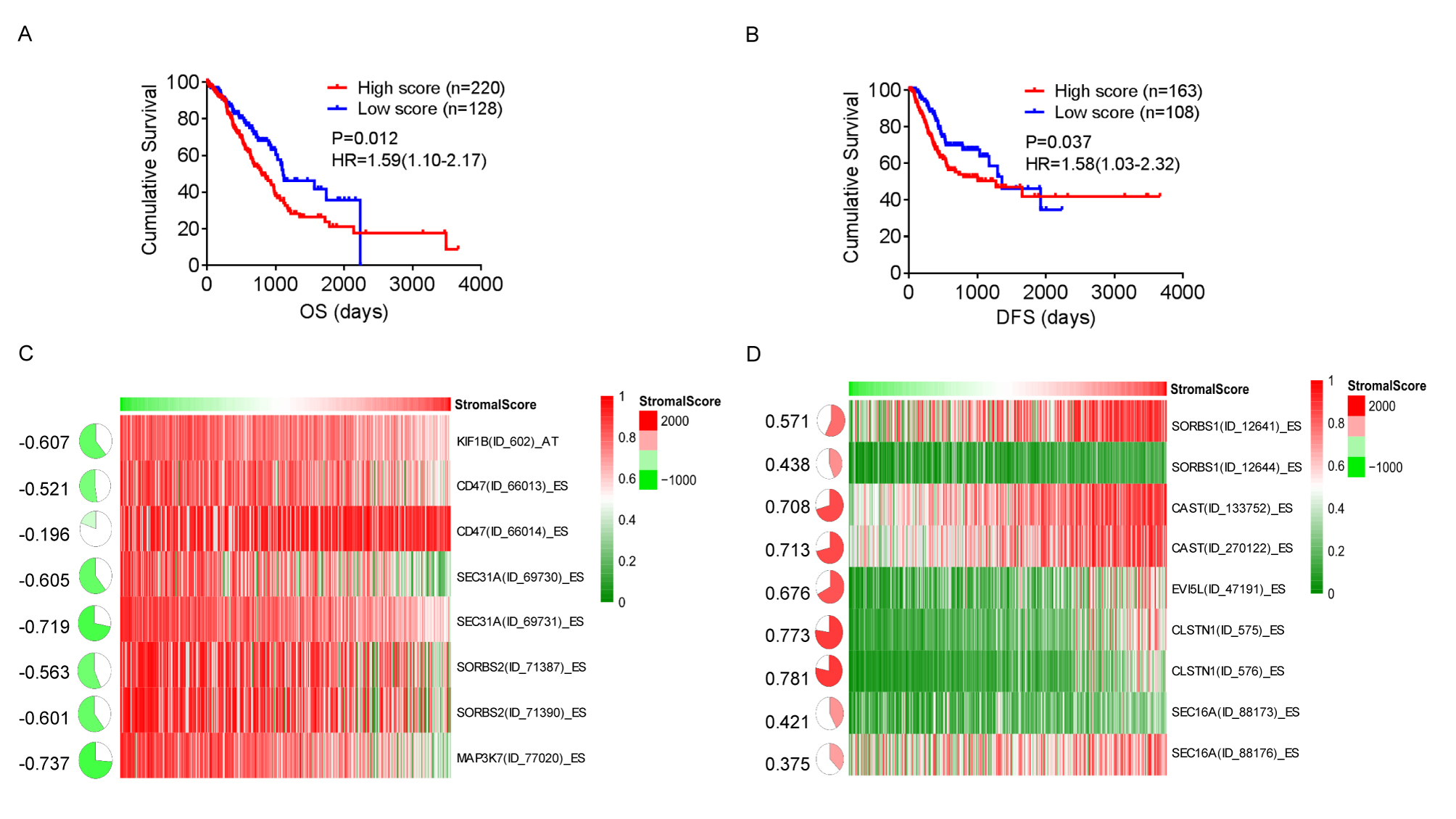

Supplement: Supplementary file 4 — Fig S4 [file JCMM-24-12667-s004.jpg]

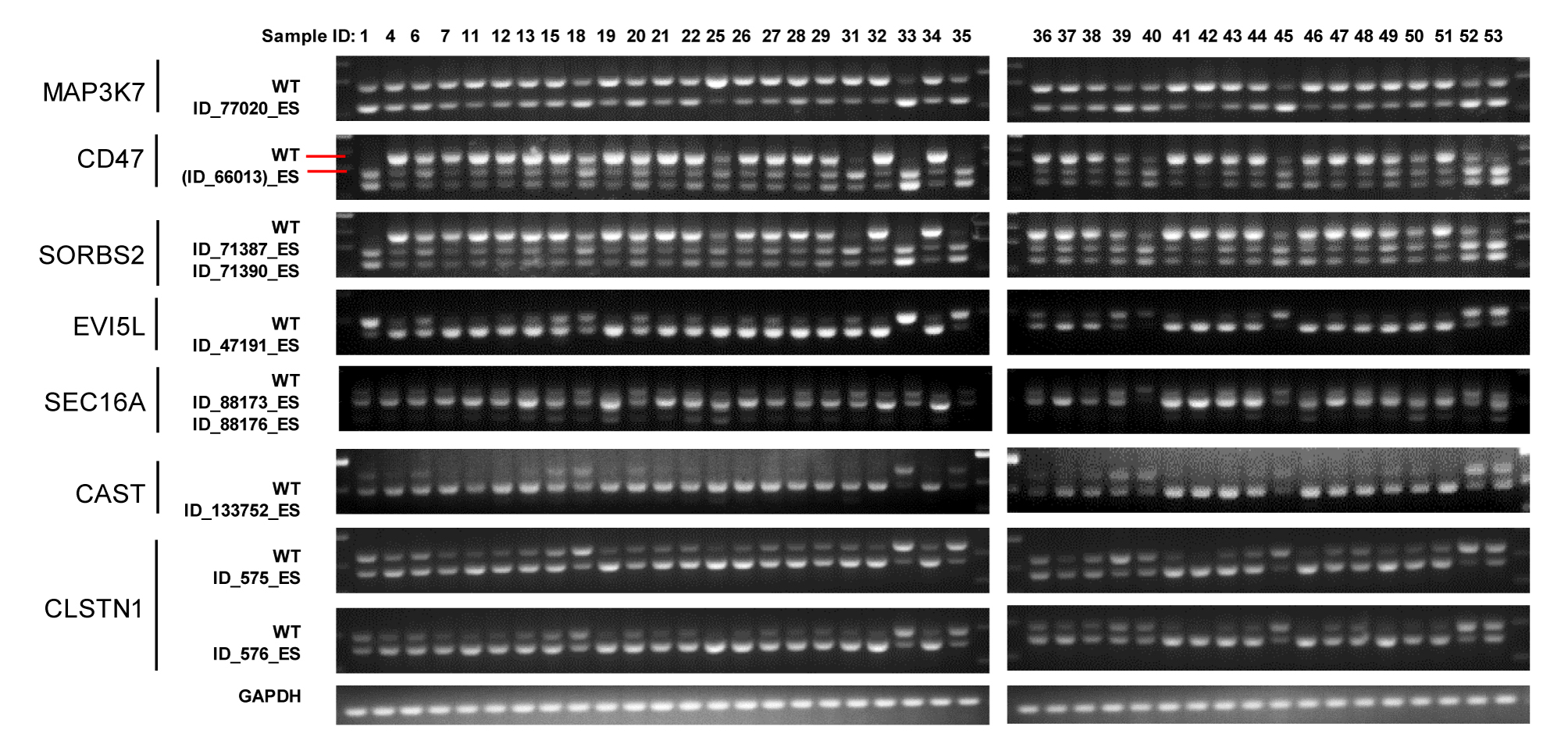

Supplement: Supplementary file 5 — Fig S5 [file JCMM-24-12667-s005.jpg]

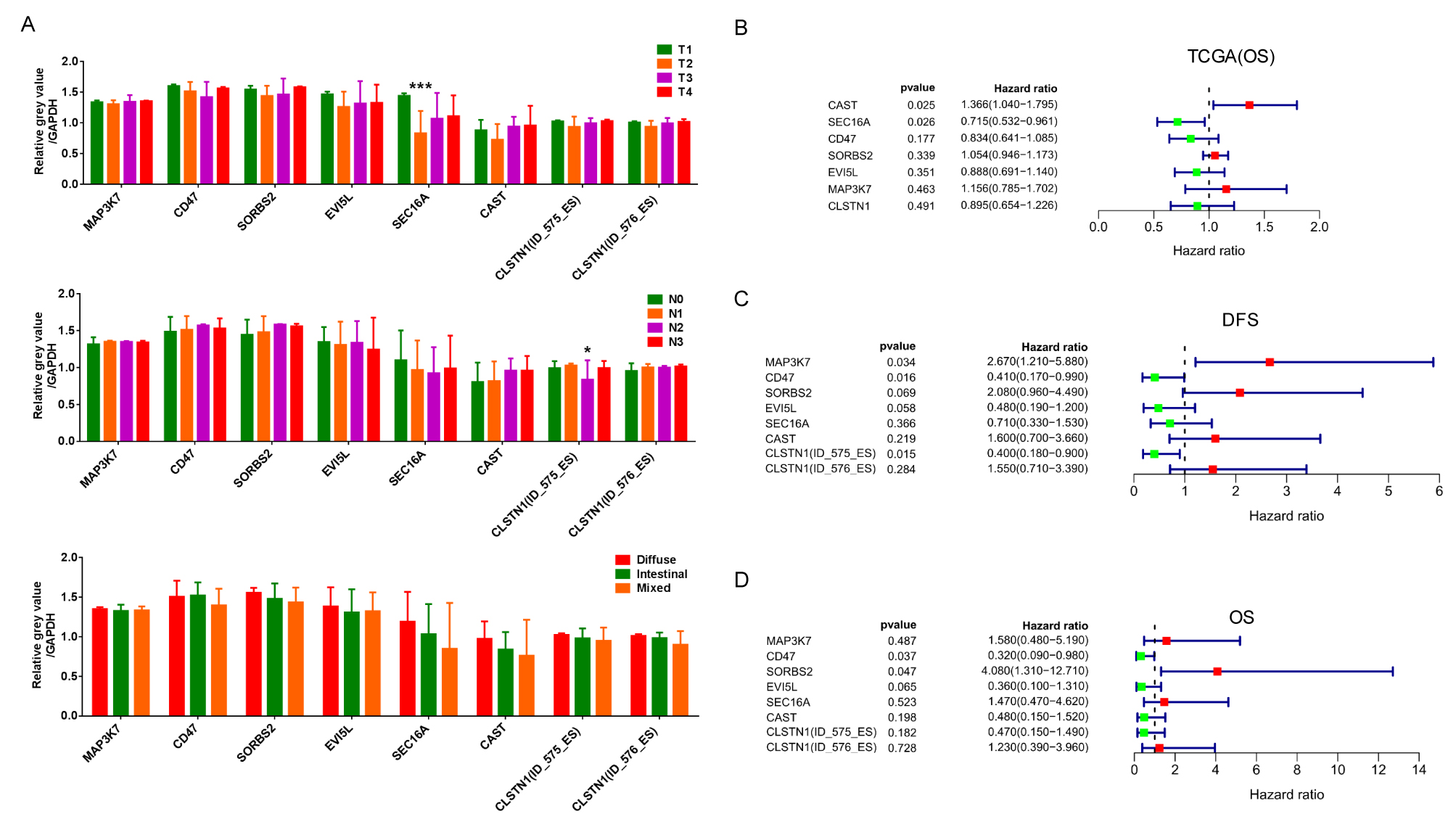

Supplement: Supplementary file 6 — Fig S6 [file JCMM-24-12667-s006.jpg]
