# Supplementary material for: Identification and validation of critical alternative splicing events and splicing factors in gastric cancer progression
Source: J Cell Mol Med. 2020 Sep 16;24(21):12667–80. doi: 10.1111/jcmm.15835 (PMC7686978; doi:10.1111/jcmm.15835)
Supplement: Supplementary file 7 — Table S1 [file JCMM-24-12667-s007.docx]

Table S1. Primers used in this study.

| Gene symbol | AS ID | Splice type | exons | Forward | Reverse |
| --- | --- | --- | --- | --- | --- |
| MAP3K7 | ID_77020 | ES | 11 | CTGAGGGCAAGAGGATGAGT | GACCAGGTTCTGTTCCAGTTAC |
| KIF1B | ID_602 | AT | 52.2 | AGTGTCTCAGTTCTGCTTTTGG | CGGGGTTTCATCATATTGG |
| KIF1B |  |  | 52.1 | TCATCCTCCTTTCTCCCATC | TTGCCTGTGCTCCTGACTAG |
| CD47 | ID_66014 | ES | 8:09:10 | TCTGTATTGCGGCGTGTATA | TCCAAATCGGAGTCCATCA |
| CD47 | ID_66013 | ES | 9:10 |  |  |
| SORBS2 | ID_71387 | ES | 9.1:9.2 | GCCACTGACTCCTACTTCCTCT | GGAATATGTAGCATCTCCGTTC |
| SORBS2 | ID_71390 | ES | 8:9.1:9.2 |  |  |
| SEC31A | ID_69731 | ES | 26.1:26.2 | TCCCACATCTCCAGGTCATA | GAGGGTCATTCCAACCATTC |
| SEC31A | ID_69730 | ES | 26.1:26.2:27 |  |  |
| EVI5L | ID_47191 | ES | 12 | AGAAGATGAAGAGGCTGGAGA | CCCGCTTGATGACGTAGTT |
| SORBS1 | ID_12641 | ES | 9.1:9.2 | GCCAAGGGCTACAGAAGTGT | GCTTGCTAACTGATGGAAAGG |
| SORBS1 | ID_12644 | ES | 5 | ATGAATGGCTTGGCACCT | CCTGGTTTGCTTTCGTGTT |
| SEC16A | ID_88176 | ES | 1.017361 | GAAGAGCAGCAGGAACCAGA | GCAGGGTTGTAGAAGGGCAT |
| SEC16A | ID_88173 | ES | 25 |  |  |
| CAST | ID_133752 | ES | 8.2:9 | CAGTGAATCGCCTTCCAAA | TGTGAGCATCGTTACTTCCTGT |
| CAST | ID_270122 | ES | 7.1:8.2:9 |  |  |
| CLSTN1 | ID_575 | ES | 11 | TGAGGAATGGCACCACTACG | AGCCCCTCCTTGCAGGTATA |
| CLSTN1 | ID_576 | ES | 3 | TACCACGGCATAGTCACAGAG | GGTCCCTTCCCACAATCAT |
| QKI |  |  |  | GCACTTGTCCGTTCGTCTT | CCATCTGTCTCCCCTTCTTAA |
| NOVA1 |  |  |  | CATCTTCCCCAACTACCACC | GCTCCATTACAGCCTTCACA |
| DAZAP1 |  |  |  | CGCTAGATGGCCGAAACAT | TCCGTGACCACTCCGAACTT |
| HNRNPL |  |  |  | TACGCAGCCGACAACCAAA | CCATCGCCTGAACTCCATT |
