# Supplementary material for: Identification and validation of critical alternative splicing events and splicing factors in gastric cancer progression
Source: J Cell Mol Med. 2020 Sep 16;24(21):12667–80. doi: 10.1111/jcmm.15835 (PMC7686978; doi:10.1111/jcmm.15835)
Supplement: Supplementary file 8 — Table S2 [file JCMM-24-12667-s008.docx]

Table S2. AS events significantly associated with prognosis in GC in the TCGA database (P<0.05).

| AS_ID | Symbol | | Splice type | | P value | | HR | | | Low 95%CI | | High  95%CI |
| --- | --- | --- | --- | --- | --- | --- | --- | --- | --- | --- | --- | --- |
| ID_100334 | MLLT10 | | ES | | 0.009 | | 0.256 | | | 0.092 | | 0.709 |
| ID_100880 | SEC31A | | AD | | 0.012 | | 0.095 | | | 0.015 | | 0.602 |
| ID_1014 | C1QC | | AD | | 0.011 | | 0.120 | | | 0.024 | | 0.610 |
| ID_101547 | ARHGAP8 | | ME | | 0.003 | | 96.261 | | | 4.520 | | 2050.147 |
| ID_101805 | DGKD | | ES | | 0.029 | | 8.452 | | | 1.239 | | 57.648 |
| ID_1019 | C1QC | | AD | | 0.005 | | 0.000 | | | 0.000 | | 0.002 |
| ID_102349 | MCFD2 | | ME | | 0.026 | | 0.053 | | | 0.004 | | 0.706 |
| ID_102413 | MTHFSD | | ME | | 0.017 | | 37.945 | | | 1.894 | | 760.058 |
| ID_10272 | C1orf131 | | RI | | 0.017 | | 51.257 | | | 2.019 | | 1301.513 |
| ID_10323 | PCNXL2 | | AT | | 0.007 | | 0.028 | | | 0.002 | | 0.371 |
| ID_10367 | GPR137B | | AT | | 0.018 | | 0.001 | | | 0.000 | | 0.286 |
| ID_10371 | ERO1LB | | ES | | 0.040 | | 0.055 | | | 0.004 | | 0.874 |
| ID_10405 | MTR | | ES | | 0.049 | | 520.359 | | | 1.018 | | 266018.973 |
| ID_10454 | CEP170 | | AA | | 0.005 | | 0.026 | | | 0.002 | | 0.335 |
| ID_10463 | AKT3 | | AT | | 0.043 | | 124.631 | | | 1.176 | | 13210.981 |
| ID_10470 | DESI2 | | ES | | 0.027 | | 2883.973 | | | 2.436 | | 3414294.310 |
| ID_10504 | ZNF695 | | ES | | 0.002 | | 0.005 | | | 0.000 | | 0.155 |
| ID_1051 | ZNF436 | | AP | | 0.025 | | 0.461 | | | 0.235 | | 0.906 |
| ID_10513 | ZNF669 | | AD | | 0.009 | | 4.606 | | | 1.473 | | 14.403 |
| ID_10573 | PGBD2 | | AA | | 0.046 | | 4.946 | | | 1.028 | | 23.802 |
| ID_105800 | AKAP10 | | ES | | 0.018 | | 0.041 | | | 0.003 | | 0.581 |
| ID_107348 | GPR56 | | AA | | 0.018 | | 27.855 | | | 1.770 | | 438.332 |
| ID_10815 | FRMD4A | | ES | | 0.019 | | 0.104 | | | 0.016 | | 0.694 |
| ID_10963 | NEBL | | AT | | 0.026 | | 0.043 | | | 0.003 | | 0.687 |
| ID_10973 | MLLT10 | | ES | | 0.017 | | 0.245 | | | 0.077 | | 0.778 |
| ID_10974 | MLLT10 | | ES | | 0.037 | | 0.161 | | | 0.029 | | 0.895 |
| ID_11000 | KIAA1217 | | RI | | 0.007 | | 0.273 | | | 0.105 | | 0.706 |
| ID_11002 | KIAA1217 | | ES | | 0.018 | | 0.198 | | | 0.052 | | 0.755 |
| ID_11050 | ABI1 | | ES | | 0.003 | | 69.011 | | | 4.394 | | 1083.940 |
| ID_11107 | SVIL | | AP | | 0.017 | | 2.212 | | | 1.153 | | 4.243 |
| ID_11120 | MAP3K8 | | AP | | 0.033 | | 0.341 | | | 0.126 | | 0.918 |
| ID_11156 | EPC1 | | AP | | 0.001 | | 0.048 | | | 0.008 | | 0.303 |
| ID_11184 | ITGB1 | | AP | | 0.025 | | 40767.298 | | | 3.821 | | 434981710.609 |
| ID_11201 | NRP1 | | ES | | 0.037 | | 171.845 | | | 1.375 | | 21474.225 |
| ID_11245 | CREM | | ES | | 0.002 | | 0.137 | | | 0.040 | | 0.474 |
| ID_11264 | CREM | | ES | | 0.012 | | 4.923 | | | 1.429 | | 16.961 |
| ID_11338 | ZNF485 | | ES | | 0.027 | | 623.001 | | | 2.067 | | 187782.820 |
| ID_11433 | SYT15 | | AT | | 0.011 | | 4.697 | | | 1.425 | | 15.481 |
| ID_115012 | TPM1 | | ES | | 0.005 | | 4.275 | | | 1.568 | | 11.656 |
| ID_11536 | NCOA4 | | AP | | 0.003 | | 0.060 | | | 0.009 | | 0.396 |
| ID_116241 | ANXA11 | | ES | | 0.037 | | 0.272 | | | 0.080 | | 0.926 |
| ID_118420 | UFD1L | | ES | | 0.048 | | 0.229 | | | 0.053 | | 0.984 |
| ID_11852 | ANK3 | | ME | | 0.003 | | 2.486 | | | 1.356 | | 4.556 |
| ID_11865 | ARID5B | | AP | | 0.022 | | 3.643 | | | 1.205 | | 11.007 |
| ID_11960 | CCAR1 | | AD | | 0.043 | | 0.060 | | | 0.004 | | 0.914 |
| ID_119816 | RAB40C | | ES | | 0.033 | | 0.033 | | | 0.001 | | 0.764 |
| ID_120409 | HNRNPA1 | | ES | | 0.040 | | 0.003 | | | 0.000 | | 0.762 |
| ID_12079 | ASCC1 | | ES | | 0.034 | | 0.318 | | | 0.110 | | 0.917 |
| ID_12080 | ASCC1 | | ES | | 0.045 | | 0.049 | | | 0.003 | | 0.932 |
| ID_12125 | NUDT13 | | ES | | 0.049 | | 0.113 | | | 0.013 | | 0.995 |
| ID_12152 | PPP3CB | | AP | | 0.040 | | 0.000 | | | 0.000 | | 0.591 |
| ID_12155 | PPP3CB | | ES | | 0.011 | | 0.209 | | | 0.063 | | 0.694 |
| ID_12156 | PPP3CB | | AA | | 0.049 | | 0.127 | | | 0.016 | | 0.988 |
| ID_12249 | CAMK2G | | ES | | 0.011 | | 4.761 | | | 1.423 | | 15.927 |
| ID_12262 | KAT6B | | AP | | 0.003 | | 0.165 | | | 0.050 | | 0.545 |
| ID_12297 | RPS24 | | AA | | 0.014 | | 0.386 | | | 0.180 | | 0.827 |
| ID_1231 | CNKSR1 | | AP | | 0.042 | | 0.246 | | | 0.064 | | 0.949 |
| ID_12379 | TSPAN14 | | ES | | 0.038 | | 0.130 | | | 0.019 | | 0.895 |
| ID_12409 | CCSER2 | | ES | | 0.019 | | 2.538 | | | 1.164 | | 5.533 |
| ID_124369 | ZFPM1 | | ES | | 0.012 | | 0.009 | | | 0.000 | | 0.356 |
| ID_12474 | ACTA2 | | AP | | 0.032 | | 41.471 | | | 1.379 | | 1247.227 |
| ID_12481 | FAS | | ES | | 0.018 | | 0.066 | | | 0.007 | | 0.629 |
| ID_12487 | LIPA | | AD | | 0.037 | | 46.706 | | | 1.263 | | 1726.520 |
| ID_12499 | KIF20B | | AA | | 0.022 | | 8.645 | | | 1.369 | | 54.599 |
| ID_1250 | UBXN11 | | AT | | 0.012 | | 4.701 | | | 1.408 | | 15.699 |
| ID_12545 | EXOC6 | | ES | | 0.012 | | 35.393 | | | 2.189 | | 572.377 |
| ID_12552 | MYOF | | ES | | 0.005 | | 2.423 | | | 1.314 | | 4.468 |
| ID_12589 | | HELLS | | ES | | 0.048 | | 9939025051.20 | 1.240 | | 79678199620158200000 | |
| ID_1263 | UBXN11 | | ES | | 0.003 | | 0.109 | | | 0.026 | | 0.462 |
| ID_12641 | SORBS1 | | ES | | 0.001 | | 3.050 | | | 1.595 | | 5.834 |
| ID_12644 | SORBS1 | | ES | | 0.008 | | 16.360 | | | 2.067 | | 129.496 |
| ID_12645 | ALDH18A1 | | AD | | 0.007 | | 2.895 | | | 1.345 | | 6.232 |
| ID_12654 | ENTPD1 | | ES | | 0.042 | | 2030.210 | | | 1.340 | | 3076930.120 |
| ID_12859 | C10orf2 | | AD | | 0.042 | | 5.129 | | | 1.058 | | 24.858 |
| ID_130134 | NR5A2 | | ME | | 0.004 | | 0.331 | | | 0.157 | | 0.700 |
| ID_13029 | OBFC1 | | AP | | 0.030 | | 0.186 | | | 0.040 | | 0.851 |
| ID_13032 | COL17A1 | | AT | | 0.000 | | 0.004 | | | 0.000 | | 0.063 |
| ID_13040 | WDR96 | | AT | | 0.047 | | 0.503 | | | 0.255 | | 0.991 |
| ID_13077 | ADD3 | | ES | | 0.024 | | 0.295 | | | 0.102 | | 0.853 |
| ID_13156 | TCF7L2 | | ES | | 0.023 | | 0.243 | | | 0.072 | | 0.819 |
| ID_13160 | HABP2 | | AT | | 0.032 | | 0.000 | | | 0.000 | | 0.502 |
| ID_1323 | MAP3K6 | | ES | | 0.004 | | 5.048 | | | 1.695 | | 15.028 |
| ID_13261 | RGS10 | | AP | | 0.001 | | 0.001 | | | 0.000 | | 0.067 |
| ID_13270 | INPP5F | | AP | | 0.012 | | 4.210 | | | 1.380 | | 12.847 |
| ID_13315 | FGFR2 | | ES | | 0.001 | | 0.000 | | | 0.000 | | 0.000 |
| ID_13333 | TACC2 | | AP | | 0.026 | | 0.083 | | | 0.009 | | 0.745 |
| ID_13345 | TACC2 | | ES | | 0.035 | | 4.120 | | | 1.104 | | 15.380 |
| ID_13346 | TACC2 | | AD | | 0.015 | | 6.567 | | | 1.448 | | 29.775 |
| ID_133752 | CAST | | ES | | 0.004 | | 3.539 | | | 1.494 | | 8.381 |
| ID_13386 | ACADSB | | ES | | 0.000 | | 0.000 | | | 0.000 | | 0.009 |
| ID_13477 | BNIP3 | | AT | | 0.020 | | 6.535 | | | 1.347 | | 31.707 |
| ID_13554 | FUOM | | ES | | 0.009 | | 0.000 | | | 0.000 | | 0.079 |
| ID_13555 | PAOX | | ES | | 0.021 | | 0.113 | | | 0.018 | | 0.724 |
| ID_13596 | SIRT3 | | AD | | 0.011 | | 14.710 | | | 1.842 | | 117.480 |
| ID_13697 | PHRF1 | | AA | | 0.043 | | 0.055 | | | 0.003 | | 0.915 |
| ID_137504 | CTBP2 | | ES | | 0.012 | | 33.932 | | | 2.145 | | 536.775 |
| ID_137505 | CTBP2 | | ES | | 0.007 | | 5.604 | | | 1.592 | | 19.723 |
| ID_13753 | PDDC1 | | AA | | 0.038 | | 0.007 | | | 0.000 | | 0.770 |
| ID_13924 | KCNQ1 | | AP | | 0.049 | | 0.011 | | | 0.000 | | 0.976 |
| ID_14004 | PGAP2 | | AD | | 0.032 | | 0.236 | | | 0.063 | | 0.886 |
| ID_141 | DVL1 | | AD | | 0.002 | | 0.166 | | | 0.055 | | 0.506 |
| ID_14106 | SMPD1 | | AD | | 0.013 | | 0.001 | | | 0.000 | | 0.217 |
| ID_1412681 | RPS6 | | ES | | 0.004 | | 0.018 | | | 0.001 | | 0.274 |
| ID_14127 | TRIM3 | | AP | | 0.004 | | 0.067 | | | 0.011 | | 0.413 |
| ID_14220 | RIC3 | | AT | | 0.004 | | 3.120 | | | 1.442 | | 6.749 |
| ID_14373 | MRVI1 | | AA | | 0.018 | | 2.126 | | | 1.138 | | 3.972 |
| ID_14400 | MRVI1 | | AD | | 0.032 | | 3.285 | | | 1.109 | | 9.730 |
| ID_1443 | PUM1 | | AD | | 0.024 | | 19.057 | | | 1.483 | | 244.836 |
| ID_14436 | TEAD1 | | ES | | 0.044 | | 1.913 | | | 1.016 | | 3.599 |
| ID_14536 | USH1C | | AP | | 0.026 | | 0.001 | | | 0.000 | | 0.412 |
| ID_14642 | LDHA | | RI | | 0.007 | | 2.222 | | | 1.240 | | 3.981 |
| ID_14723 | PRMT3 | | ES | | 0.005 | | 0.013 | | | 0.001 | | 0.262 |
| ID_1475993 | COL3A1 | | ES | | 0.019 | | 6.918 | | | 1.368 | | 34.985 |
| ID_14877 | TCP11L1 | | AD | | 0.049 | | 0.507 | | | 0.258 | | 0.998 |
| ID_14938 | LMO2 | | AA | | 0.034 | | 122.728 | | | 1.433 | | 10511.063 |
| ID_14961 | EHF | | AT | | 0.014 | | 0.005 | | | 0.000 | | 0.335 |
| ID_15457 | TTC17 | | ES | | 0.022 | | 0.001 | | | 0.000 | | 0.379 |
| ID_15490 | TP53I11 | | AD | | 0.019 | | 0.121 | | | 0.021 | | 0.705 |
| ID_15540 | DGKZ | | AP | | 0.012 | | 0.083 | | | 0.012 | | 0.573 |
| ID_15584 | ATG13 | | ES | | 0.002 | | 0.042 | | | 0.006 | | 0.309 |
| ID_15673 | DDB2 | | ES | | 0.040 | | 153.517 | | | 1.271 | | 18542.372 |
| ID_15933 | CTNND1 | | ES | | 0.029 | | 0.277 | | | 0.088 | | 0.879 |
| ID_15935 | CTNND1 | | ES | | 0.011 | | 0.441 | | | 0.235 | | 0.827 |
| ID_15945 | CTNND1 | | ES | | 0.045 | | 1.838 | | | 1.014 | | 3.332 |
| ID_15957 | CTNND1 | | ES | | 0.042 | | 182.395 | | | 1.222 | | 27233.153 |
| ID_15979 | CTNND1 | | ES | | 0.021 | | 2.597 | | | 1.156 | | 5.836 |
| ID_15999 | CTNND1 | | ES | | 0.044 | | 2.154 | | | 1.021 | | 4.546 |
| ID_16064 | MS4A6A | | ES | | 0.003 | | 0.002 | | | 0.000 | | 0.120 |
| ID_16083 | MS4A4A | | ES | | 0.039 | | 0.228 | | | 0.056 | | 0.927 |
| ID_1611 | ZBTB8OS | | AT | | 0.015 | | 0.071 | | | 0.008 | | 0.602 |
| ID_16143 | CD6 | | ES | | 0.018 | | 39.079 | | | 1.892 | | 807.215 |
| ID_16308 | RAB3IL1 | | AP | | 0.004 | | 0.010 | | | 0.000 | | 0.225 |
| ID_16342 | ASRGL1 | | AA | | 0.011 | | 5.858 | | | 1.501 | | 22.867 |
| ID_16366 | ROM1 | | AP | | 0.006 | | 3.567 | | | 1.445 | | 8.804 |
| ID_1638 | S100PBP | | AD | | 0.046 | | 0.107 | | | 0.012 | | 0.963 |
| ID_16403 | BSCL2 | | AP | | 0.018 | | 4.281 | | | 1.288 | | 14.225 |
| ID_16426 | TAF6L | | AA | | 0.028 | | 0.001 | | | 0.000 | | 0.499 |
| ID_1646 | RNF19B | | AA | | 0.046 | | 0.083 | | | 0.007 | | 0.955 |
| ID_16651 | RPS6KA4 | | AD | | 0.009 | | 5116.682 | | | 8.211 | | 3188490.484 |
| ID_1670059 | COL3A1 | | ES | | 0.017 | | 0.086 | | | 0.011 | | 0.645 |
| ID_1670077 | COL3A1 | | ES | | 0.012 | | 0.070 | | | 0.009 | | 0.553 |
| ID_16732 | SAC3D1 | | RI | | 0.026 | | 0.169 | | | 0.035 | | 0.812 |
| ID_16748 | ZFPL1 | | AT | | 0.046 | | 261.767 | | | 1.099 | | 62377.195 |
| ID_16914 | KAT5 | | RI | | 0.030 | | 0.153 | | | 0.028 | | 0.832 |
| ID_16941 | FOSL1 | | AA | | 0.021 | | 0.004 | | | 0.000 | | 0.438 |
| ID_17064 | ZDHHC24 | | AT | | 0.003 | | 0.038 | | | 0.004 | | 0.331 |
| ID_17139 | PC | | ES | | 0.002 | | 12.284 | | | 2.551 | | 59.147 |
| ID_17140 | LRFN4 | | AP | | 0.002 | | 29.360 | | | 3.389 | | 254.343 |
| ID_17161 | SSH3 | | AA | | 0.046 | | 26.238 | | | 1.061 | | 648.624 |
| ID_17369 | ORAOV1 | | AT | | 0.012 | | 0.092 | | | 0.014 | | 0.588 |
| ID_17372 | ORAOV1 | | ES | | 0.046 | | 0.003 | | | 0.000 | | 0.904 |
| ID_17387 | ANO1 | | ES | | 0.047 | | 1.960 | | | 1.009 | | 3.807 |
| ID_1760 | MAP7D1 | | RI | | 0.004 | | 0.039 | | | 0.004 | | 0.349 |
| ID_17666 | ATG16L2 | | AA | | 0.026 | | 2.975 | | | 1.136 | | 7.793 |
| ID_17693 | RELT | | RI | | 0.019 | | 8.352 | | | 1.412 | | 49.416 |
| ID_17800 | XRRA1 | | ES | | 0.022 | | 4.643 | | | 1.253 | | 17.211 |
| ID_18017 | USP35 | | AP | | 0.047 | | 2.450 | | | 1.010 | | 5.944 |
| ID_18152 | SYTL2 | | ES | | 0.045 | | 2.963 | | | 1.025 | | 8.563 |
| ID_18171 | PICALM | | AA | | 0.011 | | 4401.299 | | | 7.013 | | 2762066.143 |
| ID_18186 | C11orf73 | | ES | | 0.034 | | 0.004 | | | 0.000 | | 0.651 |
| ID_18193 | ME3 | | AP | | 0.030 | | 2.723 | | | 1.105 | | 6.712 |
| ID_18390 | CEP57 | | AT | | 0.007 | | 0.022 | | | 0.001 | | 0.353 |
| ID_18489 | DYNC2H1 | | ES | | 0.004 | | 0.007 | | | 0.000 | | 0.195 |
| ID_1858 | GJA9 | | AT | | 0.046 | | 0.166 | | | 0.028 | | 0.971 |
| ID_1860 | RHBDL2 | | AP | | 0.033 | | 2.732 | | | 1.084 | | 6.886 |
| ID_18675 | PPP2R1B | | ES | | 0.003 | | 3035.873 | | | 15.769 | | 584459.609 |
| ID_18707 | DIXDC1 | | AP | | 0.019 | | 2.152 | | | 1.137 | | 4.072 |
| ID_18722 | C11orf57 | | AA | | 0.021 | | 30.879 | | | 1.691 | | 563.714 |
| ID_18767 | NCAM1 | | AT | | 0.045 | | 0.244 | | | 0.062 | | 0.967 |
| ID_18878 | SIK3 | | ES | | 0.029 | | 7.572 | | | 1.227 | | 46.718 |
| ID_1888 | MACF1 | | ES | | 0.016 | | 2.073 | | | 1.147 | | 3.747 |
| ID_18886 | SIDT2 | | RI | | 0.010 | | 0.051 | | | 0.005 | | 0.484 |
| ID_18894 | TAGLN | | AP | | 0.019 | | 529.880 | | | 2.843 | | 98771.196 |
| ID_1897 | PABPC4 | | AD | | 0.010 | | 0.027 | | | 0.002 | | 0.427 |
| ID_19038 | PHLDB1 | | RI | | 0.012 | | 0.286 | | | 0.107 | | 0.763 |
| ID_19096 | HMBS | | AD | | 0.011 | | 0.266 | | | 0.096 | | 0.738 |
| ID_19150 | TRIM29 | | AP | | 0.031 | | 0.521 | | | 0.288 | | 0.942 |
| ID_192 | MIB2 | | ES | | 0.033 | | 222.797 | | | 1.566 | | 31695.482 |
| ID_1924 | TRIT1 | | ES | | 0.011 | | 0.001 | | | 0.000 | | 0.202 |
| ID_19552 | ACAD8 | | AT | | 0.016 | | 120.265 | | | 2.397 | | 6032.835 |
| ID_19611 | WNK1 | | AP | | 0.005 | | 0.014 | | | 0.001 | | 0.286 |
| ID_19744 | TEAD4 | | ES | | 0.017 | | 0.002 | | | 0.000 | | 0.317 |
| ID_198 | MIB2 | | AD | | 0.015 | | 17.667 | | | 1.745 | | 178.832 |
| ID_19837 | SCNN1A | | AP | | 0.004 | | 0.187 | | | 0.060 | | 0.580 |
| ID_19862 | VAMP1 | | RI | | 0.038 | | 0.172 | | | 0.033 | | 0.910 |
| ID_19892 | NOP2 | | AA | | 0.048 | | 7.227 | | | 1.019 | | 51.283 |
| ID_19927 | ZNF384 | | AD | | 0.031 | | 0.233 | | | 0.062 | | 0.876 |
| ID_19988 | USP5 | | AD | | 0.044 | | 54.441 | | | 1.118 | | 2651.151 |
| ID_20067 | C1S | | AP | | 0.029 | | 49.590 | | | 1.501 | | 1638.482 |
| ID_20177 | NECAP1 | | ES | | 0.041 | | 0.008 | | | 0.000 | | 0.818 |
| ID_20178 | CLEC4A | | ES | | 0.000 | | 0.010 | | | 0.001 | | 0.132 |
| ID_20481 | YBX3 | | ES | | 0.033 | | 5.177 | | | 1.143 | | 23.458 |
| ID_20514 | APOLD1 | | AP | | 0.031 | | 0.498 | | | 0.265 | | 0.939 |
| ID_20516 | APOLD1 | | AT | | 0.031 | | 2.724 | | | 1.097 | | 6.767 |
| ID_20549 | ATF7IP | | AA | | 0.034 | | 0.151 | | | 0.026 | | 0.869 |
| ID_2069 | FOXJ3 | | ES | | 0.030 | | 0.105 | | | 0.014 | | 0.800 |
| ID_207 | SLC35E2B | | ES | | 0.036 | | 3.417 | | | 1.086 | | 10.754 |
| ID_20883 | ARNTL2 | | AD | | 0.002 | | 33.905 | | | 3.822 | | 300.731 |
| ID_20893 | PPFIBP1 | | ES | | 0.023 | | 3.070 | | | 1.167 | | 8.073 |
| ID_20926 | FAR2 | | ES | | 0.029 | | 5366176.487 | | | 4.887 | | 5892537640597.62 |
| ID_20977 | DDX11 | | ES | | 0.000 | | 20.773 | | | 4.381 | | 98.492 |
| ID_21013 | AMN1 | | ES | | 0.034 | | 2.441 | | | 1.069 | | 5.577 |
| ID_21030 | FGD4 | | AP | | 0.019 | | 0.420 | | | 0.204 | | 0.865 |
| ID_21046 | DNM1L | | ES | | 0.015 | | 0.250 | | | 0.082 | | 0.762 |
| ID_21064 | ALG10 | | AT | | 0.028 | | 0.068 | | | 0.006 | | 0.744 |
| ID_21078 | KIF21A | | ES | | 0.030 | | 97.509 | | | 1.561 | | 6091.497 |
| ID_211385 | ANXA11 | | ES | | 0.015 | | 3.581 | | | 1.277 | | 10.045 |
| ID_21411 | SENP1 | | AD | | 0.010 | | 5.192 | | | 1.472 | | 18.310 |
| ID_21470 | CACNB3 | | AP | | 0.006 | | 3.464 | | | 1.423 | | 8.431 |
| ID_21488 | RND1 | | AD | | 0.006 | | 19.809 | | | 2.312 | | 169.764 |
| ID_21522 | LMBR1L | | ES | | 0.018 | | 0.167 | | | 0.038 | | 0.739 |
| ID_21538 | TUBA1A | | AP | | 0.000 | | 908.942 | | | 20.004 | | 41300.759 |
| ID_2154 | C1orf210 | | AD | | 0.038 | | 0.025 | | | 0.001 | | 0.812 |
| ID_21565 | TROAP | | AA | | 0.006 | | 20.341 | | | 2.371 | | 174.528 |
| ID_21603 | FMNL3 | | ES | | 0.038 | | 3.011 | | | 1.065 | | 8.515 |
| ID_21613 | TMBIM6 | | AD | | 0.017 | | 0.141 | | | 0.028 | | 0.702 |
| ID_2185 | HYI | | AD | | 0.040 | | 13.196 | | | 1.125 | | 154.737 |
| ID_21925 | TENC1 | | AP | | 0.025 | | 3.192 | | | 1.158 | | 8.803 |
| ID_2194 | ST3GAL3 | | AP | | 0.010 | | 15.959 | | | 1.919 | | 132.691 |
| ID_21955 | CSAD | | RI | | 0.044 | | 2.447 | | | 1.023 | | 5.852 |
| ID_21976 | ITGB7 | | AD | | 0.009 | | 15.615 | | | 2.008 | | 121.411 |
| ID_21979 | RARG | | AP | | 0.036 | | 0.405 | | | 0.174 | | 0.941 |
| ID_22073 | TARBP2 | | AA | | 0.005 | | 30.679 | | | 2.736 | | 343.970 |
| ID_2210 | ST3GAL3 | | ES | | 0.043 | | 0.000 | | | 0.000 | | 0.787 |
| ID_22216 | ITGA7 | | ES | | 0.005 | | 0.164 | | | 0.046 | | 0.575 |
| ID_22234 | BLOC1S1 | | ES | | 0.018 | | 7.770 | | | 1.425 | | 42.365 |
| ID_22271 | MMP19 | | ES | | 0.043 | | 696.286 | | | 1.243 | | 389948.410 |
| ID_22302 | DGKA | | ES | | 0.020 | | 0.001 | | | 0.000 | | 0.362 |
| ID_2233 | ST3GAL3 | | ES | | 0.003 | | 0.000 | | | 0.000 | | 0.020 |
| ID_22379 | MYL6 | | RI | | 0.011 | | 0.002 | | | 0.000 | | 0.226 |
| ID_22391 | SMARCC2 | | AD | | 0.019 | | 0.234 | | | 0.069 | | 0.791 |
| ID_22770 | LRIG3 | | AP | | 0.043 | | 0.050 | | | 0.003 | | 0.915 |
| ID_22772 | LRIG3 | | ES | | 0.020 | | 116.130 | | | 2.140 | | 6303.256 |
| ID_22932 | MDM1 | | AA | | 0.003 | | 5.353 | | | 1.788 | | 16.032 |
| ID_22958 | RAP1B | | ES | | 0.018 | | 0.018 | | | 0.001 | | 0.502 |
| ID_22959 | RAP1B | | AD | | 0.003 | | 0.005 | | | 0.000 | | 0.171 |
| ID_23415 | TBC1D15 | | ES | | 0.022 | | 0.155 | | | 0.031 | | 0.766 |
| ID_23501 | OSBPL8 | | ES | | 0.023 | | 0.321 | | | 0.120 | | 0.854 |
| ID_23603 | C12orf29 | | ES | | 0.003 | | 0.082 | | | 0.015 | | 0.435 |
| ID_23664 | DCN | | ES | | 0.047 | | 0.269 | | | 0.074 | | 0.980 |
| ID_23717 | CRADD | | AA | | 0.005 | | 12.669 | | | 2.113 | | 75.967 |
| ID_23837 | NEDD1 | | ES | | 0.048 | | 0.030 | | | 0.001 | | 0.969 |
| ID_23870 | APAF1 | | AT | | 0.042 | | 0.005 | | | 0.000 | | 0.814 |
| ID_24045 | PARPBP | | AA | | 0.017 | | 0.077 | | | 0.009 | | 0.632 |
| ID_24163 | RIC8B | | ES | | 0.049 | | 10.046 | | | 1.007 | | 100.214 |
| ID_24208 | CMKLR1 | | ES | | 0.016 | | 0.007 | | | 0.000 | | 0.392 |
| ID_24321 | MMAB | | ES | | 0.008 | | 2.672 | | | 1.291 | | 5.534 |
| ID_24363 | TCHP | | AP | | 0.000 | | 0.008 | | | 0.001 | | 0.097 |
| ID_24370 | GIT2 | | ES | | 0.003 | | 3.733 | | | 1.588 | | 8.776 |
| ID_24376 | GIT2 | | ES | | 0.022 | | 0.020 | | | 0.001 | | 0.566 |
| ID_24391 | GIT2 | | AA | | 0.043 | | 18.184 | | | 1.102 | | 299.959 |
| ID_24446 | VPS29 | | ES | | 0.001 | | 0.175 | | | 0.060 | | 0.510 |
| ID_24569 | ERP29 | | ES | | 0.034 | | 0.007 | | | 0.000 | | 0.695 |
| ID_24678 | FBXO21 | | AA | | 0.023 | | 8.423 | | | 1.342 | | 52.866 |
| ID_24772 | RNF10 | | ES | | 0.038 | | 0.009 | | | 0.000 | | 0.767 |
| ID_24873 | KDM2B | | AP | | 0.027 | | 2.859 | | | 1.130 | | 7.233 |
| ID_24921 | BCL7A | | AD | | 0.044 | | 0.130 | | | 0.018 | | 0.948 |
| ID_24956 | CLIP1 | | ES | | 0.026 | | 0.041 | | | 0.002 | | 0.680 |
| ID_251 | C1orf86 | | ES | | 0.047 | | 111.681 | | | 1.071 | | 11645.170 |
| ID_25110 | TCTN2 | | AA | | 0.022 | | 0.219 | | | 0.059 | | 0.806 |
| ID_25146 | NCOR2 | | AA | | 0.012 | | 100.155 | | | 2.811 | | 3568.777 |
| ID_252014 | C16orf72 | | ES | | 0.034 | | 0.002 | | | 0.000 | | 0.621 |
| ID_25202 | STX2 | | ES | | 0.036 | | 0.441 | | | 0.205 | | 0.946 |
| ID_25324 | ZNF140 | | ES | | 0.009 | | 0.003 | | | 0.000 | | 0.236 |
| ID_25326 | ZNF140 | | AA | | 0.000 | | 0.000 | | | 0.000 | | 0.021 |
| ID_25327 | ZNF140 | | ES | | 0.009 | | 0.190 | | | 0.055 | | 0.657 |
| ID_25334 | ZNF140 | | ES | | 0.027 | | 59.725 | | | 1.578 | | 2261.245 |
| ID_25340 | ZNF10 | | AD | | 0.044 | | 19.314 | | | 1.081 | | 345.093 |
| ID_25400 | MPHOSPH8 | | AT | | 0.017 | | 0.078 | | | 0.010 | | 0.634 |
| ID_25474 | SPATA13 | | AP | | 0.002 | | 0.193 | | | 0.067 | | 0.556 |
| ID_25487 | CENPJ | | ES | | 0.034 | | 0.007 | | | 0.000 | | 0.696 |
| ID_25590 | N4BP2L1 | | ME | | 0.030 | | 0.245 | | | 0.069 | | 0.873 |
| ID_25596 | N4BP2L2 | | AA | | 0.025 | | 0.000 | | | 0.000 | | 0.322 |
| ID_25671 | POSTN | | ES | | 0.017 | | 7.913 | | | 1.446 | | 43.298 |
| ID_25673 | POSTN | | ES | | 0.005 | | 638.603 | | | 7.232 | | 56392.478 |
| ID_2568 | TMEM53 | | ES | | 0.031 | | 4.531 | | | 1.145 | | 17.941 |
| ID_25700 | PROSER1 | | ES | | 0.015 | | 0.057 | | | 0.006 | | 0.580 |
| ID_25750 | DGKH | | ES | | 0.027 | | 0.001 | | | 0.000 | | 0.469 |
| ID_257715 | TPM3 | | ES | | 0.024 | | 3.295 | | | 1.172 | | 9.264 |
| ID_2586 | PTCH2 | | AT | | 0.019 | | 4.498 | | | 1.282 | | 15.787 |
| ID_26065 | LMO7 | | AA | | 0.006 | | 3.287 | | | 1.411 | | 7.658 |
| ID_26067 | LMO7 | | ES | | 0.037 | | 2.328 | | | 1.052 | | 5.151 |
| ID_26112 | ABCC4 | | ES | | 0.003 | | 0.000 | | | 0.000 | | 0.041 |
| ID_26140 | MBNL2 | | ES | | 0.024 | | 0.275 | | | 0.090 | | 0.842 |
| ID_26171 | DOCK9 | | AP | | 0.000 | | 0.174 | | | 0.066 | | 0.457 |
| ID_26251 | CARKD | | AP | | 0.022 | | 21.049 | | | 1.554 | | 285.127 |
| ID_26286 | ARHGEF7 | | ES | | 0.042 | | 553.259 | | | 1.265 | | 241963.988 |
| ID_26500 | NDRG2 | | ES | | 0.014 | | 4.483 | | | 1.363 | | 14.742 |
| ID_26580 | CHD8 | | AD | | 0.035 | | 529.823 | | | 1.550 | | 181056.557 |
| ID_26624 | SLC7A7 | | RI | | 0.048 | | 6.725 | | | 1.016 | | 44.528 |
| ID_26678 | HAUS4 | | AD | | 0.003 | | 0.061 | | | 0.010 | | 0.389 |
| ID_26700 | CDH24 | | ES | | 0.014 | | 0.348 | | | 0.150 | | 0.810 |
| ID_26703 | ACIN1 | | AA | | 0.018 | | 126.229 | | | 2.262 | | 7044.739 |
| ID_26801 | DHRS4L2 | | ES | | 0.036 | | 3.130 | | | 1.076 | | 9.104 |
| ID_26822 | PCK2 | | ES | | 0.013 | | 0.001 | | | 0.000 | | 0.238 |
| ID_26993 | NFATC4 | | AD | | 0.028 | | 0.197 | | | 0.046 | | 0.843 |
| ID_27001 | KHNYN | | AP | | 0.012 | | 0.019 | | | 0.001 | | 0.427 |
| ID_27002 | KHNYN | | AA | | 0.008 | | 64.030 | | | 3.006 | | 1363.858 |
| ID_270122 | CAST | | ES | | 0.003 | | 3.782 | | | 1.574 | | 9.088 |
| ID_27118 | DTD2 | | RI | | 0.047 | | 0.004 | | | 0.000 | | 0.930 |
| ID_27164 | SNX6 | | AA | | 0.007 | | 0.000 | | | 0.000 | | 0.058 |
| ID_27210 | PPP2R3C | | ES | | 0.012 | | 0.049 | | | 0.005 | | 0.515 |
| ID_27239 | RALGAPA1 | | ES | | 0.008 | | 2.518 | | | 1.270 | | 4.991 |
| ID_27294 | SLC25A21 | | AT | | 0.001 | | 0.000 | | | 0.000 | | 0.013 |
| ID_27301 | MIPOL1 | | ES | | 0.006 | | 0.001 | | | 0.000 | | 0.118 |
| ID_27373 | CTAGE5 | | AP | | 0.004 | | 0.259 | | | 0.103 | | 0.652 |
| ID_27379 | CTAGE5 | | ES | | 0.041 | | 0.002 | | | 0.000 | | 0.777 |
| ID_27380 | CTAGE5 | | AD | | 0.031 | | 0.011 | | | 0.000 | | 0.661 |
| ID_27454 | SOS2 | | ES | | 0.029 | | 0.007 | | | 0.000 | | 0.598 |
| ID_27463 | L2HGDH | | ES | | 0.005 | | 65.942 | | | 3.643 | | 1193.682 |
| ID_27495 | NIN | | ES | | 0.013 | | 2.581 | | | 1.225 | | 5.437 |
| ID_27515 | FRMD6 | | AD | | 0.036 | | 3.990 | | | 1.097 | | 14.515 |
| ID_27542 | PTGER2 | | RI | | 0.035 | | 64.427 | | | 1.327 | | 3127.225 |
| ID_27560 | FERMT2 | | ES | | 0.007 | | 238.487 | | | 4.369 | | 13016.648 |
| ID_27593 | CGRRF1 | | ES | | 0.022 | | 0.001 | | | 0.000 | | 0.382 |
| ID_27717 | KIAA0586 | | ES | | 0.018 | | 0.271 | | | 0.092 | | 0.798 |
| ID_27757 | RTN1 | | AP | | 0.001 | | 4.334 | | | 1.864 | | 10.079 |
| ID_27846 | SYNE2 | | ES | | 0.006 | | 3.571 | | | 1.431 | | 8.909 |
| ID_27879 | ZBTB25 | | AP | | 0.045 | | 6.578 | | | 1.044 | | 41.440 |
| ID_27882 | ZBTB25 | | AT | | 0.009 | | 0.193 | | | 0.056 | | 0.669 |
| ID_28183 | MED6 | | AT | | 0.029 | | 0.088 | | | 0.010 | | 0.784 |
| ID_28294 | NUMB | | ES | | 0.001 | | 6.590 | | | 2.244 | | 19.354 |
| ID_28454 | RPS6KL1 | | RI | | 0.013 | | 3889.193 | | | 5.838 | | 2590738.411 |
| ID_28473 | ACYP1 | | AP | | 0.019 | | 0.084 | | | 0.011 | | 0.669 |
| ID_28714 | ZC3H14 | | AA | | 0.008 | | 0.020 | | | 0.001 | | 0.360 |
| ID_29154 | SERPINA3 | | AD | | 0.021 | | 0.031 | | | 0.002 | | 0.588 |
| ID_29192 | BDKRB2 | | AA | | 0.000 | | 0.056 | | | 0.012 | | 0.268 |
| ID_29200 | AK7 | | AT | | 0.020 | | 0.384 | | | 0.172 | | 0.858 |
| ID_29230 | CYP46A1 | | AT | | 0.026 | | 0.445 | | | 0.218 | | 0.906 |
| ID_29321 | PPP2R5C | | ES | | 0.008 | | 9.243 | | | 1.778 | | 48.050 |
| ID_29357 | WDR20 | | AD | | 0.022 | | 23.161 | | | 1.575 | | 340.593 |
| ID_29469 | KLC1 | | ES | | 0.014 | | 0.272 | | | 0.096 | | 0.771 |
| ID_2954 | TTC39A | | RI | | 0.010 | | 197.137 | | | 3.447 | | 11274.113 |
| ID_29588 | C14orf79 | | RI | | 0.008 | | 0.228 | | | 0.076 | | 0.680 |
| ID_29656 | CRIP1 | | RI | | 0.026 | | 3.116 | | | 1.146 | | 8.473 |
| ID_29662 | C14orf80 | | ES | | 0.004 | | 0.228 | | | 0.083 | | 0.630 |
| ID_29737 | GABRB3 | | AT | | 0.021 | | 0.015 | | | 0.000 | | 0.537 |
| ID_29913 | MEIS2 | | AA | | 0.036 | | 4.167 | | | 1.099 | | 15.794 |
| ID_29926 | RASGRP1 | | ES | | 0.010 | | 15718.845 | | | 10.073 | | 24530331.822 |
| ID_30104 | MGA | | ES | | 0.024 | | 25.529 | | | 1.532 | | 425.448 |
| ID_30116 | PLA2G4B | | AP | | 0.040 | | 0.356 | | | 0.133 | | 0.953 |
| ID_30269 | PPIP5K1 | | ES | | 0.006 | | 0.059 | | | 0.008 | | 0.452 |
| ID_30270 | PPIP5K1 | | ES | | 0.018 | | 0.128 | | | 0.023 | | 0.700 |
| ID_30547 | FAM227B | | ES | | 0.041 | | 3.846 | | | 1.054 | | 14.030 |
| ID_3058 | LRP8 | | ES | | 0.023 | | 0.336 | | | 0.131 | | 0.861 |
| ID_30646 | MYO5C | | AT | | 0.002 | | 0.013 | | | 0.001 | | 0.208 |
| ID_30675 | ARPP19 | | RI | | 0.031 | | 0.056 | | | 0.004 | | 0.766 |
| ID_30692 | ARPP19 | | RI | | 0.024 | | 0.269 | | | 0.086 | | 0.840 |
| ID_30722 | CCPG1 | | AP | | 0.008 | | 0.172 | | | 0.047 | | 0.626 |
| ID_30789 | TCF12 | | ES | | 0.010 | | 0.219 | | | 0.069 | | 0.700 |
| ID_3079 | YIPF1 | | ES | | 0.018 | | 4.952 | | | 1.309 | | 18.739 |
| ID_30987 | TPM1 | | AD | | 0.028 | | 0.246 | | | 0.070 | | 0.858 |
| ID_30990 | TPM1 | | ES | | 0.005 | | 0.010 | | | 0.000 | | 0.245 |
| ID_31170 | DPP8 | | ES | | 0.015 | | 0.097 | | | 0.015 | | 0.636 |
| ID_31237 | SLC24A1 | | ES | | 0.042 | | 0.154 | | | 0.026 | | 0.932 |
| ID_3136 | CYB5RL | | ES | | 0.011 | | 10.519 | | | 1.698 | | 65.182 |
| ID_31447 | LRRC49 | | AP | | 0.010 | | 0.316 | | | 0.131 | | 0.763 |
| ID_31619 | STOML1 | | AP | | 0.010 | | 17.100 | | | 2.001 | | 146.161 |
| ID_31728 | CLK3 | | ES | | 0.008 | | 1028.856 | | | 6.176 | | 171396.696 |
| ID_31954 | SCAPER | | ES | | 0.046 | | 11.049 | | | 1.044 | | 116.937 |
| ID_31991 | HMG20A | | ES | | 0.045 | | 0.034 | | | 0.001 | | 0.934 |
| ID_32004 | CIB2 | | ES | | 0.016 | | 0.060 | | | 0.006 | | 0.593 |
| ID_32161 | MTHFS | | AD | | 0.011 | | 9.744 | | | 1.673 | | 56.739 |
| ID_32179 | FAH | | RI | | 0.025 | | 3.191 | | | 1.153 | | 8.833 |
| ID_32181 | FAH | | AD | | 0.010 | | 0.388 | | | 0.189 | | 0.794 |
| ID_32285 | GOLGA6L4 | | RI | | 0.044 | | 4.606 | | | 1.044 | | 20.328 |
| ID_32309 | ZSCAN2 | | AD | | 0.031 | | 3.880 | | | 1.133 | | 13.283 |
| ID_32384 | DET1 | | RI | | 0.020 | | 454.195 | | | 2.666 | | 77382.768 |
| ID_3239 | INADL | | AT | | 0.048 | | 0.185 | | | 0.035 | | 0.982 |
| ID_3245 | INADL | | ES | | 0.041 | | 0.042 | | | 0.002 | | 0.874 |
| ID_32712 | LRRC28 | | AD | | 0.044 | | 47.732 | | | 1.112 | | 2049.460 |
| ID_32972 | HAGHL | | RI | | 0.045 | | 0.077 | | | 0.006 | | 0.939 |
| ID_33024 | CHTF18 | | RI | | 0.031 | | 7.184 | | | 1.202 | | 42.945 |
| ID_3307 | SGIP1 | | AP | | 0.032 | | 0.492 | | | 0.257 | | 0.939 |
| ID_33078 | UNKL | | AP | | 0.038 | | 2.974 | | | 1.060 | | 8.350 |
| ID_33195 | TSC2 | | AA | | 0.038 | | 5.362 | | | 1.094 | | 26.274 |
| ID_33245 | RNPS1 | | AP | | 0.007 | | 34.928 | | | 2.697 | | 452.353 |
| ID_3325 | WDR78 | | AP | | 0.031 | | 0.209 | | | 0.050 | | 0.867 |
| ID_3338 | MIER1 | | RI | | 0.016 | | 0.025 | | | 0.001 | | 0.508 |
| ID_33439 | IL32 | | AD | | 0.049 | | 0.304 | | | 0.093 | | 0.997 |
| ID_33538 | NAA60 | | ES | | 0.019 | | 0.003 | | | 0.000 | | 0.388 |
| ID_33790 | NUDT16L1 | | RI | | 0.048 | | 0.018 | | | 0.000 | | 0.969 |
| ID_33857 | ROGDI | | AD | | 0.038 | | 0.219 | | | 0.052 | | 0.918 |
| ID_33886 | FAM86A | | ES | | 0.033 | | 44.695 | | | 1.349 | | 1480.932 |
| ID_34068 | MKL2 | | AP | | 0.019 | | 2.396 | | | 1.154 | | 4.976 |
| ID_34220 | ABCC6 | | AT | | 0.049 | | 0.270 | | | 0.073 | | 0.996 |
| ID_34278 | TMC7 | | AP | | 0.017 | | 0.215 | | | 0.061 | | 0.760 |
| ID_34306 | TMC5 | | ES | | 0.003 | | 0.000 | | | 0.000 | | 0.000 |
| ID_34313 | GDE1 | | AA | | 0.044 | | 68.789 | | | 1.119 | | 4229.675 |
| ID_34318 | CCP110 | | ES | | 0.013 | | 0.284 | | | 0.105 | | 0.770 |
| ID_34338 | IQCK | | ES | | 0.039 | | 0.030 | | | 0.001 | | 0.842 |
| ID_34395 | ERI2 | | AA | | 0.026 | | 303.746 | | | 1.963 | | 46999.399 |
| ID_346 | DFFB | | ES | | 0.043 | | 2.864 | | | 1.031 | | 7.952 |
| ID_35506 | NPIPB4 | | RI | | 0.038 | | 0.130 | | | 0.019 | | 0.893 |
| ID_35639 | PRKCB | | AT | | 0.013 | | 1350.448 | | | 4.443 | | 410435.293 |
| ID_35642 | RBBP6 | | AT | | 0.030 | | 0.136 | | | 0.023 | | 0.827 |
| ID_35681 | NSMCE1 | | ES | | 0.028 | | 0.001 | | | 0.000 | | 0.488 |
| ID_35821 | SULT1A1 | | ES | | 0.013 | | 211.363 | | | 3.071 | | 14545.426 |
| ID_36035 | ALDOA | | AP | | 0.030 | | 0.007 | | | 0.000 | | 0.629 |
| ID_36173 | PHKG2 | | AP | | 0.021 | | 0.006 | | | 0.000 | | 0.467 |
| ID_36286 | ZNF720 | | RI | | 0.023 | | 2.644 | | | 1.146 | | 6.096 |
| ID_36517 | NLRC5 | | ES | | 0.025 | | 0.020 | | | 0.001 | | 0.609 |
| ID_36518 | NLRC5 | | ES | | 0.002 | | 103.256 | | | 5.585 | | 1909.135 |
| ID_36537 | RSPRY1 | | AD | | 0.041 | | 0.288 | | | 0.087 | | 0.951 |
| ID_36543 | CX3CL1 | | ES | | 0.003 | | 0.000 | | | 0.000 | | 0.031 |
| ID_36665 | SETD6 | | RI | | 0.042 | | 3.076 | | | 1.041 | | 9.085 |
| ID_3678 | ODF2L | | ES | | 0.015 | | 6.167 | | | 1.433 | | 26.539 |
| ID_3713 | GBP2 | | AP | | 0.008 | | 0.007 | | | 0.000 | | 0.272 |
| ID_37147 | NUTF2 | | AD | | 0.029 | | 0.055 | | | 0.004 | | 0.739 |
| ID_37158 | SLC12A4 | | RI | | 0.045 | | 0.009 | | | 0.000 | | 0.903 |
| ID_37188 | NFATC3 | | ES | | 0.038 | | 248119.501 | | | 1.969 | | 31273840960.7965 |
| ID_37236 | ZFP90 | | AA | | 0.048 | | 3.657 | | | 1.013 | | 13.196 |
| ID_37279 | COG8 | | AT | | 0.015 | | 0.109 | | | 0.018 | | 0.653 |
| ID_37321 | WWP2 | | ES | | 0.048 | | 0.169 | | | 0.029 | | 0.987 |
| ID_37322 | WWP2 | | ES | | 0.044 | | 0.195 | | | 0.040 | | 0.960 |
| ID_37614 | TMEM170A | | AD | | 0.028 | | 0.018 | | | 0.001 | | 0.645 |
| ID_37686 | MAF | | AT | | 0.011 | | 0.033 | | | 0.002 | | 0.450 |
| ID_37706 | CMC2 | | ES | | 0.037 | | 15.702 | | | 1.182 | | 208.587 |
| ID_37728 | CMC2 | | ES | | 0.050 | | 0.032 | | | 0.001 | | 0.997 |
| ID_37767 | CMIP | | AP | | 0.002 | | 0.000 | | | 0.000 | | 0.014 |
| ID_37797 | OSGIN1 | | AP | | 0.040 | | 0.055 | | | 0.003 | | 0.874 |
| ID_3788 | FNBP1L | | RI | | 0.002 | | 0.141 | | | 0.042 | | 0.475 |
| ID_37897 | EMC8 | | ES | | 0.023 | | 0.020 | | | 0.001 | | 0.578 |
| ID_37921 | MTHFSD | | AD | | 0.026 | | 0.011 | | | 0.000 | | 0.581 |
| ID_38032 | GALNS | | AT | | 0.038 | | 185.198 | | | 1.325 | | 25888.211 |
| ID_38116 | CDK10 | | ES | | 0.045 | | 4.355 | | | 1.037 | | 18.297 |
| ID_38286 | ABR | | AP | | 0.037 | | 0.266 | | | 0.077 | | 0.922 |
| ID_38337 | INPP5K | | ES | | 0.033 | | 0.062 | | | 0.005 | | 0.794 |
| ID_3842 | SNX7 | | ES | | 0.034 | | 16.985 | | | 1.239 | | 232.856 |
| ID_38808 | XAF1 | | ES | | 0.044 | | 0.283 | | | 0.083 | | 0.967 |
| ID_38845 | DLG4 | | AP | | 0.031 | | 18.303 | | | 1.301 | | 257.575 |
| ID_38915 | GPS2 | | RI | | 0.033 | | 0.265 | | | 0.078 | | 0.898 |
| ID_38974 | TNFSF13 | | ES | | 0.008 | | 0.097 | | | 0.017 | | 0.538 |
| ID_39149 | SLC25A35 | | AT | | 0.007 | | 20.404 | | | 2.257 | | 184.444 |
| ID_39154 | SLC25A35 | | RI | | 0.040 | | 66.149 | | | 1.215 | | 3600.434 |
| ID_39189 | NDEL1 | | ES | | 0.005 | | 4.309 | | | 1.562 | | 11.885 |
| ID_39199 | MYH10 | | ES | | 0.034 | | 0.226 | | | 0.057 | | 0.892 |
| ID_392312 | RPL13 | | AD | | 0.007 | | 0.169 | | | 0.047 | | 0.614 |
| ID_39375 | ZNF286A | | AT | | 0.002 | | 0.003 | | | 0.000 | | 0.122 |
| ID_39457 | MPRIP | | ES | | 0.002 | | 3.846 | | | 1.645 | | 8.995 |
| ID_39475 | COPS3 | | AA | | 0.022 | | 0.007 | | | 0.000 | | 0.487 |
| ID_39505 | SREBF1 | | ES | | 0.030 | | 25.049 | | | 1.369 | | 458.173 |
| ID_39598 | MIEF2 | | AT | | 0.014 | | 0.013 | | | 0.000 | | 0.420 |
| ID_39691 | GRAPL | | AT | | 0.023 | | 3.205 | | | 1.173 | | 8.754 |
| ID_3981 | CLCC1 | | ES | | 0.046 | | 6.184 | | | 1.034 | | 36.990 |
| ID_39847 | KSR1 | | AP | | 0.036 | | 0.156 | | | 0.027 | | 0.885 |
| ID_39858 | LGALS9 | | ES | | 0.001 | | 0.217 | | | 0.091 | | 0.518 |
| ID_39880 | IFT20 | | RI | | 0.017 | | 0.035 | | | 0.002 | | 0.545 |
| ID_40128 | TEFM | | AA | | 0.046 | | 0.041 | | | 0.002 | | 0.944 |
| ID_40157 | RAB11FIP4 | | AP | | 0.007 | | 0.045 | | | 0.005 | | 0.425 |
| ID_40180 | RHOT1 | | ES | | 0.016 | | 0.338 | | | 0.140 | | 0.815 |
| ID_40200 | C17orf75 | | AA | | 0.004 | | 0.012 | | | 0.001 | | 0.253 |
| ID_40217 | TMEM98 | | ES | | 0.011 | | 214.679 | | | 3.378 | | 13641.531 |
| ID_40302 | SLFN13 | | AP | | 0.047 | | 0.455 | | | 0.209 | | 0.991 |
| ID_40536 | DDX52 | | RI | | 0.003 | | 0.001 | | | 0.000 | | 0.098 |
| ID_40540 | DDX52 | | ES | | 0.018 | | 0.007 | | | 0.000 | | 0.423 |
| ID_40548 | TBC1D3F | | AP | | 0.027 | | 0.158 | | | 0.031 | | 0.807 |
| ID_40649 | CDK12 | | AA | | 0.015 | | 14.351 | | | 1.671 | | 123.225 |
| ID_40797 | GSDMB | | ES | | 0.001 | | 0.035 | | | 0.005 | | 0.257 |
| ID_40840 | THRA | | AT | | 0.010 | | 0.156 | | | 0.038 | | 0.638 |
| ID_40858 | RARA | | AP | | 0.024 | | 0.237 | | | 0.068 | | 0.826 |
| ID_4089 | AHCYL1 | | AP | | 0.050 | | 0.028 | | | 0.001 | | 0.995 |
| ID_410440 | COL1A1 | | ES | | 0.012 | | 0.015 | | | 0.001 | | 0.403 |
| ID_416 | TNFRSF25 | | ES | | 0.045 | | 0.076 | | | 0.006 | | 0.940 |
| ID_41726 | CD300LG | | AT | | 0.015 | | 0.230 | | | 0.071 | | 0.748 |
| ID_4178 | RAP1A | | AP | | 0.019 | | 0.002 | | | 0.000 | | 0.363 |
| ID_41788 | TMUB2 | | AD | | 0.035 | | 0.007 | | | 0.000 | | 0.705 |
| ID_41878 | C17orf104 | | AT | | 0.017 | | 3.464 | | | 1.252 | | 9.583 |
| ID_41927 | DCAKD | | AP | | 0.023 | | 0.251 | | | 0.076 | | 0.829 |
| ID_4197 | KCND3 | | ES | | 0.025 | | 15575.541 | | | 3.324 | | 72993866.439 |
| ID_42013 | KANSL1 | | AA | | 0.043 | | 0.049 | | | 0.003 | | 0.915 |
| ID_42039 | WNT9B | | AT | | 0.027 | | 0.383 | | | 0.164 | | 0.894 |
| ID_42054 | CDC27 | | AA | | 0.042 | | 0.042 | | | 0.002 | | 0.887 |
| ID_42079 | NPEPPS | | AA | | 0.025 | | 169.943 | | | 1.912 | | 15102.219 |
| ID_42198 | HOXB3 | | AT | | 0.026 | | 44.246 | | | 1.562 | | 1253.437 |
| ID_42359 | SGCA | | ES | | 0.046 | | 3.651 | | | 1.025 | | 13.004 |
| ID_42492 | SPAG9 | | AP | | 0.008 | | 0.040 | | | 0.004 | | 0.428 |
| ID_42576 | HLF | | AP | | 0.002 | | 0.160 | | | 0.051 | | 0.503 |
| ID_42725 | TRIM37 | | ES | | 0.030 | | 6.782 | | | 1.207 | | 38.111 |
| ID_42770 | GDPD1 | | AT | | 0.040 | | 0.145 | | | 0.023 | | 0.918 |
| ID_42815 | TUBD1 | | ES | | 0.044 | | 11.272 | | | 1.064 | | 119.445 |
| ID_42828 | TUBD1 | | ES | | 0.014 | | 12.766 | | | 1.687 | | 96.598 |
| ID_4293 | PTPN22 | | ES | | 0.010 | | 0.006 | | | 0.000 | | 0.295 |
| ID_42963 | STRADA | | RI | | 0.012 | | 5.224 | | | 1.429 | | 19.101 |
| ID_42964 | STRADA | | AD | | 0.019 | | 52.095 | | | 1.894 | | 1432.504 |
| ID_42976 | STRADA | | ES | | 0.041 | | 0.343 | | | 0.123 | | 0.957 |
| ID_42980 | STRADA | | ES | | 0.041 | | 0.357 | | | 0.132 | | 0.961 |
| ID_43047 | ICAM2 | | AA | | 0.018 | | 0.003 | | | 0.000 | | 0.371 |
| ID_43117 | BPTF | | ES | | 0.037 | | 2.150 | | | 1.045 | | 4.422 |
| ID_43143 | PRKAR1A | | AP | | 0.010 | | 0.108 | | | 0.020 | | 0.584 |
| ID_43183 | ABCA5 | | AP | | 0.031 | | 0.355 | | | 0.139 | | 0.907 |
| ID_43223 | C17orf80 | | ES | | 0.035 | | 0.000 | | | 0.000 | | 0.573 |
| ID_43302 | NAT9 | | AA | | 0.025 | | 6.140 | | | 1.255 | | 30.043 |
| ID_43317 | FDXR | | RI | | 0.039 | | 0.002 | | | 0.000 | | 0.726 |
| ID_43450 | CASKIN2 | | AP | | 0.012 | | 0.030 | | | 0.002 | | 0.458 |
| ID_4346 | TSPAN2 | | ES | | 0.028 | | 0.003 | | | 0.000 | | 0.541 |
| ID_43489 | ITGB4 | | ES | | 0.006 | | 5.382 | | | 1.639 | | 17.675 |
| ID_43491 | ITGB4 | | AD | | 0.028 | | 0.075 | | | 0.008 | | 0.751 |
| ID_43524 | TRIM47 | | AD | | 0.040 | | 0.050 | | | 0.003 | | 0.869 |
| ID_43538 | ACOX1 | | ES | | 0.044 | | 0.023 | | | 0.001 | | 0.901 |
| ID_43565 | EXOC7 | | ES | | 0.036 | | 0.468 | | | 0.230 | | 0.953 |
| ID_43566 | EXOC7 | | ES | | 0.031 | | 0.313 | | | 0.109 | | 0.899 |
| ID_43572 | EXOC7 | | ES | | 0.017 | | 0.357 | | | 0.154 | | 0.831 |
| ID_43628 | METTL23 | | RI | | 0.008 | | 0.005 | | | 0.000 | | 0.256 |
| ID_4370 | TRIM45 | | AP | | 0.011 | | 0.062 | | | 0.007 | | 0.531 |
| ID_43750 | TNRC6C | | ES | | 0.049 | | 17.419 | | | 1.010 | | 300.513 |
| ID_43940 | LGALS3BP | | RI | | 0.015 | | 0.076 | | | 0.010 | | 0.603 |
| ID_43989 | C1QTNF1 | | ES | | 0.042 | | 0.006 | | | 0.000 | | 0.835 |
| ID_44111 | ENTHD2 | | ES | | 0.028 | | 14.016 | | | 1.335 | | 147.127 |
| ID_44112 | ENTHD2 | | AD | | 0.043 | | 2.647 | | | 1.030 | | 6.802 |
| ID_4419 | PDE4DIP | | AA | | 0.037 | | 0.337 | | | 0.121 | | 0.937 |
| ID_44201 | ANAPC11 | | AP | | 0.008 | | 10021.506 | | | 11.413 | | 8799419.110 |
| ID_44207 | ANAPC11 | | ES | | 0.048 | | 3.950 | | | 1.013 | | 15.408 |
| ID_44211 | ANAPC11 | | ES | | 0.018 | | 9.092 | | | 1.451 | | 56.981 |
| ID_44214 | ANAPC11 | | AD | | 0.022 | | 0.071 | | | 0.007 | | 0.680 |
| ID_44250 | ASPSCR1 | | AP | | 0.044 | | 40.427 | | | 1.101 | | 1483.958 |
| ID_44276 | GPS1 | | RI | | 0.029 | | 18.254 | | | 1.356 | | 245.769 |
| ID_44649 | MPPE1 | | ES | | 0.045 | | 50.772 | | | 1.084 | | 2378.296 |
| ID_44658 | IMPA2 | | AP | | 0.006 | | 0.003 | | | 0.000 | | 0.177 |
| ID_44767 | GREB1L | | AT | | 0.004 | | 0.003 | | | 0.000 | | 0.153 |
| ID_44862 | CABYR | | ES | | 0.002 | | 0.002 | | | 0.000 | | 0.113 |
| ID_45011 | B4GALT6 | | ES | | 0.002 | | 0.004 | | | 0.000 | | 0.143 |
| ID_45012 | B4GALT6 | | ES | | 0.001 | | 0.059 | | | 0.011 | | 0.307 |
| ID_45145 | ZNF397 | | AT | | 0.038 | | 0.170 | | | 0.032 | | 0.909 |
| ID_45391 | C18orf25 | | ES | | 0.007 | | 7.455 | | | 1.747 | | 31.809 |
| ID_45394 | RNF165 | | AT | | 0.004 | | 0.278 | | | 0.116 | | 0.663 |
| ID_45508 | MBD1 | | RI | | 0.038 | | 5.590 | | | 1.102 | | 28.364 |
| ID_45821 | ZNF407 | | AT | | 0.012 | | 0.005 | | | 0.000 | | 0.310 |
| ID_46241 | NFATC1 | | AD | | 0.005 | | 6.074 | | | 1.747 | | 21.115 |
| ID_46289 | TXNL4A | | ES | | 0.022 | | 0.005 | | | 0.000 | | 0.467 |
| ID_46421 | CIRBP | | RI | | 0.006 | | 0.073 | | | 0.011 | | 0.478 |
| ID_46490 | RPS15 | | RI | | 0.002 | | 0.158 | | | 0.050 | | 0.499 |
| ID_46541 | TCF3 | | RI | | 0.049 | | 0.001 | | | 0.000 | | 0.967 |
| ID_46573 | MOB3A | | AP | | 0.027 | | 0.055 | | | 0.004 | | 0.715 |
| ID_46679 | NFIC | | ES | | 0.041 | | 0.238 | | | 0.060 | | 0.941 |
| ID_46707 | TBXA2R | | RI | | 0.010 | | 0.005 | | | 0.000 | | 0.280 |
| ID_46771 | SIRT6 | | AA | | 0.011 | | 0.008 | | | 0.000 | | 0.324 |
| ID_46796 | MPND | | ES | | 0.047 | | 0.005 | | | 0.000 | | 0.941 |
| ID_46833 | KDM4B | | ES | | 0.025 | | 0.159 | | | 0.032 | | 0.790 |
| ID_46861 | RPL36 | | RI | | 0.004 | | 0.040 | | | 0.005 | | 0.350 |
| ID_46926 | DUS3L | | AP | | 0.015 | | 0.005 | | | 0.000 | | 0.361 |
| ID_46945 | FUT3 | | ES | | 0.007 | | 0.051 | | | 0.006 | | 0.443 |
| ID_46956 | CAPS | | AD | | 0.047 | | 8.989 | | | 1.026 | | 78.769 |
| ID_46962 | RANBP3 | | AD | | 0.021 | | 47.179 | | | 1.811 | | 1228.986 |
| ID_47024 | CLPP | | AP | | 0.040 | | 0.011 | | | 0.000 | | 0.820 |
| ID_47052 | DENND1C | | AP | | 0.008 | | 0.303 | | | 0.125 | | 0.736 |
| ID_47068 | CD70 | | AT | | 0.024 | | 0.232 | | | 0.065 | | 0.826 |
| ID_47074 | GPR108 | | AP | | 0.011 | | 0.068 | | | 0.009 | | 0.535 |
| ID_47099 | INSR | | ES | | 0.025 | | 2.730 | | | 1.132 | | 6.588 |
| ID_47191 | EVI5L | | ES | | 0.006 | | 2.465 | | | 1.288 | | 4.718 |
| ID_47194 | MAP2K7 | | ES | | 0.014 | | 2.428 | | | 1.195 | | 4.934 |
| ID_47245 | MYO1F | | AD | | 0.034 | | 0.001 | | | 0.000 | | 0.564 |
| ID_47359 | ZNF121 | | ES | | 0.046 | | 0.226 | | | 0.053 | | 0.974 |
| ID_47387 | ZNF562 | | ES | | 0.043 | | 91.434 | | | 1.152 | | 7256.261 |
| ID_47390 | ZNF562 | | ES | | 0.019 | | 137.553 | | | 2.215 | | 8543.090 |
| ID_47401 | ZNF846 | | AT | | 0.044 | | 0.378 | | | 0.147 | | 0.975 |
| ID_47503 | ICAM3 | | RI | | 0.041 | | 0.476 | | | 0.234 | | 0.970 |
| ID_47559 | SLC44A2 | | AP | | 0.001 | | 0.270 | | | 0.122 | | 0.597 |
| ID_47599 | CARM1 | | ES | | 0.049 | | 8.536 | | | 1.007 | | 72.348 |
| ID_47605 | YIPF2 | | AD | | 0.005 | | 0.086 | | | 0.015 | | 0.481 |
| ID_47609 | SMARCA4 | | AA | | 0.035 | | 0.055 | | | 0.004 | | 0.813 |
| ID_47644 | DOCK6 | | ES | | 0.001 | | 5.933 | | | 2.065 | | 17.039 |
| ID_47678 | TMEM205 | | AD | | 0.026 | | 0.005 | | | 0.000 | | 0.521 |
| ID_47688 | DKFZP761J1410 | | AA | | 0.049 | | 8.104 | | | 1.006 | | 65.307 |
| ID_47813 | ZNF791 | | ES | | 0.015 | | 0.283 | | | 0.103 | | 0.782 |
| ID_47823 | WDR83 | | AD | | 0.019 | | 0.068 | | | 0.007 | | 0.641 |
| ID_47863 | HOOK2 | | AP | | 0.012 | | 0.052 | | | 0.005 | | 0.526 |
| ID_47883 | GCDH | | AA | | 0.010 | | 0.002 | | | 0.000 | | 0.225 |
| ID_47907 | NFIX | | ES | | 0.003 | | 0.051 | | | 0.007 | | 0.366 |
| ID_47967 | CD97 | | ES | | 0.002 | | 13.903 | | | 2.540 | | 76.112 |
| ID_47969 | CD97 | | ES | | 0.011 | | 0.154 | | | 0.037 | | 0.645 |
| ID_48045 | EMR2 | | ES | | 0.030 | | 3.552 | | | 1.130 | | 11.166 |
| ID_48140 | HSH2D | | ES | | 0.049 | | 0.129 | | | 0.017 | | 0.990 |
| ID_48281 | GTPBP3 | | AP | | 0.006 | | 0.034 | | | 0.003 | | 0.381 |
| ID_48368 | FCHO1 | | AA | | 0.050 | | 54.740 | | | 1.003 | | 2988.273 |
| ID_484887 | COL1A2 | | ES | | 0.026 | | 0.145 | | | 0.026 | | 0.796 |
| ID_48492 | C19orf60 | | AA | | 0.027 | | 0.042 | | | 0.003 | | 0.698 |
| ID_48504 | CRTC1 | | ES | | 0.019 | | 6.242 | | | 1.356 | | 28.728 |
| ID_48505 | COMP | | ES | | 0.024 | | 0.030 | | | 0.001 | | 0.630 |
| ID_485082 | COL3A1 | | ES | | 0.034 | | 0.151 | | | 0.026 | | 0.869 |
| ID_485096 | COL3A1 | | ES | | 0.031 | | 0.105 | | | 0.014 | | 0.811 |
| ID_48542 | SUGP2 | | AP | | 0.008 | | 6.759 | | | 1.659 | | 27.535 |
| ID_48642 | GATAD2A | | AA | | 0.031 | | 0.109 | | | 0.015 | | 0.812 |
| ID_48666 | LPAR2 | | ES | | 0.030 | | 3.042 | | | 1.112 | | 8.326 |
| ID_48693 | ZNF253 | | ES | | 0.017 | | 0.090 | | | 0.012 | | 0.654 |
| ID_48743 | ZNF430 | | AA | | 0.025 | | 5.383 | | | 1.236 | | 23.447 |
| ID_48798 | ZNF208 | | AT | | 0.032 | | 0.431 | | | 0.200 | | 0.931 |
| ID_48934 | SLC7A10 | | AT | | 0.018 | | 0.118 | | | 0.020 | | 0.696 |
| ID_48957 | GPI | | AP | | 0.041 | | 0.031 | | | 0.001 | | 0.865 |
| ID_49007 | ZNF792 | | AP | | 0.045 | | 0.222 | | | 0.051 | | 0.965 |
| ID_49017 | SCN1B | | AA | | 0.011 | | 0.001 | | | 0.000 | | 0.196 |
| ID_49112 | CD22 | | ES | | 0.036 | | 0.065 | | | 0.005 | | 0.841 |
| ID_49237 | ETV2 | | ES | | 0.048 | | 0.005 | | | 0.000 | | 0.944 |
| ID_49329 | CLIP3 | | AP | | 0.032 | | 3.844 | | | 1.125 | | 13.140 |
| ID_49610 | YIF1B | | AD | | 0.036 | | 0.269 | | | 0.079 | | 0.920 |
| ID_49676 | MAP4K1 | | ES | | 0.027 | | 10792320.977 | | | 6.386 | | 18238939541977.2 |
| ID_49697 | HNRNPL | | AP | | 0.047 | | 0.340 | | | 0.118 | | 0.986 |
| ID_49841 | DYRK1B | | AP | | 0.010 | | 38.689 | | | 2.406 | | 622.110 |
| ID_49966 | C19orf54 | | RI | | 0.001 | | 0.047 | | | 0.008 | | 0.285 |
| ID_49970 | C19orf54 | | RI | | 0.040 | | 0.002 | | | 0.000 | | 0.746 |
| ID_50020 | CYP2B6 | | ES | | 0.003 | | 0.166 | | | 0.050 | | 0.552 |
| ID_50046 | TMEM91 | | RI | | 0.046 | | 0.477 | | | 0.231 | | 0.986 |
| ID_50052 | TMEM91 | | RI | | 0.030 | | 0.457 | | | 0.225 | | 0.928 |
| ID_50215 | ZNF576 | | RI | | 0.009 | | 2.969 | | | 1.305 | | 6.757 |
| ID_50216 | ZNF576 | | AA | | 0.017 | | 15.473 | | | 1.631 | | 146.799 |
| ID_50230 | PLAUR | | AT | | 0.027 | | 43.813 | | | 1.534 | | 1251.444 |
| ID_50293 | ZNF226 | | ES | | 0.010 | | 14.516 | | | 1.907 | | 110.523 |
| ID_50306 | ZNF235 | | AT | | 0.030 | | 16.638 | | | 1.312 | | 211.068 |
| ID_50447 | ERCC1 | | AD | | 0.001 | | 0.082 | | | 0.018 | | 0.380 |
| ID_50498 | EML2 | | ES | | 0.004 | | 7.613 | | | 1.888 | | 30.708 |
| ID_50499 | EML2 | | ES | | 0.012 | | 410993.932 | | | 16.911 | | 9988246095.642 |
| ID_50598 | AP2S1 | | AP | | 0.028 | | 0.020 | | | 0.001 | | 0.663 |
| ID_50624 | SAE1 | | ES | | 0.014 | | 235091.974 | | | 12.060 | | 4582891556.410 |
| ID_50715 | CARD8 | | ES | | 0.030 | | 0.274 | | | 0.085 | | 0.880 |
| ID_50721 | CARD8 | | RI | | 0.017 | | 0.058 | | | 0.005 | | 0.605 |
| ID_50773 | SULT2B1 | | AP | | 0.007 | | 0.400 | | | 0.205 | | 0.780 |
| ID_50793 | SPHK2 | | AD | | 0.003 | | 5.243 | | | 1.744 | | 15.762 |
| ID_50800 | FUT2 | | AD | | 0.019 | | 16.870 | | | 1.581 | | 180.061 |
| ID_50818 | HSD17B14 | | ES | | 0.043 | | 10.613 | | | 1.083 | | 104.002 |
| ID_50884 | SNRNP70 | | AD | | 0.009 | | 0.113 | | | 0.022 | | 0.577 |
| ID_50925 | TEAD2 | | ES | | 0.014 | | 0.008 | | | 0.000 | | 0.378 |
| ID_50946 | FLT3LG | | AA | | 0.014 | | 0.108 | | | 0.018 | | 0.643 |
| ID_50958 | FCGRT | | AP | | 0.047 | | 0.215 | | | 0.047 | | 0.981 |
| ID_51080 | FUZ | | ES | | 0.006 | | 0.010 | | | 0.000 | | 0.273 |
| ID_51157 | ZNF473 | | AT | | 0.014 | | 0.003 | | | 0.000 | | 0.314 |
| ID_51203 | JOSD2 | | AP | | 0.040 | | 31.466 | | | 1.179 | | 840.126 |
| ID_51206 | JOSD2 | | AA | | 0.021 | | 0.034 | | | 0.002 | | 0.595 |
| ID_51474 | ZNF83 | | AP | | 0.035 | | 0.256 | | | 0.072 | | 0.910 |
| ID_51647 | ZNF816 | | ES | | 0.024 | | 3.434 | | | 1.175 | | 10.040 |
| ID_51714 | ZNF525 | | ME | | 0.044 | | 0.083 | | | 0.007 | | 0.936 |
| ID_51881 | LAIR1 | | AA | | 0.021 | | 0.108 | | | 0.016 | | 0.717 |
| ID_51918 | LILRB1 | | AP | | 0.022 | | 0.352 | | | 0.143 | | 0.863 |
| ID_52060 | BRSK1 | | AT | | 0.004 | | 0.024 | | | 0.002 | | 0.301 |
| ID_52343 | ZNF552 | | AD | | 0.007 | | 4.208 | | | 1.472 | | 12.030 |
| ID_52390 | ZNF135 | | AT | | 0.035 | | 0.278 | | | 0.084 | | 0.913 |
| ID_52417 | ZNF274 | | ES | | 0.025 | | 0.025 | | | 0.001 | | 0.635 |
| ID_52490 | MZF1 | | AD | | 0.040 | | 4.865 | | | 1.072 | | 22.075 |
| ID_52561 | TRAPPC12 | | AD | | 0.016 | | 0.011 | | | 0.000 | | 0.430 |
| ID_52594 | RSAD2 | | AP | | 0.038 | | 634.410 | | | 1.437 | | 280075.790 |
| ID_52678 | PQLC3 | | ES | | 0.000 | | 0.000 | | | 0.000 | | 0.026 |
| ID_52798 | MFSD2B | | AT | | 0.001 | | 0.023 | | | 0.003 | | 0.197 |
| ID_52940 | CGREF1 | | ES | | 0.048 | | 0.353 | | | 0.125 | | 0.993 |
| ID_53012 | KRTCAP3 | | AP | | 0.028 | | 29.248 | | | 1.440 | | 594.153 |
| ID_53056 | BRE | | ES | | 0.012 | | 0.059 | | | 0.007 | | 0.532 |
| ID_53180 | LTBP1 | | AP | | 0.001 | | 3.675 | | | 1.657 | | 8.150 |
| ID_53276 | SRSF7 | | RI | | 0.000 | | 0.141 | | | 0.047 | | 0.421 |
| ID_53277 | SRSF7 | | RI | | 0.041 | | 0.022 | | | 0.001 | | 0.861 |
| ID_53279 | SRSF7 | | RI | | 0.007 | | 0.233 | | | 0.081 | | 0.669 |
| ID_53362 | MTA3 | | AP | | 0.033 | | 0.282 | | | 0.088 | | 0.903 |
| ID_534726 | ANKRD36C | | ES | | 0.031 | | 0.242 | | | 0.067 | | 0.877 |
| ID_534737 | ANKRD36C | | ES | | 0.041 | | 3.030 | | | 1.044 | | 8.794 |
| ID_53615 | CCDC88A | | ES | | 0.049 | | 0.009 | | | 0.000 | | 0.979 |
| ID_53718 | EHBP1 | | ES | | 0.020 | | 0.205 | | | 0.053 | | 0.782 |
| ID_53725 | WDPCP | | AP | | 0.021 | | 0.235 | | | 0.068 | | 0.807 |
| ID_53763 | VPS54 | | ES | | 0.041 | | 0.033 | | | 0.001 | | 0.869 |
| ID_53773 | AFTPH | | ES | | 0.032 | | 0.078 | | | 0.008 | | 0.805 |
| ID_53838 | CNRIP1 | | RI | | 0.030 | | 0.166 | | | 0.033 | | 0.841 |
| ID_54060 | INO80B | | AT | | 0.039 | | 9.796 | | | 1.120 | | 85.661 |
| ID_54385 | ST3GAL5 | | AT | | 0.040 | | 14.331 | | | 1.128 | | 182.132 |
| ID_54771 | IL1R1 | | AP | | 0.001 | | 0.155 | | | 0.050 | | 0.482 |
| ID_54966 | BCL2L11 | | AD | | 0.020 | | 26.293 | | | 1.664 | | 415.561 |
| ID_55019 | POLR1B | | ES | | 0.007 | | 0.000 | | | 0.000 | | 0.102 |
| ID_55140 | PTPN4 | | AP | | 0.035 | | 0.271 | | | 0.081 | | 0.914 |
| ID_55200 | BIN1 | | ES | | 0.030 | | 3.610 | | | 1.132 | | 11.507 |
| ID_55249 | POLR2D | | ES | | 0.032 | | 153.398 | | | 1.559 | | 15097.943 |
| ID_55263 | UGGT1 | | AD | | 0.038 | | 2.821 | | | 1.061 | | 7.501 |
| ID_55264 | UGGT1 | | AD | | 0.044 | | 5.717 | | | 1.050 | | 31.118 |
| ID_55393 | LYPD1 | | AP | | 0.021 | | 0.229 | | | 0.065 | | 0.802 |
| ID_55450 | UBXN4 | | ES | | 0.046 | | 0.137 | | | 0.019 | | 0.968 |
| ID_55505 | GTDC1 | | ES | | 0.031 | | 0.051 | | | 0.003 | | 0.765 |
| ID_55538 | EPC2 | | AT | | 0.008 | | 0.017 | | | 0.001 | | 0.347 |
| ID_55608 | PRPF40A | | ES | | 0.027 | | 4.524 | | | 1.183 | | 17.305 |
| ID_55618 | NR4A2 | | AA | | 0.041 | | 9.028 | | | 1.096 | | 74.392 |
| ID_55643 | CYTIP | | AP | | 0.017 | | 243.247 | | | 2.690 | | 21995.460 |
| ID_55693 | WDSUB1 | | ES | | 0.001 | | 0.000 | | | 0.000 | | 0.039 |
| ID_55695 | WDSUB1 | | AD | | 0.027 | | 0.280 | | | 0.090 | | 0.864 |
| ID_55708 | LY75 | | AT | | 0.021 | | 0.347 | | | 0.141 | | 0.855 |
| ID_55723 | ITGB6 | | RI | | 0.017 | | 0.042 | | | 0.003 | | 0.563 |
| ID_55777 | FIGN | | AT | | 0.002 | | 0.008 | | | 0.000 | | 0.182 |
| ID_55864 | BBS5 | | AT | | 0.001 | | 0.003 | | | 0.000 | | 0.078 |
| ID_55893 | METTL5 | | RI | | 0.020 | | 5.580 | | | 1.306 | | 23.847 |
| ID_55932 | METTL8 | | ES | | 0.018 | | 2.398 | | | 1.162 | | 4.951 |
| ID_55943 | DYNC1I2 | | ES | | 0.038 | | 0.279 | | | 0.084 | | 0.934 |
| ID_56036 | CHRNA1 | | AT | | 0.023 | | 0.194 | | | 0.047 | | 0.802 |
| ID_56097 | KIAA1715 | | AP | | 0.029 | | 0.145 | | | 0.026 | | 0.823 |
| ID_56163 | PRKRA | | RI | | 0.043 | | 61.724 | | | 1.141 | | 3337.935 |
| ID_56415 | ITGA4 | | AT | | 0.040 | | 0.014 | | | 0.000 | | 0.826 |
| ID_56727 | TYW5 | | ES | | 0.016 | | 0.046 | | | 0.004 | | 0.562 |
| ID_56755 | CLK1 | | ES | | 0.002 | | 15.092 | | | 2.744 | | 82.998 |
| ID_56772 | NIF3L1 | | ES | | 0.016 | | 0.000 | | | 0.000 | | 0.177 |
| ID_56830 | CASP8 | | AA | | 0.040 | | 0.170 | | | 0.031 | | 0.919 |
| ID_56847 | TMEM237 | | AP | | 0.009 | | 3.440 | | | 1.355 | | 8.733 |
| ID_56955 | ICA1L | | AT | | 0.027 | | 7.873 | | | 1.270 | | 48.793 |
| ID_57009 | CYP20A1 | | ES | | 0.032 | | 0.031 | | | 0.001 | | 0.747 |
| ID_57170 | KLF7 | | ES | | 0.019 | | 0.016 | | | 0.000 | | 0.501 |
| ID_57181 | CREB1 | | ES | | 0.050 | | 0.172 | | | 0.029 | | 1.000 |
| ID_57192 | METTL21A | | AA | | 0.042 | | 4.532 | | | 1.055 | | 19.476 |
| ID_57431 | TNS1 | | AA | | 0.042 | | 0.040 | | | 0.002 | | 0.892 |
| ID_57454 | GPBAR1 | | AA | | 0.043 | | 0.337 | | | 0.117 | | 0.968 |
| ID_575 | CLSTN1 | | ES | | 0.003 | | 2.797 | | | 1.432 | | 5.464 |
| ID_57508 | ZNF142 | | ES | | 0.047 | | 2.812 | | | 1.015 | | 7.791 |
| ID_57510 | ZNF142 | | AA | | 0.025 | | 30.884 | | | 1.544 | | 617.853 |
| ID_57559 | TTLL4 | | AP | | 0.004 | | 416.551 | | | 6.718 | | 25830.251 |
| ID_576 | CLSTN1 | | ES | | 0.007 | | 2.993 | | | 1.356 | | 6.603 |
| ID_57673 | TUBA4A | | AP | | 0.015 | | 0.013 | | | 0.000 | | 0.436 |
| ID_57699 | SPEG | | AT | | 0.003 | | 5.141 | | | 1.749 | | 15.118 |
| ID_57793 | RHBDD1 | | ES | | 0.025 | | 0.290 | | | 0.099 | | 0.855 |
| ID_58109 | COL6A3 | | ES | | 0.032 | | 0.130 | | | 0.020 | | 0.841 |
| ID_58129 | LRRFIP1 | | AP | | 0.036 | | 0.212 | | | 0.049 | | 0.907 |
| ID_58143 | LRRFIP1 | | ES | | 0.038 | | 0.025 | | | 0.001 | | 0.813 |
| ID_58194 | UBE2F-SCLY | | AT | | 0.029 | | 0.018 | | | 0.000 | | 0.668 |
| ID_58198 | SCLY | | AT | | 0.010 | | 0.074 | | | 0.010 | | 0.543 |
| ID_58390 | STK25 | | AD | | 0.032 | | 6.717 | | | 1.178 | | 38.315 |
| ID_58425 | D2HGDH | | ES | | 0.006 | | 0.000 | | | 0.000 | | 0.076 |
| ID_58621 | SMOX | | ES | | 0.036 | | 45.756 | | | 1.281 | | 1634.352 |
| ID_58646 | TMEM230 | | AD | | 0.037 | | 0.080 | | | 0.007 | | 0.857 |
| ID_58666 | FERMT1 | | ES | | 0.037 | | 0.000 | | | 0.000 | | 0.575 |
| ID_587 | LZIC | | AP | | 0.012 | | 0.193 | | | 0.053 | | 0.700 |
| ID_58753 | MGME1 | | AD | | 0.015 | | 1670.910 | | | 4.316 | | 646912.551 |
| ID_58840 | GZF1 | | ES | | 0.000 | | 0.000 | | | 0.000 | | 0.015 |
| ID_58866 | ENTPD6 | | ES | | 0.004 | | 0.170 | | | 0.051 | | 0.562 |
| ID_58881 | FAM182B | | AT | | 0.009 | | 0.096 | | | 0.016 | | 0.561 |
| ID_590 | NMNAT1 | | AT | | 0.036 | | 0.269 | | | 0.079 | | 0.918 |
| ID_59014 | ZNF341 | | ES | | 0.024 | | 43.648 | | | 1.654 | | 1151.776 |
| ID_59206 | RBM12 | | ES | | 0.013 | | 0.133 | | | 0.027 | | 0.655 |
| ID_59223 | ROMO1 | | RI | | 0.009 | | 0.000 | | | 0.000 | | 0.147 |
| ID_59273 | EPB41L1 | | ES | | 0.026 | | 2.750 | | | 1.126 | | 6.716 |
| ID_59292 | C20orf24 | | ES | | 0.014 | | 82.253 | | | 2.468 | | 2741.476 |
| ID_59303 | NDRG3 | | ES | | 0.004 | | 65.934 | | | 3.868 | | 1123.972 |
| ID_59364 | VSTM2L | | ES | | 0.045 | | 0.004 | | | 0.000 | | 0.893 |
| ID_59383 | PPP1R16B | | ES | | 0.001 | | 0.000 | | | 0.000 | | 0.018 |
| ID_59424 | CHD6 | | AT | | 0.005 | | 0.001 | | | 0.000 | | 0.122 |
| ID_59446 | SGK2 | | AA | | 0.041 | | 0.041 | | | 0.002 | | 0.880 |
| ID_59500 | PABPC1L | | RI | | 0.032 | | 2064709832.75327 | | | 6.223 | | 685045028387213000 |
| ID_59728 | SULF2 | | ES | | 0.010 | | 4.007 | | | 1.399 | | 11.476 |
| ID_60075 | RPS21 | | AA | | 0.010 | | 0.286 | | | 0.110 | | 0.743 |
| ID_60092 | DIDO1 | | AT | | 0.025 | | 0.305 | | | 0.108 | | 0.861 |
| ID_60095 | DIDO1 | | AD | | 0.033 | | 0.581 | | | 0.353 | | 0.958 |
| ID_60135 | HELZ2 | | AP | | 0.036 | | 0.064 | | | 0.005 | | 0.837 |
| ID_60143 | RTEL1 | | AP | | 0.022 | | 2.221 | | | 1.123 | | 4.391 |
| ID_60164 | ZGPAT | | AD | | 0.042 | | 0.110 | | | 0.013 | | 0.919 |
| ID_602 | KIF1B | | AT | | 0.001 | | 0.104 | | | 0.026 | | 0.413 |
| ID_60266 | ATP5J | | AD | | 0.038 | | 0.011 | | | 0.000 | | 0.772 |
| ID_60283 | APP | | ES | | 0.009 | | 5.921 | | | 1.559 | | 22.482 |
| ID_60438 | SON | | ES | | 0.046 | | 14.857 | | | 1.051 | | 209.993 |
| ID_60695 | ZBTB21 | | ES | | 0.022 | | 2.772 | | | 1.160 | | 6.623 |
| ID_60707 | TMPRSS3 | | AT | | 0.041 | | 0.233 | | | 0.058 | | 0.943 |
| ID_60712 | UBASH3A | | AT | | 0.024 | | 0.155 | | | 0.031 | | 0.786 |
| ID_60883 | SLC19A1 | | AA | | 0.009 | | 0.014 | | | 0.001 | | 0.347 |
| ID_60898 | COL6A2 | | AA | | 0.018 | | 0.002 | | | 0.000 | | 0.359 |
| ID_60988 | BCL2L13 | | ES | | 0.038 | | 0.008 | | | 0.000 | | 0.764 |
| ID_61047 | HIRA | | AP | | 0.030 | | 0.337 | | | 0.126 | | 0.902 |
| ID_61158 | SCARF2 | | AA | | 0.040 | | 0.164 | | | 0.029 | | 0.923 |
| ID_61211 | THAP7 | | RI | | 0.030 | | 0.230 | | | 0.061 | | 0.869 |
| ID_61347 | SLC2A11 | | ES | | 0.007 | | 0.267 | | | 0.103 | | 0.693 |
| ID_61393 | GGT5 | | AA | | 0.022 | | 0.008 | | | 0.000 | | 0.500 |
| ID_61407 | GUCD1 | | AP | | 0.035 | | 8.048 | | | 1.158 | | 55.906 |
| ID_61432 | GGT1 | | RI | | 0.004 | | 52.546 | | | 3.609 | | 764.981 |
| ID_61468 | KIAA1671 | | AP | | 0.042 | | 2.083 | | | 1.027 | | 4.224 |
| ID_61613 | THOC5 | | ES | | 0.023 | | 2.640 | | | 1.146 | | 6.079 |
| ID_61690 | MTMR3 | | ES | | 0.015 | | 3.279 | | | 1.254 | | 8.573 |
| ID_61691 | MTMR3 | | ES | | 0.029 | | 0.109 | | | 0.015 | | 0.800 |
| ID_61722 | TBC1D10A | | AA | | 0.025 | | 224.843 | | | 1.993 | | 25371.499 |
| ID_61779 | PES1 | | AP | | 0.001 | | 0.001 | | | 0.000 | | 0.076 |
| ID_61796 | DUSP18 | | RI | | 0.000 | | 0.002 | | | 0.000 | | 0.053 |
| ID_61896 | DEPDC5 | | AT | | 0.021 | | 0.098 | | | 0.014 | | 0.702 |
| ID_61936 | FBXO7 | | AA | | 0.036 | | 0.015 | | | 0.000 | | 0.756 |
| ID_61952 | TOM1 | | AD | | 0.043 | | 0.031 | | | 0.001 | | 0.899 |
| ID_62023 | APOL1 | | ES | | 0.035 | | 0.038 | | | 0.002 | | 0.794 |
| ID_62141 | SH3BP1 | | ES | | 0.013 | | 0.044 | | | 0.004 | | 0.512 |
| ID_62286 | CBX7 | | AA | | 0.000 | | 147.779 | | | 9.388 | | 2326.288 |
| ID_62518 | SERHL2 | | ES | | 0.009 | | 0.019 | | | 0.001 | | 0.377 |
| ID_62559 | PACSIN2 | | ES | | 0.003 | | 26.877 | | | 3.162 | | 228.479 |
| ID_62618 | PRR5 | | AA | | 0.011 | | 0.016 | | | 0.001 | | 0.387 |
| ID_62646 | KIAA0930 | | AP | | 0.013 | | 0.043 | | | 0.004 | | 0.518 |
| ID_62657 | FAM118A | | AP | | 0.035 | | 2.250 | | | 1.057 | | 4.789 |
| ID_62753 | BRD1 | | AD | | 0.022 | | 1671.200 | | | 2.866 | | 974585.459 |
| ID_62828 | SBF1 | | ES | | 0.002 | | 4.139 | | | 1.708 | | 10.028 |
| ID_62916 | RABL2B | | AA | | 0.027 | | 19.924 | | | 1.411 | | 281.251 |
| ID_63092 | SETD5 | | ES | | 0.015 | | 4.550 | | | 1.345 | | 15.396 |
| ID_63155 | BRPF1 | | ES | | 0.041 | | 27.035 | | | 1.148 | | 636.502 |
| ID_63160 | OGG1 | | RI | | 0.046 | | 7.282 | | | 1.033 | | 51.340 |
| ID_63171 | OGG1 | | ES | | 0.010 | | 0.142 | | | 0.032 | | 0.628 |
| ID_63213 | TTLL3 | | AD | | 0.033 | | 28.990 | | | 1.305 | | 644.128 |
| ID_63262 | IL17RC | | ES | | 0.020 | | 7.772 | | | 1.377 | | 43.865 |
| ID_63395 | VGLL4 | | AP | | 0.018 | | 3.183 | | | 1.219 | | 8.315 |
| ID_63425 | PPARG | | ES | | 0.006 | | 0.108 | | | 0.022 | | 0.527 |
| ID_63433 | TSEN2 | | RI | | 0.008 | | 0.000 | | | 0.000 | | 0.138 |
| ID_63503 | HDAC11 | | ES | | 0.014 | | 0.000 | | | 0.000 | | 0.075 |
| ID_63526 | SLC6A6 | | AT | | 0.013 | | 0.026 | | | 0.002 | | 0.458 |
| ID_63587 | HACL1 | | ES | | 0.033 | | 166.909 | | | 1.502 | | 18547.674 |
| ID_63588 | HACL1 | | ES | | 0.019 | | 0.006 | | | 0.000 | | 0.436 |
| ID_63624 | ANKRD28 | | AP | | 0.020 | | 0.327 | | | 0.127 | | 0.842 |
| ID_63704 | SGOL1 | | AD | | 0.036 | | 0.000 | | | 0.000 | | 0.613 |
| ID_63780 | SLC4A7 | | ES | | 0.049 | | 0.163 | | | 0.027 | | 0.992 |
| ID_63839 | GLB1 | | ES | | 0.021 | | 0.005 | | | 0.000 | | 0.444 |
| ID_63872 | CLASP2 | | AP | | 0.025 | | 0.173 | | | 0.037 | | 0.803 |
| ID_63960 | LRRFIP2 | | ES | | 0.012 | | 2.231 | | | 1.189 | | 4.185 |
| ID_63982 | GOLGA4 | | ES | | 0.015 | | 0.307 | | | 0.119 | | 0.795 |
| ID_63993 | C3orf35 | | AT | | 0.002 | | 0.076 | | | 0.015 | | 0.388 |
| ID_64008 | PLCD1 | | AP | | 0.001 | | 0.299 | | | 0.143 | | 0.629 |
| ID_64113 | SCN11A | | AT | | 0.018 | | 0.158 | | | 0.034 | | 0.726 |
| ID_64183 | RPSA | | ES | | 0.032 | | 0.378 | | | 0.155 | | 0.922 |
| ID_64223 | ZNF619 | | AP | | 0.019 | | 0.133 | | | 0.025 | | 0.714 |
| ID_64269 | TRAK1 | | AT | | 0.025 | | 4.809 | | | 1.215 | | 19.029 |
| ID_64285 | VIPR1 | | ES | | 0.002 | | 0.074 | | | 0.014 | | 0.381 |
| ID_64426 | CXCR6 | | AP | | 0.002 | | 0.278 | | | 0.124 | | 0.620 |
| ID_64462 | ALS2CL | | RI | | 0.004 | | 4.835 | | | 1.670 | | 13.994 |
| ID_64463 | ALS2CL | | RI | | 0.028 | | 2.732 | | | 1.115 | | 6.695 |
| ID_64664 | ATRIP | | AD | | 0.013 | | 0.023 | | | 0.001 | | 0.447 |
| ID_64682 | TREX1 | | RI | | 0.002 | | 7.472 | | | 2.069 | | 26.992 |
| ID_64708 | PFKFB4 | | AD | | 0.045 | | 0.000 | | | 0.000 | | 0.817 |
| ID_64728 | SLC26A6 | | AA | | 0.032 | | 27.454 | | | 1.327 | | 568.028 |
| ID_64866 | AMT | | ME | | 0.016 | | 738.714 | | | 3.413 | | 159892.652 |
| ID_64990 | NAT6 | | AA | | 0.006 | | 8.142 | | | 1.799 | | 36.856 |
| ID_65099 | TEX264 | | AD | | 0.026 | | 0.210 | | | 0.053 | | 0.827 |
| ID_65103 | TEX264 | | ES | | 0.002 | | 0.176 | | | 0.059 | | 0.522 |
| ID_65108 | TEX264 | | AD | | 0.015 | | 0.017 | | | 0.001 | | 0.452 |
| ID_65237 | PBRM1 | | ES | | 0.029 | | 0.400 | | | 0.176 | | 0.909 |
| ID_65356 | FAM208A | | ES | | 0.034 | | 0.004 | | | 0.000 | | 0.663 |
| ID_65394 | SLMAP | | ES | | 0.012 | | 2.652 | | | 1.234 | | 5.698 |
| ID_65397 | SLMAP | | ES | | 0.026 | | 0.004 | | | 0.000 | | 0.525 |
| ID_65499 | CADPS | | AT | | 0.001 | | 0.093 | | | 0.022 | | 0.390 |
| ID_65516 | ATXN7 | | AP | | 0.029 | | 0.400 | | | 0.175 | | 0.912 |
| ID_65582 | FRMD4B | | AP | | 0.011 | | 0.426 | | | 0.221 | | 0.821 |
| ID_65609 | EIF4E3 | | AP | | 0.049 | | 0.396 | | | 0.158 | | 0.996 |
| ID_65817 | TBC1D23 | | ES | | 0.030 | | 2.203 | | | 1.081 | | 4.490 |
| ID_66001 | CBLB | | ES | | 0.005 | | 15.222 | | | 2.323 | | 99.762 |
| ID_66011 | BBX | | ES | | 0.046 | | 41.356 | | | 1.072 | | 1595.042 |
| ID_66013 | CD47 | | ES | | 0.003 | | 0.283 | | | 0.123 | | 0.654 |
| ID_66014 | CD47 | | ES | | 0.001 | | 0.032 | | | 0.004 | | 0.246 |
| ID_66023 | HHLA2 | | ES | | 0.026 | | 0.027 | | | 0.001 | | 0.655 |
| ID_66135 | C3orf17 | | AT | | 0.019 | | 344.133 | | | 2.615 | | 45290.114 |
| ID_66341 | PLA1A | | ES | | 0.003 | | 946.077 | | | 9.655 | | 92708.387 |
| ID_66531 | UMPS | | AD | | 0.001 | | 6631.557 | | | 41.582 | | 1057611.473 |
| ID_66555 | ZNF148 | | ES | | 0.008 | | 539.444 | | | 5.028 | | 57872.533 |
| ID_66615 | ABTB1 | | AP | | 0.050 | | 0.206 | | | 0.042 | | 0.998 |
| ID_66674 | ACAD9 | | ES | | 0.022 | | 0.022 | | | 0.001 | | 0.583 |
| ID_66685 | ACAD9 | | AD | | 0.033 | | 5.533 | | | 1.144 | | 26.769 |
| ID_66696 | RAB43 | | RI | | 0.014 | | 0.000 | | | 0.000 | | 0.116 |
| ID_66720 | MBD4 | | AD | | 0.012 | | 0.030 | | | 0.002 | | 0.459 |
| ID_66763 | ATP2C1 | | ES | | 0.007 | | 85.555 | | | 3.316 | | 2207.369 |
| ID_66786 | NEK11 | | ES | | 0.024 | | 0.136 | | | 0.024 | | 0.773 |
| ID_66871 | ANAPC13 | | AD | | 0.009 | | 0.008 | | | 0.000 | | 0.309 |
| ID_67091 | TFDP2 | | ES | | 0.031 | | 0.000 | | | 0.000 | | 0.286 |
| ID_67218 | TM4SF18 | | AP | | 0.020 | | 6.254 | | | 1.331 | | 29.379 |
| ID_67311 | MBNL1 | | ES | | 0.011 | | 0.053 | | | 0.006 | | 0.504 |
| ID_67347 | PLCH1 | | AT | | 0.042 | | 0.183 | | | 0.036 | | 0.943 |
| ID_67367 | TIPARP | | AP | | 0.003 | | 0.086 | | | 0.017 | | 0.425 |
| ID_67461 | IQCJ-SCHIP1 | | AT | | 0.022 | | 0.002 | | | 0.000 | | 0.399 |
| ID_67616 | CLDN11 | | AT | | 0.005 | | 2.452 | | | 1.310 | | 4.587 |
| ID_67658 | ECT2 | | AA | | 0.019 | | 0.001 | | | 0.000 | | 0.300 |
| ID_67732 | TTC14 | | RI | | 0.010 | | 85.276 | | | 2.921 | | 2489.912 |
| ID_67931 | FAM131A | | RI | | 0.034 | | 5.530 | | | 1.138 | | 26.862 |
| ID_67936 | FAM131A | | ES | | 0.042 | | 2.273 | | | 1.030 | | 5.018 |
| ID_68126 | CCDC50 | | ES | | 0.005 | | 0.357 | | | 0.175 | | 0.727 |
| ID_68181 | APOD | | ES | | 0.013 | | 0.176 | | | 0.045 | | 0.699 |
| ID_68187 | MUC20 | | RI | | 0.011 | | 8.970 | | | 1.646 | | 48.894 |
| ID_68274 | MFI2 | | AT | | 0.031 | | 0.297 | | | 0.099 | | 0.894 |
| ID_68295 | DLG1 | | ES | | 0.042 | | 3.883 | | | 1.051 | | 14.352 |
| ID_68305 | KIAA0226 | | AT | | 0.040 | | 0.026 | | | 0.001 | | 0.848 |
| ID_68341 | LMLN | | AD | | 0.045 | | 22.219 | | | 1.077 | | 458.317 |
| ID_68359 | PIGG | | AD | | 0.015 | | 0.095 | | | 0.014 | | 0.637 |
| ID_68391 | MFSD7 | | AP | | 0.019 | | 0.028 | | | 0.001 | | 0.560 |
| ID_68431 | TMEM175 | | ES | | 0.025 | | 0.218 | | | 0.057 | | 0.825 |
| ID_68487 | UVSSA | | AP | | 0.010 | | 0.170 | | | 0.045 | | 0.651 |
| ID_68530 | WHSC1 | | AD | | 0.010 | | 0.000 | | | 0.000 | | 0.089 |
| ID_68543 | NELFA | | ES | | 0.050 | | 15.384 | | | 1.006 | | 235.342 |
| ID_68549 | HAUS3 | | ES | | 0.028 | | 0.013 | | | 0.000 | | 0.622 |
| ID_68559 | ZFYVE28 | | AT | | 0.001 | | 0.152 | | | 0.051 | | 0.453 |
| ID_68808 | CC2D2A | | ES | | 0.026 | | 0.029 | | | 0.001 | | 0.650 |
| ID_68809 | CC2D2A | | ES | | 0.022 | | 2.208 | | | 1.119 | | 4.355 |
| ID_68814 | FBXL5 | | AA | | 0.012 | | 875.551 | | | 4.316 | | 177608.256 |
| ID_68881 | PACRGL | | ES | | 0.004 | | 2.519 | | | 1.345 | | 4.718 |
| ID_68883 | PACRGL | | ES | | 0.010 | | 0.258 | | | 0.091 | | 0.727 |
| ID_69017 | TBC1D1 | | ES | | 0.034 | | 4.273 | | | 1.117 | | 16.353 |
| ID_69021 | KLF3 | | AT | | 0.032 | | 0.132 | | | 0.021 | | 0.844 |
| ID_69148 | GUF1 | | ES | | 0.031 | | 0.166 | | | 0.033 | | 0.848 |
| ID_69335 | EXOC1 | | ES | | 0.016 | | 0.390 | | | 0.181 | | 0.838 |
| ID_69345 | AASDH | | ES | | 0.041 | | 0.066 | | | 0.005 | | 0.891 |
| ID_69445 | RUFY3 | | AP | | 0.022 | | 2.988 | | | 1.173 | | 7.611 |
| ID_69460 | DCK | | AD | | 0.041 | | 71.413 | | | 1.189 | | 4289.821 |
| ID_69533 | THAP6 | | ES | | 0.017 | | 126.191 | | | 2.399 | | 6637.686 |
| ID_69616 | SEPT11 | | AT | | 0.011 | | 0.064 | | | 0.008 | | 0.533 |
| ID_69660 | PAQR3 | | ME | | 0.034 | | 0.004 | | | 0.000 | | 0.654 |
| ID_69718 | SEC31A | | AP | | 0.042 | | 3.126 | | | 1.043 | | 9.372 |
| ID_69730 | SEC31A | | ES | | 0.007 | | 0.434 | | | 0.236 | | 0.798 |
| ID_69731 | SEC31A | | ES | | 0.004 | | 0.167 | | | 0.049 | | 0.565 |
| ID_69750 | LIN54 | | AP | | 0.031 | | 2.539 | | | 1.088 | | 5.923 |
| ID_69811 | WDFY3 | | ES | | 0.040 | | 2.223 | | | 1.036 | | 4.766 |
| ID_69812 | WDFY3 | | ES | | 0.007 | | 0.337 | | | 0.152 | | 0.748 |
| ID_69831 | PTPN13 | | AT | | 0.014 | | 0.003 | | | 0.000 | | 0.304 |
| ID_69991 | UNC5C | | AT | | 0.015 | | 0.099 | | | 0.015 | | 0.639 |
| ID_70003 | TSPAN5 | | AP | | 0.021 | | 0.017 | | | 0.001 | | 0.540 |
| ID_70171 | SLC9B2 | | AT | | 0.036 | | 34.801 | | | 1.256 | | 964.392 |
| ID_70175 | SLC9B2 | | ES | | 0.021 | | 0.167 | | | 0.036 | | 0.764 |
| ID_70187 | TET2 | | AT | | 0.000 | | 0.015 | | | 0.001 | | 0.154 |
| ID_70292 | LEF1 | | ES | | 0.037 | | 9.344 | | | 1.144 | | 76.345 |
| ID_70553 | MFSD8 | | ES | | 0.022 | | 25.053 | | | 1.581 | | 396.943 |
| ID_70601 | PCDH18 | | RI | | 0.004 | | 0.001 | | | 0.000 | | 0.127 |
| ID_70649 | SCOC | | ES | | 0.045 | | 0.125 | | | 0.016 | | 0.952 |
| ID_70697 | USP38 | | AT | | 0.009 | | 0.013 | | | 0.001 | | 0.339 |
| ID_70700 | GAB1 | | ES | | 0.040 | | 3.142 | | | 1.052 | | 9.381 |
| ID_70799 | ARHGAP10 | | ES | | 0.012 | | 8.327 | | | 1.605 | | 43.207 |
| ID_70810 | LRBA | | AP | | 0.032 | | 2.528 | | | 1.082 | | 5.908 |
| ID_70846 | FBXW7 | | AP | | 0.017 | | 0.201 | | | 0.054 | | 0.753 |
| ID_71001 | RAPGEF2 | | ES | | 0.016 | | 3.616 | | | 1.271 | | 10.285 |
| ID_71173 | GALNT7 | | AA | | 0.010 | | 6.337 | | | 1.559 | | 25.759 |
| ID_71228 | NEIL3 | | ES | | 0.005 | | 0.011 | | | 0.000 | | 0.261 |
| ID_71339 | LRP2BP | | RI | | 0.037 | | 3.436 | | | 1.075 | | 10.977 |
| ID_71348 | UFSP2 | | AD | | 0.005 | | 0.001 | | | 0.000 | | 0.135 |
| ID_71387 | SORBS2 | | ES | | 0.004 | | 0.415 | | | 0.226 | | 0.761 |
| ID_71390 | SORBS2 | | ES | | 0.005 | | 0.433 | | | 0.242 | | 0.774 |
| ID_71515 | ADAMTS16 | | AT | | 0.017 | | 0.330 | | | 0.133 | | 0.821 |
| ID_71633 | C5orf22 | | ES | | 0.005 | | 7.991 | | | 1.870 | | 34.141 |
| ID_71734 | RAD1 | | AP | | 0.018 | | 5.695 | | | 1.354 | | 23.957 |
| ID_71774 | IL7R | | AT | | 0.006 | | 0.023 | | | 0.002 | | 0.342 |
| ID_71837 | EGFLAM | | AP | | 0.004 | | 2.152 | | | 1.279 | | 3.623 |
| ID_71881 | OXCT1 | | AP | | 0.032 | | 0.188 | | | 0.041 | | 0.864 |
| ID_71952 | C5orf28 | | ES | | 0.027 | | 8.063 | | | 1.273 | | 51.064 |
| ID_71983 | PARP8 | | ES | | 0.047 | | 0.298 | | | 0.091 | | 0.982 |
| ID_72018 | SNX18 | | AD | | 0.045 | | 92.194 | | | 1.115 | | 7625.790 |
| ID_72019 | ESM1 | | ES | | 0.031 | | 0.053 | | | 0.004 | | 0.760 |
| ID_72172 | NDUFAF2 | | AD | | 0.032 | | 24.672 | | | 1.316 | | 462.570 |
| ID_72193 | IPO11 | | ES | | 0.023 | | 4.271 | | | 1.218 | | 14.975 |
| ID_72217 | CENPK | | ES | | 0.029 | | 0.013 | | | 0.000 | | 0.635 |
| ID_72245 | TRAPPC13 | | ES | | 0.020 | | 3.601 | | | 1.225 | | 10.584 |
| ID_723 | PRDM2 | | ES | | 0.034 | | 3.783 | | | 1.106 | | 12.936 |
| ID_72341 | CCDC125 | | AA | | 0.049 | | 4.141 | | | 1.004 | | 17.071 |
| ID_72409 | SMN2 | | ES | | 0.005 | | 0.088 | | | 0.016 | | 0.477 |
| ID_72654 | FAM151B | | AT | | 0.006 | | 0.000 | | | 0.000 | | 0.050 |
| ID_72786 | FAM172A | | ES | | 0.050 | | 0.070 | | | 0.005 | | 0.999 |
| ID_7279 | RBM8A | | AA | | 0.018 | | 0.000 | | | 0.000 | | 0.249 |
| ID_72822 | RHOBTB3 | | AP | | 0.019 | | 0.020 | | | 0.001 | | 0.519 |
| ID_72838 | CAST | | AP | | 0.025 | | 0.324 | | | 0.121 | | 0.868 |
| ID_72858 | CAST | | ES | | 0.027 | | 30.821 | | | 1.464 | | 648.874 |
| ID_72865 | ERAP1 | | AT | | 0.009 | | 0.213 | | | 0.067 | | 0.676 |
| ID_72873 | ERAP2 | | ES | | 0.028 | | 1531.591 | | | 2.194 | | 1069114.572 |
| ID_72880 | RGMB | | AP | | 0.029 | | 0.298 | | | 0.100 | | 0.885 |
| ID_72914 | PPIP5K2 | | ES | | 0.007 | | 3.769 | | | 1.439 | | 9.870 |
| ID_72917 | PPIP5K2 | | ES | | 0.020 | | 3.225 | | | 1.202 | | 8.653 |
| ID_72985 | APC | | AP | | 0.001 | | 0.071 | | | 0.015 | | 0.331 |
| ID_73008 | YTHDC2 | | AT | | 0.025 | | 0.000 | | | 0.000 | | 0.201 |
| ID_73050 | COMMD10 | | AP | | 0.027 | | 27315.487 | | | 3.170 | | 235380387.099 |
| ID_73166 | GRAMD3 | | ES | | 0.047 | | 7.103 | | | 1.030 | | 48.982 |
| ID_73237 | FNIP1 | | ES | | 0.014 | | 0.346 | | | 0.149 | | 0.808 |
| ID_73263 | P4HA2 | | ES | | 0.001 | | 0.213 | | | 0.089 | | 0.513 |
| ID_7328 | NBPF11 | | ES | | 0.020 | | 6.379 | | | 1.334 | | 30.511 |
| ID_73291 | RAD50 | | AD | | 0.034 | | 0.007 | | | 0.000 | | 0.678 |
| ID_73687 | CD14 | | RI | | 0.040 | | 0.175 | | | 0.033 | | 0.923 |
| ID_73776 | PCDHA5 | | AT | | 0.006 | | 9.093 | | | 1.890 | | 43.749 |
| ID_7384 | ACP6 | | AT | | 0.033 | | 3.325 | | | 1.105 | | 10.010 |
| ID_74 | ACAP3 | | ES | | 0.022 | | 25.793 | | | 1.592 | | 417.777 |
| ID_74053 | TIGD6 | | AD | | 0.022 | | 4.558 | | | 1.246 | | 16.679 |
| ID_7411 | SV2A | | AT | | 0.036 | | 2.562 | | | 1.064 | | 6.170 |
| ID_74125 | GPX3 | | ES | | 0.007 | | 0.015 | | | 0.001 | | 0.317 |
| ID_74403 | RNF145 | | AP | | 0.034 | | 0.046 | | | 0.003 | | 0.788 |
| ID_74410 | PWWP2A | | ES | | 0.028 | | 3.293 | | | 1.136 | | 9.546 |
| ID_74475 | SPDL1 | | ES | | 0.028 | | 0.065 | | | 0.006 | | 0.749 |
| ID_74502 | GABRP | | AT | | 0.000 | | 0.000 | | | 0.000 | | 0.010 |
| ID_74503 | GABRP | | ES | | 0.037 | | 0.229 | | | 0.057 | | 0.913 |
| ID_74570 | RPL26L1 | | AD | | 0.011 | | 0.003 | | | 0.000 | | 0.261 |
| ID_74616 | THOC3 | | AP | | 0.006 | | 0.000 | | | 0.000 | | 0.044 |
| ID_74741 | NSD1 | | AT | | 0.004 | | 0.006 | | | 0.000 | | 0.187 |
| ID_74812 | FAM153A | | AT | | 0.041 | | 0.251 | | | 0.067 | | 0.946 |
| ID_7515 | ARNT | | ES | | 0.000 | | 4743.087 | | | 40.950 | | 549372.834 |
| ID_75193 | SLC22A23 | | AT | | 0.012 | | 0.015 | | | 0.001 | | 0.396 |
| ID_75251 | RREB1 | | ES | | 0.035 | | 389.000 | | | 1.543 | | 98061.727 |
| ID_75253 | RREB1 | | ES | | 0.044 | | 3.151 | | | 1.031 | | 9.630 |
| ID_75397 | SIRT5 | | ES | | 0.019 | | 3.779 | | | 1.246 | | 11.459 |
| ID_75436 | CAP2 | | ES | | 0.040 | | 0.076 | | | 0.006 | | 0.894 |
| ID_75510 | MRS2 | | ES | | 0.041 | | 0.011 | | | 0.000 | | 0.827 |
| ID_75571 | HFE | | AA | | 0.044 | | 81.604 | | | 1.134 | | 5873.264 |
| ID_75777 | C6orf1 | | AD | | 0.016 | | 0.008 | | | 0.000 | | 0.407 |
| ID_758 | CASP9 | | AP | | 0.032 | | 0.141 | | | 0.024 | | 0.843 |
| ID_7581 | PIP5K1A | | AD | | 0.034 | | 0.243 | | | 0.066 | | 0.901 |
| ID_75982 | KCTD20 | | ES | | 0.036 | | 49.917 | | | 1.288 | | 1935.038 |
| ID_75985 | SRSF3 | | ES | | 0.001 | | 10.621 | | | 2.684 | | 42.028 |
| ID_76094 | NFYA | | ES | | 0.041 | | 2.227 | | | 1.032 | | 4.806 |
| ID_76108 | TREM1 | | AT | | 0.018 | | 0.094 | | | 0.013 | | 0.664 |
| ID_76115 | MDFI | | AP | | 0.038 | | 2.063 | | | 1.040 | | 4.093 |
| ID_7613 | RFX5 | | AD | | 0.003 | | 55.222 | | | 3.837 | | 794.720 |
| ID_76133 | PRICKLE4 | | AP | | 0.017 | | 0.293 | | | 0.106 | | 0.806 |
| ID_76156 | CCND3 | | AP | | 0.023 | | 3.209 | | | 1.173 | | 8.781 |
| ID_76167 | TAF8 | | AT | | 0.046 | | 0.212 | | | 0.046 | | 0.972 |
| ID_76200 | PPP2R5D | | ES | | 0.000 | | 0.000 | | | 0.000 | | 0.031 |
| ID_76203 | PPP2R5D | | AA | | 0.032 | | 0.000 | | | 0.000 | | 0.232 |
| ID_7636 | TUFT1 | | ES | | 0.039 | | 2.307 | | | 1.042 | | 5.108 |
| ID_76404 | RUNX2 | | ES | | 0.041 | | 0.310 | | | 0.101 | | 0.954 |
| ID_76560 | DST | | ES | | 0.033 | | 5.778 | | | 1.153 | | 28.960 |
| ID_76593 | RAB23 | | AP | | 0.004 | | 0.032 | | | 0.003 | | 0.330 |
| ID_76614 | EYS | | AT | | 0.007 | | 0.092 | | | 0.016 | | 0.518 |
| ID_767 | PLEKHM2 | | ES | | 0.029 | | 3.231 | | | 1.128 | | 9.254 |
| ID_76787 | FILIP1 | | AP | | 0.044 | | 0.148 | | | 0.023 | | 0.950 |
| ID_76829 | BCKDHB | | ES | | 0.005 | | 606.924 | | | 6.836 | | 53881.229 |
| ID_769 | FBLIM1 | | AP | | 0.022 | | 2.158 | | | 1.118 | | 4.168 |
| ID_76949 | SMIM8 | | ES | | 0.023 | | 116.595 | | | 1.931 | | 7041.176 |
| ID_77020 | MAP3K7 | | ES | | 0.006 | | 0.367 | | | 0.179 | | 0.754 |
| ID_77046 | MMS22L | | AD | | 0.010 | | 93.917 | | | 2.965 | | 2974.666 |
| ID_77092 | ASCC3 | | AT | | 0.049 | | 34.790 | | | 1.020 | | 1186.532 |
| ID_77153 | ARMC2 | | ES | | 0.002 | | 0.019 | | | 0.002 | | 0.241 |
| ID_7721 | S100A2 | | ES | | 0.026 | | 15.443 | | | 1.389 | | 171.631 |
| ID_77265 | TRAF3IP2 | | AP | | 0.014 | | 0.015 | | | 0.001 | | 0.421 |
| ID_77275 | FYN | | ES | | 0.023 | | 54.797 | | | 1.728 | | 1737.991 |
| ID_77318 | FAM26F | | ES | | 0.031 | | 0.181 | | | 0.038 | | 0.854 |
| ID_77390 | SMPDL3A | | ES | | 0.016 | | 0.072 | | | 0.008 | | 0.618 |
| ID_77424 | HDDC2 | | ES | | 0.002 | | 0.037 | | | 0.005 | | 0.304 |
| ID_77429 | NCOA7 | | AA | | 0.006 | | 0.146 | | | 0.037 | | 0.570 |
| ID_77830 | MYB | | AD | | 0.002 | | 363.077 | | | 8.820 | | 14946.039 |
| ID_77870 | MYB | | ES | | 0.025 | | 57.665 | | | 1.671 | | 1990.049 |
| ID_77877 | MYB | | ES | | 0.018 | | 0.000 | | | 0.000 | | 0.252 |
| ID_77894 | AHI1 | | ES | | 0.018 | | 0.350 | | | 0.146 | | 0.838 |
| ID_77956 | REPS1 | | ES | | 0.019 | | 0.236 | | | 0.070 | | 0.789 |
| ID_77983 | PHACTR2 | | AP | | 0.025 | | 2.481 | | | 1.120 | | 5.499 |
| ID_78023 | PLAGL1 | | ES | | 0.012 | | 4.951 | | | 1.428 | | 17.169 |
| ID_78060 | SASH1 | | AP | | 0.025 | | 0.251 | | | 0.075 | | 0.839 |
| ID_78157 | RMND1 | | AP | | 0.016 | | 0.032 | | | 0.002 | | 0.521 |
| ID_78184 | SYNE1 | | ES | | 0.032 | | 2.984 | | | 1.101 | | 8.092 |
| ID_7828 | ATP8B2 | | AP | | 0.025 | | 3.905 | | | 1.183 | | 12.893 |
| ID_78456 | MLLT4 | | ES | | 0.003 | | 2.929 | | | 1.443 | | 5.947 |
| ID_7855 | SHC1 | | AP | | 0.009 | | 3.823 | | | 1.389 | | 10.517 |
| ID_78606 | NUDT1 | | AP | | 0.022 | | 8.670 | | | 1.371 | | 54.808 |
| ID_78612 | EIF3B | | AA | | 0.028 | | 0.042 | | | 0.003 | | 0.705 |
| ID_78732 | DAGLB | | ES | | 0.018 | | 18984.813 | | | 5.268 | | 68421672.673 |
| ID_78748 | ZDHHC4 | | AD | | 0.022 | | 0.363 | | | 0.153 | | 0.863 |
| ID_78774 | MIOS | | ES | | 0.034 | | 3.206 | | | 1.092 | | 9.412 |
| ID_78868 | BZW2 | | AD | | 0.019 | | 12.725 | | | 1.519 | | 106.589 |
| ID_79078 | CPVL | | AP | | 0.024 | | 346.018 | | | 2.168 | | 55223.271 |
| ID_79244 | KIAA0895 | | AP | | 0.014 | | 0.205 | | | 0.058 | | 0.728 |
| ID_7936 | EFNA3 | | AP | | 0.014 | | 2.176 | | | 1.169 | | 4.052 |
| ID_79364 | URGCP | | AA | | 0.039 | | 0.195 | | | 0.041 | | 0.920 |
| ID_79567 | PPIA | | AD | | 0.009 | | 0.080 | | | 0.012 | | 0.531 |
| ID_79580 | MYO1G | | ES | | 0.008 | | 0.037 | | | 0.003 | | 0.418 |
| ID_79684 | IKZF1 | | ES | | 0.048 | | 0.284 | | | 0.081 | | 0.991 |
| ID_79695 | FIGNL1 | | ES | | 0.038 | | 2.246 | | | 1.045 | | 4.829 |
| ID_79785 | SUMF2 | | ES | | 0.025 | | 465.077 | | | 2.126 | | 101729.213 |
| ID_79855 | GUSB | | ES | | 0.028 | | 0.103 | | | 0.014 | | 0.786 |
| ID_80031 | ABHD11 | | AA | | 0.025 | | 29846965.539 | | | 8.745 | | 101869556997910 |
| ID_80057 | ELN | | ES | | 0.004 | | 140.990 | | | 4.734 | | 4199.286 |
| ID_80087 | GTF2I | | ES | | 0.004 | | 0.051 | | | 0.007 | | 0.397 |
| ID_80286 | KIAA1324L | | AA | | 0.002 | | 0.000 | | | 0.000 | | 0.003 |
| ID_80361 | STEAP4 | | AT | | 0.002 | | 3.526 | | | 1.586 | | 7.835 |
| ID_80392 | GTPBP10 | | ES | | 0.001 | | 0.008 | | | 0.001 | | 0.138 |
| ID_80396 | GTPBP10 | | ES | | 0.045 | | 0.104 | | | 0.011 | | 0.954 |
| ID_80447 | CDK6 | | AP | | 0.042 | | 2.441 | | | 1.035 | | 5.758 |
| ID_80560 | ASNS | | AP | | 0.039 | | 11.244 | | | 1.128 | | 112.046 |
| ID_80599 | TRRAP | | AA | | 0.045 | | 26.830 | | | 1.073 | | 670.864 |
| ID_80653 | ZNF394 | | ES | | 0.035 | | 17.596 | | | 1.226 | | 252.550 |
| ID_80866 | AZGP1 | | RI | | 0.010 | | 5.626 | | | 1.509 | | 20.976 |
| ID_80892 | AP4M1 | | ES | | 0.048 | | 0.056 | | | 0.003 | | 0.975 |
| ID_80918 | STAG3 | | ES | | 0.020 | | 2.636 | | | 1.165 | | 5.960 |
| ID_81027 | ACHE | | AA | | 0.049 | | 0.423 | | | 0.180 | | 0.995 |
| ID_81164 | ARMC10 | | ES | | 0.008 | | 18.996 | | | 2.161 | | 166.987 |
| ID_81433 | DOCK4 | | AP | | 0.002 | | 0.005 | | | 0.000 | | 0.146 |
| ID_81446 | IFRD1 | | AP | | 0.023 | | 0.025 | | | 0.001 | | 0.600 |
| ID_81457 | TMEM168 | | ES | | 0.018 | | 0.042 | | | 0.003 | | 0.580 |
| ID_81563 | ST7 | | ES | | 0.015 | | 3.154 | | | 1.250 | | 7.955 |
| ID_81656 | ZNF800 | | AD | | 0.025 | | 0.036 | | | 0.002 | | 0.658 |
| ID_81684 | IMPDH1 | | ES | | 0.006 | | 0.048 | | | 0.006 | | 0.410 |
| ID_81744 | AHCYL2 | | AP | | 0.026 | | 0.541 | | | 0.315 | | 0.927 |
| ID_81799 | CEP41 | | AD | | 0.011 | | 0.000 | | | 0.000 | | 0.110 |
| ID_81803 | MEST | | AP | | 0.001 | | 0.349 | | | 0.184 | | 0.660 |
| ID_81872 | C7orf49 | | AD | | 0.017 | | 45.551 | | | 1.986 | | 1044.616 |
| ID_81878 | C7orf49 | | AD | | 0.005 | | 9.155 | | | 1.974 | | 42.448 |
| ID_81942 | TTC26 | | AT | | 0.036 | | 0.011 | | | 0.000 | | 0.740 |
| ID_81972 | TBXAS1 | | AD | | 0.017 | | 84.050 | | | 2.197 | | 3215.679 |
| ID_81995 | RAB19 | | AP | | 0.030 | | 0.091 | | | 0.010 | | 0.796 |
| ID_82055 | SSBP1 | | AD | | 0.013 | | 0.051 | | | 0.005 | | 0.538 |
| ID_82274 | KCNH2 | | AT | | 0.012 | | 5.132 | | | 1.441 | | 18.272 |
| ID_82280 | NOS3 | | ES | | 0.007 | | 0.001 | | | 0.000 | | 0.152 |
| ID_82288 | ABCB8 | | AT | | 0.041 | | 0.316 | | | 0.104 | | 0.956 |
| ID_82365 | SMARCD3 | | ES | | 0.016 | | 0.062 | | | 0.006 | | 0.599 |
| ID_82596 | TNKS | | AT | | 0.044 | | 0.058 | | | 0.004 | | 0.924 |
| ID_82618 | MTMR9 | | ES | | 0.021 | | 10.778 | | | 1.439 | | 80.704 |
| ID_82636 | NEIL2 | | AD | | 0.040 | | 3.022 | | | 1.055 | | 8.658 |
| ID_82719 | FAM86B1 | | AD | | 0.040 | | 4.044 | | | 1.069 | | 15.304 |
| ID_82793 | VPS37A | | RI | | 0.031 | | 0.000 | | | 0.000 | | 0.348 |
| ID_830 | NBPF1 | | AP | | 0.044 | | 0.114 | | | 0.014 | | 0.944 |
| ID_83192 | PBK | | AD | | 0.028 | | 7.193 | | | 1.238 | | 41.773 |
| ID_83216 | FBXO16 | | AT | | 0.014 | | 0.155 | | | 0.035 | | 0.687 |
| ID_83350 | ERLIN2 | | RI | | 0.006 | | 0.277 | | | 0.110 | | 0.698 |
| ID_8338 | ARHGEF11 | | ES | | 0.015 | | 2.481 | | | 1.191 | | 5.169 |
| ID_83392 | WHSC1L1 | | AT | | 0.047 | | 0.138 | | | 0.019 | | 0.977 |
| ID_83399 | LETM2 | | AT | | 0.020 | | 3.041 | | | 1.192 | | 7.758 |
| ID_83410 | FGFR1 | | AP | | 0.028 | | 0.001 | | | 0.000 | | 0.486 |
| ID_83437 | TACC1 | | AP | | 0.033 | | 3.622 | | | 1.113 | | 11.793 |
| ID_83517 | GINS4 | | RI | | 0.016 | | 12.484 | | | 1.600 | | 97.399 |
| ID_83518 | GINS4 | | ES | | 0.043 | | 0.000 | | | 0.000 | | 0.715 |
| ID_83730 | SLC20A2 | | AP | | 0.004 | | 4.401 | | | 1.618 | | 11.972 |
| ID_84084 | CSPP1 | | ES | | 0.043 | | 0.098 | | | 0.010 | | 0.934 |
| ID_84160 | STAU2 | | AA | | 0.039 | | 4558.271 | | | 1.542 | | 13474435.379 |
| ID_842 | ATP13A2 | | AD | | 0.033 | | 9.682 | | | 1.195 | | 78.470 |
| ID_84208 | TCEB1 | | ES | | 0.004 | | 32.844 | | | 2.960 | | 364.453 |
| ID_84397 | NBN | | ES | | 0.026 | | 0.149 | | | 0.028 | | 0.799 |
| ID_84421 | TMEM55A | | ES | | 0.042 | | 4576.902 | | | 1.338 | | 15653874.402 |
| ID_8443 | DCAF8 | | ES | | 0.045 | | 0.248 | | | 0.064 | | 0.967 |
| ID_84563 | KIAA1429 | | AT | | 0.023 | | 7.690 | | | 1.320 | | 44.808 |
| ID_84673 | COX6C | | AP | | 0.044 | | 48.831 | | | 1.106 | | 2155.794 |
| ID_84732 | YWHAZ | | ES | | 0.001 | | 0.002 | | | 0.000 | | 0.078 |
| ID_848204 | RPS6 | | ES | | 0.021 | | 12.556 | | | 1.470 | | 107.260 |
| ID_84842 | OXR1 | | AP | | 0.005 | | 0.310 | | | 0.137 | | 0.699 |
| ID_84885 | NUDCD1 | | ES | | 0.003 | | 1886.340 | | | 13.763 | | 258545.191 |
| ID_85105 | MTSS1 | | AP | | 0.040 | | 3.103 | | | 1.054 | | 9.138 |
| ID_852 | PADI4 | | AT | | 0.049 | | 0.501 | | | 0.252 | | 0.998 |
| ID_85224 | WISP1 | | ES | | 0.006 | | 0.042 | | | 0.004 | | 0.401 |
| ID_85413 | TOP1MT | | AP | | 0.027 | | 0.040 | | | 0.002 | | 0.693 |
| ID_85423 | MROH6 | | AP | | 0.037 | | 0.422 | | | 0.188 | | 0.947 |
| ID_85618 | CYHR1 | | RI | | 0.007 | | 0.266 | | | 0.102 | | 0.694 |
| ID_85662 | ZNF7 | | ES | | 0.046 | | 0.182 | | | 0.034 | | 0.971 |
| ID_85825 | KDM4C | | AT | | 0.014 | | 0.062 | | | 0.007 | | 0.573 |
| ID_85946 | ADAMTSL1 | | AT | | 0.017 | | 0.255 | | | 0.084 | | 0.781 |
| ID_86052 | C9orf72 | | AT | | 0.013 | | 0.037 | | | 0.003 | | 0.498 |
| ID_86108 | AQP7 | | RI | | 0.003 | | 0.313 | | | 0.147 | | 0.668 |
| ID_862 | ARHGEF10L | | ES | | 0.043 | | 0.306 | | | 0.097 | | 0.965 |
| ID_86209 | IL11RA | | AP | | 0.022 | | 0.513 | | | 0.289 | | 0.910 |
| ID_86210 | IL11RA | | AT | | 0.007 | | 5.396 | | | 1.593 | | 18.281 |
| ID_86252 | RUSC2 | | AP | | 0.015 | | 3.688 | | | 1.283 | | 10.604 |
| ID_86478 | ZNF658 | | AT | | 0.019 | | 0.018 | | | 0.001 | | 0.514 |
| ID_86492 | CBWD6 | | ES | | 0.000 | | 63.345 | | | 7.004 | | 572.922 |
| ID_86508 | CBWD5 | | ES | | 0.001 | | 535.671 | | | 14.011 | | 20480.384 |
| ID_86532 | TJP2 | | AP | | 0.041 | | 1.890 | | | 1.028 | | 3.476 |
| ID_86591 | GDA | | RI | | 0.034 | | 0.099 | | | 0.012 | | 0.835 |
| ID_86623 | C9orf41 | | ES | | 0.029 | | 128.948 | | | 1.650 | | 10075.516 |
| ID_86668 | TLE4 | | ES | | 0.018 | | 49.941 | | | 1.929 | | 1292.625 |
| ID_86675 | TLE1 | | AT | | 0.001 | | 0.028 | | | 0.003 | | 0.247 |
| ID_86865 | NOL8 | | AD | | 0.020 | | 5.964 | | | 1.323 | | 26.883 |
| ID_86879 | ECM2 | | AA | | 0.038 | | 0.314 | | | 0.106 | | 0.937 |
| ID_86883 | BICD2 | | RI | | 0.004 | | 0.328 | | | 0.155 | | 0.693 |
| ID_86971 | CDC14B | | AP | | 0.015 | | 0.245 | | | 0.079 | | 0.761 |
| ID_87017 | TSTD2 | | AD | | 0.022 | | 6.751 | | | 1.319 | | 34.562 |
| ID_87149 | FAM206A | | AT | | 0.044 | | 0.023 | | | 0.001 | | 0.906 |
| ID_87328 | TNFSF15 | | AP | | 0.030 | | 0.166 | | | 0.033 | | 0.840 |
| ID_8738 | NOS1AP | | AP | | 0.042 | | 0.099 | | | 0.011 | | 0.923 |
| ID_87391 | FBXW2 | | AD | | 0.026 | | 5.077 | | | 1.212 | | 21.271 |
| ID_87430 | GSN | | ES | | 0.037 | | 17.570 | | | 1.183 | | 260.935 |
| ID_87493 | RABGAP1 | | AP | | 0.001 | | 0.066 | | | 0.013 | | 0.350 |
| ID_87573 | GAPVD1 | | ES | | 0.016 | | 4.780 | | | 1.336 | | 17.098 |
| ID_87590 | PBX3 | | ES | | 0.040 | | 0.221 | | | 0.053 | | 0.931 |
| ID_87591 | PBX3 | | ES | | 0.035 | | 0.004 | | | 0.000 | | 0.673 |
| ID_87612 | RALGPS1 | | AA | | 0.028 | | 0.064 | | | 0.006 | | 0.744 |
| ID_87614 | RALGPS1 | | AD | | 0.001 | | 0.043 | | | 0.007 | | 0.273 |
| ID_87640 | STXBP1 | | ES | | 0.038 | | 85.382 | | | 1.291 | | 5648.695 |
| ID_87727 | GOLGA2 | | ES | | 0.040 | | 0.207 | | | 0.046 | | 0.931 |
| ID_87774 | SPTAN1 | | ES | | 0.024 | | 0.233 | | | 0.066 | | 0.823 |
| ID_87785 | CCBL1 | | ES | | 0.011 | | 0.015 | | | 0.001 | | 0.389 |
| ID_87819 | FAM73B | | AA | | 0.006 | | 12.069 | | | 2.013 | | 72.352 |
| ID_87889 | FNBP1 | | ES | | 0.018 | | 2.984 | | | 1.208 | | 7.371 |
| ID_87896 | GPR107 | | ES | | 0.035 | | 0.175 | | | 0.034 | | 0.887 |
| ID_8790 | PBX1 | | ES | | 0.003 | | 0.225 | | | 0.084 | | 0.599 |
| ID_87955 | POMT1 | | ES | | 0.026 | | 0.190 | | | 0.044 | | 0.822 |
| ID_87993 | C9orf9 | | AP | | 0.004 | | 0.385 | | | 0.203 | | 0.731 |
| ID_88097 | OLFM1 | | AP | | 0.024 | | 2.224 | | | 1.112 | | 4.448 |
| ID_88110 | C9orf116 | | AP | | 0.028 | | 3.356 | | | 1.144 | | 9.851 |
| ID_88173 | SEC16A | | ES | | 0.001 | | 6.010 | | | 2.109 | | 17.124 |
| ID_88176 | SEC16A | | ES | | 0.009 | | 2.621 | | | 1.277 | | 5.378 |
| ID_88196 | FAM69B | | AP | | 0.037 | | 0.513 | | | 0.274 | | 0.960 |
| ID_88302 | EXD3 | | ES | | 0.034 | | 3.844 | | | 1.106 | | 13.361 |
| ID_88362 | CSF2RA | | AT | | 0.036 | | 0.246 | | | 0.067 | | 0.909 |
| ID_88387 | ASMTL | | AP | | 0.044 | | 0.012 | | | 0.000 | | 0.896 |
| ID_88571 | AP1S2 | | AT | | 0.045 | | 2.402 | | | 1.020 | | 5.657 |
| ID_88576 | CTPS2 | | AP | | 0.016 | | 0.133 | | | 0.026 | | 0.683 |
| ID_88689 | ACOT9 | | AP | | 0.019 | | 26.045 | | | 1.710 | | 396.788 |
| ID_88694 | ACOT9 | | ES | | 0.009 | | 2.498 | | | 1.255 | | 4.970 |
| ID_88808 | RPGR | | ES | | 0.039 | | 0.281 | | | 0.085 | | 0.936 |
| ID_88823 | BCOR | | AP | | 0.031 | | 0.086 | | | 0.009 | | 0.804 |
| ID_8884 | DCAF6 | | ES | | 0.027 | | 3.787 | | | 1.167 | | 12.292 |
| ID_8886 | DCAF6 | | ES | | 0.010 | | 8.647 | | | 1.686 | | 44.342 |
| ID_89034 | SLC35A2 | | AT | | 0.017 | | 0.111 | | | 0.018 | | 0.672 |
| ID_8912 | NME7 | | ES | | 0.016 | | 0.000 | | | 0.000 | | 0.185 |
| ID_89209 | IQSEC2 | | AP | | 0.039 | | 3.113 | | | 1.061 | | 9.129 |
| ID_89261 | TRO | | ES | | 0.031 | | 0.006 | | | 0.000 | | 0.631 |
| ID_89302 | ARHGEF9 | | AP | | 0.005 | | 0.197 | | | 0.063 | | 0.614 |
| ID_89356 | STARD8 | | ES | | 0.001 | | 0.000 | | | 0.000 | | 0.036 |
| ID_89382 | DLG3 | | ES | | 0.013 | | 0.012 | | | 0.000 | | 0.383 |
| ID_89661 | ARMCX3 | | AP | | 0.039 | | 7.153 | | | 1.101 | | 46.473 |
| ID_89768 | MORF4L2 | | ES | | 0.018 | | 711234.265 | | | 9.736 | | 51956375962.0389 |
| ID_89957 | UBE2A | | ES | | 0.019 | | 0.012 | | | 0.000 | | 0.481 |
| ID_90050 | XPNPEP2 | | AT | | 0.044 | | 0.166 | | | 0.029 | | 0.955 |
| ID_90064 | BCORL1 | | ES | | 0.026 | | 3.474 | | | 1.159 | | 10.413 |
| ID_90066 | ELF4 | | AP | | 0.007 | | 0.140 | | | 0.033 | | 0.588 |
| ID_90130 | MBNL3 | | AP | | 0.041 | | 0.457 | | | 0.215 | | 0.969 |
| ID_90151 | PHF6 | | ES | | 0.035 | | 0.004 | | | 0.000 | | 0.672 |
| ID_90168 | FAM122C | | RI | | 0.027 | | 3.095 | | | 1.138 | | 8.417 |
| ID_90198 | MAP7D3 | | AT | | 0.035 | | 2.719 | | | 1.073 | | 6.888 |
| ID_90294 | IDS | | ES | | 0.014 | | 0.009 | | | 0.000 | | 0.390 |
| ID_90313 | CXorf40A | | AD | | 0.012 | | 0.149 | | | 0.034 | | 0.663 |
| ID_90410 | ZNF185 | | AA | | 0.030 | | 5.514 | | | 1.179 | | 25.785 |
| ID_90441 | HAUS7 | | ES | | 0.028 | | 0.000 | | | 0.000 | | 0.391 |
| ID_90586 | TAZ | | ES | | 0.008 | | 0.171 | | | 0.046 | | 0.634 |
| ID_90634 | FAM3A | | AA | | 0.022 | | 3.003 | | | 1.169 | | 7.715 |
| ID_90667 | F8 | | AP | | 0.024 | | 0.408 | | | 0.188 | | 0.887 |
| ID_9071 | SEC16B | | AT | | 0.046 | | 0.005 | | | 0.000 | | 0.911 |
| ID_9103 | ABL2 | | AT | | 0.008 | | 0.043 | | | 0.004 | | 0.438 |
| ID_91172 | ZBTB7B | | ES | | 0.044 | | 0.089 | | | 0.008 | | 0.934 |
| ID_91278 | DCAF6 | | ME | | 0.030 | | 2.285 | | | 1.085 | | 4.811 |
| ID_9183 | SMG7 | | ES | | 0.045 | | 0.142 | | | 0.021 | | 0.961 |
| ID_9324 | PTPRC | | ES | | 0.015 | | 3.915 | | | 1.305 | | 11.749 |
| ID_9337 | CAMSAP2 | | AA | | 0.028 | | 0.175 | | | 0.037 | | 0.826 |
| ID_9350 | TMEM9 | | AP | | 0.038 | | 123.708 | | | 1.298 | | 11786.496 |
| ID_93912 | KNOP1 | | ES | | 0.036 | | 0.005 | | | 0.000 | | 0.699 |
| ID_94367 | DHX33 | | ES | | 0.021 | | 22.130 | | | 1.595 | | 307.102 |
| ID_9460 | ZC3H11A | | RI | | 0.028 | | 5850.414 | | | 2.563 | | 13353038.418 |
| ID_94791 | SEH1L | | ES | | 0.008 | | 0.000 | | | 0.000 | | 0.025 |
| ID_9481 | MDM4 | | ES | | 0.040 | | 0.000 | | | 0.000 | | 0.600 |
| ID_94824 | DYM | | AA | | 0.009 | | 0.002 | | | 0.000 | | 0.221 |
| ID_95194 | SAMD4B | | ES | | 0.041 | | 2.307 | | | 1.035 | | 5.142 |
| ID_9525 | DSTYK | | ES | | 0.026 | | 0.202 | | | 0.050 | | 0.823 |
| ID_9586 | RASSF5 | | AP | | 0.019 | | 2.566 | | | 1.165 | | 5.649 |
| ID_96058 | BCL2L13 | | ES | | 0.020 | | 0.039 | | | 0.003 | | 0.602 |
| ID_96084 | ARVCF | | ES | | 0.009 | | 0.370 | | | 0.176 | | 0.779 |
| ID_9663 | CD46 | | ES | | 0.012 | | 0.357 | | | 0.160 | | 0.800 |
| ID_96683 | DCAF16 | | ES | | 0.004 | | 0.053 | | | 0.007 | | 0.402 |
| ID_96873 | DROSHA | | ES | | 0.018 | | 0.065 | | | 0.007 | | 0.622 |
| ID_96875 | DROSHA | | ES | | 0.012 | | 0.127 | | | 0.025 | | 0.638 |
| ID_96931 | H2AFY | | ME | | 0.003 | | 0.113 | | | 0.026 | | 0.486 |
| ID_9729 | TMEM206 | | ES | | 0.020 | | 15.676 | | | 1.553 | | 158.230 |
| ID_97393 | NUPL2 | | ES | | 0.028 | | 0.017 | | | 0.000 | | 0.643 |
| ID_97869 | CTSB | | ES | | 0.042 | | 4497.624 | | | 1.358 | | 14897365.214 |
| ID_9805 | USH2A | | AT | | 0.003 | | 0.111 | | | 0.027 | | 0.461 |
| ID_98066 | KCNK9 | | AT | | 0.009 | | 0.463 | | | 0.260 | | 0.824 |
| ID_98132 | TPM2 | | ES | | 0.044 | | 0.015 | | | 0.000 | | 0.895 |
| ID_98133 | TPM2 | | ES | | 0.023 | | 0.383 | | | 0.168 | | 0.874 |
| ID_98167 | WNK2 | | ES | | 0.023 | | 2.680 | | | 1.147 | | 6.265 |
| ID_98321 | MED14 | | ES | | 0.047 | | 0.011 | | | 0.000 | | 0.945 |
| ID_98324 | RBM10 | | ES | | 0.006 | | 0.008 | | | 0.000 | | 0.245 |
| ID_9952 | NVL | | AA | | 0.029 | | 7231.133 | | | 2.491 | | 20992796.883 |
| ID_9953 | NVL | | ES | | 0.005 | | 0.130 | | | 0.031 | | 0.546 |
| ID_9989 | ENAH | | ES | | 0.048 | | 0.536 | | | 0.289 | | 0.994 |
| ID_9997 | EPHX1 | | AP | | 0.006 | | 0.089 | | | 0.016 | | 0.506 |
