# Supplementary material for: Identification and validation of critical alternative splicing events and splicing factors in gastric cancer progression
Source: J Cell Mol Med. 2020 Sep 16;24(21):12667–80. doi: 10.1111/jcmm.15835 (PMC7686978; doi:10.1111/jcmm.15835)
Supplement: Supplementary file 9 — Table S3 [file JCMM-24-12667-s009.docx]

Table S3. Genes whose mRNA levels were significantly associated with prognosis in GC in the TCGA database (P<0.05).

| Gene symbol | P value | HR | Low 95%CI | High 95%CI |
| --- | --- | --- | --- | --- |
| MATN3 | 0.0001 | 1.213526 | 1.100784 | 1.337815 |
| TNP1 | 0.000123 | 1.085511 | 1.040996 | 1.131929 |
| PTPRD | 0.000127 | 1.225121 | 1.104256 | 1.359216 |
| AJ239318.1 | 0.000128 | 1.115105 | 1.054646 | 1.179029 |
| NRP1 | 0.00013 | 1.495235 | 1.216822 | 1.837351 |
| GPR42 | 0.00013 | 1.081972 | 1.039176 | 1.12653 |
| SLC7A2 | 0.000138 | 1.163144 | 1.076186 | 1.257129 |
| MARCKS | 0.000172 | 1.766294 | 1.312665 | 2.376686 |
| AKR1B1 | 0.000185 | 1.388989 | 1.169221 | 1.650066 |
| GH2 | 0.000195 | 1.068025 | 1.031681 | 1.105649 |
| ENSG00000264545 | 0.00021 | 1.13085 | 1.059658 | 1.206826 |
| FRMD7 | 0.000219 | 1.078443 | 1.036112 | 1.122504 |
| TRIM25 | 0.00022 | 0.5684 | 0.421228 | 0.766991 |
| DEFB113 | 0.000223 | 1.101112 | 1.046209 | 1.158897 |
| C19orf52 | 0.000246 | 0.510169 | 0.356001 | 0.7311 |
| TMEM45A | 0.000284 | 1.267026 | 1.115016 | 1.43976 |
| GPX3 | 0.000308 | 1.234187 | 1.100888 | 1.383626 |
| CYTL1 | 0.000318 | 1.226462 | 1.097468 | 1.370619 |
| KCND2 | 0.00037 | 1.195597 | 1.083627 | 1.319137 |
| PPP1R3B | 0.000386 | 1.411885 | 1.167015 | 1.708136 |
| BOLA2 | 0.000439 | 1.315687 | 1.129082 | 1.533133 |
| METTL2B | 0.00048 | 0.581918 | 0.429416 | 0.788579 |
| FGF1 | 0.000487 | 1.306588 | 1.124288 | 1.518447 |
| FEM1A | 0.000508 | 0.630462 | 0.486087 | 0.817718 |
| SLC52A3 | 0.000518 | 0.771093 | 0.665833 | 0.892994 |
| PRTG | 0.000519 | 1.189387 | 1.078412 | 1.311782 |
| PDGFRL | 0.000542 | 1.192903 | 1.079448 | 1.318284 |
| NMNAT1 | 0.000546 | 0.62822 | 0.482681 | 0.817643 |
| VCAN | 0.000558 | 1.270967 | 1.109175 | 1.45636 |
| CGB | 0.000577 | 1.056038 | 1.023758 | 1.089335 |
| ZNF101 | 0.000588 | 0.614522 | 0.46553 | 0.811199 |
| OR10A5 | 0.000602 | 1.0612 | 1.025791 | 1.097833 |
| MEF2B | 0.000625 | 0.807361 | 0.714197 | 0.912677 |
| ZNF22 | 0.000629 | 1.607734 | 1.224609 | 2.11072 |
| MAGEL2 | 0.000642 | 1.159737 | 1.065126 | 1.262752 |
| STK32A | 0.000669 | 1.140326 | 1.057235 | 1.229948 |
| GPR173 | 0.000676 | 1.246292 | 1.097708 | 1.414989 |
| GJA1 | 0.000677 | 1.257509 | 1.101859 | 1.435146 |
| PLOD2 | 0.000685 | 1.356514 | 1.13759 | 1.617567 |
| EVX2 | 0.000688 | 1.070173 | 1.029073 | 1.112915 |
| C19orf71 | 0.000697 | 0.751563 | 0.637194 | 0.886459 |
| FAAH | 0.000727 | 0.776789 | 0.670928 | 0.899353 |
| KRTAP8-1 | 0.000757 | 1.078436 | 1.032072 | 1.126882 |
| ELOVL2 | 0.000821 | 1.174919 | 1.06904 | 1.291285 |
| LOX | 0.000823 | 1.25671 | 1.099226 | 1.436756 |
| INCENP | 0.000827 | 0.652427 | 0.507941 | 0.838011 |
| APOD | 0.000834 | 1.12155 | 1.048562 | 1.199618 |
| LIN7A | 0.000845 | 1.23494 | 1.091005 | 1.397863 |
| OR10J1 | 0.000851 | 1.100805 | 1.040404 | 1.164713 |
| PCDHB5 | 0.000893 | 1.146443 | 1.05764 | 1.242703 |
| CGB8 | 0.000898 | 1.051306 | 1.020712 | 1.082816 |
| C1QL2 | 0.0009 | 1.061403 | 1.024713 | 1.099407 |
| ACKR3 | 0.000915 | 1.328326 | 1.123084 | 1.571075 |
| AKAP8 | 0.000929 | 0.443326 | 0.273918 | 0.717508 |
| SDC2 | 0.000999 | 1.319141 | 1.11853 | 1.555731 |
| ECH1 | 0.001031 | 1.450836 | 1.161708 | 1.811923 |
| ZBTB7A | 0.001042 | 0.628488 | 0.476141 | 0.829582 |
| PDE1B | 0.001081 | 1.228254 | 1.085792 | 1.389407 |
| SNCG | 0.001086 | 1.175625 | 1.066882 | 1.295452 |
| C14orf37 | 0.001094 | 1.296762 | 1.109467 | 1.515676 |
| C4orf32 | 0.001095 | 1.469687 | 1.166376 | 1.851873 |
| SPARC | 0.001121 | 1.284349 | 1.104854 | 1.493004 |
| BCHE | 0.001123 | 1.104153 | 1.040261 | 1.171968 |
| LDB2 | 0.001132 | 1.330031 | 1.120192 | 1.579179 |
| AP003419.11 | 0.001138 | 1.059963 | 1.023427 | 1.097803 |
| INADL | 0.001153 | 0.721468 | 0.592545 | 0.878442 |
| ACSS3 | 0.001159 | 1.192109 | 1.072198 | 1.325431 |
| PLCL1 | 0.001172 | 1.276193 | 1.10143 | 1.478685 |
| TMSB15A | 0.001195 | 1.177484 | 1.066674 | 1.299805 |
| PRR15L | 0.001209 | 0.829733 | 0.741062 | 0.929015 |
| MKNK2 | 0.001226 | 0.628041 | 0.473709 | 0.832653 |
| MBL2 | 0.001229 | 1.068188 | 1.026303 | 1.111782 |
| GAFA3 | 0.001232 | 0.756412 | 0.638584 | 0.89598 |
| CCDC64 | 0.00124 | 0.730261 | 0.603429 | 0.883752 |
| IQCJ | 0.00126 | 1.068716 | 1.026412 | 1.112764 |
| NT5E | 0.001265 | 1.24863 | 1.090954 | 1.429096 |
| OR4N5 | 0.00131 | 1.084985 | 1.032334 | 1.140322 |
| STC1 | 0.001335 | 1.272831 | 1.09842 | 1.474937 |
| AXL | 0.001336 | 1.280391 | 1.100941 | 1.48909 |
| MYL4 | 0.001347 | 1.224878 | 1.082013 | 1.386606 |
| NGFRAP1 | 0.001357 | 1.214761 | 1.07845 | 1.3683 |
| FAM83G | 0.001372 | 0.695635 | 0.556997 | 0.868779 |
| PTTG1IP | 0.001373 | 1.766957 | 1.246868 | 2.503985 |
| SRPX2 | 0.001396 | 1.268882 | 1.096443 | 1.46844 |
| ADAT3 | 0.001403 | 0.721807 | 0.590938 | 0.881658 |
| OR4M2 | 0.00141 | 1.181735 | 1.066593 | 1.309307 |
| ITPRIP | 0.001419 | 1.385335 | 1.13398 | 1.692405 |
| ANAPC2 | 0.001436 | 0.539847 | 0.36952 | 0.788683 |
| OSBPL9 | 0.001452 | 1.852817 | 1.267565 | 2.708288 |
| IQCC | 0.001464 | 0.584712 | 0.420123 | 0.813782 |
| TREML4 | 0.001464 | 1.076542 | 1.028725 | 1.126581 |
| TFPI | 0.001464 | 1.266874 | 1.095083 | 1.465614 |
| APCS | 0.001469 | 1.069595 | 1.026158 | 1.114871 |
| CATSPER1 | 0.001521 | 1.198452 | 1.07157 | 1.340358 |
| ETV2 | 0.001543 | 0.726356 | 0.595944 | 0.885307 |
| GLT8D1 | 0.001562 | 1.846738 | 1.26276 | 2.700784 |
| ZBTB3 | 0.001583 | 0.609755 | 0.448601 | 0.828802 |
| ZFYVE27 | 0.001601 | 0.620582 | 0.461438 | 0.834614 |
| GAMT | 0.001661 | 1.167077 | 1.059946 | 1.285035 |
| PAPPA2 | 0.001665 | 1.108586 | 1.03959 | 1.182161 |
| RNASE1 | 0.001669 | 1.220249 | 1.077826 | 1.381492 |
| TCF20 | 0.001689 | 0.609858 | 0.447896 | 0.830386 |
| TMEM191B | 0.001714 | 0.895916 | 0.836435 | 0.959627 |
| ARMCX1 | 0.00172 | 1.231263 | 1.081083 | 1.402306 |
| SGCE | 0.001735 | 1.226452 | 1.079386 | 1.393555 |
| GPC3 | 0.001739 | 1.109063 | 1.039486 | 1.183298 |
| PYGO1 | 0.001757 | 1.19743 | 1.069613 | 1.340521 |
| CYP19A1 | 0.001764 | 1.170679 | 1.060588 | 1.292196 |
| MAGED1 | 0.001765 | 1.346701 | 1.117501 | 1.622909 |
| GALNT15 | 0.001789 | 1.169353 | 1.059999 | 1.289989 |
| DUSP1 | 0.00181 | 1.250815 | 1.08676 | 1.439636 |
| CDH6 | 0.001824 | 1.301696 | 1.10285 | 1.536395 |
| POLR1A | 0.001827 | 0.564041 | 0.393489 | 0.808517 |
| PWP2 | 0.001835 | 1.159514 | 1.056437 | 1.272649 |
| PPP1R26 | 0.001846 | 0.660802 | 0.509121 | 0.857673 |
| ELOVL4 | 0.001864 | 1.19015 | 1.066528 | 1.3281 |
| GPR176 | 0.001878 | 1.278256 | 1.094969 | 1.492223 |
| NREP | 0.001906 | 1.307692 | 1.103959 | 1.549024 |
| RAB34 | 0.001906 | 1.232885 | 1.08024 | 1.4071 |
| GLE1 | 0.001908 | 0.601657 | 0.436549 | 0.829211 |
| EGF | 0.001918 | 1.086491 | 1.031022 | 1.144943 |
| ZNF557 | 0.001934 | 0.646045 | 0.490131 | 0.851557 |
| DOT1L | 0.001966 | 0.642906 | 0.486034 | 0.85041 |
| PRICKLE1 | 0.001996 | 1.207358 | 1.071378 | 1.360598 |
| PLA2R1 | 0.002019 | 1.242455 | 1.082505 | 1.42604 |
| ARMCX2 | 0.002034 | 1.222266 | 1.075951 | 1.388478 |
| CTHRC1 | 0.002039 | 1.198105 | 1.068114 | 1.343916 |
| TTC29 | 0.002043 | 1.056808 | 1.020341 | 1.094578 |
| CCDC42B | 0.002051 | 0.848035 | 0.763662 | 0.94173 |
| ANXA5 | 0.002052 | 1.424623 | 1.137564 | 1.784119 |
| TRIM6 | 0.002069 | 1.217919 | 1.074331 | 1.380698 |
| IGFBP1 | 0.002072 | 1.072964 | 1.025936 | 1.122147 |
| GLT8D2 | 0.002084 | 1.243549 | 1.08239 | 1.428702 |
| TMEM259 | 0.002092 | 0.514317 | 0.33673 | 0.785561 |
| CACTIN | 0.002092 | 0.536139 | 0.360432 | 0.797502 |
| ABCB5 | 0.002096 | 1.100964 | 1.035519 | 1.170545 |
| FKBP10 | 0.002113 | 1.180999 | 1.062139 | 1.31316 |
| TYK2 | 0.002197 | 0.552383 | 0.3778 | 0.807641 |
| GPX8 | 0.002201 | 1.27042 | 1.089947 | 1.480774 |
| ZNF562 | 0.002225 | 0.624441 | 0.461777 | 0.844404 |
| TIMP2 | 0.00225 | 1.277129 | 1.091644 | 1.49413 |
| RGS2 | 0.002268 | 1.221698 | 1.074307 | 1.38931 |
| MMP16 | 0.002278 | 1.225891 | 1.075568 | 1.397225 |
| NCLN | 0.00228 | 0.66382 | 0.510198 | 0.863698 |
| MTA2 | 0.002307 | 0.53084 | 0.353248 | 0.797716 |
| MSI2 | 0.002314 | 0.649477 | 0.492021 | 0.857321 |
| AFAP1L1 | 0.002322 | 1.355588 | 1.114544 | 1.648764 |
| THPO | 0.002332 | 1.114422 | 1.039341 | 1.194927 |
| FUT2 | 0.002344 | 0.832116 | 0.739216 | 0.936691 |
| SOAT1 | 0.002377 | 1.40276 | 1.127649 | 1.74499 |
| NOX4 | 0.002424 | 1.240968 | 1.079355 | 1.426779 |
| FBN1 | 0.002443 | 1.217646 | 1.072033 | 1.383037 |
| DAB2 | 0.002478 | 1.312597 | 1.100572 | 1.565469 |
| VGLL3 | 0.002495 | 1.188596 | 1.062678 | 1.329434 |
| RAI14 | 0.002555 | 1.364086 | 1.114902 | 1.668964 |
| MYB | 0.002555 | 0.845374 | 0.757971 | 0.942855 |
| TMEM55A | 0.00257 | 1.251934 | 1.081796 | 1.44883 |
| IMPACT | 0.002575 | 1.40502 | 1.126315 | 1.752691 |
| EBF2 | 0.002588 | 1.155868 | 1.051923 | 1.270085 |
| FAM19A3 | 0.002589 | 1.088997 | 1.030242 | 1.151103 |
| PHLDB2 | 0.002599 | 1.192394 | 1.06337 | 1.337073 |
| FAM127C | 0.002627 | 1.244763 | 1.079286 | 1.435611 |
| TCEAL7 | 0.002638 | 1.185023 | 1.060894 | 1.323677 |
| ENSG00000198739 | 0.00267 | 1.063975 | 1.021778 | 1.107915 |
| KCNJ8 | 0.002703 | 1.241723 | 1.077924 | 1.430413 |
| EGFLAM | 0.002716 | 1.283436 | 1.090254 | 1.510847 |
| COA7 | 0.002723 | 0.592255 | 0.420492 | 0.834181 |
| SLC25A15 | 0.002727 | 0.69942 | 0.553602 | 0.883645 |
| REPIN1 | 0.002729 | 0.676616 | 0.52406 | 0.873582 |
| RGS4 | 0.002732 | 1.162013 | 1.053309 | 1.281937 |
| MED18 | 0.002766 | 0.619425 | 0.452639 | 0.847666 |
| CYR61 | 0.002774 | 1.202447 | 1.065644 | 1.356813 |
| DCBLD2 | 0.002786 | 1.228099 | 1.073365 | 1.405139 |
| ENSG00000271810 | 0.002788 | 0.947216 | 0.91414 | 0.98149 |
| CTGF | 0.002789 | 1.243357 | 1.077926 | 1.434178 |
| CPZ | 0.002811 | 1.17732 | 1.05776 | 1.310395 |
| VSX2 | 0.002822 | 1.068785 | 1.023129 | 1.116478 |
| BRI3BP | 0.002823 | 0.745539 | 0.614853 | 0.904002 |
| SPRED1 | 0.002825 | 1.631587 | 1.183217 | 2.249863 |
| LHX6 | 0.002845 | 1.25213 | 1.080218 | 1.451401 |
| RCAN1 | 0.00285 | 1.395827 | 1.12121 | 1.737706 |
| MAP4K4 | 0.002882 | 1.457264 | 1.137579 | 1.866787 |
| THBS1 | 0.002889 | 1.201104 | 1.064703 | 1.35498 |
| EDNRA | 0.002892 | 1.236797 | 1.075407 | 1.422407 |
| KCNT2 | 0.00292 | 1.176311 | 1.057007 | 1.309082 |
| NDOR1 | 0.002955 | 0.642471 | 0.479901 | 0.860113 |
| GNG11 | 0.002957 | 1.275811 | 1.086491 | 1.498121 |
| ITGAV | 0.002962 | 1.406677 | 1.123189 | 1.761718 |
| INPP5J | 0.003023 | 0.776309 | 0.656678 | 0.917734 |
| GHR | 0.003026 | 1.159448 | 1.051429 | 1.278563 |
| NID2 | 0.003027 | 1.274177 | 1.085597 | 1.495516 |
| IL17RA | 0.003032 | 0.597799 | 0.425427 | 0.840012 |
| THSD7A | 0.003044 | 1.234854 | 1.074038 | 1.419748 |
| HRNR | 0.003085 | 1.10725 | 1.034999 | 1.184544 |
| CLDN6 | 0.00309 | 1.062185 | 1.020572 | 1.105494 |
| HES5 | 0.003106 | 0.897328 | 0.835155 | 0.96413 |
| GABRG1 | 0.00311 | 1.068274 | 1.022513 | 1.116083 |
| FKBP7 | 0.003118 | 1.366144 | 1.110837 | 1.68013 |
| RECK | 0.003136 | 1.244364 | 1.076348 | 1.438608 |
| EFEMP1 | 0.003151 | 1.162821 | 1.052021 | 1.28529 |
| SPATA13 | 0.003153 | 0.698395 | 0.550312 | 0.886326 |
| MAGEH1 | 0.003162 | 1.27171 | 1.084099 | 1.491788 |
| ADAMTS6 | 0.003175 | 1.237623 | 1.074186 | 1.425928 |
| CC2D2A | 0.0032 | 1.272498 | 1.08411 | 1.493622 |
| ITIH3 | 0.003218 | 1.156219 | 1.049786 | 1.273442 |
| TMEM120B | 0.003227 | 0.57763 | 0.400897 | 0.832272 |
| HTRA3 | 0.003235 | 1.208521 | 1.065374 | 1.370902 |
| FAM53B | 0.003254 | 0.723679 | 0.583441 | 0.897625 |
| DIRC1 | 0.003262 | 1.056623 | 1.018554 | 1.096115 |
| CYB561D1 | 0.003278 | 0.6988 | 0.550307 | 0.887362 |
| SLC2A3 | 0.0033 | 1.240532 | 1.074406 | 1.432345 |
| CD109 | 0.003306 | 1.177865 | 1.055998 | 1.313796 |
| PRDM5 | 0.003334 | 1.182812 | 1.057368 | 1.323139 |
| CYP2U1 | 0.003392 | 1.285908 | 1.086805 | 1.521487 |
| CHAF1A | 0.003415 | 0.716961 | 0.573795 | 0.895849 |
| GNAS | 0.003466 | 1.44724 | 1.129525 | 1.854321 |
| GULP1 | 0.003471 | 1.230116 | 1.070597 | 1.413405 |
| SEC23A | 0.003477 | 1.475552 | 1.13666 | 1.915482 |
| IFNW1 | 0.00348 | 1.05907 | 1.019073 | 1.100637 |
| ZFYVE21 | 0.00348 | 1.664223 | 1.182557 | 2.342077 |
| PCDHB6 | 0.003497 | 1.125463 | 1.039633 | 1.218379 |
| CD36 | 0.003533 | 1.175847 | 1.05459 | 1.311046 |
| POLRMT | 0.003533 | 0.633136 | 0.465721 | 0.860732 |
| OR1C1 | 0.003539 | 1.074539 | 1.023861 | 1.127726 |
| EHMT1 | 0.003558 | 0.498803 | 0.312476 | 0.796236 |
| TFPI2 | 0.00359 | 1.12558 | 1.039437 | 1.218861 |
| CHRNE | 0.00359 | 1.241949 | 1.073417 | 1.436943 |
| ADAMTS18 | 0.003627 | 1.1281 | 1.040104 | 1.223542 |
| MARK2 | 0.003632 | 0.594964 | 0.419299 | 0.844223 |
| GABARAPL2 | 0.003642 | 1.763017 | 1.202982 | 2.58377 |
| ILF3 | 0.003649 | 0.500527 | 0.313889 | 0.79814 |
| PTPRJ | 0.003651 | 0.71312 | 0.567748 | 0.895715 |
| VTN | 0.003675 | 1.094413 | 1.02978 | 1.163103 |
| ST3GAL6 | 0.003679 | 1.284365 | 1.084786 | 1.520663 |
| GUCY1A2 | 0.0037 | 1.250297 | 1.075245 | 1.453848 |
| ZNF317 | 0.00372 | 0.503684 | 0.316905 | 0.800549 |
| TMEM200A | 0.003721 | 1.184876 | 1.056567 | 1.328767 |
| VPS35 | 0.003749 | 1.88648 | 1.228175 | 2.897637 |
| FAM198B | 0.003764 | 1.267363 | 1.079667 | 1.487688 |
| SORCS2 | 0.003811 | 1.165521 | 1.050655 | 1.292945 |
| RPS4Y2 | 0.00383 | 1.049118 | 1.015572 | 1.083772 |
| C19orf57 | 0.003964 | 0.814892 | 0.708959 | 0.936654 |
| PNMA1 | 0.003986 | 1.331274 | 1.095659 | 1.617556 |
| LMNB2 | 0.003997 | 0.717237 | 0.571977 | 0.899386 |
| POSTN | 0.004003 | 1.196653 | 1.058936 | 1.352281 |
| PIDD1 | 0.004009 | 0.689146 | 0.534781 | 0.888067 |
| COL5A2 | 0.004014 | 1.240115 | 1.071009 | 1.435923 |
| ASGR2 | 0.004014 | 1.119507 | 1.03664 | 1.208998 |
| PAMR1 | 0.004032 | 1.238914 | 1.070598 | 1.433692 |
| VWF | 0.004038 | 1.264685 | 1.07761 | 1.484236 |
| TMEFF1 | 0.004189 | 1.074686 | 1.022989 | 1.128996 |
| RAB19 | 0.004205 | 0.837998 | 0.74248 | 0.945805 |
| SYN1 | 0.004218 | 1.194732 | 1.057651 | 1.34958 |
| DAZAP1 | 0.004228 | 0.550302 | 0.365487 | 0.828571 |
| IQCA1 | 0.004251 | 1.178659 | 1.053044 | 1.319258 |
| DOK7 | 0.004252 | 0.873722 | 0.796489 | 0.958444 |
| LOXL3 | 0.004252 | 1.371792 | 1.1045 | 1.70377 |
| GDPGP1 | 0.004261 | 0.676005 | 0.516819 | 0.884222 |
| LUM | 0.00427 | 1.205996 | 1.060598 | 1.371327 |
| OR2J2 | 0.004282 | 1.075047 | 1.022974 | 1.12977 |
| APLP2 | 0.004317 | 1.527224 | 1.141849 | 2.042663 |
| PCNXL3 | 0.004382 | 0.660675 | 0.496778 | 0.878646 |
| PXDN | 0.004382 | 1.271344 | 1.077808 | 1.499632 |
| RNF144A | 0.004384 | 1.292271 | 1.083309 | 1.54154 |
| PCDHB4 | 0.004387 | 1.18898 | 1.055495 | 1.339345 |
| PTX4 | 0.004392 | 0.939689 | 0.900317 | 0.980783 |
| NF2 | 0.004415 | 0.647681 | 0.480274 | 0.873439 |
| KLHL38 | 0.004427 | 1.13963 | 1.041532 | 1.246969 |
| RAMP1 | 0.004463 | 1.145977 | 1.043245 | 1.258825 |
| VIM | 0.004467 | 1.303752 | 1.085883 | 1.565333 |
| MYL3 | 0.004475 | 1.093144 | 1.028038 | 1.162374 |
| LBH | 0.004476 | 1.296193 | 1.08388 | 1.550094 |
| C11orf57 | 0.004485 | 0.539406 | 0.352393 | 0.825667 |
| PLTP | 0.004491 | 1.225002 | 1.06498 | 1.409069 |
| OR2T5 | 0.004501 | 1.111197 | 1.033231 | 1.195047 |
| E2F2 | 0.004511 | 0.81936 | 0.714105 | 0.940128 |
| SNAPC4 | 0.004538 | 0.645678 | 0.477326 | 0.873408 |
| TRIM26 | 0.004543 | 0.588705 | 0.408294 | 0.848835 |
| SAFB2 | 0.004582 | 0.508946 | 0.319067 | 0.811824 |
| SWSAP1 | 0.004603 | 0.628569 | 0.455907 | 0.866622 |
| TMEM102 | 0.004612 | 0.736973 | 0.596688 | 0.910239 |
| FZD7 | 0.004682 | 1.221672 | 1.063391 | 1.403514 |
| OR8I2 | 0.004712 | 1.080812 | 1.024102 | 1.140662 |
| CTNNAL1 | 0.004718 | 1.37369 | 1.102164 | 1.712107 |
| HOXD11 | 0.004718 | 1.04255 | 1.012848 | 1.073123 |
| SLCO2A1 | 0.004722 | 1.22451 | 1.064005 | 1.409228 |
| RBM15 | 0.00474 | 0.591689 | 0.41108 | 0.851647 |
| GRB14 | 0.004757 | 1.144456 | 1.042113 | 1.256849 |
| RABL6 | 0.004792 | 0.628345 | 0.454963 | 0.867803 |
| BASP1 | 0.004793 | 1.224115 | 1.063645 | 1.408793 |
| REXO1 | 0.004799 | 0.660513 | 0.495112 | 0.881168 |
| ADRA1B | 0.004821 | 1.134738 | 1.039263 | 1.238984 |
| PRKD1 | 0.004823 | 1.195606 | 1.055932 | 1.353754 |
| TIRAP | 0.004835 | 0.660926 | 0.495516 | 0.881552 |
| FBXL7 | 0.004835 | 1.221522 | 1.06281 | 1.403935 |
| DDIT4L | 0.004872 | 1.113943 | 1.03333 | 1.200845 |
| NNMT | 0.004921 | 1.198711 | 1.056462 | 1.360113 |
| ZNF521 | 0.004933 | 1.22788 | 1.064145 | 1.416809 |
| KDM4A | 0.004944 | 0.606335 | 0.427753 | 0.859474 |
| CDK3 | 0.004954 | 0.864099 | 0.780399 | 0.956776 |
| FRMD6 | 0.004957 | 1.212296 | 1.059956 | 1.386531 |
| F2R | 0.004998 | 1.302641 | 1.083065 | 1.566731 |
| ELK3 | 0.005007 | 1.428008 | 1.11346 | 1.831414 |
| ST6GALNAC3 | 0.00502 | 1.227852 | 1.063831 | 1.417161 |
| LAMA4 | 0.005061 | 1.257212 | 1.071277 | 1.475418 |
| RASGRF2 | 0.005065 | 1.291444 | 1.079948 | 1.544358 |
| LAMC1 | 0.005097 | 1.289369 | 1.07929 | 1.54034 |
| RAB11FIP4 | 0.005109 | 0.738883 | 0.597837 | 0.913205 |
| SEPTIN4 | 0.005118 | 1.294643 | 1.08052 | 1.551197 |
| LRRC8E | 0.005133 | 0.814627 | 0.705666 | 0.940413 |
| VHL | 0.005142 | 0.615337 | 0.437915 | 0.864642 |
| MFSD12 | 0.005146 | 0.671975 | 0.508635 | 0.887769 |
| NUDT11 | 0.005152 | 1.148858 | 1.042417 | 1.266168 |
| ENSG00000269964 | 0.005168 | 1.069101 | 1.020186 | 1.120361 |
| FAM162B | 0.005176 | 1.227953 | 1.063319 | 1.418077 |
| AC002310.13 | 0.005222 | 1.212048 | 1.059037 | 1.387167 |
| RBM14 | 0.005239 | 0.542089 | 0.352684 | 0.833213 |
| NPTX1 | 0.005284 | 1.079668 | 1.023051 | 1.139418 |
| LEPROT | 0.005287 | 1.720451 | 1.175021 | 2.519063 |
| APH1B | 0.005288 | 1.344162 | 1.091897 | 1.654709 |
| MFAP2 | 0.005361 | 1.17209 | 1.048144 | 1.310693 |
| QKI | 0.005368 | 1.319072 | 1.085428 | 1.60301 |
| PCF11 | 0.005376 | 0.582997 | 0.398722 | 0.852439 |
| GPX7 | 0.005376 | 1.218968 | 1.060337 | 1.401331 |
| LPAR2 | 0.005414 | 0.734902 | 0.591511 | 0.913052 |
| MCEMP1 | 0.005414 | 1.135301 | 1.038186 | 1.241501 |
| NALCN | 0.005443 | 1.134989 | 1.038042 | 1.24099 |
| ANKRD52 | 0.005453 | 0.657802 | 0.489554 | 0.883872 |
| SP6 | 0.005459 | 0.864623 | 0.780308 | 0.958049 |
| ZC2HC1A | 0.005468 | 1.311965 | 1.083244 | 1.588979 |
| ZNF707 | 0.005473 | 0.65079 | 0.480628 | 0.881198 |
| CTB-54O9.9 | 0.005475 | 0.949058 | 0.914682 | 0.984726 |
| LRCH2 | 0.005479 | 1.191172 | 1.052836 | 1.347685 |
| ENSG00000269693 | 0.005486 | 0.826355 | 0.722279 | 0.945427 |
| CALCR | 0.005543 | 1.122736 | 1.034546 | 1.218445 |
| CD59 | 0.005566 | 1.354807 | 1.093055 | 1.679239 |
| PDE1A | 0.005597 | 1.192904 | 1.05296 | 1.351447 |
| ELTD1 | 0.005603 | 1.296592 | 1.078925 | 1.558172 |
| OR52M1 | 0.005608 | 1.061654 | 1.017648 | 1.107564 |
| PJA2 | 0.005608 | 1.385472 | 1.100026 | 1.744987 |
| ANTXR1 | 0.00561 | 1.202561 | 1.055409 | 1.370231 |
| ADAMTS5 | 0.005638 | 1.29768 | 1.079043 | 1.560617 |
| IFNA2 | 0.005639 | 1.07188 | 1.020471 | 1.125879 |
| BAALC | 0.00566 | 1.177013 | 1.048678 | 1.321054 |
| OTOS | 0.005679 | 1.052933 | 1.015141 | 1.092131 |
| KRTAP2-3 | 0.005721 | 1.044955 | 1.012867 | 1.078061 |
| MPO | 0.005745 | 1.13067 | 1.036306 | 1.233628 |
| TTF2 | 0.005772 | 0.665198 | 0.498016 | 0.888503 |
| OR5D16 | 0.005786 | 1.085173 | 1.023969 | 1.150034 |
| SNAI2 | 0.005799 | 1.225576 | 1.060675 | 1.416112 |
| CLCF1 | 0.005804 | 1.260287 | 1.069271 | 1.485426 |
| SVEP1 | 0.005831 | 1.151865 | 1.041724 | 1.273651 |
| KRTAP20-1 | 0.005838 | 1.072914 | 1.020549 | 1.127965 |
| ERG | 0.005847 | 1.258356 | 1.068645 | 1.481747 |
| IGSF21 | 0.005857 | 1.168464 | 1.045982 | 1.305288 |
| EIF1AD | 0.005904 | 0.510957 | 0.316794 | 0.824124 |
| GNAI1 | 0.005953 | 1.218407 | 1.058411 | 1.402588 |
| TACC1 | 0.005958 | 1.258434 | 1.068274 | 1.482445 |
| ABCA1 | 0.005969 | 1.303655 | 1.079119 | 1.57491 |
| KRTAP20-3 | 0.005975 | 1.066314 | 1.018603 | 1.11626 |
| PSAPL1 | 0.006016 | 1.059667 | 1.016742 | 1.104404 |
| ERRFI1 | 0.006036 | 1.264498 | 1.06947 | 1.495092 |
| PRSS23 | 0.006066 | 1.227766 | 1.060395 | 1.421553 |
| SMARCA1 | 0.00609 | 1.217451 | 1.05777 | 1.401237 |
| ECM2 | 0.006144 | 1.229321 | 1.060537 | 1.424966 |
| FBXW9 | 0.006151 | 0.654233 | 0.482954 | 0.886255 |
| SERPINF1 | 0.006206 | 1.180215 | 1.048152 | 1.328917 |
| ENGASE | 0.006212 | 0.710266 | 0.555903 | 0.907491 |
| CCDC134 | 0.006222 | 0.696255 | 0.53719 | 0.902419 |
| SAMD1 | 0.006237 | 0.688104 | 0.526399 | 0.899484 |
| CDH11 | 0.006254 | 1.209728 | 1.055392 | 1.386633 |
| MCC | 0.006269 | 1.21661 | 1.057044 | 1.400263 |
| SLITRK4 | 0.006281 | 1.122379 | 1.033186 | 1.219273 |
| SRMS | 0.006305 | 0.888748 | 0.816629 | 0.967236 |
| ADAMTS12 | 0.006314 | 1.186822 | 1.049546 | 1.342052 |
| GPR133 | 0.006342 | 1.1269 | 1.034257 | 1.227841 |
| CPE | 0.006349 | 1.154846 | 1.041413 | 1.280634 |
| CIDEC | 0.006352 | 1.09397 | 1.025634 | 1.166859 |
| SMAD5 | 0.006362 | 1.62577 | 1.146698 | 2.304989 |
| RASAL1 | 0.006366 | 0.888882 | 0.816761 | 0.967371 |
| MUM1L1 | 0.006383 | 1.057774 | 1.01593 | 1.101341 |
| KIFAP3 | 0.006401 | 1.417784 | 1.103123 | 1.822201 |
| RAB3B | 0.006404 | 1.137639 | 1.036914 | 1.248148 |
| PCDHGA3 | 0.006417 | 1.144173 | 1.038561 | 1.260524 |
| GPSM2 | 0.006417 | 0.689529 | 0.527787 | 0.900837 |
| TSSK6 | 0.006419 | 0.74844 | 0.607664 | 0.921829 |
| DLC1 | 0.006447 | 1.246243 | 1.063699 | 1.460115 |
| EHD3 | 0.006452 | 1.2468 | 1.063817 | 1.461257 |
| KRTAP2-2 | 0.006459 | 1.076578 | 1.020902 | 1.135291 |
| USP51 | 0.006469 | 1.185246 | 1.048776 | 1.339474 |
| DDO | 0.006469 | 1.164335 | 1.043557 | 1.299091 |
| CMTM3 | 0.00647 | 1.254588 | 1.065618 | 1.47707 |
| ZBTB10 | 0.006485 | 1.209341 | 1.054663 | 1.386704 |
| CDH2 | 0.006509 | 1.135994 | 1.036305 | 1.245273 |
| KLHL4 | 0.006531 | 1.131138 | 1.035027 | 1.236174 |
| AKAP5 | 0.006542 | 0.800981 | 0.682586 | 0.939912 |
| DRICH1 | 0.006545 | 0.918655 | 0.864156 | 0.97659 |
| SLC22A16 | 0.006566 | 1.102443 | 1.027576 | 1.182764 |
| CDC37 | 0.006571 | 0.600366 | 0.415549 | 0.86738 |
| NPC2 | 0.006574 | 1.447391 | 1.108598 | 1.889721 |
| MAPK14 | 0.006577 | 1.62656 | 1.14524 | 2.310168 |
| CNTN1 | 0.006589 | 1.091608 | 1.024721 | 1.16286 |
| IPP | 0.006621 | 0.616639 | 0.434985 | 0.874155 |
| LARP6 | 0.006628 | 1.174154 | 1.045659 | 1.318438 |
| IGFBP7 | 0.006636 | 1.243216 | 1.062388 | 1.454823 |
| SLC12A7 | 0.006636 | 0.758511 | 0.621287 | 0.926043 |
| YPEL4 | 0.006663 | 1.195179 | 1.050744 | 1.359466 |
| RAVER1 | 0.006672 | 0.689703 | 0.527348 | 0.902042 |
| PROS1 | 0.006674 | 1.212741 | 1.054979 | 1.394094 |
| FIBIN | 0.006687 | 1.175612 | 1.045887 | 1.321427 |
| NKAIN4 | 0.006709 | 1.115051 | 1.030627 | 1.20639 |
| OSBPL1A | 0.006724 | 1.264769 | 1.067183 | 1.498937 |
| ANKRD53 | 0.006755 | 1.215028 | 1.055312 | 1.398917 |
| NR1D1 | 0.006761 | 1.287134 | 1.072244 | 1.545092 |
| ANKRD6 | 0.006766 | 1.20712 | 1.053379 | 1.383298 |
| ATG4D | 0.006784 | 0.680812 | 0.515401 | 0.899311 |
| CXCR4 | 0.006819 | 1.201072 | 1.051788 | 1.371546 |
| SAPCD1 | 0.006834 | 0.88951 | 0.817156 | 0.968271 |
| TACR1 | 0.006858 | 1.116154 | 1.030688 | 1.208706 |
| ZNF121 | 0.006885 | 0.713035 | 0.557923 | 0.91127 |
| TUSC3 | 0.006901 | 1.175254 | 1.045328 | 1.321329 |
| OLFML2B | 0.006905 | 1.18804 | 1.048428 | 1.346244 |
| PLXDC2 | 0.006916 | 1.194372 | 1.049932 | 1.358684 |
| BEX4 | 0.006935 | 1.177723 | 1.045855 | 1.326218 |
| STK11IP | 0.006938 | 0.628153 | 0.448195 | 0.880367 |
| GXYLT2 | 0.006947 | 1.153987 | 1.040011 | 1.280454 |
| F5 | 0.006949 | 1.110683 | 1.029169 | 1.198653 |
| LETM1 | 0.006965 | 0.693093 | 0.531076 | 0.904537 |
| HSPB2 | 0.006999 | 1.172675 | 1.044487 | 1.316595 |
| PIEZO2 | 0.007014 | 1.219494 | 1.055679 | 1.408729 |
| PDGFRA | 0.007043 | 1.198589 | 1.050636 | 1.367378 |
| MAMDC4 | 0.007067 | 0.796454 | 0.674906 | 0.939893 |
| AMIGO2 | 0.007079 | 1.182937 | 1.046797 | 1.336783 |
| OR8D4 | 0.007079 | 1.048259 | 1.012913 | 1.084839 |
| OR2J3 | 0.007094 | 1.064841 | 1.017237 | 1.114671 |
| FLRT2 | 0.007095 | 1.144265 | 1.03734 | 1.262212 |
| SUGP2 | 0.007141 | 0.638762 | 0.460805 | 0.885445 |
| COMMD10 | 0.007178 | 1.523686 | 1.12088 | 2.071246 |
| DPP9 | 0.007198 | 0.564186 | 0.371654 | 0.856458 |
| C22orf29 | 0.007208 | 0.695483 | 0.533642 | 0.906407 |
| TF | 0.007215 | 1.074854 | 1.019719 | 1.132971 |
| IFNA17 | 0.007218 | 1.074862 | 1.019717 | 1.13299 |
| RASSF8 | 0.007222 | 1.167848 | 1.042851 | 1.307827 |
| SH3BP1 | 0.007264 | 0.789671 | 0.664617 | 0.938256 |
| ZNF331 | 0.007275 | 1.204956 | 1.05158 | 1.380702 |
| STARD8 | 0.007281 | 1.294009 | 1.07198 | 1.562023 |
| FTO | 0.007301 | 1.538971 | 1.123172 | 2.108698 |
| VAT1 | 0.007302 | 1.340339 | 1.082116 | 1.660181 |
| SLC22A17 | 0.007332 | 1.163051 | 1.041476 | 1.298819 |
| DENND1C | 0.007357 | 0.71784 | 0.563306 | 0.914767 |
| EXD3 | 0.007369 | 0.675176 | 0.506582 | 0.899879 |
| DPH2 | 0.007398 | 0.631672 | 0.451333 | 0.884067 |
| DKK1 | 0.007403 | 1.060503 | 1.015878 | 1.107089 |
| HSFY2 | 0.007409 | 1.062371 | 1.016353 | 1.110473 |
| FAM114A2 | 0.007426 | 1.748818 | 1.16152 | 2.633071 |
| MICU3 | 0.007428 | 1.172255 | 1.04349 | 1.316909 |
| LAMP5 | 0.007442 | 1.129818 | 1.033212 | 1.235457 |
| KYNU | 0.007498 | 1.170958 | 1.043039 | 1.314565 |
| RNF217 | 0.00752 | 1.197979 | 1.049361 | 1.367646 |
| PEX5 | 0.007528 | 0.654306 | 0.47938 | 0.893061 |
| CHSY3 | 0.007544 | 1.262705 | 1.064119 | 1.498352 |
| MXRA8 | 0.007574 | 1.178002 | 1.044551 | 1.328504 |
| TRO | 0.007596 | 1.199453 | 1.049526 | 1.370797 |
| RFX5 | 0.007599 | 0.67239 | 0.502406 | 0.899887 |
| TMEM257 | 0.007601 | 1.05307 | 1.013836 | 1.093822 |
| LRRC26 | 0.007639 | 0.900958 | 0.834497 | 0.972712 |
| ZNF823 | 0.007659 | 0.665082 | 0.492826 | 0.897545 |
| C9orf117 | 0.007662 | 0.736888 | 0.588764 | 0.922278 |
| CTSK | 0.007753 | 1.216158 | 1.053003 | 1.404593 |
| CHRDL1 | 0.007753 | 1.071289 | 1.018339 | 1.126992 |
| UPP1 | 0.007778 | 1.320045 | 1.075939 | 1.619534 |
| ABCA6 | 0.007781 | 1.156191 | 1.038993 | 1.286608 |
| KIRREL | 0.007785 | 1.254424 | 1.061555 | 1.482334 |
| STEAP4 | 0.007788 | 1.13743 | 1.034511 | 1.250587 |
| FNDC1 | 0.007795 | 1.113626 | 1.028753 | 1.2055 |
| CLIP4 | 0.0078 | 1.195329 | 1.048106 | 1.36323 |
| NWD2 | 0.007819 | 1.106248 | 1.026924 | 1.1917 |
| CXorf49 | 0.007859 | 1.057331 | 1.014749 | 1.1017 |
| DYNC1I1 | 0.007895 | 1.13755 | 1.03437 | 1.251023 |
| EHF | 0.007908 | 0.86049 | 0.770178 | 0.961391 |
| FAP | 0.007913 | 1.162276 | 1.040186 | 1.298696 |
| USP1 | 0.007951 | 0.628735 | 0.446319 | 0.885707 |
| SUGCT | 0.007958 | 1.190999 | 1.046763 | 1.355111 |
| DAB2IP | 0.007975 | 0.733231 | 0.583027 | 0.922131 |
| HTRA1 | 0.007989 | 1.2384 | 1.057417 | 1.450359 |
| RBMS1 | 0.008018 | 1.282215 | 1.066967 | 1.540886 |
| COLEC11 | 0.008025 | 1.156817 | 1.038704 | 1.288362 |
| LBP | 0.008044 | 1.071261 | 1.01809 | 1.127208 |
| FAM221B | 0.008045 | 0.931929 | 0.884585 | 0.981807 |
| EXT2 | 0.008085 | 1.740375 | 1.154945 | 2.622553 |
| AFF2 | 0.008088 | 1.131602 | 1.032659 | 1.240024 |
| RIMS2 | 0.008118 | 1.095381 | 1.023931 | 1.171817 |
| VSTM2L | 0.008118 | 1.093423 | 1.023455 | 1.168173 |
| BLMH | 0.008131 | 1.431285 | 1.097489 | 1.866603 |
| IFNA14 | 0.008206 | 1.066392 | 1.01676 | 1.118447 |
| HOOK2 | 0.00822 | 0.671668 | 0.500004 | 0.902267 |
| GPR115 | 0.008231 | 1.087389 | 1.021874 | 1.157104 |
| LPPR4 | 0.008315 | 1.163249 | 1.039677 | 1.301508 |
| FERMT2 | 0.00832 | 1.165189 | 1.040114 | 1.305305 |
| DUSP16 | 0.008342 | 0.736007 | 0.586106 | 0.924247 |
| PPP1R1C | 0.008349 | 1.141815 | 1.03466 | 1.260069 |
| ENTPD2 | 0.00836 | 0.88621 | 0.810113 | 0.969455 |
| ASPN | 0.008362 | 1.136109 | 1.033309 | 1.249136 |
| AC009491.1 | 0.008385 | 1.087808 | 1.021823 | 1.158053 |
| ADAMTS1 | 0.008415 | 1.186097 | 1.04469 | 1.346646 |
| SYN2 | 0.008419 | 1.102022 | 1.025193 | 1.184609 |
| SLC5A1 | 0.008425 | 0.916086 | 0.85826 | 0.977808 |
| MYO5A | 0.008461 | 1.265865 | 1.062122 | 1.508691 |
| VEZT | 0.008471 | 1.635951 | 1.134032 | 2.360016 |
| TMTC1 | 0.008485 | 1.156392 | 1.037803 | 1.288531 |
| COL3A1 | 0.008525 | 1.173575 | 1.041645 | 1.322216 |
| KIF24 | 0.008546 | 0.769396 | 0.632842 | 0.935415 |
| NTAN1 | 0.008561 | 1.412947 | 1.091966 | 1.828281 |
| CRISP2 | 0.008582 | 1.037765 | 1.00947 | 1.066853 |
| AVPR1A | 0.0086 | 1.177407 | 1.042366 | 1.329942 |
| MOCS1 | 0.008616 | 1.22406 | 1.052671 | 1.423354 |
| HBG1 | 0.00862 | 1.059469 | 1.014772 | 1.106134 |
| L3MBTL2 | 0.008665 | 0.554849 | 0.357406 | 0.861365 |
| PCOLCE2 | 0.008683 | 1.103143 | 1.025162 | 1.187057 |
| PER1 | 0.008709 | 1.208312 | 1.049009 | 1.391806 |
| HEYL | 0.008748 | 1.197461 | 1.046537 | 1.370151 |
| HCFC1 | 0.008785 | 0.676717 | 0.505306 | 0.906276 |
| SURF6 | 0.008796 | 0.681252 | 0.511216 | 0.907845 |
| LHX9 | 0.008829 | 1.067091 | 1.016468 | 1.120237 |
| PRR25 | 0.008829 | 0.936453 | 0.891547 | 0.983622 |
| SNCA | 0.008845 | 1.15654 | 1.03723 | 1.289574 |
| DGCR8 | 0.008862 | 0.532151 | 0.331803 | 0.853474 |
| RBMS3 | 0.008888 | 1.17794 | 1.041941 | 1.331691 |
| OR51E2 | 0.008902 | 1.148584 | 1.035342 | 1.274211 |
| MMRN2 | 0.00892 | 1.253135 | 1.058159 | 1.484038 |
| HAUS5 | 0.008958 | 0.703016 | 0.539766 | 0.915642 |
| SERPINA5 | 0.008982 | 1.10082 | 1.024289 | 1.183069 |
| MUC15 | 0.009017 | 1.041507 | 1.010197 | 1.073788 |
| DOK5 | 0.009017 | 1.174962 | 1.041042 | 1.326109 |
| PCDHB2 | 0.00902 | 1.143782 | 1.034077 | 1.265126 |
| FSTL1 | 0.00902 | 1.22507 | 1.051938 | 1.426697 |
| ENSG00000266728 | 0.009039 | 0.920795 | 0.86548 | 0.979644 |
| PCDHA12 | 0.009042 | 1.053168 | 1.012992 | 1.094938 |
| CAV1 | 0.009044 | 1.164687 | 1.038716 | 1.305934 |
| UBE2B | 0.009062 | 1.547037 | 1.114759 | 2.146943 |
| MID2 | 0.009084 | 1.186872 | 1.043533 | 1.349899 |
| KBTBD2 | 0.009092 | 1.692748 | 1.139825 | 2.513893 |
| XKR5 | 0.009115 | 1.120315 | 1.028623 | 1.220182 |
| BFAR | 0.009117 | 1.7127 | 1.142983 | 2.566391 |
| LRAT | 0.009125 | 1.079173 | 1.019099 | 1.142788 |
| CKAP4 | 0.009139 | 1.393387 | 1.0858 | 1.788107 |
| ARHGAP29 | 0.009142 | 1.284636 | 1.064116 | 1.550855 |
| NOS3 | 0.009145 | 1.286646 | 1.064519 | 1.555123 |
| LRRC32 | 0.009156 | 1.21251 | 1.048938 | 1.401589 |
| ROR2 | 0.009184 | 1.13639 | 1.032171 | 1.251131 |
| DPH7 | 0.009197 | 0.609139 | 0.419487 | 0.884535 |
| AKAP12 | 0.009199 | 1.137822 | 1.032471 | 1.253924 |
| KRTAP13-3 | 0.00922 | 1.082634 | 1.019826 | 1.149311 |
| OR2T27 | 0.009255 | 1.084174 | 1.020154 | 1.152213 |
| ITGBL1 | 0.009287 | 1.100911 | 1.023986 | 1.183616 |
| STEAP1B | 0.009297 | 1.184393 | 1.042587 | 1.345487 |
| C21orf33 | 0.009299 | 1.11728 | 1.027704 | 1.214665 |
| OLIG3 | 0.009306 | 1.048312 | 1.01169 | 1.086259 |
| PCDH12 | 0.009329 | 1.290976 | 1.064871 | 1.56509 |
| MTHFR | 0.00936 | 0.690137 | 0.52174 | 0.912886 |
| ITGB5 | 0.009386 | 1.388548 | 1.083921 | 1.778788 |
| LCN1 | 0.009412 | 1.05102 | 1.012277 | 1.091246 |
| ABCA2 | 0.009416 | 0.76974 | 0.631762 | 0.937853 |
| FAM229B | 0.009426 | 1.196273 | 1.044897 | 1.369578 |
| PACRGL | 0.009438 | 0.632925 | 0.448086 | 0.89401 |
| CALML6 | 0.009443 | 0.939301 | 0.895919 | 0.984783 |
| PTCHD4 | 0.009468 | 1.129843 | 1.030313 | 1.238988 |
| KIAA1462 | 0.009507 | 1.225876 | 1.050992 | 1.429861 |
| NID1 | 0.009581 | 1.256363 | 1.057129 | 1.493146 |
| PMCH | 0.009605 | 0.947185 | 0.909076 | 0.986891 |
| ZNF490 | 0.009645 | 0.746972 | 0.598916 | 0.931629 |
| ZNF662 | 0.009646 | 1.133029 | 1.030782 | 1.245418 |
| TBX18 | 0.009649 | 1.108171 | 1.025244 | 1.197807 |
| KPNA7 | 0.009655 | 1.128864 | 1.029849 | 1.237398 |
| CDO1 | 0.009659 | 1.108621 | 1.025333 | 1.198675 |
| PEX26 | 0.009715 | 0.635578 | 0.450793 | 0.89611 |
| OLFML1 | 0.009794 | 1.224478 | 1.050061 | 1.427866 |
| BGN | 0.00982 | 1.185985 | 1.041955 | 1.349924 |
| RMI1 | 0.009822 | 0.701139 | 0.535496 | 0.918019 |
| BICC1 | 0.009841 | 1.175492 | 1.03969 | 1.329032 |
| OR4A5 | 0.009842 | 1.085929 | 1.020041 | 1.156073 |
| AVP | 0.009867 | 1.040994 | 1.009707 | 1.073251 |
| LAMA2 | 0.00988 | 1.15963 | 1.036234 | 1.29772 |
| NR3C1 | 0.009919 | 1.256137 | 1.056236 | 1.49387 |
| CRACR2B | 0.009922 | 0.833341 | 0.725504 | 0.957207 |
| SPRED3 | 0.009946 | 1.251224 | 1.055178 | 1.483694 |
| UCK2 | 0.009959 | 0.669132 | 0.492966 | 0.908253 |
| NPY1R | 0.009988 | 1.084834 | 1.01967 | 1.154162 |
| ETV6 | 0.010013 | 0.638016 | 0.453212 | 0.898177 |
| ANGPT1 | 0.010098 | 1.188729 | 1.042021 | 1.356091 |
| LRP12 | 0.010112 | 1.257651 | 1.056064 | 1.497718 |
| THRAP3 | 0.01017 | 0.515798 | 0.311319 | 0.854582 |
| NPR1 | 0.010217 | 1.191878 | 1.042459 | 1.362713 |
| FN1 | 0.010273 | 1.146237 | 1.03278 | 1.272157 |
| NCAM2 | 0.010286 | 1.099029 | 1.022554 | 1.181222 |
| ZNF474 | 0.010368 | 1.135551 | 1.030373 | 1.251466 |
| SMC1A | 0.010386 | 0.639861 | 0.454756 | 0.900312 |
| KCNQ1 | 0.010387 | 0.871218 | 0.784034 | 0.968096 |
| MMRN1 | 0.01041 | 1.119885 | 1.026961 | 1.221218 |
| ZFP36 | 0.010426 | 1.255889 | 1.054954 | 1.495094 |
| HTR2A | 0.010487 | 1.114952 | 1.025811 | 1.211839 |
| MIEF1 | 0.010549 | 0.58611 | 0.389182 | 0.882683 |
| PPFIA3 | 0.010584 | 0.798602 | 0.67211 | 0.9489 |
| SFRP2 | 0.010587 | 1.078311 | 1.017738 | 1.14249 |
| CNTN4 | 0.010597 | 1.174637 | 1.038233 | 1.328962 |
| LCE4A | 0.010617 | 1.069618 | 1.015798 | 1.126289 |
| ARHGAP10 | 0.010649 | 1.218436 | 1.047025 | 1.417909 |
| ZBTB7B | 0.010687 | 0.705275 | 0.539425 | 0.922116 |
| CCDC54 | 0.010688 | 1.050956 | 1.011608 | 1.091835 |
| C2CD4D | 0.01071 | 0.819744 | 0.703692 | 0.954935 |
| P3H2 | 0.01072 | 1.148453 | 1.032619 | 1.277281 |
| CPVL | 0.010771 | 1.172985 | 1.037609 | 1.326023 |
| CXorf36 | 0.010782 | 1.249498 | 1.05287 | 1.482848 |
| OR51A2 | 0.010783 | 1.115435 | 1.025588 | 1.213152 |
| NAA40 | 0.010796 | 0.665896 | 0.487117 | 0.910288 |
| COPS8 | 0.010798 | 1.606693 | 1.11583 | 2.313489 |
| FAM127A | 0.010868 | 1.256813 | 1.05409 | 1.498524 |
| RAD23A | 0.010882 | 0.606491 | 0.412732 | 0.891211 |
| JAM3 | 0.010909 | 1.187302 | 1.040288 | 1.355092 |
| C8orf22 | 0.010909 | 1.046566 | 1.010526 | 1.083892 |
| SLC17A7 | 0.01091 | 1.137788 | 1.030141 | 1.256684 |
| MRPL4 | 0.010947 | 0.70874 | 0.543646 | 0.92397 |
| OR51S1 | 0.010979 | 1.067217 | 1.015034 | 1.122082 |
| SPSB4 | 0.011007 | 1.147202 | 1.031963 | 1.275309 |
| ARHGEF39 | 0.011042 | 0.795825 | 0.667308 | 0.949094 |
| CRAMP1L | 0.011043 | 0.675732 | 0.499449 | 0.914236 |
| LPL | 0.011043 | 1.150328 | 1.032556 | 1.281532 |
| THBD | 0.011066 | 1.230227 | 1.048494 | 1.44346 |
| BSDC1 | 0.011073 | 0.556701 | 0.354295 | 0.874742 |
| EFS | 0.011088 | 1.171936 | 1.036892 | 1.324568 |
| CHRD | 0.011132 | 1.166196 | 1.035662 | 1.313183 |
| CST2 | 0.011145 | 1.099849 | 1.021916 | 1.183724 |
| MARVELD1 | 0.011158 | 1.242756 | 1.050722 | 1.469886 |
| MAGI2 | 0.011219 | 1.178666 | 1.038035 | 1.33835 |
| PDGFRB | 0.01127 | 1.225115 | 1.047081 | 1.433419 |
| HNRNPM | 0.0113 | 0.504679 | 0.297333 | 0.85662 |
| PDDC1 | 0.011313 | 0.715442 | 0.552133 | 0.927056 |
| THOP1 | 0.011339 | 0.699959 | 0.53107 | 0.922559 |
| S100Z | 0.011349 | 1.114832 | 1.024855 | 1.21271 |
| SDF2 | 0.011358 | 1.570252 | 1.107244 | 2.226873 |
| KEAP1 | 0.011399 | 0.623769 | 0.432757 | 0.899091 |
| ANKRD50 | 0.011432 | 1.347715 | 1.069471 | 1.698351 |
| ANKRD33 | 0.011438 | 1.053329 | 1.011759 | 1.096608 |
| FAM73B | 0.011473 | 0.673641 | 0.495913 | 0.915064 |
| PAPPA | 0.01151 | 1.173251 | 1.036495 | 1.328051 |
| LCE2D | 0.011554 | 1.063645 | 1.013912 | 1.115816 |
| UBE2Q2 | 0.011573 | 1.333624 | 1.066537 | 1.667596 |
| DNAJC16 | 0.011576 | 0.633785 | 0.444829 | 0.903006 |
| INHBA | 0.011589 | 1.169311 | 1.035592 | 1.320296 |
| DGKI | 0.011604 | 1.164417 | 1.034599 | 1.310524 |
| RANGAP1 | 0.01162 | 0.652144 | 0.467892 | 0.908954 |
| FREM1 | 0.011658 | 1.093533 | 1.020135 | 1.172211 |
| BCL11B | 0.011665 | 0.802179 | 0.675895 | 0.952057 |
| ADAM12 | 0.011685 | 1.139215 | 1.029452 | 1.26068 |
| SOHLH1 | 0.011745 | 1.03989 | 1.008727 | 1.072015 |
| ENSG00000256966 | 0.011763 | 0.934208 | 0.88603 | 0.985007 |
| OR5L2 | 0.011817 | 1.09303 | 1.019897 | 1.171407 |
| PKIA | 0.01186 | 1.143013 | 1.029994 | 1.268433 |
| C2orf82 | 0.011875 | 0.869147 | 0.779192 | 0.969488 |
| TCF3 | 0.011892 | 0.712386 | 0.546952 | 0.927857 |
| MYO5C | 0.011972 | 0.800811 | 0.673426 | 0.952293 |
| COL1A2 | 0.011983 | 1.170693 | 1.035273 | 1.323827 |
| ARSK | 0.012039 | 1.524761 | 1.096992 | 2.119337 |
| ENSG00000259900 | 0.01207 | 0.953727 | 0.919089 | 0.98967 |
| FAM110B | 0.012096 | 1.146509 | 1.030385 | 1.27572 |
| INIP | 0.012135 | 0.557905 | 0.353604 | 0.880246 |
| OMD | 0.012152 | 1.089585 | 1.018917 | 1.165155 |
| MYCL | 0.012161 | 0.826359 | 0.711906 | 0.959212 |
| TRAF2 | 0.012206 | 0.67425 | 0.495391 | 0.917686 |
| ENSG00000261717 | 0.01223 | 0.936987 | 0.890474 | 0.98593 |
| OLFM3 | 0.012246 | 1.049415 | 1.010549 | 1.089775 |
| METTL2A | 0.012253 | 0.575829 | 0.373874 | 0.886873 |
| AEBP1 | 0.012277 | 1.169387 | 1.034587 | 1.321751 |
| CPLX4 | 0.012289 | 1.056563 | 1.012022 | 1.103065 |
| CACNB3 | 0.012301 | 1.368875 | 1.070535 | 1.750359 |
| EPDR1 | 0.012354 | 1.152929 | 1.031303 | 1.288899 |
| KCNJ15 | 0.012408 | 1.112639 | 1.023335 | 1.209737 |
| RERG | 0.012416 | 1.121418 | 1.025066 | 1.226826 |
| CDK14 | 0.012416 | 1.181549 | 1.036699 | 1.346638 |
| GTPBP3 | 0.012425 | 0.677875 | 0.499764 | 0.919464 |
| DOK6 | 0.012464 | 1.195678 | 1.039285 | 1.375605 |
| CDR2 | 0.012469 | 1.58906 | 1.104995 | 2.285178 |
| OR4K15 | 0.012478 | 1.057882 | 1.012199 | 1.105626 |
| MAGEA11 | 0.012487 | 1.036712 | 1.007797 | 1.066458 |
| AKT3 | 0.012522 | 1.185546 | 1.037289 | 1.354995 |
| ACLY | 0.01255 | 1.309011 | 1.059559 | 1.617192 |
| PLXDC1 | 0.012574 | 1.271855 | 1.052971 | 1.536239 |
| MMD | 0.012578 | 1.27111 | 1.052829 | 1.534646 |
| FGF7 | 0.012595 | 1.119948 | 1.02459 | 1.22418 |
| CREM | 0.01261 | 1.41955 | 1.077974 | 1.869359 |
| GMCL1 | 0.012627 | 0.634263 | 0.443493 | 0.907094 |
| SHQ1 | 0.012635 | 0.594303 | 0.39482 | 0.894576 |
| ZNF282 | 0.012653 | 0.636321 | 0.44602 | 0.907817 |
| PDE2A | 0.012659 | 1.1646 | 1.033128 | 1.312804 |
| CHERP | 0.012698 | 0.616601 | 0.42155 | 0.9019 |
| ZC3H7B | 0.012704 | 0.668561 | 0.487092 | 0.917636 |
| SPRY1 | 0.012706 | 1.387821 | 1.072467 | 1.795905 |
| PCDHGA12 | 0.012715 | 1.172662 | 1.034572 | 1.329184 |
| EDDM3A | 0.012759 | 1.043612 | 1.009134 | 1.079268 |
| UHRF1 | 0.012772 | 0.810259 | 0.686594 | 0.956197 |
| ZNF300 | 0.012773 | 1.147928 | 1.029803 | 1.279602 |
| ENPEP | 0.012784 | 1.1881 | 1.037355 | 1.36075 |
| OR13C9 | 0.012794 | 1.072131 | 1.014924 | 1.132563 |
| MAP7D2 | 0.012839 | 1.07197 | 1.014863 | 1.13229 |
| GDF6 | 0.012842 | 1.082718 | 1.017013 | 1.152668 |
| RAB31 | 0.012855 | 1.221258 | 1.043319 | 1.429546 |
| HIGD1B | 0.012886 | 1.296792 | 1.056613 | 1.591566 |
| STARD5 | 0.012895 | 0.784658 | 0.648139 | 0.949933 |
| ARRDC3 | 0.012906 | 1.33944 | 1.063828 | 1.686456 |
| CAV2 | 0.012921 | 1.178087 | 1.035285 | 1.340588 |
| TRMT1 | 0.012952 | 0.664774 | 0.481748 | 0.917337 |
| P4HA3 | 0.012953 | 1.188998 | 1.037256 | 1.362938 |
| C8orf4 | 0.01302 | 1.163135 | 1.032356 | 1.310481 |
| PIM1 | 0.013056 | 1.282384 | 1.053725 | 1.560663 |
| HIST1H4B | 0.013059 | 0.912885 | 0.849492 | 0.981008 |
| PARD6A | 0.013072 | 0.781336 | 0.642995 | 0.94944 |
| GPR78 | 0.013098 | 1.122276 | 1.024526 | 1.229352 |
| HDGFRP3 | 0.013122 | 1.176257 | 1.03465 | 1.337246 |
| TAF6L | 0.013134 | 0.619887 | 0.424799 | 0.904569 |
| MAP3K9 | 0.013143 | 0.762262 | 0.615072 | 0.944676 |
| PNMA2 | 0.013173 | 1.128578 | 1.025651 | 1.241833 |
| PCDHB12 | 0.013186 | 1.190216 | 1.037114 | 1.365918 |
| C10orf10 | 0.013201 | 1.185439 | 1.03622 | 1.356147 |
| TBX3 | 0.013249 | 0.864259 | 0.770039 | 0.970008 |
| KRTAP21-1 | 0.013291 | 1.065028 | 1.013214 | 1.119491 |
| THY1 | 0.013331 | 1.199594 | 1.038585 | 1.385565 |
| COL4A1 | 0.013334 | 1.258146 | 1.048926 | 1.509097 |
| COL10A1 | 0.013344 | 1.088401 | 1.017769 | 1.163936 |
| PRKCSH | 0.013419 | 0.65801 | 0.472216 | 0.916904 |
| DCLK1 | 0.013434 | 1.114253 | 1.022664 | 1.214045 |
| KRTAP19-1 | 0.013436 | 1.066148 | 1.013356 | 1.121691 |
| FBXO17 | 0.013445 | 1.1304 | 1.025705 | 1.245782 |
| TSPYL4 | 0.013517 | 1.341058 | 1.062456 | 1.692716 |
| ENSG00000258539 | 0.013602 | 0.871475 | 0.78127 | 0.972096 |
| NFE2L3 | 0.013613 | 0.79837 | 0.667603 | 0.954751 |
| DZIP1 | 0.013629 | 1.168637 | 1.032542 | 1.32267 |
| OMP | 0.013655 | 0.925192 | 0.869753 | 0.984166 |
| AP4B1 | 0.01368 | 0.678332 | 0.498254 | 0.923495 |
| COPZ2 | 0.013711 | 1.173165 | 1.033248 | 1.332029 |
| DCN | 0.013731 | 1.157382 | 1.030361 | 1.300061 |
| CLDN11 | 0.013737 | 1.123578 | 1.024124 | 1.23269 |
| SMPD3 | 0.013783 | 0.886036 | 0.804698 | 0.975596 |
| PTGER3 | 0.013806 | 1.119933 | 1.023376 | 1.2256 |
| CP | 0.013819 | 1.094525 | 1.018586 | 1.176126 |
| FBLN1 | 0.013871 | 1.115653 | 1.022516 | 1.217272 |
| PDGFD | 0.013888 | 1.188237 | 1.035688 | 1.363255 |
| C1QTNF2 | 0.013889 | 1.157977 | 1.030269 | 1.301516 |
| T | 0.013911 | 1.044675 | 1.008917 | 1.081701 |
| EID1 | 0.013924 | 1.302873 | 1.055181 | 1.608707 |
| TMEM180 | 0.013972 | 0.738638 | 0.580124 | 0.940466 |
| NRG3 | 0.014056 | 1.075432 | 1.014792 | 1.139694 |
| ASPA | 0.014068 | 1.127215 | 1.024461 | 1.240274 |
| HLX | 0.01408 | 1.287539 | 1.052301 | 1.575362 |
| C6 | 0.014133 | 1.072221 | 1.014134 | 1.133635 |
| CPT1C | 0.014135 | 1.163654 | 1.030973 | 1.313409 |
| CTC-360G5.8 | 0.014166 | 1.049299 | 1.00972 | 1.090431 |
| GUCY1B3 | 0.014202 | 1.187953 | 1.035172 | 1.363282 |
| SNCB | 0.014296 | 1.068669 | 1.013367 | 1.126989 |
| TENM3 | 0.014297 | 1.104022 | 1.019981 | 1.194986 |
| OR2L3 | 0.014305 | 1.065043 | 1.012674 | 1.120121 |
| MED22 | 0.014314 | 0.654343 | 0.466026 | 0.918758 |
| STON2 | 0.014338 | 1.232152 | 1.042546 | 1.456241 |
| CHRFAM7A | 0.014365 | 1.142415 | 1.0269 | 1.270925 |
| CSNK1A1L | 0.014373 | 1.148399 | 1.02796 | 1.282948 |
| PLIN2 | 0.014448 | 1.217398 | 1.039857 | 1.425251 |
| ABCC9 | 0.014485 | 1.132584 | 1.025006 | 1.251452 |
| ELL2 | 0.014542 | 1.288725 | 1.051485 | 1.579494 |
| CHI3L2 | 0.014568 | 1.122406 | 1.023092 | 1.231361 |
| DNAJC9 | 0.014569 | 0.697149 | 0.521945 | 0.931165 |
| KCNS2 | 0.014675 | 1.113111 | 1.021317 | 1.213156 |
| SLC35A3 | 0.014741 | 0.767251 | 0.620101 | 0.94932 |
| HGF | 0.014748 | 1.16123 | 1.029769 | 1.309473 |
| FANCC | 0.014775 | 0.645702 | 0.454259 | 0.917827 |
| LHFP | 0.01482 | 1.186965 | 1.034106 | 1.36242 |
| APOC3 | 0.014867 | 1.037093 | 1.007138 | 1.067939 |
| TMEM233 | 0.014868 | 1.23305 | 1.041758 | 1.459467 |
| SLIT2 | 0.014952 | 1.107764 | 1.020116 | 1.202942 |
| PCDHA11 | 0.014991 | 1.056188 | 1.010678 | 1.103747 |
| PTPRM | 0.015012 | 1.227008 | 1.040513 | 1.44693 |
| SELP | 0.015014 | 1.118476 | 1.021972 | 1.224093 |
| GJD2 | 0.015069 | 1.046577 | 1.008856 | 1.085709 |
| STAB2 | 0.015075 | 1.143909 | 1.026375 | 1.274903 |
| C5orf46 | 0.015079 | 1.055122 | 1.010442 | 1.101778 |
| FABP4 | 0.01509 | 1.085236 | 1.015954 | 1.159242 |
| TNFAIP2 | 0.015098 | 0.844981 | 0.737641 | 0.967942 |
| TCN2 | 0.015108 | 1.230254 | 1.040881 | 1.454082 |
| MEOX2 | 0.015111 | 1.086119 | 1.0161 | 1.160963 |
| CNRIP1 | 0.015129 | 1.209284 | 1.037393 | 1.409655 |
| C1orf210 | 0.01518 | 0.859852 | 0.761184 | 0.97131 |
| GPR146 | 0.015207 | 1.221171 | 1.039227 | 1.434968 |
| CA10 | 0.015261 | 1.06342 | 1.011885 | 1.117581 |
| ZNF367 | 0.015273 | 0.774555 | 0.630102 | 0.952123 |
| LTBP2 | 0.015288 | 1.177928 | 1.031929 | 1.344582 |
| WDR5 | 0.015354 | 0.679467 | 0.497119 | 0.928701 |
| FBN2 | 0.015392 | 1.165593 | 1.029716 | 1.3194 |
| C5orf58 | 0.015421 | 1.134946 | 1.024457 | 1.257351 |
| SGCZ | 0.015454 | 1.046032 | 1.008616 | 1.084836 |
| ABCA8 | 0.015476 | 1.093651 | 1.017195 | 1.175855 |
| IL1A | 0.015477 | 1.106196 | 1.019406 | 1.200376 |
| HEXIM2 | 0.015482 | 0.726778 | 0.561297 | 0.941046 |
| PICK1 | 0.015516 | 0.669736 | 0.48407 | 0.926614 |
| PTX3 | 0.015519 | 1.129208 | 1.02337 | 1.245992 |
| CEP78 | 0.015524 | 0.697105 | 0.520452 | 0.933716 |
| RNF43 | 0.015529 | 0.894492 | 0.81725 | 0.979035 |
| C3orf62 | 0.015549 | 0.700541 | 0.525067 | 0.934657 |
| ABCG4 | 0.01555 | 1.193406 | 1.03414 | 1.377202 |
| GRB10 | 0.015648 | 1.297804 | 1.050529 | 1.603282 |
| ZFHX4 | 0.015769 | 1.110822 | 1.019972 | 1.209764 |
| DNMT1 | 0.015776 | 0.687724 | 0.507472 | 0.932003 |
| LRRC55 | 0.015794 | 1.14115 | 1.025129 | 1.270303 |
| MPND | 0.015809 | 0.727321 | 0.561599 | 0.941946 |
| PTGFR | 0.015894 | 1.126822 | 1.022602 | 1.241663 |
| SERINC1 | 0.015915 | 1.529641 | 1.082736 | 2.161009 |
| RHBDD3 | 0.015922 | 0.706988 | 0.533311 | 0.937225 |
| EPPK1 | 0.015947 | 0.863568 | 0.766463 | 0.972974 |
| CORO2B | 0.015977 | 1.150688 | 1.02653 | 1.289863 |
| MFAP5 | 0.01602 | 1.106471 | 1.019019 | 1.201429 |
| ZFPM2 | 0.016042 | 1.140643 | 1.024784 | 1.269601 |
| NSMF | 0.016053 | 0.7999 | 0.666965 | 0.959331 |
| KIF21B | 0.016093 | 0.803638 | 0.672585 | 0.960227 |
| NRK | 0.016093 | 1.079202 | 1.014252 | 1.148311 |
| SSPN | 0.016126 | 1.155665 | 1.027186 | 1.300215 |
| NOVA1 | 0.016156 | 1.102566 | 1.018244 | 1.193871 |
| GLUD2 | 0.016157 | 1.251052 | 1.042345 | 1.501547 |
| FAM155B | 0.016172 | 1.061774 | 1.011154 | 1.114929 |
| PHYHIP | 0.016196 | 1.165574 | 1.028729 | 1.320621 |
| GFPT2 | 0.016215 | 1.142375 | 1.024893 | 1.273324 |
| NGF | 0.016229 | 1.120625 | 1.021248 | 1.229672 |
| BST1 | 0.016308 | 1.188534 | 1.032292 | 1.368425 |
| ZBTB32 | 0.016324 | 0.825449 | 0.70583 | 0.96534 |
| FHL5 | 0.016347 | 1.167027 | 1.028783 | 1.323846 |
| EDA | 0.016348 | 1.138628 | 1.024137 | 1.26592 |
| PIAS4 | 0.016392 | 0.657782 | 0.467222 | 0.926064 |
| UBAC1 | 0.016467 | 0.619597 | 0.419008 | 0.916213 |
| TM4SF18 | 0.016535 | 1.241966 | 1.040292 | 1.482738 |
| TNN | 0.016536 | 1.097634 | 1.017126 | 1.184515 |
| VEGFC | 0.016572 | 1.246375 | 1.0409 | 1.492411 |
| GEM | 0.016601 | 1.178158 | 1.030253 | 1.347297 |
| OPLAH | 0.016635 | 0.8417 | 0.730973 | 0.9692 |
| MDGA2 | 0.016651 | 1.051104 | 1.009083 | 1.094874 |
| MROH2B | 0.016662 | 1.050298 | 1.008938 | 1.093353 |
| FAM180A | 0.01672 | 1.105099 | 1.018242 | 1.199364 |
| MRGPRX1 | 0.016805 | 1.058697 | 1.010335 | 1.109374 |
| SH3BGRL | 0.016828 | 1.222735 | 1.036877 | 1.441907 |
| SIAH3 | 0.016846 | 1.094001 | 1.016299 | 1.177645 |
| C11orf96 | 0.016922 | 1.167802 | 1.028217 | 1.326335 |
| KLF7 | 0.016923 | 1.228774 | 1.037646 | 1.455107 |
| MOXD1 | 0.01696 | 1.137747 | 1.023381 | 1.264893 |
| PDE9A | 0.016964 | 1.133455 | 1.022686 | 1.256222 |
| SRRM4 | 0.016966 | 1.072961 | 1.012689 | 1.13682 |
| PAPSS2 | 0.016986 | 1.213181 | 1.035177 | 1.421793 |
| FRG2 | 0.016988 | 1.053103 | 1.009299 | 1.098808 |
| C10orf90 | 0.016999 | 1.056502 | 1.009876 | 1.105281 |
| MAN2A2 | 0.017006 | 0.730148 | 0.563946 | 0.945332 |
| MAGED4 | 0.017011 | 1.086654 | 1.014963 | 1.163408 |
| FAM171B | 0.017035 | 1.212275 | 1.034966 | 1.41996 |
| CC2D1A | 0.017036 | 0.705523 | 0.529746 | 0.939625 |
| LRP1 | 0.017045 | 1.241215 | 1.039318 | 1.482332 |
| C6orf48 | 0.01705 | 1.294099 | 1.047077 | 1.599398 |
| GLP2R | 0.017083 | 1.10098 | 1.017289 | 1.191557 |
| HIST2H3C | 0.017107 | 0.938728 | 0.891184 | 0.988808 |
| GPRC6A | 0.017108 | 1.041768 | 1.00731 | 1.077404 |
| NTM | 0.017152 | 1.139999 | 1.023553 | 1.269693 |
| BEND6 | 0.017167 | 1.137738 | 1.023177 | 1.265126 |
| SNIP1 | 0.017206 | 0.536697 | 0.32164 | 0.895548 |
| TMEM161A | 0.017278 | 0.700546 | 0.522627 | 0.939036 |
| CYB5RL | 0.017292 | 0.719807 | 0.549103 | 0.94358 |
| NUP50 | 0.017331 | 0.666605 | 0.477307 | 0.930977 |
| C7orf62 | 0.017367 | 1.064006 | 1.010985 | 1.119809 |
| HOXD3 | 0.017377 | 1.156606 | 1.025939 | 1.303916 |
| PKD1L1 | 0.01743 | 1.197054 | 1.032093 | 1.388381 |
| TELO2 | 0.01745 | 0.68922 | 0.507084 | 0.936776 |
| SV2B | 0.017451 | 1.121914 | 1.02039 | 1.23354 |
| MSRB3 | 0.017496 | 1.122113 | 1.020383 | 1.233986 |
| NEK5 | 0.017532 | 0.821499 | 0.698469 | 0.966199 |
| SPAG16 | 0.017549 | 1.157095 | 1.025827 | 1.305161 |
| RNASE2 | 0.017556 | 1.138828 | 1.022971 | 1.267806 |
| CCDC8 | 0.01758 | 1.121904 | 1.020278 | 1.233652 |
| CYP1B1 | 0.017584 | 1.097055 | 1.016294 | 1.184233 |
| WBP5 | 0.017691 | 1.200241 | 1.032215 | 1.39562 |
| RFXAP | 0.017693 | 0.728901 | 0.561296 | 0.946554 |
| SBNO2 | 0.017711 | 0.727299 | 0.559021 | 0.946231 |
| C1orf167 | 0.017746 | 1.064756 | 1.010934 | 1.121443 |
| LMNTD2 | 0.01776 | 0.811814 | 0.683276 | 0.964532 |
| ITSN1 | 0.017779 | 1.407742 | 1.060976 | 1.867844 |
| APCDD1L | 0.017808 | 1.115814 | 1.019124 | 1.221677 |
| PCDH18 | 0.017815 | 1.223879 | 1.03553 | 1.446486 |
| PEG10 | 0.017816 | 1.07596 | 1.012732 | 1.143135 |
| HBE1 | 0.017844 | 1.037011 | 1.006292 | 1.068666 |
| DCBLD1 | 0.017854 | 1.372692 | 1.056176 | 1.784063 |
| GPC6 | 0.017868 | 1.163011 | 1.026382 | 1.317828 |
| G6PC2 | 0.017873 | 0.954117 | 0.91774 | 0.991935 |
| ZDHHC2 | 0.017883 | 1.162045 | 1.026218 | 1.31585 |
| PGPEP1 | 0.017976 | 0.745807 | 0.584947 | 0.950903 |
| CD93 | 0.01802 | 1.231807 | 1.036367 | 1.464103 |
| FAM26E | 0.018024 | 1.173907 | 1.027848 | 1.340721 |
| FXYD2 | 0.018169 | 1.091748 | 1.015058 | 1.174231 |
| MROH1 | 0.018171 | 0.735798 | 0.57043 | 0.949108 |
| NETO2 | 0.018202 | 1.144656 | 1.023237 | 1.280483 |
| DPP10 | 0.018229 | 1.049892 | 1.008303 | 1.093196 |
| SMCR8 | 0.018247 | 0.720754 | 0.549173 | 0.945944 |
| TLN2 | 0.01833 | 0.818241 | 0.692621 | 0.966645 |
| TMEM200B | 0.018349 | 1.15974 | 1.025358 | 1.311733 |
| GGT5 | 0.018396 | 1.184019 | 1.028896 | 1.362529 |
| KLHDC8A | 0.018408 | 1.196322 | 1.030676 | 1.38859 |
| COL24A1 | 0.018412 | 1.141644 | 1.022576 | 1.274575 |
| ALAS2 | 0.018435 | 1.07966 | 1.012988 | 1.15072 |
| ANKRD42 | 0.018437 | 0.6298 | 0.428755 | 0.925116 |
| CALM2 | 0.018517 | 1.535802 | 1.074644 | 2.194854 |
| MFGE8 | 0.018563 | 1.246573 | 1.037598 | 1.497637 |
| CD97 | 0.018568 | 0.744775 | 0.582741 | 0.951862 |
| OR2M5 | 0.018573 | 1.071697 | 1.011658 | 1.1353 |
| CFH | 0.018601 | 1.169829 | 1.026572 | 1.333076 |
| TCEB3CL2 | 0.018634 | 1.070144 | 1.011383 | 1.13232 |
| IKBIP | 0.018639 | 1.357182 | 1.052302 | 1.750394 |
| PREX2 | 0.018671 | 1.182364 | 1.028318 | 1.359487 |
| AP3D1 | 0.018718 | 0.584285 | 0.373315 | 0.914481 |
| TGFBR1 | 0.018727 | 1.315852 | 1.046704 | 1.654209 |
| OR5K2 | 0.018754 | 1.042605 | 1.006955 | 1.079518 |
| DFFA | 0.018761 | 0.646566 | 0.449444 | 0.930144 |
| C8orf48 | 0.018766 | 1.156237 | 1.024395 | 1.305048 |
| TDRP | 0.018819 | 1.151764 | 1.023681 | 1.295872 |
| GPR4 | 0.018822 | 1.273 | 1.04079 | 1.557018 |
| PTH1R | 0.018851 | 1.141024 | 1.022064 | 1.27383 |
| TNPO2 | 0.018858 | 0.646482 | 0.449203 | 0.930402 |
| STOM | 0.018873 | 1.242645 | 1.036556 | 1.489708 |
| PI15 | 0.018878 | 1.092209 | 1.014681 | 1.175662 |
| BEX2 | 0.018895 | 1.070125 | 1.011254 | 1.132424 |
| OTX2 | 0.018903 | 1.044239 | 1.007171 | 1.082672 |
| HP | 0.018916 | 1.070557 | 1.011311 | 1.133274 |
| DDX24 | 0.01896 | 1.767719 | 1.098347 | 2.84503 |
| ADAM18 | 0.018969 | 1.046306 | 1.007479 | 1.08663 |
| MAGEE2 | 0.018987 | 1.04517 | 1.007293 | 1.084471 |
| CCDC64B | 0.018992 | 0.859651 | 0.757608 | 0.975438 |
| LAMB1 | 0.018995 | 1.273269 | 1.040521 | 1.558079 |
| NAV3 | 0.019043 | 1.128682 | 1.020061 | 1.24887 |
| SLCO4A1 | 0.01908 | 0.879027 | 0.789185 | 0.979098 |
| TEKT4 | 0.019103 | 1.052002 | 1.008332 | 1.097563 |
| RGAG1 | 0.019105 | 1.099265 | 1.015609 | 1.189813 |
| MMP2 | 0.019117 | 1.153515 | 1.023634 | 1.299876 |
| GPR1 | 0.019187 | 1.092938 | 1.014599 | 1.177327 |
| EWSR1 | 0.019189 | 0.51634 | 0.296949 | 0.897819 |
| ECSCR | 0.019201 | 1.216195 | 1.032415 | 1.432689 |
| FAT3 | 0.019221 | 1.093793 | 1.014706 | 1.179043 |
| CGB2 | 0.019242 | 1.038196 | 1.006117 | 1.071298 |
| JAZF1 | 0.019252 | 1.251856 | 1.037207 | 1.510927 |
| CAPN15 | 0.019273 | 0.759942 | 0.603854 | 0.956377 |
| CDA | 0.019279 | 1.109412 | 1.017009 | 1.210209 |
| EZH2 | 0.019283 | 0.796767 | 0.658701 | 0.963773 |
| ENTPD8 | 0.019353 | 0.931444 | 0.877624 | 0.988566 |
| SMARCD3 | 0.01937 | 1.196572 | 1.029465 | 1.390806 |
| CSNK1G2 | 0.019374 | 0.64361 | 0.444843 | 0.93119 |
| SCUBE2 | 0.019419 | 1.125597 | 1.019288 | 1.242995 |
| KAL1 | 0.019452 | 1.152589 | 1.023162 | 1.298387 |
| MAPKAPK5 | 0.019469 | 0.577873 | 0.364787 | 0.91543 |
| ABCA9 | 0.019512 | 1.114783 | 1.017629 | 1.221213 |
| POU6F2 | 0.019582 | 1.052481 | 1.008236 | 1.098669 |
| NPDC1 | 0.019612 | 0.846868 | 0.736533 | 0.973733 |
| MMP11 | 0.019659 | 1.114418 | 1.017464 | 1.22061 |
| CCDC80 | 0.019689 | 1.104033 | 1.015923 | 1.199786 |
| CD200 | 0.019717 | 1.224114 | 1.032763 | 1.450918 |
| MPDZ | 0.019728 | 1.150775 | 1.02263 | 1.294976 |
| TEK | 0.019749 | 1.204751 | 1.030099 | 1.409014 |
| PHYHIPL | 0.019795 | 1.073429 | 1.011322 | 1.13935 |
| IDE | 0.019832 | 0.682249 | 0.49457 | 0.941147 |
| C3orf36 | 0.019868 | 1.169984 | 1.025178 | 1.335243 |
| WDR17 | 0.019928 | 1.087242 | 1.013302 | 1.166577 |
| C1orf100 | 0.019945 | 0.943296 | 0.898044 | 0.990827 |
| CLGN | 0.019978 | 1.095212 | 1.01444 | 1.182416 |
| PROC | 0.020017 | 1.103887 | 1.015676 | 1.199758 |
| NUAK1 | 0.020085 | 1.255988 | 1.036412 | 1.522085 |
| TPRN | 0.020097 | 0.813358 | 0.683337 | 0.96812 |
| C9orf114 | 0.020098 | 0.654982 | 0.458437 | 0.935792 |
| BATF2 | 0.020157 | 0.868859 | 0.7717 | 0.97825 |
| DDR2 | 0.020197 | 1.136718 | 1.020212 | 1.266528 |
| IFNA4 | 0.020219 | 1.076601 | 1.011581 | 1.145799 |
| GLIS2 | 0.020219 | 1.202106 | 1.029133 | 1.404151 |
| ABCG1 | 0.020237 | 1.248759 | 1.035238 | 1.50632 |
| KRTAP24-1 | 0.020253 | 1.046891 | 1.007164 | 1.088185 |
| TNFAIP8L3 | 0.020257 | 1.146431 | 1.021513 | 1.286625 |
| TGFB1 | 0.020259 | 1.251686 | 1.035581 | 1.512888 |
| CRISPLD2 | 0.020262 | 1.185375 | 1.026835 | 1.368392 |
| LOXL4 | 0.020284 | 1.114359 | 1.016988 | 1.221053 |
| IRX6 | 0.020294 | 1.052433 | 1.007978 | 1.098848 |
| CERCAM | 0.0203 | 1.172559 | 1.025057 | 1.341285 |
| BMP1 | 0.02033 | 1.327204 | 1.044931 | 1.685729 |
| RBP4 | 0.020359 | 1.072211 | 1.010871 | 1.137274 |
| PLSCR4 | 0.020389 | 1.177695 | 1.025652 | 1.352275 |
| F13B | 0.020391 | 1.044927 | 1.006828 | 1.084468 |
| EDIL3 | 0.020458 | 1.208441 | 1.029664 | 1.418258 |
| SLC9A3R2 | 0.02047 | 1.312249 | 1.042828 | 1.651278 |
| NOX5 | 0.020503 | 1.104521 | 1.015437 | 1.201419 |
| SH3GLB2 | 0.020518 | 0.715861 | 0.539527 | 0.949825 |
| ADM | 0.020536 | 1.157906 | 1.022817 | 1.310836 |
| OR4S1 | 0.020568 | 1.060955 | 1.009134 | 1.115438 |
| LENG9 | 0.020576 | 0.804815 | 0.669697 | 0.967194 |
| MANEAL | 0.020581 | 0.860973 | 0.758512 | 0.977273 |
| CLDN9 | 0.020631 | 1.084943 | 1.012572 | 1.162487 |
| SMYD3 | 0.020646 | 1.345732 | 1.046524 | 1.730485 |
| ABL2 | 0.020685 | 1.46994 | 1.060664 | 2.037144 |
| TJP3 | 0.020687 | 0.861482 | 0.759261 | 0.977464 |
| TENM4 | 0.020713 | 1.132559 | 1.019189 | 1.258539 |
| SH3BP2 | 0.020736 | 0.680381 | 0.490926 | 0.942948 |
| RGS1 | 0.020777 | 1.159698 | 1.022817 | 1.314899 |
| NOTCH1 | 0.020807 | 0.789067 | 0.645467 | 0.964615 |
| AKAP3 | 0.02082 | 0.855159 | 0.748896 | 0.9765 |
| KIAA1324L | 0.020827 | 1.114369 | 1.016589 | 1.221555 |
| UGT1A1 | 0.020836 | 1.050551 | 1.007518 | 1.095423 |
| GPRASP2 | 0.020875 | 1.20996 | 1.029318 | 1.422304 |
| ENSG00000278685 | 0.020903 | 1.048885 | 1.007254 | 1.092238 |
| POLDIP3 | 0.020927 | 0.63333 | 0.429801 | 0.933238 |
| CECR5 | 0.020975 | 0.719429 | 0.543957 | 0.951507 |
| DHRS7 | 0.021021 | 1.329265 | 1.043812 | 1.692782 |
| FAM153A | 0.021037 | 1.093271 | 1.013515 | 1.179302 |
| AK4 | 0.021048 | 1.137814 | 1.019617 | 1.269713 |
| TCEANC2 | 0.021113 | 0.639614 | 0.437479 | 0.935145 |
| WDR83 | 0.021184 | 0.651164 | 0.452114 | 0.93785 |
| SEMA6A | 0.021193 | 1.127766 | 1.01814 | 1.249195 |
| SLC7A1 | 0.021209 | 0.758887 | 0.600147 | 0.959615 |
| EFEMP2 | 0.021215 | 1.190906 | 1.026441 | 1.381723 |
| PLAT | 0.021233 | 1.247414 | 1.033546 | 1.505537 |
| TIGD6 | 0.021243 | 1.586677 | 1.071297 | 2.349996 |
| SART3 | 0.021264 | 0.520148 | 0.29824 | 0.90717 |
| GADD45B | 0.021302 | 1.215485 | 1.029463 | 1.43512 |
| MAP1LC3C | 0.021303 | 1.087242 | 1.012524 | 1.167473 |
| CTNNA3 | 0.021327 | 1.067299 | 1.009728 | 1.128153 |
| IL1RAP | 0.021333 | 1.247336 | 1.033386 | 1.505582 |
| USP17L10 | 0.021345 | 1.103895 | 1.014789 | 1.200825 |
| THBS2 | 0.021363 | 1.120429 | 1.017018 | 1.234354 |
| CRB3 | 0.021372 | 0.828345 | 0.705594 | 0.972451 |
| HABP2 | 0.02141 | 1.064342 | 1.009277 | 1.12241 |
| HIF1AN | 0.021415 | 0.667463 | 0.472988 | 0.941899 |
| RBBP4 | 0.02143 | 0.621531 | 0.414463 | 0.932052 |
| CD82 | 0.021442 | 0.811324 | 0.678918 | 0.969553 |
| TIE1 | 0.021447 | 1.224788 | 1.030433 | 1.455801 |
| GMEB1 | 0.021469 | 0.595958 | 0.383383 | 0.926399 |
| RTL1 | 0.021475 | 1.050473 | 1.007297 | 1.095498 |
| IGFBP5 | 0.021482 | 1.149717 | 1.020809 | 1.294905 |
| CASZ1 | 0.021483 | 0.801276 | 0.663387 | 0.967825 |
| HBD | 0.021499 | 1.057742 | 1.008315 | 1.109591 |
| NAP1L2 | 0.021523 | 1.086515 | 1.012302 | 1.16617 |
| PLEKHS1 | 0.021525 | 0.916511 | 0.850851 | 0.987237 |
| GREM1 | 0.021551 | 1.097484 | 1.013784 | 1.188095 |
| COL1A1 | 0.021557 | 1.140679 | 1.019554 | 1.276194 |
| CXorf49B | 0.021591 | 1.04927 | 1.00709 | 1.093215 |
| AXIN2 | 0.021598 | 0.887645 | 0.801826 | 0.982648 |
| AKAP17A | 0.021669 | 0.698185 | 0.513789 | 0.948761 |
| FEN1 | 0.021687 | 0.772643 | 0.619933 | 0.962971 |
| CNN3 | 0.021698 | 1.254821 | 1.033747 | 1.523174 |
| OR4K17 | 0.021722 | 1.070579 | 1.010011 | 1.13478 |
| CSK | 0.02173 | 0.724107 | 0.549636 | 0.95396 |
| C9orf172 | 0.021799 | 0.803167 | 0.66599 | 0.968599 |
| SLC7A3 | 0.021827 | 1.056172 | 1.007977 | 1.106671 |
| PCDHAC2 | 0.021835 | 1.077474 | 1.010903 | 1.148429 |
| AGT | 0.021836 | 1.117284 | 1.016246 | 1.228368 |
| OR6N1 | 0.021868 | 1.054932 | 1.00779 | 1.104278 |
| TBCEL | 0.021913 | 1.229635 | 1.03039 | 1.467408 |
| FGD6 | 0.021944 | 1.269235 | 1.035081 | 1.55636 |
| CCDC177 | 0.021949 | 1.055401 | 1.007827 | 1.10522 |
| EDNRB | 0.022002 | 1.183827 | 1.02464 | 1.367744 |
| CADM2 | 0.022017 | 1.06568 | 1.009212 | 1.125308 |
| AES | 0.022064 | 0.694786 | 0.508688 | 0.948966 |
| EXOC6B | 0.022082 | 1.438505 | 1.053651 | 1.96393 |
| FBXL2 | 0.022105 | 1.197162 | 1.026175 | 1.396641 |
| TUBB4A | 0.022142 | 1.100631 | 1.013839 | 1.194853 |
| GOT1L1 | 0.022146 | 1.04007 | 1.005647 | 1.075671 |
| TUBB6 | 0.022168 | 1.177734 | 1.023699 | 1.354946 |
| UNC119B | 0.022204 | 0.734673 | 0.564065 | 0.956883 |
| DNPEP | 0.022248 | 0.652171 | 0.452072 | 0.940841 |
| OR51H1 | 0.02227 | 1.070683 | 1.009781 | 1.135258 |
| LRRC29 | 0.022287 | 1.271647 | 1.034816 | 1.562679 |
| COX10 | 0.022339 | 0.664846 | 0.468413 | 0.943654 |
| SMYD4 | 0.022348 | 0.639496 | 0.435764 | 0.938479 |
| KDELC1 | 0.022352 | 1.285314 | 1.036285 | 1.594187 |
| MASP1 | 0.022354 | 1.112316 | 1.015228 | 1.218687 |
| RP11-162P23.2 | 0.022408 | 0.959061 | 0.92526 | 0.994097 |
| OR2T12 | 0.022457 | 1.069012 | 1.009476 | 1.132059 |
| CPA3 | 0.022481 | 1.110467 | 1.014902 | 1.215031 |
| SPIRE1 | 0.022492 | 1.177992 | 1.023383 | 1.355959 |
| ARRDC1 | 0.022539 | 0.756599 | 0.595371 | 0.961488 |
| ACTA2 | 0.02257 | 1.11768 | 1.015766 | 1.229819 |
| MTCP1 | 0.022596 | 0.763819 | 0.605918 | 0.962869 |
| SFRP4 | 0.022605 | 1.075522 | 1.010273 | 1.144984 |
| TPST1 | 0.022613 | 1.209015 | 1.026994 | 1.423297 |
| LGALS12 | 0.022628 | 1.095252 | 1.012841 | 1.184367 |
| SZT2 | 0.022653 | 0.695414 | 0.508845 | 0.950389 |
| MTF1 | 0.022668 | 0.658928 | 0.460293 | 0.94328 |
| RELN | 0.022725 | 1.08123 | 1.010964 | 1.156379 |
| BEND5 | 0.022761 | 1.11276 | 1.015005 | 1.219929 |
| FAM111A | 0.022764 | 0.746515 | 0.580459 | 0.960074 |
| HMG20B | 0.022781 | 0.677811 | 0.484995 | 0.947282 |
| P3H3 | 0.022845 | 1.179851 | 1.023232 | 1.360444 |
| ASCL2 | 0.02287 | 0.924262 | 0.863644 | 0.989135 |
| AP1M1 | 0.02289 | 1.450334 | 1.052872 | 1.997838 |
| COLEC12 | 0.022902 | 1.124117 | 1.016336 | 1.243327 |
| PRR9 | 0.022908 | 1.034718 | 1.004737 | 1.065594 |
| AK1 | 0.022915 | 0.817249 | 0.686817 | 0.972452 |
| KCNE4 | 0.022936 | 1.144194 | 1.018802 | 1.285019 |
| KRTAP5-7 | 0.022942 | 0.957729 | 0.922739 | 0.994047 |
| BSPRY | 0.022952 | 0.836831 | 0.717738 | 0.975685 |
| ZSCAN22 | 0.022966 | 0.668922 | 0.473006 | 0.945986 |
| TTF1 | 0.022969 | 0.629848 | 0.422857 | 0.938163 |
| C1orf123 | 0.022982 | 1.432429 | 1.050844 | 1.952576 |
| A2M | 0.022993 | 1.183977 | 1.023567 | 1.369527 |
| ZNF215 | 0.022995 | 1.126607 | 1.016577 | 1.248547 |
| PLVAP | 0.023005 | 1.258937 | 1.032252 | 1.535401 |
| DOHH | 0.02302 | 0.739326 | 0.569825 | 0.959248 |
| MAGED2 | 0.023022 | 1.347385 | 1.041926 | 1.742395 |
| ZNF528 | 0.023057 | 1.133038 | 1.017326 | 1.261911 |
| SYDE1 | 0.023096 | 1.20316 | 1.025715 | 1.411302 |
| LETM2 | 0.023108 | 1.246697 | 1.030716 | 1.507935 |
| MYCT1 | 0.023126 | 1.226661 | 1.028403 | 1.463139 |
| YARS | 0.023131 | 0.694807 | 0.507462 | 0.951318 |
| CD34 | 0.023135 | 1.236949 | 1.029569 | 1.4861 |
| AMOTL1 | 0.023155 | 1.161384 | 1.020695 | 1.321465 |
| CRTAC1 | 0.023228 | 1.072168 | 1.009554 | 1.138666 |
| KIAA1161 | 0.023269 | 0.804337 | 0.66643 | 0.970781 |
| SLC24A2 | 0.023301 | 1.091676 | 1.012 | 1.177625 |
| CBS | 0.023348 | 1.107571 | 1.013961 | 1.209822 |
| SIRPB2 | 0.02335 | 1.166046 | 1.021063 | 1.331615 |
| SHC4 | 0.023356 | 1.136972 | 1.017565 | 1.27039 |
| CALU | 0.023365 | 1.343594 | 1.04086 | 1.734378 |
| ANGPT2 | 0.023385 | 1.197039 | 1.024664 | 1.398414 |
| MAP7D3 | 0.023389 | 1.187152 | 1.023509 | 1.376958 |
| SRRM1 | 0.023414 | 0.704095 | 0.519848 | 0.953644 |
| ZNF18 | 0.023416 | 0.725445 | 0.549625 | 0.957509 |
| PODN | 0.023443 | 1.11749 | 1.015121 | 1.230181 |
| LIMD1 | 0.023455 | 0.700646 | 0.515063 | 0.953098 |
| CDR1 | 0.023466 | 1.061802 | 1.008127 | 1.118336 |
| FBLN5 | 0.023476 | 1.156404 | 1.019797 | 1.311311 |
| CEP295 | 0.023538 | 0.753797 | 0.590229 | 0.962696 |
| AMELY | 0.023544 | 1.050082 | 1.006593 | 1.09545 |
| TOP3B | 0.023551 | 0.839106 | 0.7209 | 0.976694 |
| PARVA | 0.023564 | 1.24059 | 1.029389 | 1.495124 |
| ENSG00000281079 | 0.023577 | 0.97075 | 0.94612 | 0.996022 |
| ENSG00000279081 | 0.023636 | 1.085527 | 1.01105 | 1.165491 |
| DCLRE1B | 0.02365 | 0.684188 | 0.492502 | 0.95048 |
| PPP1R10 | 0.023694 | 0.635086 | 0.428545 | 0.941172 |
| MGP | 0.023717 | 1.106344 | 1.013573 | 1.207605 |
| RHOBTB3 | 0.023734 | 1.167967 | 1.020913 | 1.336204 |
| SIGIRR | 0.023749 | 0.720717 | 0.542596 | 0.957312 |
| DENND5A | 0.023764 | 1.221152 | 1.026952 | 1.452077 |
| FAM126A | 0.023785 | 1.212478 | 1.025953 | 1.432916 |
| C16orf62 | 0.023812 | 1.240932 | 1.029086 | 1.49639 |
| GPR116 | 0.023838 | 1.24613 | 1.02962 | 1.508168 |
| GPATCH3 | 0.023967 | 0.656609 | 0.455725 | 0.946042 |
| FOXP2 | 0.023973 | 1.087302 | 1.011095 | 1.169254 |
| DPYSL3 | 0.024001 | 1.115658 | 1.014512 | 1.226887 |
| PDE3A | 0.024025 | 1.129866 | 1.016186 | 1.256263 |
| F13A1 | 0.024052 | 1.098832 | 1.012455 | 1.192578 |
| SYT16 | 0.024069 | 1.081442 | 1.010328 | 1.157562 |
| TPPP2 | 0.024075 | 1.047615 | 1.006121 | 1.09082 |
| TCTEX1D1 | 0.024075 | 1.18895 | 1.022965 | 1.381866 |
| MAP6 | 0.024089 | 1.120102 | 1.014981 | 1.23611 |
| PRR5 | 0.024097 | 0.795749 | 0.652463 | 0.970501 |
| PKD2 | 0.024109 | 1.218625 | 1.026237 | 1.447079 |
| TMC6 | 0.024127 | 0.770876 | 0.614837 | 0.966516 |
| KRT85 | 0.024143 | 1.047833 | 1.006129 | 1.091265 |
| FAM20A | 0.024151 | 1.162764 | 1.019909 | 1.325627 |
| ESRRA | 0.024175 | 0.7669 | 0.608875 | 0.965937 |
| CHST9 | 0.024212 | 1.051591 | 1.006579 | 1.098615 |
| MBOAT4 | 0.024227 | 1.142935 | 1.017555 | 1.283763 |
| OR6K3 | 0.024273 | 1.051211 | 1.006513 | 1.097894 |
| RNF180 | 0.024314 | 1.145206 | 1.017745 | 1.28863 |
| KLHDC7A | 0.024326 | 0.924727 | 0.863841 | 0.989905 |
| ZC3HAV1 | 0.024367 | 0.624716 | 0.414768 | 0.940936 |
| ZNF783 | 0.024402 | 0.701008 | 0.51449 | 0.955143 |
| ENSG00000269476 | 0.024417 | 0.957717 | 0.922352 | 0.994438 |
| OR2M2 | 0.024417 | 1.057829 | 1.007284 | 1.11091 |
| CLDN10 | 0.02442 | 1.049684 | 1.006279 | 1.094962 |
| DNM2 | 0.02442 | 0.707876 | 0.523941 | 0.956383 |
| ZNF786 | 0.024424 | 0.707998 | 0.524105 | 0.956412 |
| TPP1 | 0.024461 | 1.465301 | 1.050454 | 2.04398 |
| ADH4 | 0.024483 | 1.053901 | 1.006779 | 1.103228 |
| FZR1 | 0.024548 | 0.651456 | 0.448382 | 0.946501 |
| PECAM1 | 0.024563 | 1.223516 | 1.026201 | 1.458772 |
| H1FOO | 0.024566 | 1.044546 | 1.005603 | 1.084997 |
| TSEN54 | 0.024571 | 0.736767 | 0.564498 | 0.961606 |
| INTS2 | 0.024669 | 0.709014 | 0.525243 | 0.957081 |
| COL5A1 | 0.024672 | 1.164917 | 1.019661 | 1.330867 |
| GIPC3 | 0.024677 | 1.276914 | 1.031663 | 1.580468 |
| ARHGAP28 | 0.024689 | 1.146401 | 1.017565 | 1.29155 |
| SHOX2 | 0.024739 | 1.107612 | 1.01308 | 1.210966 |
| FGG | 0.024779 | 1.034542 | 1.004319 | 1.065675 |
| FZD2 | 0.024799 | 1.175489 | 1.02071 | 1.353738 |
| TSNARE1 | 0.024831 | 0.771423 | 0.614967 | 0.967683 |
| CAST | 0.024865 | 1.366294 | 1.040232 | 1.79456 |
| ALB | 0.024894 | 1.046687 | 1.005775 | 1.089263 |
| IFNA16 | 0.024909 | 1.070869 | 1.008672 | 1.136901 |
| PRICKLE4 | 0.024912 | 0.891408 | 0.80621 | 0.98561 |
| MLEC | 0.024944 | 0.747898 | 0.580188 | 0.964088 |
| NPR3 | 0.024954 | 1.10348 | 1.012468 | 1.202673 |
| SLC16A7 | 0.024987 | 1.131525 | 1.015646 | 1.260625 |
| ISLR | 0.025031 | 1.120334 | 1.014348 | 1.237395 |
| PDLIM3 | 0.025066 | 1.106877 | 1.012791 | 1.209704 |
| SAC3D1 | 0.025106 | 0.778218 | 0.624895 | 0.969161 |
| ENOX1 | 0.025124 | 1.147162 | 1.017284 | 1.293621 |
| EFNA3 | 0.025183 | 0.892766 | 0.808364 | 0.985981 |
| LRIT3 | 0.025193 | 0.92097 | 0.856917 | 0.989811 |
| GPNMB | 0.025199 | 1.149118 | 1.017437 | 1.297841 |
| PROK2 | 0.025239 | 1.080538 | 1.009661 | 1.15639 |
| CCDC185 | 0.025254 | 1.046223 | 1.005621 | 1.088465 |
| MRI1 | 0.025261 | 0.785426 | 0.635648 | 0.970496 |
| FEZ1 | 0.025264 | 1.174987 | 1.020193 | 1.353267 |
| LPHN1 | 0.025274 | 0.823475 | 0.694629 | 0.97622 |
| VN1R4 | 0.025276 | 1.046568 | 1.005656 | 1.089145 |
| RTP1 | 0.025279 | 1.042226 | 1.005137 | 1.080683 |
| NTMT1 | 0.025282 | 0.724871 | 0.546803 | 0.960928 |
| LZTS1 | 0.025347 | 1.176497 | 1.020274 | 1.356641 |
| SMIM10 | 0.025356 | 1.15459 | 1.0179 | 1.309635 |
| PROCR | 0.025373 | 1.183567 | 1.021001 | 1.372018 |
| SLX4 | 0.025427 | 0.699345 | 0.511077 | 0.956967 |
| KCNJ11 | 0.025476 | 0.855818 | 0.746551 | 0.981076 |
| GRIA3 | 0.025501 | 1.127044 | 1.014766 | 1.251745 |
| PALM2-AKAP2 | 0.025585 | 1.135601 | 1.015642 | 1.269728 |
| COL11A1 | 0.02564 | 1.073902 | 1.008717 | 1.143299 |
| SLC12A5 | 0.025641 | 1.141962 | 1.01629 | 1.283174 |
| DST | 0.025644 | 1.164915 | 1.018752 | 1.332048 |
| TMEM35 | 0.025804 | 1.066475 | 1.007802 | 1.128564 |
| PBX4 | 0.025863 | 0.821095 | 0.69039 | 0.976545 |
| DEXI | 0.025865 | 1.389339 | 1.040384 | 1.855338 |
| OR6A2 | 0.025911 | 1.042589 | 1.005023 | 1.08156 |
| CYYR1 | 0.025964 | 1.217595 | 1.023868 | 1.447977 |
| ST6GALNAC4 | 0.02603 | 0.803922 | 0.663357 | 0.974272 |
| CCDC140 | 0.026051 | 1.047022 | 1.005497 | 1.090263 |
| SPANXN3 | 0.026085 | 1.042223 | 1.004937 | 1.080891 |
| SPANXB2 | 0.026103 | 1.032736 | 1.00384 | 1.062464 |
| BMPR2 | 0.026121 | 1.406451 | 1.041381 | 1.899501 |
| RADIL | 0.026126 | 1.125278 | 1.014127 | 1.248611 |
| ADAM7 | 0.02615 | 1.045883 | 1.00534 | 1.088061 |
| FRMD8 | 0.026283 | 0.722234 | 0.542028 | 0.962352 |
| TUB | 0.026332 | 1.108824 | 1.012226 | 1.214641 |
| SEC16A | 0.026342 | 0.714877 | 0.531622 | 0.961303 |
| C5AR1 | 0.026355 | 1.173871 | 1.019016 | 1.352259 |
| SNAI1 | 0.026413 | 1.211012 | 1.022686 | 1.434019 |
| PDE8B | 0.026417 | 1.207287 | 1.022312 | 1.425731 |
| LRRC16B | 0.026487 | 0.852334 | 0.740149 | 0.981522 |
| CNTNAP1 | 0.026624 | 1.164186 | 1.017781 | 1.331651 |
| IGFBP6 | 0.026624 | 1.154123 | 1.016757 | 1.310047 |
| ARHGAP20 | 0.026644 | 1.135727 | 1.01485 | 1.271002 |
| ADH1B | 0.026652 | 1.062176 | 1.007008 | 1.120367 |
| IRAK1 | 0.026657 | 0.760226 | 0.596576 | 0.968768 |
| HCFC2 | 0.026724 | 1.284194 | 1.029272 | 1.602252 |
| COL14A1 | 0.026762 | 1.111679 | 1.012264 | 1.220858 |
| QTRT1 | 0.026818 | 0.731431 | 0.554548 | 0.964732 |
| PCDH7 | 0.026919 | 1.112538 | 1.012255 | 1.222755 |
| THSD7B | 0.026953 | 1.07648 | 1.008438 | 1.149112 |
| SHISA2 | 0.026996 | 1.115461 | 1.012509 | 1.22888 |
| TRPC3 | 0.027012 | 1.178633 | 1.018859 | 1.363463 |
| ADAMTS16 | 0.027021 | 1.107126 | 1.011631 | 1.211636 |
| GLIPR1 | 0.027064 | 1.163101 | 1.017277 | 1.329828 |
| ANTXR2 | 0.027069 | 1.166802 | 1.017639 | 1.337828 |
| MEDAG | 0.027093 | 1.122708 | 1.01319 | 1.244065 |
| TGFB2 | 0.027142 | 1.151314 | 1.016039 | 1.3046 |
| SYCE2 | 0.027155 | 0.84058 | 0.720558 | 0.980593 |
| PCDH17 | 0.027175 | 1.215732 | 1.022266 | 1.445812 |
| TMEM14C | 0.027257 | 1.420551 | 1.040195 | 1.939986 |
| BDH2 | 0.02726 | 1.268384 | 1.027045 | 1.566433 |
| FOXO4 | 0.027273 | 0.778283 | 0.622997 | 0.972274 |
| LRRC4C | 0.027317 | 1.089607 | 1.009651 | 1.175895 |
| PPAN | 0.027327 | 0.754037 | 0.586814 | 0.968914 |
| TXLNA | 0.027329 | 0.616981 | 0.401791 | 0.947421 |
| CNIH4 | 0.027337 | 1.461706 | 1.043353 | 2.047805 |
| KLHL13 | 0.027356 | 1.109524 | 1.011676 | 1.216835 |
| TTC16 | 0.027461 | 0.908041 | 0.833423 | 0.989341 |
| SMIM3 | 0.027461 | 1.196027 | 1.020084 | 1.402316 |
| SYT12 | 0.027482 | 1.093603 | 1.009979 | 1.184151 |
| OR5D18 | 0.027497 | 1.068331 | 1.007356 | 1.132997 |
| CNGA2 | 0.027548 | 1.061251 | 1.006596 | 1.118873 |
| GMIP | 0.027556 | 0.751468 | 0.582826 | 0.968908 |
| DISC1 | 0.027581 | 1.260907 | 1.025924 | 1.549713 |
| CORO7-PAM16 | 0.027616 | 0.95125 | 0.909874 | 0.994508 |
| ZNF775 | 0.027621 | 0.780708 | 0.626357 | 0.973095 |
| SLC24A3 | 0.02763 | 1.138842 | 1.01442 | 1.278525 |
| HDAC8 | 0.027641 | 0.625093 | 0.411479 | 0.949603 |
| AGMAT | 0.027669 | 0.870653 | 0.769664 | 0.984893 |
| DNASE1L2 | 0.027724 | 0.888616 | 0.799923 | 0.987143 |
| GPR87 | 0.027759 | 1.049667 | 1.005316 | 1.095974 |
| TNFAIP1 | 0.027783 | 1.567991 | 1.050365 | 2.340707 |
| BCAT1 | 0.027852 | 1.139472 | 1.014313 | 1.280075 |
| ISM1 | 0.027906 | 1.093056 | 1.009704 | 1.183288 |
| ADTRP | 0.027918 | 1.083923 | 1.00878 | 1.164664 |
| PDGFC | 0.028011 | 1.211178 | 1.020897 | 1.436924 |
| SRI | 0.028011 | 1.296469 | 1.028423 | 1.634378 |
| ZNF589 | 0.028013 | 0.756338 | 0.589553 | 0.970307 |
| GAS1 | 0.028015 | 1.096297 | 1.009972 | 1.190001 |
| C16orf90 | 0.028067 | 0.962197 | 0.929671 | 0.995861 |
| MMACHC | 0.028082 | 0.71822 | 0.534531 | 0.965033 |
| CYP7B1 | 0.028084 | 1.130691 | 1.013296 | 1.261687 |
| CYTH3 | 0.028094 | 1.296421 | 1.028294 | 1.634462 |
| MAP3K7CL | 0.028119 | 1.294308 | 1.028075 | 1.629486 |
| HNRNPL | 0.028123 | 0.566806 | 0.34145 | 0.940896 |
| TUBA1A | 0.028127 | 1.154512 | 1.015534 | 1.31251 |
| ARHGEF38 | 0.028161 | 0.878667 | 0.782826 | 0.986243 |
| ASB6 | 0.028184 | 0.639639 | 0.429168 | 0.953328 |
| TFIP11 | 0.028193 | 0.638091 | 0.427195 | 0.953102 |
| MEF2BNB-MEF2B | 0.028259 | 0.838868 | 0.716997 | 0.981455 |
| SLC25A21 | 0.02826 | 1.090459 | 1.009268 | 1.178181 |
| EMCN | 0.028265 | 1.182828 | 1.018044 | 1.374285 |
| D2HGDH | 0.028276 | 0.756351 | 0.589326 | 0.970714 |
| BPI | 0.02833 | 1.112467 | 1.011376 | 1.223662 |
| PRSS35 | 0.028395 | 1.102632 | 1.010387 | 1.203299 |
| RMDN2 | 0.028439 | 1.182478 | 1.017844 | 1.373742 |
| MBTPS2 | 0.028444 | 1.427151 | 1.038235 | 1.961751 |
| FFAR3 | 0.028452 | 1.06392 | 1.006555 | 1.124554 |
| SPESP1 | 0.028458 | 1.103711 | 1.010456 | 1.205572 |
| GRID1 | 0.028485 | 1.120684 | 1.012065 | 1.240959 |
| ENSG00000269533 | 0.028516 | 1.092955 | 1.009384 | 1.183445 |
| ATXN3L | 0.02856 | 1.068706 | 1.006991 | 1.134205 |
| SIK1 | 0.02857 | 1.100905 | 1.010124 | 1.199845 |
| BAI3 | 0.028576 | 1.080844 | 1.008176 | 1.15875 |
| NACC1 | 0.028589 | 0.714458 | 0.528735 | 0.965417 |
| COL8A1 | 0.028609 | 1.114047 | 1.011357 | 1.227164 |
| PCDHGB5 | 0.028609 | 1.122856 | 1.01219 | 1.245621 |
| GSR | 0.02863 | 0.769822 | 0.609041 | 0.973048 |
| GLI1 | 0.028645 | 1.128884 | 1.012732 | 1.258358 |
| COX15 | 0.028648 | 0.67977 | 0.481077 | 0.960525 |
| NELFB | 0.028666 | 0.678391 | 0.47921 | 0.960359 |
| MRGPRX2 | 0.028698 | 1.041217 | 1.004212 | 1.079586 |
| FBXO48 | 0.028703 | 0.724512 | 0.542815 | 0.96703 |
| LRRC6 | 0.028719 | 1.138091 | 1.013536 | 1.277954 |
| FGF10 | 0.028731 | 1.077396 | 1.007774 | 1.151829 |
| DTNA | 0.028742 | 1.095092 | 1.009475 | 1.187971 |
| ANGPTL2 | 0.028812 | 1.149931 | 1.014553 | 1.303373 |
| POTEI | 0.028864 | 1.185321 | 1.017688 | 1.380565 |
| APBB1 | 0.028883 | 1.15101 | 1.014595 | 1.305766 |
| FJX1 | 0.028889 | 1.222321 | 1.02089 | 1.463496 |
| MUC13 | 0.028923 | 0.916187 | 0.846987 | 0.991042 |
| FLT1 | 0.02894 | 1.269569 | 1.024816 | 1.572774 |
| TSHZ3 | 0.028953 | 1.163892 | 1.015698 | 1.333708 |
| HSPB8 | 0.02897 | 1.093548 | 1.009212 | 1.184932 |
| CFHR3 | 0.028973 | 1.053675 | 1.005374 | 1.104296 |
| VSTM4 | 0.029041 | 1.14318 | 1.013761 | 1.289121 |
| C20orf194 | 0.029072 | 1.171573 | 1.016277 | 1.3506 |
| SOCS3 | 0.029072 | 1.22078 | 1.02055 | 1.460295 |
| TBC1D31 | 0.029099 | 0.75605 | 0.58812 | 0.971929 |
| HAND1 | 0.029107 | 1.038229 | 1.003825 | 1.073811 |
| ZRSR1 | 0.029163 | 0.928121 | 0.867952 | 0.99246 |
| GGA1 | 0.029179 | 0.611693 | 0.393284 | 0.951395 |
| MAST3 | 0.029201 | 0.767095 | 0.604445 | 0.973512 |
| PODNL1 | 0.029231 | 1.146865 | 1.013948 | 1.297205 |
| BOC | 0.029244 | 1.099672 | 1.009643 | 1.197728 |
| CLIP3 | 0.029255 | 1.13127 | 1.012529 | 1.263935 |
| GNB4 | 0.029263 | 1.191502 | 1.017837 | 1.394798 |
| ZNF157 | 0.029278 | 1.0571 | 1.005614 | 1.111221 |
| ZEB1 | 0.029293 | 1.155659 | 1.014681 | 1.316225 |
| MYH4 | 0.029302 | 1.044249 | 1.004369 | 1.085712 |
| C8orf82 | 0.029304 | 0.777736 | 0.620375 | 0.975011 |
| PCDHA10 | 0.029336 | 1.088178 | 1.008528 | 1.174117 |
| TTC9C | 0.029347 | 0.631112 | 0.417148 | 0.954824 |
| TMEM201 | 0.029362 | 0.750977 | 0.580409 | 0.97167 |
| LANCL2 | 0.029375 | 1.236673 | 1.02153 | 1.497127 |
| SPOCK1 | 0.029424 | 1.089324 | 1.008593 | 1.176516 |
| EHD2 | 0.029444 | 1.171807 | 1.015964 | 1.351555 |
| RBM28 | 0.029469 | 0.671676 | 0.46942 | 0.961076 |
| ADCYAP1 | 0.029488 | 1.068304 | 1.006606 | 1.133783 |
| CD200R1L | 0.0295 | 1.040776 | 1.003988 | 1.078912 |
| PRKG1 | 0.029552 | 1.155495 | 1.014455 | 1.316143 |
| SPARCL1 | 0.029563 | 1.113865 | 1.010759 | 1.227489 |
| DACT1 | 0.029578 | 1.141895 | 1.013243 | 1.286881 |
| CASC10 | 0.029586 | 1.188318 | 1.017248 | 1.388156 |
| RPRM | 0.029632 | 1.051924 | 1.005017 | 1.10102 |
| VEGFB | 0.029669 | 1.278595 | 1.024542 | 1.595645 |
| SUMO3 | 0.029679 | 1.400062 | 1.033736 | 1.896203 |
| LONP1 | 0.02968 | 0.675876 | 0.474797 | 0.962113 |
| TTC37 | 0.029681 | 1.374493 | 1.031856 | 1.830906 |
| BCL6B | 0.029691 | 1.222367 | 1.019981 | 1.464912 |
| PABPC4L | 0.0297 | 1.144621 | 1.013391 | 1.292843 |
| FLT4 | 0.029726 | 1.23547 | 1.021012 | 1.494975 |
| MEF2A | 0.029729 | 1.2892 | 1.02529 | 1.62104 |
| TCTN1 | 0.029754 | 1.252496 | 1.022351 | 1.53445 |
| HMCN1 | 0.029764 | 1.118204 | 1.011023 | 1.236747 |
| IQCB1 | 0.029772 | 0.71247 | 0.524776 | 0.967295 |
| FAM25A | 0.02978 | 1.028231 | 1.002733 | 1.054377 |
| LRRC70 | 0.029786 | 1.201334 | 1.01814 | 1.417489 |
| SRPX | 0.029797 | 1.11181 | 1.010435 | 1.223355 |
| POTEB3 | 0.02985 | 1.069 | 1.006537 | 1.135339 |
| CALD1 | 0.029919 | 1.12103 | 1.011175 | 1.242819 |
| RNF220 | 0.029926 | 0.594654 | 0.371945 | 0.950714 |
| TRABD | 0.029931 | 0.753086 | 0.58299 | 0.972811 |
| OLFML3 | 0.029943 | 1.14614 | 1.013338 | 1.296345 |
| COL8A2 | 0.029944 | 1.138019 | 1.012638 | 1.278924 |
| ACOX2 | 0.029945 | 1.127565 | 1.01173 | 1.256662 |
| ADPRH | 0.030002 | 1.240919 | 1.021118 | 1.508034 |
| RPGRIP1L | 0.030018 | 1.40989 | 1.033787 | 1.922825 |
| APOA1 | 0.030071 | 1.051613 | 1.004865 | 1.100536 |
| NHS | 0.0301 | 1.180653 | 1.016118 | 1.371831 |
| TTR | 0.030105 | 1.047653 | 1.004491 | 1.092669 |
| RPS6KA6 | 0.030106 | 1.081185 | 1.007541 | 1.160212 |
| HHIP | 0.030146 | 1.086576 | 1.008005 | 1.171272 |
| DUSP14 | 0.030158 | 1.326806 | 1.027506 | 1.713287 |
| CNTROB | 0.030218 | 0.650024 | 0.440301 | 0.959642 |
| SLC1A4 | 0.030271 | 0.781248 | 0.624884 | 0.976738 |
| ATXN7L3 | 0.030321 | 0.66566 | 0.460584 | 0.962048 |
| TCEAL5 | 0.030358 | 1.042583 | 1.003964 | 1.082687 |
| MTX3 | 0.030367 | 1.231293 | 1.019925 | 1.486465 |
| VPS36 | 0.030401 | 0.694842 | 0.499729 | 0.966135 |
| CFDP1 | 0.030401 | 1.422933 | 1.033941 | 1.958274 |
| CSH1 | 0.030438 | 1.052113 | 1.004809 | 1.101645 |
| NRP2 | 0.030456 | 1.151558 | 1.013401 | 1.308551 |
| ANGPTL4 | 0.030553 | 1.129591 | 1.011496 | 1.261473 |
| G0S2 | 0.030559 | 1.124524 | 1.011066 | 1.250713 |
| ATF3 | 0.030611 | 1.162482 | 1.014175 | 1.332476 |
| GRM1 | 0.030628 | 1.098997 | 1.008855 | 1.197193 |
| TCIRG1 | 0.030664 | 0.751162 | 0.579493 | 0.973685 |
| SUSD1 | 0.030665 | 1.323378 | 1.026456 | 1.706189 |
| KLF9 | 0.030725 | 1.174263 | 1.01503 | 1.358475 |
| SEMA5B | 0.030759 | 1.180759 | 1.01552 | 1.372885 |
| MB21D1 | 0.030761 | 0.805622 | 0.662159 | 0.980169 |
| SPON1 | 0.030766 | 1.097674 | 1.008671 | 1.19453 |
| SLC25A35 | 0.030792 | 0.795881 | 0.646947 | 0.979102 |
| OR2T1 | 0.030817 | 1.066153 | 1.005934 | 1.129977 |
| PDZRN4 | 0.030821 | 1.062855 | 1.005645 | 1.123319 |
| PKNOX2 | 0.030847 | 1.116917 | 1.010247 | 1.234851 |
| GFAP | 0.030857 | 1.123543 | 1.010793 | 1.24887 |
| HNRNPK | 0.030861 | 0.52845 | 0.29616 | 0.942933 |
| STRIP1 | 0.030867 | 0.595585 | 0.372061 | 0.953396 |
| PRAF2 | 0.030892 | 1.228855 | 1.019132 | 1.481735 |
| ENG | 0.030897 | 1.254746 | 1.021083 | 1.541881 |
| C16orf52 | 0.030948 | 1.47297 | 1.036136 | 2.093973 |
| FCN1 | 0.030972 | 1.122397 | 1.010625 | 1.246532 |
| OR4A16 | 0.030996 | 1.072691 | 1.006434 | 1.14331 |
| ABCA7 | 0.031068 | 0.79893 | 0.651463 | 0.979777 |
| USF1 | 0.031134 | 0.652686 | 0.442799 | 0.962059 |
| ST6GALNAC5 | 0.031138 | 1.118946 | 1.010238 | 1.239352 |
| PLXNC1 | 0.031175 | 1.18336 | 1.015341 | 1.379183 |
| PDK4 | 0.031242 | 1.095921 | 1.008284 | 1.191175 |
| GFRA1 | 0.031312 | 1.076352 | 1.006621 | 1.150914 |
| CLTCL1 | 0.031342 | 1.176585 | 1.014666 | 1.364343 |
| ECSIT | 0.031416 | 0.710681 | 0.52068 | 0.970016 |
| MCFD2 | 0.031417 | 1.599616 | 1.04276 | 2.453845 |
| ATP2A1 | 0.03143 | 0.843522 | 0.722395 | 0.984959 |
| PDSS1 | 0.031468 | 0.815073 | 0.676525 | 0.981995 |
| FZD1 | 0.031474 | 1.222155 | 1.017979 | 1.467283 |
| MUTYH | 0.031508 | 0.713678 | 0.524796 | 0.970542 |
| EFNB3 | 0.031516 | 1.121864 | 1.01024 | 1.245821 |
| SGPP2 | 0.031561 | 0.880121 | 0.783402 | 0.988781 |
| GGN | 0.031657 | 1.254779 | 1.020138 | 1.54339 |
| PSG4 | 0.031685 | 1.044806 | 1.003851 | 1.087432 |
| KCNF1 | 0.031696 | 1.086713 | 1.007314 | 1.172371 |
| SBF1 | 0.031747 | 0.747246 | 0.572772 | 0.974868 |
| AC008686.1 | 0.031749 | 0.958602 | 0.922318 | 0.996314 |
| ATXN7 | 0.031749 | 0.677961 | 0.475504 | 0.96662 |
| CHAC1 | 0.031776 | 0.860468 | 0.750173 | 0.98698 |
| IER5L | 0.031779 | 0.846981 | 0.72784 | 0.985624 |
| RAX2 | 0.031822 | 1.046374 | 1.00395 | 1.090592 |
| OGN | 0.031873 | 1.064256 | 1.005413 | 1.126542 |
| GPR85 | 0.031919 | 1.217283 | 1.017142 | 1.456806 |
| ADARB1 | 0.031925 | 1.222937 | 1.017544 | 1.46979 |
| FAM213B | 0.031937 | 0.802783 | 0.656802 | 0.98121 |
| HECW2 | 0.031947 | 1.21449 | 1.01691 | 1.450457 |
| OR52A5 | 0.032003 | 1.045616 | 1.003843 | 1.089126 |
| PCYT1B | 0.03201 | 1.090242 | 1.007454 | 1.179833 |
| CLDND1 | 0.032044 | 1.472386 | 1.033742 | 2.097156 |
| RHOQ | 0.032054 | 1.309139 | 1.023361 | 1.674723 |
| CTLA4 | 0.032055 | 0.879311 | 0.78176 | 0.989035 |
| CHRNA7 | 0.032058 | 1.104635 | 1.008565 | 1.209856 |
| ADAMTS15 | 0.032101 | 1.116765 | 1.009484 | 1.235447 |
| CHST1 | 0.032137 | 1.175387 | 1.013877 | 1.362626 |
| COL15A1 | 0.032138 | 1.167571 | 1.0133 | 1.34533 |
| MAPK10 | 0.032195 | 1.123967 | 1.00998 | 1.250819 |
| ACKR1 | 0.032196 | 1.085186 | 1.006971 | 1.169475 |
| ERBB4 | 0.032374 | 1.069928 | 1.005696 | 1.138264 |
| BNIP3L | 0.032405 | 1.340731 | 1.024895 | 1.753897 |
| HEG1 | 0.032406 | 1.199143 | 1.015346 | 1.416212 |
| DCLK3 | 0.032448 | 1.178244 | 1.013813 | 1.369346 |
| PLA2G15 | 0.032486 | 1.388781 | 1.027782 | 1.876578 |
| KCNK2 | 0.032577 | 1.057544 | 1.004652 | 1.11322 |
| MREG | 0.032612 | 0.790169 | 0.636658 | 0.980696 |
| ZNF653 | 0.032626 | 0.688523 | 0.488924 | 0.969609 |
| OR56A1 | 0.032631 | 1.051822 | 1.004186 | 1.101717 |
| WHAMM | 0.032637 | 0.653545 | 0.4424 | 0.965462 |
| LRRC45 | 0.032709 | 0.787652 | 0.632698 | 0.980557 |
| TAF15 | 0.032717 | 0.662942 | 0.454599 | 0.966768 |
| STON1 | 0.032721 | 1.116575 | 1.009104 | 1.235491 |
| DNASE2 | 0.032761 | 0.715957 | 0.52683 | 0.972978 |
| RPS6KA4 | 0.032767 | 0.745391 | 0.56915 | 0.976206 |
| RRH | 0.032801 | 0.931351 | 0.872474 | 0.994201 |
| STRIP2 | 0.032823 | 0.857548 | 0.744674 | 0.98753 |
| TGFB3 | 0.032837 | 1.14735 | 1.011277 | 1.301732 |
| RCCD1 | 0.032916 | 0.758175 | 0.587893 | 0.977779 |
| SLC45A1 | 0.032942 | 1.15802 | 1.011959 | 1.325163 |
| MAP2 | 0.032954 | 1.134172 | 1.010246 | 1.2733 |
| C4orf48 | 0.033032 | 1.145088 | 1.010974 | 1.296993 |
| SUDS3 | 0.033041 | 0.619887 | 0.399343 | 0.962229 |
| SELE | 0.033058 | 1.109685 | 1.008405 | 1.221138 |
| PGAP2 | 0.033106 | 0.728223 | 0.543966 | 0.974895 |
| ARGLU1 | 0.033141 | 0.749243 | 0.574479 | 0.977172 |
| ST8SIA6 | 0.03327 | 1.091287 | 1.006953 | 1.182683 |
| C8B | 0.033297 | 1.039542 | 1.003075 | 1.077334 |
| PRMT2 | 0.033302 | 1.427004 | 1.028544 | 1.979828 |
| KLHL30 | 0.033346 | 1.122246 | 1.009144 | 1.248025 |
| PDPN | 0.03337 | 1.162563 | 1.011939 | 1.335606 |
| SUSD5 | 0.033371 | 1.108152 | 1.008124 | 1.218105 |
| MAU2 | 0.033433 | 0.63109 | 0.412922 | 0.964527 |
| GALNT13 | 0.033442 | 1.063284 | 1.004823 | 1.125146 |
| ADAMTS7 | 0.033443 | 1.207116 | 1.014869 | 1.435781 |
| LIN54 | 0.033448 | 0.663713 | 0.454899 | 0.968381 |
| SPC24 | 0.033455 | 0.800847 | 0.652613 | 0.982751 |
| COL6A2 | 0.033475 | 1.161739 | 1.011799 | 1.333899 |
| ZFP92 | 0.033483 | 1.137736 | 1.010142 | 1.281446 |
| MDFIC | 0.033532 | 1.190915 | 1.013712 | 1.399093 |
| RGS5 | 0.03354 | 1.161093 | 1.011704 | 1.332541 |
| ZNF692 | 0.033553 | 0.734248 | 0.552241 | 0.976242 |
| ENSG00000261833 | 0.03356 | 1.05779 | 1.004381 | 1.11404 |
| HDX | 0.033561 | 1.147689 | 1.010774 | 1.303149 |
| CCL14 | 0.033592 | 1.107655 | 1.007969 | 1.217199 |
| AKTIP | 0.033627 | 1.453571 | 1.029393 | 2.052539 |
| OR51L1 | 0.033649 | 1.056081 | 1.004229 | 1.11061 |
| FOXS1 | 0.03368 | 1.151936 | 1.010977 | 1.31255 |
| ENSG00000183292 | 0.033721 | 1.046062 | 1.003472 | 1.09046 |
| FAM83H | 0.03378 | 0.845835 | 0.724678 | 0.987247 |
| PPM1J | 0.033806 | 0.838332 | 0.712349 | 0.986596 |
| RDH13 | 0.033813 | 0.753015 | 0.579469 | 0.978536 |
| DNM3 | 0.033875 | 1.169669 | 1.012008 | 1.351891 |
| BORA | 0.033891 | 0.801427 | 0.653194 | 0.983299 |
| ZC3H12C | 0.033942 | 1.170339 | 1.011997 | 1.353455 |
| PIK3C2B | 0.033948 | 0.733869 | 0.551343 | 0.976822 |
| CALB2 | 0.033999 | 1.071957 | 1.005262 | 1.143077 |
| MMP13 | 0.03402 | 1.06863 | 1.005018 | 1.136269 |
| ALDH1L2 | 0.034052 | 1.167973 | 1.011753 | 1.348315 |
| KIT | 0.034122 | 1.122764 | 1.00871 | 1.249715 |
| MAGEE1 | 0.034131 | 1.128273 | 1.009074 | 1.261554 |
| ADD2 | 0.034135 | 1.100046 | 1.00716 | 1.201498 |
| SYT9 | 0.034156 | 1.066444 | 1.004818 | 1.131849 |
| OR5P3 | 0.034166 | 1.042216 | 1.003092 | 1.082865 |
| SYPL2 | 0.034221 | 1.11178 | 1.007913 | 1.226352 |
| SLCO1C1 | 0.03423 | 1.138751 | 1.009705 | 1.284291 |
| SYNDIG1 | 0.034281 | 1.118398 | 1.008323 | 1.24049 |
| ADAMTS2 | 0.034289 | 1.136909 | 1.009544 | 1.280342 |
| MSL2 | 0.03429 | 0.654754 | 0.442355 | 0.969138 |
| RBP7 | 0.034357 | 1.139035 | 1.009638 | 1.285016 |
| THRB | 0.034389 | 1.115653 | 1.008078 | 1.234708 |
| KRTAP13-4 | 0.034423 | 1.062952 | 1.004487 | 1.124819 |
| RAB3IL1 | 0.034426 | 1.175934 | 1.011954 | 1.366485 |
| DGKQ | 0.03443 | 0.770659 | 0.605366 | 0.981086 |
| SLC6A9 | 0.03444 | 0.815367 | 0.674838 | 0.98516 |
| PRR35 | 0.034453 | 1.04197 | 1.003013 | 1.082439 |
| LRFN1 | 0.034476 | 1.115651 | 1.008029 | 1.234764 |
| COL12A1 | 0.03451 | 1.147461 | 1.010077 | 1.303531 |
| KIAA0408 | 0.034526 | 1.049632 | 1.003533 | 1.097848 |
| OR5K4 | 0.034527 | 1.062406 | 1.004417 | 1.123743 |
| ANKRD22 | 0.034563 | 0.887875 | 0.79516 | 0.991401 |
| ROBO4 | 0.034602 | 1.227143 | 1.014934 | 1.483722 |
| MAMDC2 | 0.034607 | 1.076272 | 1.005335 | 1.152213 |
| ZNF684 | 0.034632 | 0.752571 | 0.578118 | 0.979667 |
| VPS53 | 0.034647 | 0.681554 | 0.477551 | 0.972703 |
| GALNT10 | 0.034668 | 1.288548 | 1.018442 | 1.63029 |
| CSF1R | 0.034724 | 1.150305 | 1.010104 | 1.309965 |
| SCHIP1 | 0.034724 | 1.138878 | 1.00938 | 1.284989 |
| ZNF500 | 0.034738 | 0.682224 | 0.478371 | 0.972946 |
| CHST14 | 0.034742 | 1.295485 | 1.018736 | 1.647415 |
| AMBP | 0.034744 | 1.081513 | 1.005634 | 1.163119 |
| SSBP2 | 0.03475 | 1.146282 | 1.009831 | 1.301171 |
| C14orf183 | 0.034757 | 0.960831 | 0.925841 | 0.997142 |
| LCE1F | 0.03479 | 1.04907 | 1.003429 | 1.096787 |
| SLAIN1 | 0.03484 | 1.128893 | 1.00867 | 1.263447 |
| TCEAL2 | 0.034847 | 1.045143 | 1.003147 | 1.088896 |
| ALG1L2 | 0.034891 | 0.91157 | 0.836435 | 0.993453 |
| CUL4A | 0.03494 | 0.682418 | 0.478445 | 0.97335 |
| CDK5RAP3 | 0.034943 | 0.713869 | 0.521895 | 0.976461 |
| AAMP | 0.035077 | 0.714997 | 0.523367 | 0.976793 |
| BEX1 | 0.03508 | 1.041186 | 1.002828 | 1.08101 |
| PTPN5 | 0.035085 | 1.090032 | 1.006048 | 1.181027 |
| QPRT | 0.035122 | 1.086574 | 1.005809 | 1.173824 |
| TECTA | 0.035148 | 1.145033 | 1.009475 | 1.298796 |
| PASK | 0.035162 | 0.772621 | 0.60775 | 0.982217 |
| RASA4 | 0.035176 | 1.171279 | 1.011046 | 1.356905 |
| GABPB2 | 0.035209 | 0.785466 | 0.627371 | 0.983401 |
| UBE2QL1 | 0.035239 | 1.108807 | 1.007169 | 1.220701 |
| ANKK1 | 0.035243 | 0.935736 | 0.879632 | 0.995418 |
| CPQ | 0.035292 | 1.215505 | 1.013537 | 1.457719 |
| NRARP | 0.035337 | 0.847456 | 0.726391 | 0.988699 |
| LY96 | 0.035388 | 1.167106 | 1.010627 | 1.347812 |
| FAM9A | 0.035399 | 1.039301 | 1.002638 | 1.077304 |
| PDYN | 0.035411 | 1.042901 | 1.002873 | 1.084527 |
| ZNF564 | 0.035426 | 0.769055 | 0.602135 | 0.982246 |
| RRAGD | 0.035456 | 1.15864 | 1.010073 | 1.329058 |
| OLFML2A | 0.035475 | 1.199029 | 1.012414 | 1.420043 |
| IFNA10 | 0.035487 | 1.060394 | 1.00399 | 1.119968 |
| NEK7 | 0.035505 | 1.421355 | 1.024131 | 1.972647 |
| SALL2 | 0.035515 | 1.10513 | 1.006797 | 1.213067 |
| ZDHHC15 | 0.035534 | 1.108181 | 1.006975 | 1.219558 |
| COL6A3 | 0.035543 | 1.153497 | 1.009703 | 1.317768 |
| CLUH | 0.035647 | 0.757246 | 0.58422 | 0.981515 |
| GOLGA1 | 0.03565 | 0.668389 | 0.458983 | 0.973336 |
| IL1RAPL1 | 0.03571 | 1.084405 | 1.005426 | 1.169589 |
| SLC35F6 | 0.035722 | 0.678562 | 0.472514 | 0.97446 |
| ITGA11 | 0.03573 | 1.134542 | 1.008452 | 1.276396 |
| ACPT | 0.035764 | 0.918266 | 0.848008 | 0.994345 |
| DUSP13 | 0.035781 | 1.042006 | 1.002737 | 1.082814 |
| RRAD | 0.035807 | 1.104068 | 1.006584 | 1.210993 |
| ROS1 | 0.035839 | 1.066021 | 1.004237 | 1.131607 |
| PRG4 | 0.035892 | 1.096469 | 1.006084 | 1.194974 |
| UPK1B | 0.035921 | 1.048227 | 1.0031 | 1.095384 |
| EEF1A2 | 0.035972 | 1.054878 | 1.003503 | 1.108883 |
| PPP1R14A | 0.035987 | 1.108093 | 1.006733 | 1.219658 |
| ACBD5 | 0.036008 | 0.784508 | 0.625281 | 0.984282 |
| ATP13A1 | 0.036018 | 0.694958 | 0.494567 | 0.976543 |
| ZG16 | 0.036054 | 1.052893 | 1.003358 | 1.104874 |
| MAP1LC3B | 0.036112 | 1.467946 | 1.025168 | 2.101963 |
| FAM19A5 | 0.036224 | 1.131347 | 1.007953 | 1.269846 |
| FOLR2 | 0.036285 | 1.118407 | 1.007174 | 1.241924 |
| PNPLA6 | 0.036304 | 0.711797 | 0.517763 | 0.978547 |
| GDF3 | 0.036387 | 1.067859 | 1.004169 | 1.135589 |
| SPAG9 | 0.03641 | 1.411692 | 1.022051 | 1.949879 |
| NUDT22 | 0.036412 | 0.723371 | 0.534093 | 0.979727 |
| KCNA1 | 0.036431 | 1.04281 | 1.002651 | 1.084579 |
| CD248 | 0.036477 | 1.179349 | 1.010434 | 1.376502 |
| FAM120C | 0.03652 | 1.268495 | 1.015025 | 1.585261 |
| GRTP1 | 0.036533 | 0.831273 | 0.699061 | 0.98849 |
| KRTAP5-4 | 0.036589 | 0.964493 | 0.932346 | 0.997748 |
| GLI3 | 0.036606 | 1.126447 | 1.007443 | 1.259509 |
| ZNF558 | 0.036612 | 0.818116 | 0.67773 | 0.987581 |
| PLXNB2 | 0.036623 | 0.764153 | 0.593781 | 0.98341 |
| LRRN1 | 0.036629 | 1.066126 | 1.003988 | 1.13211 |
| TLL1 | 0.036653 | 1.118001 | 1.006944 | 1.241306 |
| TCF4 | 0.036665 | 1.200453 | 1.011389 | 1.424861 |
| NTN4 | 0.036673 | 1.179631 | 1.010286 | 1.377362 |
| TEX26 | 0.036695 | 1.041711 | 1.00253 | 1.082424 |
| NMNAT2 | 0.036708 | 1.100107 | 1.005911 | 1.203125 |
| CCDC184 | 0.036713 | 1.163491 | 1.009393 | 1.341113 |
| SLC37A2 | 0.036721 | 1.157309 | 1.009055 | 1.327345 |
| KLF13 | 0.036759 | 0.74908 | 0.571182 | 0.982385 |
| OR51G2 | 0.036816 | 1.052595 | 1.003143 | 1.104485 |
| NDUFS1 | 0.036849 | 0.643663 | 0.425599 | 0.973455 |
| MAGEA9 | 0.036888 | 1.029156 | 1.001751 | 1.05731 |
| SPATS2L | 0.036908 | 1.361687 | 1.018939 | 1.819728 |
| HIST3H3 | 0.036923 | 0.962158 | 0.927919 | 0.997661 |
| COL4A2 | 0.036943 | 1.196494 | 1.01093 | 1.41612 |
| FAT1 | 0.036947 | 0.810422 | 0.6652 | 0.987348 |
| CAMSAP2 | 0.036951 | 1.375891 | 1.019513 | 1.856844 |
| ARHGAP11B | 0.036979 | 0.81733 | 0.67622 | 0.987886 |
| RAMP2 | 0.037003 | 1.186856 | 1.010383 | 1.394152 |
| SGCD | 0.037024 | 1.111769 | 1.006398 | 1.228172 |
| ITGB3 | 0.037046 | 1.154231 | 1.008656 | 1.320818 |
| BCL2L14 | 0.037148 | 0.888614 | 0.795211 | 0.992989 |
| CHMP4C | 0.037187 | 0.858293 | 0.743381 | 0.990967 |
| RAPSN | 0.037269 | 0.942348 | 0.891135 | 0.996504 |
| GPR152 | 0.037294 | 0.962181 | 0.927896 | 0.997734 |
| PLAGL1 | 0.037299 | 1.152704 | 1.008395 | 1.317664 |
| NAP1L3 | 0.037425 | 1.111813 | 1.006188 | 1.228525 |
| CTF1 | 0.037425 | 1.110516 | 1.00612 | 1.225745 |
| C6orf118 | 0.037441 | 1.037681 | 1.002152 | 1.074469 |
| CTSL | 0.037443 | 1.231017 | 1.012152 | 1.497208 |
| KIAA1549L | 0.037446 | 1.096043 | 1.005343 | 1.194926 |
| STARD3NL | 0.037448 | 1.359513 | 1.018001 | 1.815593 |
| GGCX | 0.037526 | 1.443334 | 1.021403 | 2.039561 |
| HELT | 0.037526 | 1.03804 | 1.002157 | 1.075209 |
| FES | 0.03754 | 1.211989 | 1.011144 | 1.452728 |
| RNPS1 | 0.037553 | 0.64005 | 0.420325 | 0.974636 |
| FAM49A | 0.037554 | 1.160406 | 1.008602 | 1.335059 |
| DCDC2C | 0.037688 | 1.039228 | 1.002192 | 1.077633 |
| OR6C70 | 0.037689 | 1.048476 | 1.002697 | 1.096344 |
| CCDC181 | 0.03769 | 1.142043 | 1.007586 | 1.294443 |
| GLYATL2 | 0.037702 | 1.06504 | 1.003588 | 1.130255 |
| EYA4 | 0.037788 | 1.094991 | 1.005133 | 1.192883 |
| PRTFDC1 | 0.037809 | 1.190543 | 1.00987 | 1.40354 |
| MTPAP | 0.03783 | 0.715628 | 0.521846 | 0.981369 |
| SMO | 0.037848 | 1.132675 | 1.007016 | 1.274014 |
| UQCRHL | 0.037853 | 0.809771 | 0.663536 | 0.988234 |
| ALDH5A1 | 0.037941 | 0.867299 | 0.758192 | 0.992107 |
| GRIK2 | 0.037941 | 1.087725 | 1.004691 | 1.177621 |
| CMA1 | 0.03796 | 1.044322 | 1.002413 | 1.087983 |
| GLRB | 0.037972 | 1.114921 | 1.006057 | 1.235565 |
| TMEM50B | 0.038049 | 1.386201 | 1.018167 | 1.887269 |
| MAGEC3 | 0.038086 | 1.040655 | 1.002192 | 1.080593 |
| SIGLEC5 | 0.038114 | 1.131007 | 1.006771 | 1.270575 |
| FARSA | 0.038121 | 0.725863 | 0.536207 | 0.982602 |
| FRY | 0.038121 | 1.187225 | 1.009446 | 1.396314 |
| ERICH3 | 0.038184 | 1.056672 | 1.003007 | 1.113208 |
| PLBD2 | 0.03821 | 1.46743 | 1.021059 | 2.108938 |
| BNIP3 | 0.038251 | 1.134601 | 1.00686 | 1.278547 |
| RHOJ | 0.038292 | 1.168989 | 1.008458 | 1.355075 |
| MASTL | 0.038345 | 0.75992 | 0.586053 | 0.985369 |
| CRYAB | 0.038351 | 1.098492 | 1.005053 | 1.200619 |
| OR4A15 | 0.038357 | 1.073899 | 1.003831 | 1.148858 |
| HS6ST2 | 0.038371 | 1.065392 | 1.003398 | 1.131216 |
| OLR1 | 0.038386 | 1.117013 | 1.005936 | 1.240355 |
| PLCB3 | 0.038405 | 0.811697 | 0.666231 | 0.988923 |
| TRPC1 | 0.038431 | 1.137947 | 1.006906 | 1.286042 |
| ALDH3A2 | 0.038446 | 0.788424 | 0.629522 | 0.987436 |
| TMEM108 | 0.038577 | 1.151733 | 1.007451 | 1.316677 |
| RFTN2 | 0.038591 | 1.236645 | 1.01121 | 1.512337 |
| RP11-468E2.6 | 0.038623 | 0.96368 | 0.930478 | 0.998066 |
| RAVER2 | 0.03865 | 0.776679 | 0.61124 | 0.986896 |
| C11orf86 | 0.038657 | 1.049888 | 1.002542 | 1.09947 |
| STAB1 | 0.038681 | 1.190786 | 1.009129 | 1.405143 |
| FGFR1 | 0.03872 | 1.132806 | 1.006486 | 1.274979 |
| FBLN2 | 0.038735 | 1.112678 | 1.005544 | 1.231226 |
| HIST1H2BM | 0.038738 | 0.956163 | 0.916372 | 0.997682 |
| PLS3 | 0.038748 | 1.280238 | 1.012858 | 1.618202 |
| FZD4 | 0.038803 | 1.22548 | 1.010516 | 1.486173 |
| SP5 | 0.038806 | 0.923654 | 0.856629 | 0.995923 |
| STX10 | 0.038816 | 0.728423 | 0.53931 | 0.98385 |
| SSC5D | 0.038828 | 1.099724 | 1.004891 | 1.203506 |
| PTCD3 | 0.038829 | 0.691871 | 0.487821 | 0.981273 |
| SLC22A5 | 0.038839 | 0.725809 | 0.535526 | 0.983703 |
| OR4S2 | 0.038869 | 1.06487 | 1.003219 | 1.13031 |
| TMEM179 | 0.038907 | 1.04343 | 1.002168 | 1.086391 |
| TM6SF1 | 0.038907 | 1.152924 | 1.007275 | 1.319632 |
| LGALS1 | 0.038909 | 1.165919 | 1.007849 | 1.34878 |
| SUGP1 | 0.038978 | 0.576706 | 0.341983 | 0.972535 |
| MS4A4A | 0.039022 | 1.142822 | 1.006749 | 1.297287 |
| GPRASP1 | 0.039114 | 1.113576 | 1.005387 | 1.233408 |
| AATK | 0.039208 | 0.897911 | 0.810552 | 0.994686 |
| CTSF | 0.039243 | 1.136928 | 1.006348 | 1.284451 |
| ERCC6L | 0.039292 | 0.841738 | 0.714539 | 0.991581 |
| CALN1 | 0.039344 | 1.054358 | 1.002588 | 1.108802 |
| MYF5 | 0.039363 | 1.057604 | 1.002733 | 1.115478 |
| BMPR1B | 0.039368 | 1.082904 | 1.003887 | 1.168141 |
| ZNF365 | 0.039369 | 1.105994 | 1.004919 | 1.217236 |
| HIST1H2BI | 0.039411 | 0.959932 | 0.923298 | 0.998019 |
| TNMD | 0.039491 | 1.041074 | 1.001939 | 1.081738 |
| KRTAP9-7 | 0.039502 | 1.051592 | 1.002421 | 1.103174 |
| SMR3A | 0.039506 | 1.041333 | 1.001948 | 1.082266 |
| TUBB3 | 0.03954 | 1.127274 | 1.005753 | 1.263479 |
| CCBE1 | 0.039558 | 1.109586 | 1.004982 | 1.225078 |
| RPAP2 | 0.03957 | 0.669551 | 0.456966 | 0.981034 |
| HBB | 0.039638 | 1.10009 | 1.004532 | 1.204738 |
| PLN | 0.039697 | 1.071458 | 1.003258 | 1.144294 |
| TET1 | 0.039754 | 1.160684 | 1.007005 | 1.337815 |
| PAQR4 | 0.039789 | 0.840987 | 0.713 | 0.991949 |
| H2BFM | 0.039804 | 1.039837 | 1.001822 | 1.079294 |
| PRDM9 | 0.039812 | 1.038224 | 1.001748 | 1.076028 |
| KLK4 | 0.039835 | 1.039351 | 1.001795 | 1.078316 |
| GTF3C5 | 0.039838 | 0.733798 | 0.546256 | 0.985727 |
| POF1B | 0.039848 | 0.909697 | 0.831191 | 0.995619 |
| SERPINE2 | 0.039877 | 1.124222 | 1.005431 | 1.257049 |
| NOM1 | 0.039973 | 0.699628 | 0.497552 | 0.983776 |
| PRPF3 | 0.040004 | 0.671791 | 0.459573 | 0.982006 |
| S1PR1 | 0.040064 | 1.169981 | 1.007146 | 1.359142 |
| C7 | 0.040068 | 1.057861 | 1.002554 | 1.11622 |
| DCK | 0.040256 | 0.71404 | 0.517543 | 0.985143 |
| FAM24A | 0.040272 | 1.038021 | 1.001657 | 1.075705 |
| CD300LG | 0.040306 | 1.059874 | 1.002574 | 1.12045 |
| CLMP | 0.040332 | 1.10328 | 1.004341 | 1.211966 |
| MAGEA3 | 0.040353 | 1.023554 | 1.001024 | 1.04659 |
| NTRK3 | 0.040427 | 1.084796 | 1.003557 | 1.172613 |
| CD302 | 0.040455 | 1.162947 | 1.006586 | 1.343595 |
| GOLGA2 | 0.040468 | 0.676962 | 0.466108 | 0.9832 |
| GPR114 | 0.04048 | 0.86274 | 0.749101 | 0.993618 |
| TMEM125 | 0.040505 | 0.866971 | 0.756294 | 0.993845 |
| KRTAP4-9 | 0.04051 | 1.051393 | 1.002169 | 1.103036 |
| TRUB2 | 0.04057 | 0.704785 | 0.504239 | 0.98509 |
| TXNDC9 | 0.040626 | 1.444502 | 1.015816 | 2.054099 |
| DNAAF3 | 0.04064 | 0.895661 | 0.805984 | 0.995316 |
| KAZALD1 | 0.04074 | 0.848077 | 0.724244 | 0.993082 |
| CST6 | 0.040834 | 1.062145 | 1.002516 | 1.12532 |
| FILIP1L | 0.040839 | 1.151255 | 1.005885 | 1.317633 |
| F7 | 0.040851 | 1.062447 | 1.002523 | 1.125953 |
| DFNA5 | 0.040887 | 1.134785 | 1.005252 | 1.281009 |
| OR5M3 | 0.040933 | 1.059617 | 1.002389 | 1.120111 |
| XPNPEP3 | 0.04096 | 0.717362 | 0.521679 | 0.986446 |
| RPP14 | 0.040968 | 0.692865 | 0.487347 | 0.985053 |
| AK5 | 0.040989 | 1.113672 | 1.004418 | 1.23481 |
| CFAP221 | 0.041039 | 1.067611 | 1.002667 | 1.136762 |
| ATF7 | 0.041133 | 1.553247 | 1.017888 | 2.370179 |
| PALMD | 0.041236 | 1.148985 | 1.005539 | 1.312893 |
| NRBP2 | 0.041367 | 0.782001 | 0.617442 | 0.990418 |
| S100A5 | 0.041457 | 0.900365 | 0.81396 | 0.995943 |
| MYL9 | 0.041472 | 1.0908 | 1.003366 | 1.185854 |
| NOP14 | 0.041474 | 0.69901 | 0.495424 | 0.986256 |
| NDN | 0.041549 | 1.131024 | 1.004726 | 1.273197 |
| SLC26A6 | 0.041569 | 0.817879 | 0.674083 | 0.992349 |
| CFL2 | 0.041729 | 1.123517 | 1.004371 | 1.256799 |
| TUSC5 | 0.041731 | 1.045402 | 1.001664 | 1.09105 |
| TLR7 | 0.04177 | 1.116275 | 1.004106 | 1.240974 |
| COL2A1 | 0.041833 | 1.045314 | 1.001639 | 1.090894 |
| TSPAN18 | 0.041868 | 1.130445 | 1.004521 | 1.272154 |
| ZNF334 | 0.04188 | 1.078765 | 1.002789 | 1.160498 |
| AMPH | 0.04189 | 1.129332 | 1.004472 | 1.269712 |
| SYT3 | 0.04189 | 1.08414 | 1.002968 | 1.17188 |
| SAMD14 | 0.041939 | 1.202706 | 1.006752 | 1.436801 |
| RP11-38C17.1 | 0.04194 | 1.119997 | 1.00414 | 1.249222 |
| BRCC3 | 0.041962 | 0.722128 | 0.527676 | 0.988236 |
| PDHA2 | 0.041976 | 1.041029 | 1.00146 | 1.082162 |
| GNB1L | 0.042039 | 0.780149 | 0.614095 | 0.991105 |
| NDC1 | 0.042081 | 0.787042 | 0.624767 | 0.991465 |
| CXorf38 | 0.042106 | 0.733478 | 0.543973 | 0.989003 |
| AKR1C2 | 0.042125 | 1.074566 | 1.002562 | 1.151741 |
| ANXA11 | 0.042139 | 0.763353 | 0.588324 | 0.990455 |
| FNDC4 | 0.04218 | 1.156223 | 1.005141 | 1.330014 |
| UGT2B10 | 0.042199 | 1.033933 | 1.001177 | 1.06776 |
| ARHGEF17 | 0.042206 | 1.193954 | 1.00626 | 1.416657 |
| RASA1 | 0.042307 | 1.405983 | 1.011905 | 1.953532 |
| BACE1 | 0.042353 | 1.248895 | 1.007701 | 1.54782 |
| WNT7A | 0.042366 | 1.047219 | 1.001591 | 1.094926 |
| CACNA2D3 | 0.042412 | 1.110613 | 1.003599 | 1.229038 |
| SOCS7 | 0.042624 | 0.757344 | 0.578895 | 0.990801 |
| NOTCH3 | 0.042695 | 1.203769 | 1.006124 | 1.44024 |
| ENSG00000234857 | 0.042778 | 0.81078 | 0.661866 | 0.993199 |
| RND1 | 0.042795 | 1.138048 | 1.004206 | 1.289729 |
| AGAP5 | 0.042802 | 0.851705 | 0.729186 | 0.994809 |
| CRYBB2 | 0.042869 | 0.956727 | 0.916628 | 0.998581 |
| ZNF423 | 0.042878 | 1.125746 | 1.003805 | 1.262501 |
| CYP4V2 | 0.042962 | 0.812169 | 0.66398 | 0.993432 |
| HPR | 0.043016 | 1.066416 | 1.002023 | 1.134947 |
| FGL1 | 0.043041 | 1.043202 | 1.001325 | 1.086831 |
| HRH4 | 0.043055 | 1.069332 | 1.002097 | 1.141079 |
| ATP8A2 | 0.043071 | 1.121615 | 1.003584 | 1.253529 |
| SLIT3 | 0.043091 | 1.110663 | 1.003267 | 1.229556 |
| CLEC14A | 0.043113 | 1.196413 | 1.00557 | 1.423475 |
| ENSG00000265118 | 0.043123 | 1.053372 | 1.001609 | 1.107809 |
| NME7 | 0.04314 | 1.324111 | 1.008698 | 1.73815 |
| ASTL | 0.043163 | 0.947652 | 0.899529 | 0.998348 |
| CXXC4 | 0.043183 | 1.110682 | 1.003223 | 1.229651 |
| LGR6 | 0.043215 | 0.93617 | 0.878179 | 0.99799 |
| SNCAIP | 0.043217 | 1.090863 | 1.002656 | 1.18683 |
| TBATA | 0.043224 | 1.041074 | 1.001227 | 1.082506 |
| GOLGA8T | 0.043238 | 1.040727 | 1.001214 | 1.081798 |
| SMARCA4 | 0.043258 | 0.736337 | 0.547243 | 0.990769 |
| DPP3 | 0.04329 | 0.770687 | 0.598642 | 0.992177 |
| CRABP2 | 0.04338 | 1.079633 | 1.002281 | 1.162954 |
| POLA2 | 0.043393 | 0.741912 | 0.555331 | 0.991181 |
| APLNR | 0.043422 | 1.148829 | 1.004107 | 1.31441 |
| JAKMIP2 | 0.04343 | 1.124591 | 1.00347 | 1.26033 |
| PHYHD1 | 0.043454 | 1.116011 | 1.003232 | 1.241468 |
| CDC42BPG | 0.04346 | 0.852323 | 0.729871 | 0.995319 |
| CYP27C1 | 0.043476 | 1.103505 | 1.002889 | 1.214216 |
| NACAD | 0.043486 | 1.113182 | 1.003141 | 1.235294 |
| GPR98 | 0.043488 | 1.080334 | 1.002262 | 1.164489 |
| C1QL3 | 0.043496 | 0.917361 | 0.843674 | 0.997485 |
| TTC12 | 0.043526 | 0.772656 | 0.601489 | 0.992533 |
| ATP11C | 0.04353 | 1.303658 | 1.007731 | 1.686485 |
| RALGDS | 0.043541 | 0.716315 | 0.518095 | 0.990374 |
| CTRC | 0.043545 | 0.944842 | 0.894196 | 0.998358 |
| THEG | 0.043556 | 1.037958 | 1.001078 | 1.076196 |
| GPR157 | 0.043592 | 0.794743 | 0.635802 | 0.993416 |
| ENSG00000279624 | 0.043604 | 0.952435 | 0.908402 | 0.998602 |
| CYP46A1 | 0.043663 | 1.140225 | 1.003737 | 1.295272 |
| DEGS2 | 0.043668 | 0.911026 | 0.832169 | 0.997357 |
| CYP4X1 | 0.043686 | 0.914709 | 0.838808 | 0.997478 |
| CSRNP1 | 0.043743 | 1.225732 | 1.005728 | 1.493863 |
| CH25H | 0.043763 | 1.113976 | 1.003023 | 1.237203 |
| ENSG00000258555 | 0.043856 | 0.944178 | 0.892883 | 0.99842 |
| ZNF74 | 0.043867 | 0.778424 | 0.61013 | 0.993138 |
| BTBD19 | 0.04388 | 1.190731 | 1.004799 | 1.411067 |
| ABRA | 0.043907 | 0.945824 | 0.895944 | 0.99848 |
| PABPC5 | 0.043941 | 1.120656 | 1.003097 | 1.251993 |
| LRFN4 | 0.043945 | 0.848052 | 0.722415 | 0.995539 |
| SCG5 | 0.043976 | 1.139197 | 1.003523 | 1.293214 |
| VKORC1 | 0.043979 | 1.275087 | 1.006576 | 1.615225 |
| PYY | 0.044013 | 1.063745 | 1.001658 | 1.129679 |
| TUBB2A | 0.044065 | 1.196 | 1.004768 | 1.423627 |
| XG | 0.044066 | 1.073275 | 1.001881 | 1.149757 |
| CDH19 | 0.044074 | 1.051414 | 1.001331 | 1.104002 |
| PRRX1 | 0.044084 | 1.11598 | 1.002911 | 1.241797 |
| ACADL | 0.044135 | 1.061419 | 1.001566 | 1.124848 |
| FABP3 | 0.044239 | 1.130684 | 1.003171 | 1.274405 |
| ZCCHC16 | 0.044239 | 1.042411 | 1.001071 | 1.085459 |
| AR | 0.044239 | 1.093545 | 1.002308 | 1.193088 |
| RGP1 | 0.044261 | 0.725748 | 0.531063 | 0.991804 |
| DFFB | 0.044268 | 0.762206 | 0.585018 | 0.993061 |
| RIN1 | 0.044288 | 0.857967 | 0.738994 | 0.996093 |
| OR13G1 | 0.04434 | 1.052331 | 1.001292 | 1.105973 |
| GCDH | 0.044345 | 0.683094 | 0.471137 | 0.990408 |
| KHK | 0.044376 | 0.852727 | 0.730062 | 0.996002 |
| CARD11 | 0.044422 | 1.111391 | 1.002637 | 1.23194 |
| EIF2AK4 | 0.044425 | 1.503204 | 1.01021 | 2.236785 |
| PRDM6 | 0.044432 | 1.118884 | 1.0028 | 1.248407 |
| PGF | 0.044444 | 1.196816 | 1.004472 | 1.425991 |
| DEPDC5 | 0.044445 | 0.680269 | 0.467214 | 0.990479 |
| THEM5 | 0.044519 | 0.925422 | 0.858032 | 0.998104 |
| USP17L3 | 0.044613 | 1.080899 | 1.001873 | 1.166157 |
| SLC1A5 | 0.044648 | 0.847485 | 0.721078 | 0.996053 |
| PLAC8L1 | 0.04467 | 0.910583 | 0.831013 | 0.997773 |
| TBX15 | 0.044802 | 1.120482 | 1.002642 | 1.25217 |
| TCF7L1 | 0.04483 | 1.1562 | 1.003354 | 1.33233 |
| DLG4 | 0.044877 | 1.190933 | 1.004002 | 1.41267 |
| EFNA4 | 0.044891 | 0.826441 | 0.685978 | 0.995665 |
| AOX1 | 0.044908 | 1.086274 | 1.001881 | 1.177774 |
| CEPT1 | 0.044926 | 0.677584 | 0.463182 | 0.991231 |
| AASS | 0.044933 | 1.166905 | 1.003494 | 1.356924 |
| PDCD1 | 0.044936 | 0.895546 | 0.804003 | 0.997512 |
| FGF5 | 0.044948 | 1.04932 | 1.001085 | 1.09988 |
| ATP1B2 | 0.044948 | 1.116386 | 1.002483 | 1.24323 |
| ATG2A | 0.044955 | 0.720594 | 0.523098 | 0.992654 |
| PSPC1 | 0.045046 | 0.673333 | 0.457355 | 0.991303 |
| ALG10B | 0.045061 | 1.179379 | 1.003639 | 1.385892 |
| AP005242.1 | 0.045062 | 1.05793 | 1.00124 | 1.117829 |
| SETBP1 | 0.045122 | 1.113657 | 1.002343 | 1.237332 |
| GNA12 | 0.045141 | 1.390032 | 1.007155 | 1.91846 |
| MAVS | 0.045153 | 0.756031 | 0.575046 | 0.993979 |
| COX18 | 0.045233 | 0.719006 | 0.520603 | 0.99302 |
| EYA3 | 0.045255 | 0.676026 | 0.460808 | 0.99176 |
| ANKRD7 | 0.045269 | 1.040268 | 1.000832 | 1.081258 |
| DAZ2 | 0.045273 | 1.036137 | 1.000748 | 1.072778 |
| NECAB1 | 0.045289 | 1.093866 | 1.001884 | 1.194293 |
| LRRC8C | 0.045334 | 1.250288 | 1.004651 | 1.555984 |
| RNF175 | 0.04535 | 1.149431 | 1.002887 | 1.317388 |
| PAGE4 | 0.045352 | 1.034882 | 1.00071 | 1.070221 |
| TMEM88 | 0.045361 | 1.152345 | 1.002932 | 1.324017 |
| CASP2 | 0.045371 | 0.719356 | 0.520997 | 0.993236 |
| SKIL | 0.045391 | 1.260163 | 1.004755 | 1.580495 |
| ASB9 | 0.045471 | 1.158781 | 1.002974 | 1.338791 |
| SLC11A2 | 0.045486 | 0.740032 | 0.550968 | 0.993972 |
| OR11H12 | 0.045487 | 1.044956 | 1.000883 | 1.090969 |
| RXRA | 0.045511 | 0.745329 | 0.558786 | 0.994147 |
| PSG5 | 0.045588 | 1.03905 | 1.000752 | 1.078814 |
| TSPAN17 | 0.04563 | 0.721731 | 0.524206 | 0.993684 |
| EPB41L3 | 0.045635 | 1.156153 | 1.00282 | 1.33293 |
| TNFRSF14 | 0.04566 | 0.797512 | 0.638807 | 0.995644 |
| NLGN4X | 0.045691 | 1.088266 | 1.001621 | 1.182406 |
| SPNS2 | 0.045774 | 0.903446 | 0.817772 | 0.998095 |
| ITGA1 | 0.045777 | 1.16192 | 1.00282 | 1.346261 |
| GK5 | 0.045836 | 0.773992 | 0.601909 | 0.995272 |
| IKBKB | 0.045842 | 0.712143 | 0.510337 | 0.99375 |
| VIPAS39 | 0.045844 | 1.691541 | 1.009751 | 2.833678 |
| ZSCAN12 | 0.045855 | 1.23833 | 1.003944 | 1.527438 |
| IMPAD1 | 0.045909 | 1.441482 | 1.006665 | 2.064114 |
| AC008074.1 | 0.045979 | 0.874039 | 0.765782 | 0.9976 |
| PTPN12 | 0.045997 | 1.358929 | 1.005465 | 1.836653 |
| NR4A3 | 0.046016 | 1.11921 | 1.001994 | 1.250139 |
| PPP1R3D | 0.046016 | 0.802348 | 0.646274 | 0.996113 |
| PCDHA13 | 0.04603 | 1.04302 | 1.000742 | 1.087083 |
| CAPN11 | 0.046132 | 1.128399 | 1.002075 | 1.270648 |
| LRRC20 | 0.046197 | 0.791606 | 0.629115 | 0.996067 |
| PPAP2A | 0.046223 | 1.20876 | 1.00318 | 1.456468 |
| TRHDE | 0.046296 | 1.072514 | 1.00115 | 1.148966 |
| P2RY11 | 0.04631 | 0.79715 | 0.637809 | 0.996299 |
| GLS2 | 0.046362 | 0.883136 | 0.781493 | 0.997998 |
| L3MBTL3 | 0.046362 | 1.252927 | 1.003642 | 1.56413 |
| RPS6KA2 | 0.046425 | 1.23883 | 1.003398 | 1.529502 |
| ABCA4 | 0.046451 | 1.108214 | 1.001617 | 1.226156 |
| MEX3B | 0.046481 | 1.188849 | 1.0027 | 1.409556 |
| PAIP2B | 0.046497 | 1.117915 | 1.001731 | 1.247574 |
| ZNF440 | 0.046503 | 0.780161 | 0.610997 | 0.996162 |
| KIF3C | 0.046509 | 1.168149 | 1.002406 | 1.361296 |
| FAM220A | 0.046517 | 1.443436 | 1.005679 | 2.071744 |
| TBC1D19 | 0.046526 | 1.247098 | 1.003403 | 1.549979 |
| OR5H14 | 0.046542 | 1.062681 | 1.000931 | 1.128241 |
| ADORA3 | 0.046589 | 1.133659 | 1.001896 | 1.282751 |
| NDRG4 | 0.046652 | 1.146575 | 1.002029 | 1.311972 |
| IL6 | 0.046705 | 1.083664 | 1.001172 | 1.172953 |
| BNC2 | 0.04673 | 1.101162 | 1.001395 | 1.210869 |
| P4HA2 | 0.046735 | 1.30429 | 1.003846 | 1.694656 |
| EFHD2 | 0.046756 | 0.796014 | 0.635717 | 0.996731 |
| PCOLCE | 0.046788 | 1.153451 | 1.002031 | 1.327754 |
| SOX9 | 0.046819 | 0.851839 | 0.727269 | 0.997746 |
| ADAM33 | 0.04683 | 1.078738 | 1.001063 | 1.162439 |
| HSD17B14 | 0.046883 | 1.1302 | 1.001689 | 1.275199 |
| FAM69B | 0.046896 | 1.149525 | 1.001915 | 1.318883 |
| YBX1 | 0.046942 | 0.699622 | 0.491841 | 0.995182 |
| CTSV | 0.046974 | 1.1093 | 1.001388 | 1.22884 |
| CYB5B | 0.046996 | 0.783694 | 0.616167 | 0.996769 |
| COPS7B | 0.047117 | 0.653495 | 0.429376 | 0.994598 |
| GPR162 | 0.04712 | 1.168825 | 1.001987 | 1.363442 |
| NECAB2 | 0.047175 | 1.083128 | 1.000997 | 1.171999 |
| CCDC42 | 0.047184 | 1.048537 | 1.00059 | 1.098782 |
| YY1 | 0.047216 | 1.756087 | 1.006946 | 3.062568 |
| OR2G2 | 0.047219 | 1.040102 | 1.000483 | 1.08129 |
| EYA1 | 0.047236 | 1.077394 | 1.00091 | 1.159723 |
| CCDC141 | 0.047259 | 1.108363 | 1.001246 | 1.226941 |
| MGAT5 | 0.047272 | 0.799875 | 0.641524 | 0.997315 |
| CLEC2L | 0.047306 | 1.049972 | 1.00058 | 1.101803 |
| QRICH1 | 0.047322 | 0.594854 | 0.35603 | 0.99388 |
| ATP8B2 | 0.047397 | 1.150042 | 1.001607 | 1.320475 |
| AFM | 0.047429 | 1.034739 | 1.000387 | 1.07027 |
| NUDT10 | 0.047436 | 1.065227 | 1.000715 | 1.133897 |
| CDH5 | 0.047442 | 1.202613 | 1.002084 | 1.44327 |
| KLF16 | 0.047504 | 0.795482 | 0.634387 | 0.997484 |
| OR2AE1 | 0.047587 | 0.965875 | 0.933259 | 0.999631 |
| FAM104A | 0.047616 | 0.635069 | 0.405242 | 0.99524 |
| RBBP9 | 0.047727 | 0.745141 | 0.556873 | 0.997059 |
| YPEL5 | 0.047761 | 1.395791 | 1.003294 | 1.941835 |
| MAP3K10 | 0.0479 | 0.777269 | 0.605557 | 0.997673 |
| RBM10 | 0.047924 | 0.646455 | 0.419573 | 0.996022 |
| ZNF253 | 0.047925 | 0.827347 | 0.685688 | 0.99827 |
| CA6 | 0.048052 | 0.962649 | 0.926995 | 0.999674 |
| HLA-G | 0.048059 | 1.111124 | 1.0009 | 1.233487 |
| GMPPA | 0.048062 | 0.751546 | 0.566198 | 0.997568 |
| WHSC1 | 0.048081 | 0.746977 | 0.559351 | 0.99754 |
| KLF5 | 0.04812 | 0.87525 | 0.766907 | 0.998899 |
| ADAMTSL3 | 0.048151 | 1.096402 | 1.000749 | 1.201199 |
| PDIK1L | 0.048161 | 0.748815 | 0.562037 | 0.997663 |
| PGAM5 | 0.048201 | 0.774036 | 0.600347 | 0.997976 |
| CDC42EP3 | 0.04829 | 1.131903 | 1.000932 | 1.280012 |
| IMMT | 0.048313 | 0.615635 | 0.380372 | 0.996409 |
| AGPAT1 | 0.04836 | 1.344613 | 1.002136 | 1.80413 |
| RPS4Y1 | 0.048364 | 1.031255 | 1.000221 | 1.063252 |
| PRSS1 | 0.048364 | 1.044559 | 1.000313 | 1.090761 |
| HIF1A | 0.048429 | 1.27513 | 1.001679 | 1.623232 |
| DENND2C | 0.048441 | 1.134651 | 1.000866 | 1.28632 |
| ADCY6 | 0.048477 | 0.77067 | 0.594968 | 0.998259 |
| GUCY1A3 | 0.048489 | 1.111779 | 1.000703 | 1.235184 |
| PRTN3 | 0.048503 | 1.036882 | 1.000238 | 1.074869 |
| CCDC94 | 0.048548 | 0.737751 | 0.545333 | 0.998063 |
| NDUFA4L2 | 0.048565 | 1.151415 | 1.000888 | 1.324579 |
| OR1S2 | 0.048588 | 1.045861 | 1.000278 | 1.093522 |
| TOE1 | 0.048653 | 0.696733 | 0.486475 | 0.997866 |
| GMEB2 | 0.048656 | 0.72165 | 0.521781 | 0.998078 |
| WDR4 | 0.048662 | 0.748359 | 0.560995 | 0.998299 |
| C11orf44 | 0.048728 | 1.043037 | 1.000235 | 1.087671 |
| BRINP1 | 0.048752 | 1.05496 | 1.000293 | 1.112615 |
| PROK1 | 0.048758 | 1.040302 | 1.000215 | 1.081996 |
| GAGE12J | 0.04884 | 1.03741 | 1.000187 | 1.076019 |
| NOXO1 | 0.048875 | 0.959049 | 0.919965 | 0.999794 |
| TSC22D3 | 0.048932 | 1.147813 | 1.000646 | 1.316624 |
| TSPAN5 | 0.048968 | 1.16168 | 1.000678 | 1.348586 |
| SDCCAG3 | 0.049009 | 0.716025 | 0.513436 | 0.99855 |
| FOXO6 | 0.049009 | 0.90424 | 0.818007 | 0.999563 |
| LRRC17 | 0.049098 | 1.124916 | 1.000465 | 1.264848 |
| OPN1SW | 0.049112 | 1.199892 | 1.000709 | 1.438721 |
| GPR83 | 0.049178 | 0.916004 | 0.839328 | 0.999684 |
| UNC80 | 0.049222 | 1.071906 | 1.000237 | 1.148711 |
| LCE2B | 0.049281 | 1.059507 | 1.000182 | 1.12235 |
| TUBG2 | 0.049314 | 1.228184 | 1.000617 | 1.507506 |
| GPR15 | 0.049318 | 1.052899 | 1.000154 | 1.108426 |
| C11orf63 | 0.049322 | 1.134905 | 1.000375 | 1.287526 |
| FAM98C | 0.049433 | 0.759302 | 0.576933 | 0.999317 |
| ATP2B3 | 0.04944 | 1.05847 | 1.000139 | 1.120204 |
| ATP1B4 | 0.049447 | 1.046951 | 1.000111 | 1.095985 |
| SLC38A5 | 0.049462 | 1.083218 | 1.000188 | 1.17314 |
| CHL1 | 0.04954 | 1.098184 | 1.000188 | 1.20578 |
| MGARP | 0.049572 | 1.077758 | 1.00014 | 1.1614 |
| CLIC4 | 0.049583 | 1.178516 | 1.0003 | 1.388483 |
| PPEF1 | 0.049607 | 1.13837 | 1.000223 | 1.295597 |
| MED12L | 0.049647 | 1.103477 | 1.000152 | 1.217476 |
| AADAC | 0.049661 | 1.057672 | 1.000083 | 1.118576 |
| SEC61G | 0.049701 | 1.17328 | 1.000209 | 1.376298 |
| ABCB11 | 0.049706 | 1.054009 | 1.000067 | 1.11086 |
| BRIP1 | 0.049723 | 0.832442 | 0.693114 | 0.999778 |
| CROCC | 0.049748 | 0.781916 | 0.611558 | 0.999729 |
| PTRF | 0.049756 | 1.140653 | 1.00014 | 1.300907 |
| TRMT2B | 0.049782 | 0.719306 | 0.517563 | 0.999687 |
| RAB8B | 0.049872 | 1.289535 | 1.000142 | 1.662663 |
| CELA3A | 0.049936 | 1.035902 | 1.00001 | 1.073083 |
| NOP9 | 0.049977 | 0.70624 | 0.498792 | 0.999966 |
| POLR3H | 0.049998 | 0.674952 | 0.455562 | 0.999997 |
