# Supplementary material for: Identification and validation of critical alternative splicing events and splicing factors in gastric cancer progression
Source: J Cell Mol Med. 2020 Sep 16;24(21):12667–80. doi: 10.1111/jcmm.15835 (PMC7686978; doi:10.1111/jcmm.15835)
Supplement: Supplementary file 10 — Table S4 [file JCMM-24-12667-s010.docx]

Table S4. AS events used for Cox regression modeling in the TCGA database.

| Id | Symbol | Coef | HR | Low 95%CI | High 95%CI | P value |
| --- | --- | --- | --- | --- | --- | --- |
| ID_1019 | C1QC | -17.3314 | 2.97E-08 | 9.95E-16 | 0.887998 | 0.04844 |
| ID_11000 | KIAA1217 | 1.915035 | 6.787177 | 0.876408 | 52.56201 | 0.066706 |
| ID_13261 | RGS10 | -12.5911 | 3.40E-06 | 6.91E-09 | 0.001674 | 6.86E-05 |
| ID_141 | DVL1 | 2.626575 | 13.82633 | 0.724873 | 263.7252 | 0.080799 |
| ID_16064 | MS4A6A | -11.3761 | 1.15E-05 | 9.88E-09 | 0.013313 | 0.00158 |
| ID_16651 | RPS6KA4 | 16.46328 | 14122493 | 3124.204 | 6.38E+10 | 0.000126 |
| ID_17064 | ZDHHC24 | 4.876556 | 131.1782 | 1.698695 | 10129.96 | 0.027886 |
| ID_17140 | LRFN4 | 4.304671 | 74.04485 | 2.616812 | 2095.16 | 0.011603 |
| ID_18886 | SIDT2 | -9.47827 | 7.65E-05 | 9.32E-07 | 0.006279 | 2.50E-05 |
| ID_22073 | TARBP2 | 5.854397 | 348.7645 | 10.72148 | 11345.14 | 0.000983 |
| ID_22959 | RAP1B | -9.14699 | 0.000107 | 5.58E-07 | 0.020352 | 0.000642 |
| ID_24321 | MMAB | 1.789615 | 5.987148 | 1.90216 | 18.84486 | 0.00222 |
| ID_27373 | CTAGE5 | -2.62214 | 0.072648 | 0.012982 | 0.406538 | 0.002841 |
| ID_27882 | ZBTB25 | -2.53478 | 0.079279 | 0.007455 | 0.843054 | 0.035597 |
| ID_28714 | ZC3H14 | -7.94023 | 0.000356 | 3.68E-06 | 0.034503 | 0.000667 |
| ID_30269 | PPIP5K1 | -7.39628 | 0.000614 | 2.19E-05 | 0.017203 | 1.37E-05 |
| ID_30646 | MYO5C | 5.002685 | 148.8122 | 1.702955 | 13003.92 | 0.02828 |
| ID_31728 | CLK3 | 5.6769 | 292.0428 | 0.105258 | 810286.8 | 0.160497 |
| ID_33245 | RNPS1 | -3.32436 | 0.035995 | 0.000721 | 1.798204 | 0.095731 |
| ID_37767 | CMIP | -17.0128 | 4.09E-08 | 2.91E-13 | 0.005742 | 0.004905 |
| ID_3788 | FNBP1L | 3.944889 | 51.67061 | 4.317452 | 618.3859 | 0.00184 |
| ID_39149 | SLC25A35 | -4.93746 | 0.007173 | 0.00019 | 0.270244 | 0.007662 |
| ID_392312 | RPL13 | 1.990906 | 7.322163 | 0.827241 | 64.81067 | 0.073536 |
| ID_39375 | ZNF286A | 4.282116 | 72.39348 | 0.242258 | 21633.22 | 0.140898 |
| ID_39457 | MPRIP | 3.384713 | 29.50952 | 3.155255 | 275.9878 | 0.003004 |
| ID_40840 | THRA | -3.6506 | 0.025976 | 0.002464 | 0.273832 | 0.002383 |
| ID_43628 | METTL23 | -10.227 | 3.62E-05 | 1.00E-07 | 0.013053 | 0.000664 |
| ID_44658 | IMPA2 | 6.470831 | 646.0202 | 0.911055 | 458086.8 | 0.053341 |
| ID_47605 | YIPF2 | -3.67167 | 0.025434 | 0.000926 | 0.698752 | 0.029855 |
| ID_49966 | C19orf54 | -4.67011 | 0.009371 | 0.00068 | 0.129066 | 0.000483 |
| ID_53279 | SRSF7 | -2.25233 | 0.105154 | 0.018298 | 0.604306 | 0.011586 |
| ID_55693 | WDSUB1 | -15.693 | 1.53E-07 | 1.28E-10 | 0.000183 | 1.43E-05 |
| ID_576 | CLSTN1 | -4.71135 | 0.008993 | 0.000625 | 0.12949 | 0.000536 |
| ID_58866 | ENTPD6 | -2.70275 | 0.067021 | 0.005842 | 0.768879 | 0.029925 |
| ID_59223 | ROMO1 | -19.7633 | 2.61E-09 | 7.58E-13 | 9.00E-06 | 1.98E-06 |
| ID_59303 | NDRG3 | -4.50772 | 0.011024 | 0.000111 | 1.094219 | 0.054659 |
| ID_59424 | CHD6 | -12.1132 | 5.49E-06 | 1.69E-09 | 0.017858 | 0.003331 |
| ID_59728 | SULF2 | 3.267268 | 26.23956 | 4.127873 | 166.7964 | 0.000535 |
| ID_60283 | APP | 8.180203 | 3569.581 | 142.7064 | 89287.58 | 6.36E-07 |
| ID_62286 | CBX7 | 5.234603 | 187.6546 | 4.243997 | 8297.422 | 0.006776 |
| ID_62559 | PACSIN2 | 10.49187 | 36021.34 | 564.8995 | 2296934 | 7.46E-07 |
| ID_66531 | UMPS | 15.54277 | 5625238 | 1549.026 | 2.04E+10 | 0.000202 |
| ID_66763 | ATP2C1 | 9.864453 | 19234.35 | 217.9824 | 1697203 | 1.59E-05 |
| ID_66871 | ANAPC13 | -12.3404 | 4.37E-06 | 1.23E-08 | 0.001552 | 3.80E-05 |
| ID_67616 | CLDN11 | -1.27944 | 0.278193 | 0.087707 | 0.882388 | 0.029824 |
| ID_68126 | CCDC50 | 1.60244 | 4.965135 | 0.982946 | 25.08027 | 0.052483 |
| ID_68559 | ZFYVE28 | -2.5018 | 0.081937 | 0.013675 | 0.490954 | 0.006167 |
| ID_69730 | SEC31A | -4.19816 | 0.015023 | 0.003309 | 0.068214 | 5.38E-08 |
| ID_70187 | TET2 | 5.562579 | 260.4938 | 3.362377 | 20181.27 | 0.012198 |
| ID_70697 | USP38 | -8.57617 | 0.000189 | 1.55E-06 | 0.022974 | 0.000466 |
| ID_72865 | ERAP1 | -1.45826 | 0.23264 | 0.033863 | 1.598238 | 0.138053 |
| ID_74616 | THOC3 | -16.5242 | 6.66E-08 | 1.07E-12 | 0.004155 | 0.003353 |
| ID_77424 | HDDC2 | -5.90161 | 0.002735 | 5.75E-05 | 0.129995 | 0.002739 |
| ID_7855 | SHC1 | -3.08162 | 0.045885 | 0.006048 | 0.348125 | 0.002877 |
| ID_80087 | GTF2I | -8.63271 | 0.000178 | 5.74E-06 | 0.005535 | 8.47E-07 |
| ID_84732 | YWHAZ | 8.645001 | 5681.674 | 1.813909 | 17796597 | 0.035295 |
| ID_86508 | CBWD5 | 4.96849 | 143.8096 | 0.75434 | 27416.29 | 0.063635 |
| ID_86675 | TLE1 | -4.59863 | 0.010066 | 0.000251 | 0.404406 | 0.014671 |
| ID_86883 | BICD2 | 3.645857 | 38.31561 | 4.81899 | 304.646 | 0.000568 |
| ID_9103 | ABL2 | 4.684369 | 108.242 | 2.383503 | 4915.591 | 0.016124 |
