# Supplementary material for: Identification and validation of critical alternative splicing events and splicing factors in gastric cancer progression
Source: J Cell Mol Med. 2020 Sep 16;24(21):12667–80. doi: 10.1111/jcmm.15835 (PMC7686978; doi:10.1111/jcmm.15835)
Supplement: Supplementary file 11 — Table S5 [file JCMM-24-12667-s011.docx]

Table S5. Genes with prognostic mRNA levels used for Cox regression modeling in the TCGA database.

| Gene symbol | Coef | HR | Low  95%CI | High 95%CI | P value |
| --- | --- | --- | --- | --- | --- |
| ACKR3 | -0.36524 | 0.694027 | 0.495996 | 0.971124 | 0.033096 |
| ACSS3 | 0.240814 | 1.272284 | 0.980135 | 1.651514 | 0.070418 |
| AJ239318.1 | 0.042524 | 1.043441 | 0.969226 | 1.12334 | 0.258636 |
| AKR1B1 | 0.39202 | 1.479968 | 1.112619 | 1.968603 | 0.00708 |
| AP003419.11 | 0.033216 | 1.033774 | 0.987506 | 1.08221 | 0.155088 |
| APOD | 0.309793 | 1.363143 | 1.184231 | 1.569086 | 1.59E-05 |
| AXL | 0.235561 | 1.265619 | 0.889499 | 1.800779 | 0.190475 |
| C14orf37 | -0.41633 | 0.659461 | 0.438797 | 0.991094 | 0.045177 |
| C1QL2 | 0.047272 | 1.048407 | 0.99869 | 1.100599 | 0.05651 |
| C4orf32 | 0.338798 | 1.40326 | 0.963354 | 2.044044 | 0.077493 |
| CGB | 0.054497 | 1.05601 | 1.01183 | 1.102119 | 0.012444 |
| CYTL1 | 0.137706 | 1.147638 | 0.982527 | 1.340495 | 0.082291 |
| DEFB113 | -0.03679 | 0.963878 | 0.884824 | 1.049995 | 0.399437 |
| ECH1 | 0.867825 | 2.381726 | 1.663799 | 3.409437 | 2.12E-06 |
| ENSG00000264545 | 0.218034 | 1.243629 | 1.128531 | 1.370466 | 1.08E-05 |
| ETV2 | -0.46253 | 0.629691 | 0.456945 | 0.867743 | 0.004698 |
| EVX2 | 0.030254 | 1.030717 | 0.971309 | 1.093758 | 0.317863 |
| FAAH | -0.38517 | 0.680333 | 0.510178 | 0.907238 | 0.008719 |
| FAM83G | -0.20465 | 0.814935 | 0.56513 | 1.175162 | 0.273192 |
| FEM1A | -0.96424 | 0.381274 | 0.220813 | 0.658339 | 0.00054 |
| FGF1 | 0.289275 | 1.335459 | 0.94577 | 1.885714 | 0.100333 |
| GH2 | -0.0415 | 0.959349 | 0.908991 | 1.012497 | 0.131421 |
| GLT8D1 | 0.554234 | 1.740607 | 0.936238 | 3.236051 | 0.079821 |
| GPR173 | 0.500841 | 1.650108 | 1.245284 | 2.186534 | 0.000488 |
| GPR42 | 0.05621 | 1.05782 | 1.004451 | 1.114025 | 0.033329 |
| GPX3 | -0.26639 | 0.766141 | 0.602393 | 0.9744 | 0.029905 |
| INADL | 0.191252 | 1.210764 | 0.819529 | 1.788771 | 0.336822 |
| INCENP | 0.862725 | 2.36961 | 1.389347 | 4.041503 | 0.001539 |
| IQCC | -1.06511 | 0.34469 | 0.208387 | 0.570147 | 3.35E-05 |
| IQCJ | 0.041519 | 1.042393 | 0.978757 | 1.110166 | 0.196408 |
| ITPRIP | 0.623302 | 1.865076 | 1.177952 | 2.953013 | 0.007849 |
| KCND2 | 0.102274 | 1.107687 | 0.941237 | 1.303572 | 0.218314 |
| LDB2 | -0.14657 | 0.863665 | 0.5901 | 1.264051 | 0.450722 |
| LIN7A | -0.19988 | 0.818825 | 0.63162 | 1.061516 | 0.131244 |
| LOX | 0.38243 | 1.465842 | 0.948172 | 2.266142 | 0.085335 |
| MAGEL2 | 0.089275 | 1.093381 | 0.92025 | 1.299084 | 0.310092 |
| MATN3 | -0.13688 | 0.872073 | 0.723161 | 1.051648 | 0.151906 |
| MEF2B | -0.22428 | 0.799092 | 0.687767 | 0.928436 | 0.003389 |
| MKNK2 | 0.391079 | 1.478576 | 0.922501 | 2.369847 | 0.104201 |
| MYL4 | 0.160901 | 1.174568 | 0.964217 | 1.43081 | 0.110031 |
| NGFRAP1 | -0.23613 | 0.789677 | 0.621347 | 1.003611 | 0.053545 |
| NT5E | 0.226934 | 1.254747 | 1.034605 | 1.521731 | 0.021133 |
| OR10A5 | 0.077513 | 1.080596 | 1.029081 | 1.134691 | 0.00187 |
| OSBPL9 | 1.078756 | 2.941018 | 1.496105 | 5.781401 | 0.001759 |
| PDGFRL | -0.64965 | 0.52223 | 0.403712 | 0.675541 | 7.55E-07 |
| PLOD2 | -0.44179 | 0.642886 | 0.452255 | 0.913871 | 0.013822 |
| PTPRD | 0.320845 | 1.378292 | 1.151686 | 1.649486 | 0.000464 |
| SNCG | -0.11798 | 0.888711 | 0.746949 | 1.057378 | 0.183287 |
| SPARC | 0.451807 | 1.571149 | 0.986614 | 2.502 | 0.057015 |
| SRPX2 | -0.33455 | 0.715662 | 0.504673 | 1.014859 | 0.06049 |
| TFPI | -0.3681 | 0.692045 | 0.541789 | 0.883972 | 0.003204 |
| TMEM45A | 0.194041 | 1.214146 | 0.945286 | 1.559477 | 0.128668 |
| TNP1 | -0.04559 | 0.955429 | 0.894219 | 1.02083 | 0.177116 |
| TREML4 | 0.124023 | 1.132041 | 1.063382 | 1.205134 | 0.000102 |
| TRIM25 | -0.5876 | 0.55566 | 0.346517 | 0.891034 | 0.014736 |
| VCAN | -0.61909 | 0.538433 | 0.362684 | 0.799346 | 0.002134 |
| ZBTB7A | 0.691284 | 1.996277 | 1.034768 | 3.851223 | 0.039217 |
| ZFYVE27 | -0.28695 | 0.750548 | 0.462321 | 1.218466 | 0.24576 |
| ZNF101 | -0.64523 | 0.524542 | 0.324548 | 0.847778 | 0.008435 |
| ZNF22 | 0.677401 | 1.968754 | 1.286776 | 3.012174 | 0.001796 |
