# Supplementary material for: Identification and validation of critical alternative splicing events and splicing factors in gastric cancer progression
Source: J Cell Mol Med. 2020 Sep 16;24(21):12667–80. doi: 10.1111/jcmm.15835 (PMC7686978; doi:10.1111/jcmm.15835)
Supplement: Supplementary file 12 — Table S6 [file JCMM-24-12667-s012.docx]

Table S6. Hub AS events screened by co-expression network analysis of prognostic gene AS events and prognostic gene mRNA expression levels.

| AS ID | Gene name | AS | Exons | From exon | To exon | HR |
| --- | --- | --- | --- | --- | --- | --- |
| ID_77020 | MAP3K7 | ES | 11 | 10 | 12 | 0.367271 |
| ID_602 | KIF1B | AT | 52.2 | -- | -- | 0.103703 |
| ID_66014 | CD47 | ES | 8:09:10 | 7 | 11 | 0.031867 |
| ID_66013 | CD47 | ES | 9:10 | 8 | 11 | 0.283083 |
| ID_71387 | SORBS2 | ES | 9.1:9.2 | 8 | 10 | 0.415117 |
| ID_71390 | SORBS2 | ES | 8:9.1:9.2 | 7 | 10 | 0.433024 |
| ID_69731 | SEC31A | ES | 26.1:26.2 | 25.1 | 28 | 0.167238 |
| ID_69730 | SEC31A | ES | 26.1:26.2:27 | 25.1 | 28 | 0.433962 |
| ID_47191 | EVI5L | ES | 12 | 11 | 13 | 2.464947 |
| ID_12641 | SORBS1 | ES | 9.1:9.2 | 8 | 10 | 3.050021 |
| ID_12644 | SORBS1 | ES | 5 | 4 | 6 | 16.36011 |
| ID_88176 | SEC16A | ES | 24:25:00 | 23.12 | 26 | 2.620677 |
| ID_88173 | SEC16A | ES | 25 | 24 | 26 | 6.010263 |
| ID_133752 | CAST | ES | 8.2:9 | 7.1 | 10 | 3.538653 |
| ID_270122 | CAST | ES | 7.1:8.2:9 | 5.2 | 10 | 3.782077 |
| ID_575 | CLSTN1 | ES | 11 | 10 | 12 | 2.796858 |
| ID_576 | CLSTN1 | ES | 3 | 2 | 4 | 2.992609 |
