# Supplementary material for: Identification and validation of critical alternative splicing events and splicing factors in gastric cancer progression
Source: J Cell Mol Med. 2020 Sep 16;24(21):12667–80. doi: 10.1111/jcmm.15835 (PMC7686978; doi:10.1111/jcmm.15835)
Supplement: Supplementary file 13 — Table S7 [file JCMM-24-12667-s013.docx]

Table S7. Splicing factor and spliceosome-related genes whose mRNA levels are significantly associated with prognosis (P<0.05).

| Gene symbol | P value | HR | Low 95%CI | High 95%CI | Type |
| --- | --- | --- | --- | --- | --- |
| DAZAP1 | 0.004228 | 0.550302 | 0.365487 | 0.828571 | Splicing factor |
| QKI | 0.005368 | 1.319072 | 1.085428 | 1.60301 | Splicing factor |
| NOVA1 | 0.016156 | 1.102566 | 1.018244 | 1.193871 | Splicing factor |
| SRRM1 | 0.023414 | 0.704095 | 0.519848 | 0.953644 | Splicing factor |
| HNRNPL | 0.028123 | 0.566806 | 0.34145 | 0.940896 | Splicing factor |
| YBX1 | 0.046942 | 0.699622 | 0.491841 | 0.995182 | Splicing factor |
| CHERP | 0.012698 | 0.616601 | 0.42155 | 0.9019 | Spliceosome |
| PRPF3 | 0.040004 | 0.671791 | 0.459573 | 0.982006 | Spliceosome |
| HNRNPM | 0.0113 | 0.504679 | 0.297333 | 0.85662 | Splicing factor/Spliceosome |
| HNRNPK | 0.030861 | 0.52845 | 0.29616 | 0.942933 | Splicing factor/Spliceosome |
